# Correlation between inflammatory marker and lipid metabolism in patients with uterine leiomyomas

**DOI:** 10.3389/fmed.2023.1124697

**Published:** 2023-05-03

**Authors:** Yanan Duan, Lizhu Guo, Yiqing Peng, Xiuling Shi, Ying Zhao, Kunyan Liu, Runsheng Zhou, Junjie Fu, Cunxu Peng

**Affiliations:** ^1^Jining Medical University, Jining, Shandong Province, China; ^2^Department of Obstetrics and Gynecology, Affiliated Hospital of Jining Medical University, Jining, Shandong, China

**Keywords:** uterine leiomyomas, inflammation marker, lipid metabolism, NLR, triglycerides

## Abstract

**Introduction:**

Obesity is a risk factor for the development of uterine leiomyoma (UL), and the inflammatory response plays a key role in the pathogenesis of UL. Our objective was to assess whether there was an independent relationship between inflammatory markers and triglycerides (TG) in patients with UL.

**Methods:**

1,477 UL participants who were hospitalized at the Jining Medical University between January 2016 and December 2022 were included in this cross-sectional study. The independent and dependent variables measured at baseline were inflammatory markers and TG levels, respectively. The covariates were age, body mass index (BMI), UL and menstrual status. Based on the number of fibroids, the study population was divided into Single-group and Multiple-group.

**Results:**

Univariate and multiple regression analyses and stratified analyses revealed significant positive correlations between neutrophil-lymphocyte ratio and systemic immune inflammation index and TG, and significant negative correlations between monocyte-lymphocyte ratio and TG.

**Conclusion:**

The findings show a significant correlation between the inflammatory response and lipid metabolism levels in UL patients. This provides direction for further research into the pathophysiology of UL and also helps to formulate hypotheses for predictive models of UL.

## 1. Introduction

Uterine leiomyomas (ULs) are the most prevalent benign uterine tumors in women of reproductive age. Increased menstrual bleeding, dysmenorrhea, and infertility are UL symptoms, and they are also amongst the leading causes of hysterectomy (1–3). According to earlier research (4, 5), there may be a strong relation between triglyceride levels and the number of fibroids, and high lipid levels may be a significant risk factor for the formation of UL. Also, there is high expression of lipids in recurrent fibroid tissue. (6) Therefore, it is necessary for us to monitor lipid levels in the management and follow-up of patients with uterine fibroids.

Dyslipidemia, which includes abnormal triglycerides (TG) and cholesterol, is a recognised risk factor for cardiovascular disease (7, 8). A cohort study (6) examined the lipid levels in a population with a first diagnosis of fibroids and recurrent fibroids in order to confirm the involvement of lipids in the pathogenesis of UL. Neutrophil-lymphocyte ratio (NLR), platelet-lymphocyte ratio (PLR), monocyte-lymphocyte ratio (MLR), and systemic immune-inflammation index (SII) are emerging inflammatory markers. PLR is an important indicator of the inflammatory state of the body and a biomarker of the change between the transformed lymphocyte count and the neutrophil count.NLR, like PLR, is a comprehensive response to the change between the body’s defences and inflammatory immunity.MLR, an emerging inflammatory indicator, is easily available and can reflect the inflammatory state of many diseases.SII is an emerging immune marker based on neutrophil, lymphocyte and platelet counts. SII is an emerging immune marker based on neutrophil, lymphocyte and platelet counts. Accumulating evidence (9, 10) suggests that NLR, PLR, MLR, and SII are all useful indicators of the body’s systemic immune and inflammatory status. When tumour cells trigger the body’s immune defence response, this leads to a relative decrease in lymphocytes and a relative increase in platelet count, which causes an increase in peripheral blood PLR values (11). NLR, MLR, and PLR are used to predict poor prognostic outcomes in patients with solid tumours in addition to early diagnosis of solid tumours, and the important role of NLR, MLR and PLR in predicting the prognosis of solid tumours has been mentioned in several recent studies on breast cancer and bile duct cancer. In addition, high SII values are an independent risk factor for many solid tumours and tumour metastases (12, 13). According to new research (14), systemic inflammation is crucial to myofibrosis, disease progression, and the pathogenesis of uterine smooth muscle tumours. The relationship between systemic inflammation and lipids has been demonstrated in a variety of diseases (15, 16), but its relationship in patients with newly diagnosed uterine fibroids has not been clarified. This study investigated whether there was a correlation between inflammatory markers and triglycerides in patients with UL by analysing clinical data from newly diagnosed UL patients.

## 2. Materials and methods

### 2.1. Research design

This cross-sectional study examined the relationship between inflammatory response and TG by using baseline inflammatory markers (Platelet Count, Lymphocyte Count, Neutrophil Count, Monocyte Count, NLR, PLR, MLR, SII) as independent variables and TG levels as dependent variables.

### 2.2. Study population

This study involved UL patients, and information was gathered from the gynecology division of the Affiliated Hospital of Jining Medical University. Information on the healthy population was collected from the Medical Examination Centre of the Affiliated Hospital of Jining Medical University. Data were taken from the hospital’s electronic medical record system without participant identifiers in order to protect patient privacy. It was not necessary to obtain informed consent because the study cohort could be tracked. The study was approved by the hospital’s Institutional Review Board (approval number: 2022C114).

Between January 2016 and December 2022, 10,126 female patients of childbearing age who were diagnosed with uterine fibroids at the Department of Gynecology, Affiliated Hospital of Jining Medical University. Following were the criteria for inclusion: (1) Age 18–59 years, (2) uterine leiomyoma determined by postoperative pathology, (3) uterine leiomyoma determined by auxiliary examination and preoperative clinical manifestation, and (4) first diagnosis of uterine leiomyoma. These criteria were used to exclude people: (1) use of anti-biotics and antithrombotic medications within 3 months of surgery, (2) haematological disorders, malignant tumors, autoimmune disorders, metabolic disorders, hypersplenism, or active infections, (3) use of glucocorticoids, long-term immunoregulatory drugs, or anti-inflammatory drugs, (4) age < 18 years, (5) pregnancy or lactation, and (6) prior use of medication or surgical procedure for uterine leiomyoma. In the end, 1,477 cases in total were included in the study. According to the number of fibroids, the 1,477 study population was split into single and multiple groups.

### 2.3. Variables

Patients’ data, including age, BMI, menstrual status, number and size of lesions, and routine blood indices(TGs, TC, HDL-C, LDL-C, VLDL-C, haemoglobin, platelet count, lymphocyte count, neutrophil count, monocyte count), were collected retrospectively. Anaemia is defined as haemoglobin below 120 g/L. The regularity of menstruation, the amount of menstruation and the absence of dysmenorrhoea are obtained by asking the patient during history taking. The criteria for regular menstruation are a menstrual cycle of 21 to 35 days and a period of 4 to 6 days. Those who meet the criteria are considered regular and those who do not are considered irregular. Menstrual flow is judged to be less than 20 ml, 20-80 ml is normal and over 80 ml is too much. The criteria for anaemia is a haemoglobin of less than 120 g/L. During hospitalisation, the following routine blood indices were obtained. After an 8 h fast, peripheral venous blood was collected and processed in the laboratory. Blood indicators were usually obtained 2 days before the procedure. All measurements were taken in our hospital by laboratory technicians and inspectors. Platelet counts, lymphocyte count, neutrophil count, and monocyte count were measured using the Sysmex XN2000 blood cell analyzer. The sensitivity of the Sysmex XN2000 haematocrit analyzer is described in (17). Triglycerides, total cholesterol, high-density lipoprotein cholesterol, low-density lipoprotein cholesterol, and very low-density lipoprotein cholesterol were measured using a Cobas 8,000 c702 fully automated biochemical immunoassay analyzer. The sensitivity of the Cobas8000 c702 fully automated biochemical immunoassay analyzer can be found in (18). PLR was calculated by dividing the lymphocyte count by the platelet count, NLR by dividing the neutrophil count by the lymphocyte count, MLR by dividing the monocyte count by the lymphocyte count, and SII by multiplying the platelet count by the neutrophil count divided by the lymphocyte count, all using the same blood sample.

This study used the following types of covariates: (1) demographic data, (2) previously published factors affecting variables in this study, and (3) variables chosen based on clinical expertise. As a result, the variables listed below were used to create a fully tuned model: (1) categorical variables, such as menstrual status and the number of lesions (at baseline) and (2) continuous variables, such as age, body mass index (BMI), lesion size, and routine blood indices (obtained at baseline).

### 2.4. Statistical analysis

Continuous variables are presented in two ways: those having normal distribution are presented as the average ± standard deviation (SD). Categorical variables are expressed as frequencies or percentages. We used the χ2 test (categorical variables) and one-way analysis of variance (normal distribution) to analyze differences amongst groups. The data analysis was divided into two steps. In Step 1, univariate and multivariate linear regression analyses were used to build models adjusting for all covariates (age, BMI, maximum diameter of fibroids, menstrual pattern, menstrual flow volume, dysmenorrhea and anaemia). In Step 2, a generalised additive model and smooth curve fitting (punitive spline method) were used to solve the nonlinearity of Platelet Count, Lymphocyte Count, Neutrophil Count, Monocyte Count, NLR, PLR, MLR, SII and TG. All analyses were performed using the R statistical package (http://www.R-project.org *R* Foundation). Statistical significance was set at *p* values <0.05 (double-sided).

## 3. Results

### 3.1. Baseline characteristics of the study population

**Figure 1:**
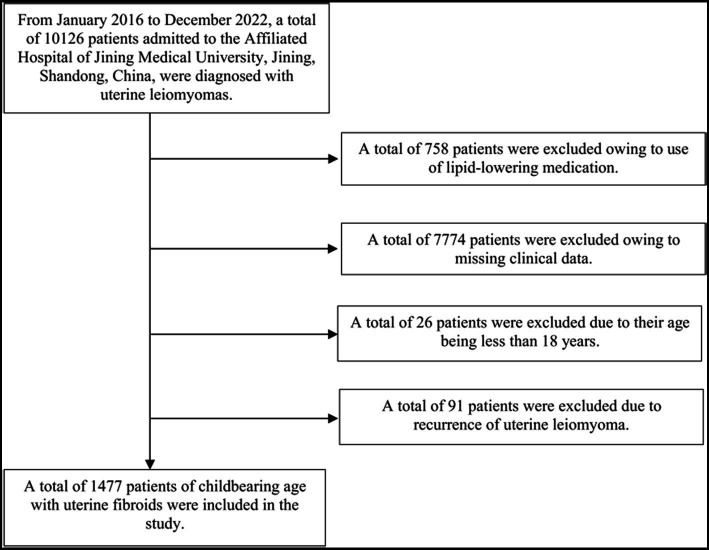
Inclusion and exclusion criteria process.

According to the inclusion criteria, 1,477 individuals were included in the collection of quantitative data (Figure 1). Table 1 displays the baseline characteristics of the chosen subjects. They were divided into single UL (Single) and multiple UL (Multiple) according to the type of disease. The mean age of total participants was 45.49 ± 7.09 years, of which approximately 54.16% had multiple UL. Mean platelet count, lymphocyte count, neutrophil count, monocyte count, PLR, NLR, MLR, SII and TG were 281.44 ± 75.61^*^10^9^/L, 1.83 ± 0.56*10^9^/L, 3.63 ± 1.62^*^10^9^/L, 0.39 ± 0.14^*^10^9^/L, 167.18 ± 68.75, 2.21 ± 1.49, 0.23 ± 0.11, 620.06 ± 446.19, 1145.80 ± 711.79 μmol/L, respectively. In addition, there were no statistical differences in the means of TGs, TC, HDL-C, LDL-C, lymphocyte count, neutrophil count, monocyte count, NLR, MLR and SII between the single and multiple groups.

**Table 1:** Baseline characteristics of the study population.

	Total	Number of fibroids		
Characteristics		Single	Multiple	*p*-value
Quantity, *n*	1477	677	800	
Age (years, mean ± SD)	45.49 ± 7.09	44.24 ± 7.63	46.54 ± 6.42	<0.001
BMI (kg/m^2^, mean ± SD)	24.75 ± 3.32	24.37 ± 3.35	25.06 ± 3.25	<0.001
Maximum diameter of fibroids (cm)	5.57 ± 3.99	5.18 ± 2.78	6.01 ± 4.98	<0.001
Menstrual pattern *n* (%)				0.012
Regularl*y*	1,447 (97.97%)	670 (98.97%)	777 (97.12%)	
Irregularl*y*	30 (2.03%)	7 (1.03%)	23 (2.88%)	
Menstrual flow volume n (%)				<0.001
Light	26 (1.76%)	9 (1.33%)	17 (2.12%)	
Moderate	1,280 (86.66%)	611 (90.25%)	669 (83.62%)	
Heavy	171 (11.58%)	57 (8.42%)	114 (14.25%)	
Dysmenorrhea *n* (%)				0.005
No	1,223 (82.80%)	581 (85.82%)	642 (80.25%)	
Yes	254 (17.20%)	96 (14.18%)	158 (19.75%)	
Anemia *n* (%)				0.098
Yes	848 (57.41%)	373 (55.10%)	475 (59.38%)	
No	629 (42.59%)	304 (44.90%)	325 (40.62%)	
TGs (μmol/L, mean ± SD)	1145.80 ± 711.79	1128.63 ± 743.23	1160.33 ± 684.18	0.394
TC (μmol/L, mean ± SD)	4433.43 ± 879.32	4421.36 ± 903.43	4443.65 ± 858.82	0.628
HDL-C (μmol/L, mean ± SD)	1375.69 ± 301.31	1383.86 ± 303.40	1368.78 ± 299.54	0.338
LDL-C (μmol/L, mean ± SD)	2663.24 ± 703.57	2659.42 ± 725.68	2666.46 ± 684.74	0.848
VLDL-C (μmol/L, mean ± SD)	394.01 ± 255.94	376.87 ± 254.33	408.51 ± 256.56	0.018
Platelet Count (10^9^/L, mean ± SD)	281.44 ± 75.61	275.34 ± 74.09	286.60 ± 76.54	0.004
Lymphocyte Count (10^9^/L, mean ± SD)	1.83 ± 0.56	1.84 ± 0.56	1.82 ± 0.57	0.568
Neutrophil Count (10^9^/L, mean ± SD)	3.63 ± 1.62	3.64 ± 1.67	3.62 ± 1.57	0.777
Monocyte Count (10^9^/L, mean ± SD)	0.39 ± 0.14	0.39 ± 0.14	0.40 ± 0.14	0.313
PLR	167.18 ± 68.75	161.71 ± 64.65	171.80 ± 71.75	0.005
NLR	2.21 ± 1.49	2.19 ± 1.48	2.22 ± 1.51	0.781
MLR	0.23 ± 0.11	0.23 ± 0.10	0.24 ± 0.12	0.077
SII	620.06 ± 446.19	602.13 ± 419.40	635.24 ± 467.39	0.155

UL, Uterine leiomyoma. BMI, body mass index. TGs, triglyceride. TC, total cholesterol. HDL-C, high-density lipoprotein cholesterol. LDL-C, low-density lipoprotein cholesterol. VLDL-C, very low-density lipoprotein cholesterol. NLR, Neutrophil-Lymphocyte Ratio. PLR, Platelet-Lymphocyte Ratio. MLR, Monocyte-Lymphocyte Ratio. SSI, systemic immune-inflammation index.

### 3.2. Univariate analysis of TGs

Table 2 shows the results of the univariate analysis. In the study population, age, BMI, TC, HDL-C, LDL-C, and VLDL-C were associated with TG. Next, lymphocyte count (*β* = 162.32, 95% *CI* = 98.54, 226.09) and neutrophil count (*β* = 74.22, 95% *CI* = 52.06, 96.38) were all positively associated with TG in the total study population. Interestingly, PLR (*β* = −0.76, 95% *CI* = −1.29, −0.23) and MLR (*β* = −394.70, 95% *CI* = −724.64, −64.77) were all negatively correlated with TG. Separate analyses of the study population by single-and multiple-occurrence groups yielded results that were generally consistent with the total study population. Interestingly, the presence or absence of anaemia was negatively correlated with TG in the solitary group, and the maximum myoma diameter was positively correlated with TG in the multiple group.

**Table 2:** Univariate analysis of TGs (μmol/L).

Variable	Total *β* (95% CI) *p*-value	Number of fibroids *β* (95% CI) *p*-value	
		Single	Multiple
Age, years	9.88 (4.71, 15.05) 0.0002	8.99 (1.67, 16.30) 0.0163	10.95 (3.59, 18.30) 0.0036
BMI, kg/m^2^	36.09 (25.23, 46.96) <0.0001	41.37 (24.93, 57.81) <0.0001	31.36 (16.94, 45.78) <0.0001
Maximum diameter of fibroids (cm)	10.11 (−1.31, 21.53) 0.0830	2.23 (−21.55, 26.00) 0.8543	12.90 (0.66, 25.14) 0.0394
*Menstrual pattern n (%)*			
Regularly	Reference	Reference	Reference
Irregularly	−58.01 (−315.99, 199.96) 0.6594	−46.25 (−600.11, 507.61) 0.8700	−61.66 (−345.53, 222.21) 0.6704
*Menstrual flow volume n (%)*			
Light	Reference	Reference	Reference
Moderate	199.21 (−77.29, 475.71) 0.1581	284.34 (−204.41, 773.08) 0.2546	150.75 (−178.84, 480.34) 0.3703
Heavy	148.78 (−144.86, 442.41) 0.3208	142.63 (−379.46, 664.72) 0.5925	149.60 (−199.30, 498.51) 0.4009
*Dysmenorrhea n (%)*			
No	Reference	Reference	Reference
Yes	−62.57 (−159.02, 33.87) 0.2037	−158.13 (−318.30, 2.03) 0.0534	−0.48 (−119.65, 118.68) 0.9937
*Anemia n (%)*			
Yes	Reference	Reference	Reference
No	−51.65 (−125.12, 21.81) 0.1684	−114.23 (−226.54, −1.92) 0.0466	2.67 (−93.93, 99.26) 0.9569
TC, μmol/L	0.21 (0.17, 0.25) <0.0001	0.24 (0.18, 0.30) <0.0001	0.17 (0.12, 0.22) <0.0001
HDL-C, μmol/L	−0.82 (−0.93, −0.71) <0.0001	−0.84 (−1.01, −0.66) <0.0001	−0.81 (−0.95, −0.66) <0.0001
LDL-C, μmol/L	0.21 (0.16, 0.27) <0.0001	0.27 (0.19, 0.34) <0.0001	0.16 (0.09, 0.23) <0.0001
VLDL-C, μmol/L	1.96 (1.85, 2.06) <0.0001	2.08 (1.92, 2.23) <0.0001	1.85 (1.72, 1.99) <0.0001
Platelet Count, 10^9^/L	0.42 (−0.06, 0.90) 0.0894	0.53 (−0.23, 1.28) 0.1711	0.33 (−0.29, 0.95) 0.2976
Lymphocyte Count, 10^9^/L	162.32 (98.54, 226.09) <0.0001	150.88 (52.28, 249.49) 0.0028	171.98 (88.85, 255.10) <0.0001
Neutrophil Count, 10^9^/L	74.22 (52.06, 96.38) <0.0001	69.46 (36.30, 102.62) <0.0001	78.78 (49.03, 108.53) <0.0001
Monocyte Count, 10^9^/L	70.69 (−186.53, 327.91) 0.5902	185.74 (−218.11, 589.59) 0.3677	−20.49 (−351.60, 310.63) 0.9035
PLR	−0.76 (−1.29, −0.23) 0.0048	−0.84 (−1.70, 0.03) 0.0576	−0.71 (−1.37, −0.05) 0.0355
NLR	23.31 (−0.96, 47.59) 0.0600	28.36 (−9.54, 66.25) 0.1429	19.23 (−12.18, 50.63) 0.2305
MLR	−394.70 (−724.64, −64.77) 0.0192	−331.21 (−884.25, 221.83) 0.2409	−435.12 (−840.54, −29.70) 0.0357
SII	0.08 (−0.00, 0.16) 0.0587	0.09 (−0.04, 0.23) 0.1776	0.07 (−0.03, 0.17) 0.1803

UL, Uterine leiomyoma. BMI, body mass index. CI, confidence interval. TGs, triglyceride. TC, total cholesterol. HDL-C, high-density lipoprotein cholesterol. LDL-C, low-density lipoprotein cholesterol. VLDL-C, very low-density lipoprotein cholesterol. NLR, Neutrophil-Lymphocyte Ratio. PLR, Platelet-Lymphocyte Ratio. MLR, Monocyte-Lymphocyte Ratio. SSI, systemic immune-inflammation index.

### 3.3. Results of unadjusted and adjusted linear regression

This study adjusted for confounders such as age, BMI, maximum diameter of fibroids, menstrual pattern, menstrual flow volume, dysmenorrhea and anaemia to examine the separate effects of inflammatory markers (PLR, NLR, MLR and SII) on TG levels (Table 3). After adjusting for all covariates, both NLR (*β* = 0.0282) and SII (*β* = 0.0422) were positively associated with TG. However, MLR (*β* = 0.0176) was negatively associated with TG. Next, the study population was analysed separately by subgroup according to the number of white muscle tumours and the results for the single and multiple recurrence groups were largely consistent with the total study population.

**Table 3:** Relationship between inflammatory markers and TGs (μmol/L) in different models.

	Total *β* (95% CI) *p*-value	Number of fibroids	
		Single *β* (95% CI) *p*-value	Multiple *β* (95% CI) *p*-value
PLR	−0.42 (−1.08, 0.24) 0.2118	−0.90 (−1.90, 0.11) 0.0824	0.03 (−0.83, 0.88) 0.9518
NLR	33.33 (3.60, 63.06) 0.0282	37.13 (−8.68, 82.94) 0.1128	31.39 (−6.94, 69.72) 0.1091
MLR	−484.77 (−884.43, −85.11) 0.0176	−718.72 (−1368.04, −69.39) 0.0305	−292.66 (−786.01, 200.69) 0.2456
SII	0.10 (0.00, 0.20) 0.0422	0.11 (−0.05, 0.27) 0.1737	0.10 (−0.02, 0.23) 0.1158

Adjusted variables: age(smooth), BMI(smooth), maximum diameter of fibroids(smooth), menstrual pattern, menstrual flow volume, dysmenorrhea and anemia.

BMI, body mass index. CI, confidence interval. NLR, Neutrophil-Lymphocyte Ratio. PLR, Platelet-Lymphocyte Ratio. MLR, Monocyte-Lymphocyte Ratio. SSI, systemic immune-inflammation index.

### 3.4. Relationship between inflammatory indicators and TGs

**Figure 2:**
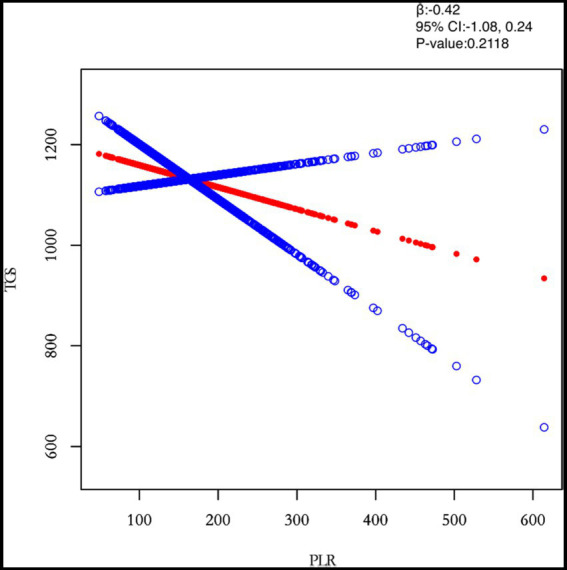
Relation between PLR and TGs (μmol/L). The figure shows the smooth fitting curve of PLR and TGs. The solid red line represents the smooth curve fit between the variables. Blue bands represent the 95% confidence interval of the fit. The model was adjusted for age, BMI, maximum diameter of fibroids, menstrual pattern, menstrual flow volume, dysmenorrhea and anaemia.

**Figure 3:**
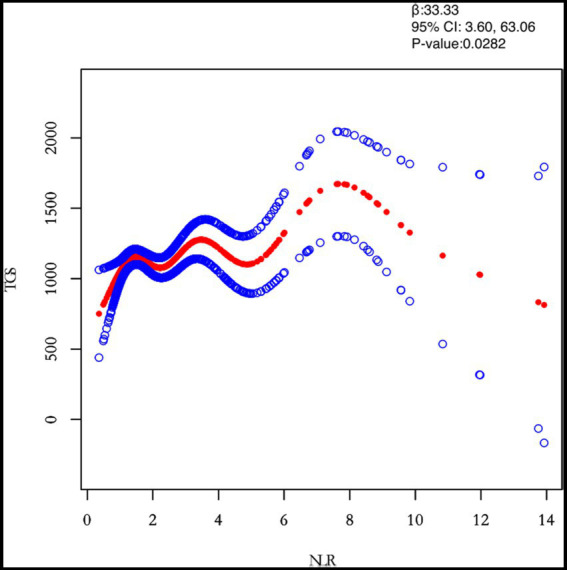
Relation between NLR and TGs (μmol/L). Figure 2 shows the smooth fitting curve of NLR and TGs. The solid red line represents the smooth curve fit between the variables. Blue bands represent the 95% confidence interval of the fit. The model was adjusted for age, BMI, maximum diameter of fibroids, menstrual pattern, menstrual flow volume, dysmenorrhea and anaemia.

**Figure 4:**
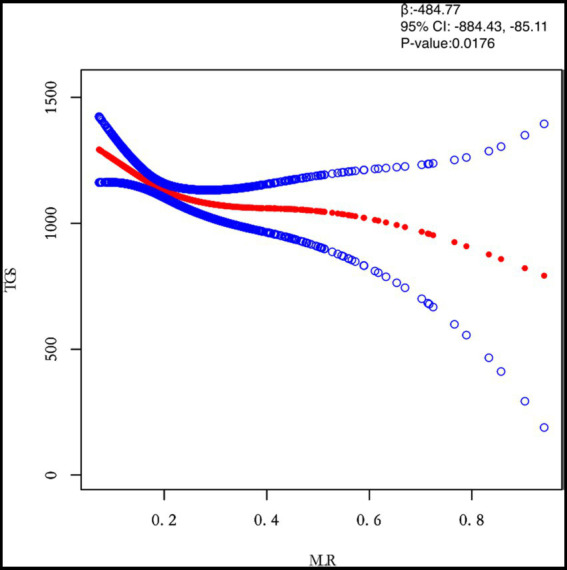
Relation between MLR and TGs (μmol/L). Figure 2 shows the smooth fitting curve of MLR and TGs. The solid red line represents the smooth curve fit between the variables. Blue bands represent the 95% confidence interval of the fit. The model was adjusted for age, BMI, maximum diameter of fibroids, menstrual pattern, menstrual flow volume, dysmenorrhea and anemia.

**Figure 5:**
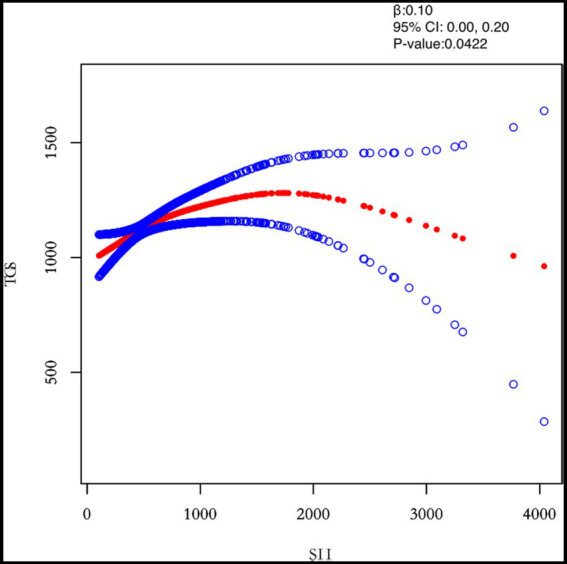
Relation between SII and TGs (μmol/L). Figure 2 shows the smooth fitting curve of SII and TGs. The solid red line represents the smooth curve fit between the variables. Blue bands represent the 95% confidence interval of the fit. The model was adjusted for age, BMI, maximum diameter of fibroids, menstrual pattern, menstrual flow volume, dysmenorrhea and anemia.

In this study, we investigated the relationship between inflammatory indicators and TG (Figures 2–5). According to the results of the smoothed fitted curves and the generalised additive model, TG increased with increasing NLR and SII and TG decreased with increasing PLR and MLR after controlling for age, BMI, menstrual pattern, menstrual volume, dysmenorrhoea and anaemia. Threshold effects were further explored based on curve fitting, as shown in Table 4. There was no significant correlation between PLR and TG. Interestingly, NLR was positively correlated with TG when NLR < 1.25 (*β* = 452.76, 95% *CI* = 52.18, 853.34), whilst there was no significant correlation between NLR and TG when NLR > 1.25. Meanwhile, MLR was negatively correlated with TG when MLR < 0.18 (*β* = −3018.74, 95% *CI* = −5074.77, −962.72), whilst MLR was not significantly correlated with TG when MLR > 0.18. Furthermore, SII was positively correlated with TG when SII < 595.63 (*β* = 0.57, 95% *CI* = 0.23, 0.92), whilst the smoothed fitted curves of SII and TG tended to decrease when PLR > 595.63.

**Table 4:** Threshold effect analysis of the relationship between inflammatory markers and TGs levels.

Adjusted Indicators	PLR *β* (95% CI) *p*-value	NLR *β* (95% CI) *p*-value	MLR *β* (95% CI) *p*-value	SII *β* (95% CI) *p*-value
*Model I*				
One linear effect	−0.44 (−1.09, 0.22) 0.1919	33.05 (3.31, 62.78) 0.0296	−498.54 (−897.29, −99.79) 0.0144	0.10 (0.00, 0.20) 0.0452
*Model II*				
Break point (k)	257.14	1.25	0.18	595.63
<k segment effect 1	−0.11 (−1.04, 0.82) 0.8179	452.76 (52.18, 853.34) 0.0270	−3018.74 (−5074.77, −962.72) 0.0041	0.57 (0.23, 0.92) 0.0013
>k segment effect 2	−1.22 (−2.92, 0.48) 0.1592	22.47 (−8.88, 53.82) 0.1604	−180.90 (−652.94, 291.14) 0.4527	−0.02 (−0.15, 0.11) 0.8039
Effect difference between 2 and 1	−1.11 (−3.34, 1.11) 0.3269	−430.29 (−839.84, −20.75) 0.0397	2837.84 (566.41, 5109.27) 0.0145	−0.59 (−1.01, −0.17) 0.0057
Predicted value of equation at break point	1134.26 (1026.99, 1241.53)	1128.52 (1068.64, 1188.41)	1094.90 (1028.44, 1161.36)	1222.30 (1147.90, 1296.71)
LRT test	0.324	0.039	0.014	0.005

Model I, linear analysis; Model II, nonlinear analysis. LRT test, logarithmic likelihood ratio test. (*p* < 0.05 denotes that Model II significantly differs from Model I, revealing a nonlinear relationship) Adjusted variables: age, BMI, maximum diameter of fibroids, menstrual pattern, menstrual flow volume, dysmenorrhea and anaemia. *p* < 0.05 is significant.

### 3.5. Relationship between inflammatory indicators and TGs in a healthy population

The study included 303 healthy women of childbearing age to explore the correlation between inflammatory markers and TGs in patients without UL. Two models were developed in this study to examine the separate effects of inflammatory markers (PLR, NLR, MLR, and SII) on TG levels in a healthy population (Supplementary Table S1). In the unadjusted model, PLR were all positively correlated with TG. However, PLR (*β* = −2.45) was negatively correlated with TG. Meanwhile, NLR, MLR and SII were not significantly correlated with TG. As shown in Supplementary Figure S1, after adjusting for age, the correlation between PLR and TG tended to decrease according to the smoothed fitted curve fitted scatter plot. However, there was no significant trend in the correlation between NLR, MLR, SII, and TG.

## 4. Discussion

In this study, the relationship between inflammatory markers (PLR, NLR, MLR, SII) and lipid metabolism was analysed comprehensively using univariate analysis, multiple regression analysis and saturation threshold analysis. The results showed that NLR, MLR and SII were significantly associated with TG. Interestingly, the smoothed fit curve of NLR with TG showed a significant upward trend in the first half and a non-significant upward trend in the second half. The smoothed fit curve of MLR with TG showed a significant downward trend in the first half and a non-significant downward trend in the second half. The smoothed fit curve of SIITG showed a significant upward trend in the first half and a downward trend in the second half.

The most frequently occurring female pelvic tumour is UL. Despite its high incidence, our understanding of its pathogenesis, incidence, natural history and risk factors is still far from complete. Some epidemiological and meta-analytic studies (19, 20) have shown that age, high oestrogen status, and obesity are a significant risk signal for the growth of UL after puberty, and that there is a non-linear relationship. Therefore, to investigate the connection between inflammatory response and obesity in patients with UL, the study population selected was women of childbearing age, whilst age was adjusted for in multiple regression analyses. Ciavattini et al. (21) used ultrasound to measure preperitoneal fat thickness in a population to demonstrate that visceral fat expression was associated with the presence of fibroids, whereas subcutaneous fat thickness was not. It was also shown that the role of adipose tissue in promoting inflammation was more pronounced in the visceral than in the subcutaneous area. Two recent clinical case–control studies (22, 23) noted a positive relation between obesity including waist circumference, waist-to-hip ratio, lipid levels and the risk of developing uterine fibroids. Studies (24) have shown that lipid metabolism affects cellular function by regulating the secretion of inflammatory cytokines, and Afrin et al. (25) suggest that adipocytes release inflammatory, pro-fibrotic and angiogenic factors that regulate the metabolic activity of uterine smooth muscle tumour cells by promoting inflammation, fibrosis and angiogenesis. This is similar to the findings of Nair (26), but it is unknown what role inflammation plays in the development of UL. Szydłowska et al. (27) stated that markers of inflammatory and vascular parameters in uterine fibroids treated with selective progesterone receptor modulators corroborated that an important factor in the pathogenesis of UL is the inflammatory response.

PLR, NLR, MLR, and SII are emerging markers of inflammation that are clinically accessible and can be used as biomarkers for a variety of diseases. (28) Guo has suggested (29) that PLR and NLR can reflect the immune cell infiltration status of the tumour microenvironment. The growth and proliferation of tumour stroma and cancerous cells are significantly impacted by the increased secretion of adipokines that is linked to hyperlipidaemia. Adipose tissue produces adipokines, which are also expressed in other organs and tissues. An example of an adipokine is tumour necrosis factor alpha (TNF-alpha). Uterine smooth muscle tumor cells proliferate because of altered cell growth and differentiation that is linked to increased and sustained cytokine production. Also, we concluded from the control group results that NLR, MLR, SII and TG were not significantly correlated in healthy women of childbearing age. Our study found that NLR, MLR and SII were significantly associated with TG in the total study population and in stratified analysis. This suggests that there is an overlap between the inflammatory response and abnormal lipid metabolism in the underlying mechanisms of UL pathogenesis, and also that the pathogenesis of UL is significantly influenced by the inflammatory response. Furthermore, indicators of lipid metabolism and inflammation are important indicators of prevention, diagnosis and treatment as well as prognosis of UL. Therefore, dynamic monitoring of inflammatory markers and TG can effectively assess the progression of UL and guide clinical treatment.

### 4.1. Comparison with previous studies and contribution to existing knowledge

Dyslipidemia is a high risk factor for the development of UL. Our findings showed that inflammatory indicators in UL patients were positively correlated with TG after adjusting for other variables, suggesting that there may be overlapping mechanisms between inflammatory responses and abnormal lipid metabolism in the development and progression of the disease. The large number of people included in this study is replicable and generalisable.

### 4.2. Strengths and limitations

The strength of this study is that it is an observational study in which non-linearity is discussed. And it was also statistically adjusted to rigorously reduce the impact factors. Also, the large amount of data included in this study was effective in reducing population selection bias.

However, there are some limitations to this study. Firstly, our study participants were UL patients diagnosed in southwestern Shandong, China. Therefore, more multi-centre studies are needed to demonstrate this. Secondly, family history is an important factor influencing patients with UL; however, fewer data were collected on patients with UL with a family history. Finally, only patients with a first diagnosis of UL included in this study, and patients with recurrent UL were not included. This study also cannot be generalised to the excluded population.

## 5. Conclusion

To our knowledge, this is the first study to examine the correlation between inflammatory markers and lipids in patients with UL. Moreover, this study analysed single and multiple leiomyosarcomas in subgroups. The results of the study showed a significant correlation between the inflammatory response and lipid metabolism levels in patients with UL. This provides direction for further research into the pathophysiology of UL and also helps to develop hypotheses for predictive models of UL.

## Data availability statement

The original contributions presented in the study are included in the article/Supplementary material, further inquiries can be directed to the corresponding author.

## Ethics statement

The studies involving human participants were reviewed and approved by Medical Science Research Ethics Committee of the Affiliated Hospital of Jining Medical University. Written informed consent for participation was not required for this study in accordance with the national legislation and the institutional requirements.

## Author contributions

CP interpreted the patient data on UL. YD collated the data and was the main contributor to writing the manuscript. LG analysed the patient data on UL. YP conducted the survey and wrote the manuscript. XS performed the validation. YZ and RZ performed the software manipulation. KL and JF performed the visualisation. All authors contributed to the article and approved the submitted version.

## Funding

This work was supported by the TCM Science & Technology Development Plan Project of Shandong Province [grant number 2019–0482].

## Conflict of interest

The authors declare that the research was conducted in the absence of any commercial or financial relationships that could be construed as a potential conflict of interest.

## Publisher’s note

All claims expressed in this article are solely those of the authors and do not necessarily represent those of their affiliated organizations, or those of the publisher, the editors and the reviewers. Any product that may be evaluated in this article, or claim that may be made by its manufacturer, is not guaranteed or endorsed by the publisher.
