# Supplementary material for: Correlation between inflammatory marker and lipid metabolism in patients with uterine leiomyomas
Source: Front Med (Lausanne). 2023 May 3;10:1124697. doi: 10.3389/fmed.2023.1124697 (PMC10189017; doi:10.3389/fmed.2023.1124697)
Supplement: Supplementary file 1 [file Data_Sheet_1.pdf]

XH	AGE	WEIGHT	HEIGH	H1	BMI	JLSL
236	51	73	160	1.6	28.52	1
1819	35	72	168	1.68	25.51	1
779	35	70	170	1.7	24.22	1
778	31	68	164	1.64	25.28	1
1093	31	44	156	1.56	18.08	1
4007	47	67	163	1.63	25.22	1
720	46	47	170	1.7	16.26	1
824	37	65	167	1.67	23.31	1
724	30	59.5	164	1.64	22.12	1
2085	49	62	160	1.6	24.22	2
797	41	57.5	155	1.55	23.93	2
7772	33	53	160	1.6	20.70	2
776	32	95	165	1.65	34.89	1
1507	28	75	160	1.6	29.30	1
3329	30	55	165	1.65	20.20	1
1253	41	57.5	155	1.55	23.93	1
3586	41	60	153	1.53	25.63	1
2214	42	64	166	1.66	23.23	2
1286	35	65	160	1.6	25.39	2
759	37	53	160	1.6	20.70	1
493	52	55	155	1.55	22.89	1
1341	37	80	158	1.58	32.05	1
516	55	80	158	1.58	32.05	1
780	38	71	163	1.63	26.72	1
1685	35	57.5	164	1.64	21.38	1
7621	40	74.5	165	1.65	27.36	1
386	59	62	160	1.6	24.22	1
5003	54	60	160	1.6	23.44	1
738	31	61	162	1.62	23.24	1
1244	33	45	160	1.6	17.58	1
4456	26	50	157	1.57	20.28	1
4280	34	64	162	1.62	24.39	1
728	34	55	160	1.6	21.48	1
6220	57	75	168	1.68	26.57	1
2871	59	72	158	1.58	28.84	1
1278	39	65	163	1.63	24.46	1
3151	54	53	160	1.6	20.70	1
1997	56	59	153	1.53	25.20	1
58	44	78	161	1.61	30.09	1
1448	44	47	150	1.5	20.89	1
6366	52	65	162	1.62	24.77	1
823	53	50	158	1.58	20.03	1
5098	40	66	155	1.55	27.47	1
331	56	72.5	162	1.62	27.63	1
4442	46	70	166	1.66	25.40	1
958	35	67	165	1.65	24.61	1
1512	29	75	168	1.68	26.57	1

3223	53	62	160	1.6	24.22	1
761	40	67	168	1.68	23.74	2
737	46	56	153	1.53	23.92	2
3637	38	53	157	1.57	21.50	2
3985	42	62	162	1.62	23.62	2
268	38	65	164	1.64	24.17	2
7446	42	66	167	1.67	23.67	2
5657	46	86	170	1.7	29.76	2
3225	48	75	170	1.7	25.95	2
797	55	54	158	1.58	21.63	2
839	53	64.5	162	1.62	24.58	2
1628	42	58.5	160	1.6	22.85	2
5297	46	61	152	1.52	26.40	2
4292	52	70	164	1.64	26.03	2
3561	44	62	160	1.6	24.22	2
766	44	53	165	1.65	19.47	1
287	57	70	165	1.65	25.71	1
777	36	74	165	1.65	27.18	1
475	47	73	155	1.55	30.39	1
1651	40	61	165	1.65	22.41	1
4778	39	65	162	1.62	24.77	1
7023	39	70	168.5	1.685	24.65	1
205	46	76	173	1.73	25.39	1
217	36	70	168	1.68	24.80	1
189	52	55	158	1.58	22.03	1
8569	56	55	155	1.55	22.89	1
1602	55	66	165	1.65	24.24	1
1487	49	59	160	1.6	23.05	1
3328	30	75	160	1.6	29.30	1
1348	51	57	158	1.58	22.83	1
1726	49	58	158	1.58	23.23	1
6899	55	52	155	1.55	21.64	1
1555	53	57	157	1.57	23.12	1
3009	43	60	167	1.67	21.51	1
3491	36	54.5	160	1.6	21.29	1
2228	49	92	158	1.58	36.85	1
588	58	70	165	1.65	25.71	1
546	52	70	160	1.6	27.34	1
37	52	67.5	163	1.63	25.41	1
1824	53	62	158	1.58	24.84	1
6429	47	65.5	166	1.66	23.77	1
5376	25	62	165	1.65	22.77	2
1577	43	65	155	1.55	27.06	2
5597	43	83	161	1.61	32.02	2
1020	42	68	165	1.65	24.98	2
903	51	54	153	1.53	23.07	2
4922	50	59	160	1.6	23.05	2
1317	41	79	157	1.57	32.05	2

2570	74	51.5	150	1.5	22.89	2
749 41		57	160	1.6	22.27	1
1556	57	65.5	163	1.63	24.65	1
762 29		85	165	1.65	31.22	1
572 43		62	160	1.6	24.22	2
751 43		54	155	1.55	22.48	1
7648	41	57	161	1.61	21.99	1
1635	42	62	170	1.7	21.45	1
757 53		84	165	1.65	30.85	1
723 40		77	170	1.7	26.64	1
722 29		61	160	1.6	23.83	1
7459	52	72.5	155	1.55	30.18	1
753 54		59	147	1.47	27.30	1
1478	47	62	162	1.62	23.62	1
117	33	65	160	1.6	25.39	1
2380	39	78	169	1.69	27.31	1
5463	45	70	159	1.59	27.69	1
429	41	65	167	1.67	23.31	1
654	37	50.5	155	1.55	21.02	1
980	58	64	150	1.5	28.44	1
756	41	72	170	1.7	24.91	1
7083	42	69	163	1.63	25.97	1
441	36	66	165	1.65	24.24	1
7348	33	58.5	166	1.66	21.23	1
773 34		82	160	1.6	32.03	1
1668	38	62	160	1.6	24.22	1
4586	32	77	167	1.67	27.61	1
161 35		56	158	1.58	22.43	1
1832	44	66	160	1.6	25.78	1
2488	58	56.5	164	1.64	21.01	1
7077	31	54.5	163	1.63	20.51	1
76	51	75	157	1.57	30.43	1
1800	50	65	168	1.68	23.03	1
4716	43	80.5	158	1.58	32.25	1
1012	39	65	161	1.61	25.08	1
3285	47	54	153	1.53	23.07	1
4244	54	60	147	1.47	27.77	1
3763	40	67.5	160	1.6	26.37	1
914	45	62	162	1.62	23.62	1
492	64	62	158	1.58	24.84	1
425	50	75	158	1.58	30.04	1
4356	45	61	165	1.65	22.41	1
1894	48	70	171	1.71	23.94	1
6018	64	71.1	163	1.63	26.76	1
699	39	60	162	1.62	22.86	1
499	46	77	167	1.67	27.61	1
2979	38	57	165	1.65	20.94	1
2726	52	48.5	155	1.55	20.19	1

2853	42	70	163	1.63	26.35	1
740	47	54	160	1.6	21.09	1
685	38	86.5	163	1.63	32.56	2
733 42		65	162	1.62	24.77	2
3451	43	73	158	1.58	29.24	2
1927	42	52.5	157	1.57	21.30	2
633	63	59	156	1.56	24.24	2
1264	42	57	156	1.56	23.42	2
7842	45	64	163	1.63	24.09	2
2297	49	60	165	1.65	22.04	2
7435	45	44.5	155	1.55	18.52	2
2502	44	55	160	1.6	21.48	2
1395	51	66	160	1.6	25.78	2
898	44	69.5	158	1.58	27.84	2
5298	58	60	158	1.58	24.03	2
18	51	74.5	158	1.58	29.84	2
3302	39	49	162	1.62	18.67	2
190	50	48	153	1.53	20.50	2
704 51		80	162	1.62	30.48	2
1256	49	53	155	1.55	22.06	2
758 42		68	165	1.65	24.98	1
1357	48	92	165	1.65	33.79	1
1016	50	54	175	1.75	17.63	1
760	36	73	165	1.65	26.81	1
677	36	67.5	160	1.6	26.37	1
6349	53	60	157	1.57	24.34	1
665	49	55	158	1.58	22.03	1
1970	50	57	163	1.63	21.45	1
5146	53	65	153	1.53	27.77	1
623	54	68	160	1.6	26.56	1
1847	54	70	166	1.66	25.40	1
1655	43	65	160	1.6	25.39	1
3620	55	63	164	1.64	23.42	1
726 40		58	155	1.55	24.14	1
1806	50	57	158	1.58	22.83	1
511	41	78	162	1.62	29.72	1
1102	50	71	160	1.6	27.73	1
2254	46	53	155	1.55	22.06	1
1139	41	67	160	1.6	26.17	1
3918	49	66.5	165	1.65	24.43	1
1388	56	60	160	1.6	23.44	1
4411	53	77	168	1.68	27.28	2
739 44		58	158	1.58	23.23	2
5966	45	61	168	1.68	21.61	2
2984	38	59	172	1.72	19.94	2
931	29	50	160	1.6	19.53	2
4600	49	48	150	1.5	21.33	2
754	38	58	164	1.64	21.56	2

645	45	72	158	1.58	28.84	2
4459	58	61	155	1.55	25.39	2
408	26	63.5	158	1.58	25.44	2
565	38	55	163	1.63	20.70	2
370	45	63	160	1.6	24.61	2
4731	41	52	160	1.6	20.31	1
36	53	57	160	1.6	22.27	1
1142	50	65	158	1.58	26.04	1
570	48	75	155	1.55	31.22	1
4628	51	77.5	163	1.63	29.17	1
2480	52	65	160	1.6	25.39	1
1100	41	41.5	155	1.55	17.27	1
3890	49	59	158	1.58	23.63	1
1269	52	62	159	1.59	24.52	1
3416	52	70	165	1.65	25.71	1
1435	59	60	158	1.58	24.03	1
8091	56	85	170	1.7	29.41	1
4300	45	65	158	1.58	26.04	1
976	28	56	165	1.65	20.57	1
420	52	59	150	1.5	26.22	1
805	31	61.5	162	1.62	23.43	1
1127	50	57	160	1.6	22.27	1
4905	49	50	165	1.65	18.37	1
7623	41	67	156	1.56	27.53	1
3572	46	60	165	1.65	22.04	1
1174	42	60	168	1.68	21.26	1
7876	48	69	158	1.58	27.64	1
780	52	47.5	150	1.5	21.11	1
2772	52	64	158	1.58	25.64	1
333	52	59	153	1.53	25.20	1
1878	47	70	163	1.63	26.35	1
126	47	72	167	1.67	25.82	1
1455	58	68	163	1.63	25.59	1
2761	42	56	162	1.62	21.34	1
6703	48	66	163	1.63	24.84	1
6504	52	70	158	1.58	28.04	1
2870	48	73	167	1.67	26.18	1
1406	36	53	160	1.6	20.70	1
2092	50	71	159	1.59	28.08	1
6556	74	46	152	1.52	19.91	1
4342	51	47	157	1.57	19.07	1
2026	51	49	150	1.5	21.78	1
4360	48	56	158	1.58	22.43	1
4696	45	79	170	1.7	27.34	1
377	36	62	168	1.68	21.97	1
8022	44	65	167	1.67	23.31	1
3400	42	70	161	1.61	27.01	1
302	41	55	160	1.6	21.48	1

4570	42	53	153	1.53	22.64	1
1650	49	51	154	1.54	21.50	1
421	39	60	170	1.7	20.76	2
1904	57	63	158	1.58	25.24	2
1117	35	70	158	1.58	28.04	2
2198	46	66	163	1.63	24.84	2
737	48	65	160	1.6	25.39	2
597	53	69	158	1.58	27.64	2
5233	45	57	160	1.6	22.27	2
1157	49	55	158	1.58	22.03	2
3860	33	66.5	176	1.76	21.47	2
1717	40	87	169	1.69	30.46	2
2326	46	66	160	1.6	25.78	2
1705	27	55	163	1.63	20.70	2
1867	52	58	155	1.55	24.14	2
1843	43	60	155	1.55	24.97	2
1604	52	61	165	1.65	22.41	2
1618	48	60	162	1.62	22.86	2
1687	41	55	158	1.58	22.03	2
7219	54	71	166	1.66	25.77	2
1624	49	69	168	1.68	24.45	2
2870	49	73	158	1.58	29.24	2
3565	42	65	165	1.65	23.88	2
5509	50	62	164	1.64	23.05	2
756	44	71	165	1.65	26.08	2
4767	53	57	158	1.58	22.83	2
6696	50	59	150	1.5	26.22	2
1835	49	60	163	1.63	22.58	2
4206	47	64	156	1.56	26.30	2
747	51	70	160	1.6	27.34	2
746	53	75	172	1.72	25.35	2
11	42	76	169	1.69	26.61	2
2929	43	58.5	164	1.64	21.75	1
180	25	59	170	1.7	20.42	1
1274	50	61	165	1.65	22.41	1
1480	60	69	158	1.58	27.64	1
5295	49	67	164	1.64	24.91	1
327	41	64	160	1.6	25.00	1
1507	34	61	154	1.54	25.72	1
579	49	62.5	160	1.6	24.41	1
233	33	56	165	1.65	20.57	1
106	35	64.5	160	1.6	25.20	1
7725	47	45	155	1.55	18.73	1
7332	48	49	155	1.55	20.40	1
5684	38	44	167	1.67	15.78	2
419	42	65	163	1.63	24.46	2
6760	67	73	163	1.63	27.48	2
171	40	57	163	1.63	21.45	2

8533	29	74	165	1.65	27.18	2
570	43	55	165	1.65	20.20	2
1420	67	58	153	1.53	24.78	2
558 33		58	162	1.62	22.10	2
14 40		64	163	1.63	24.09	2
741 38		73	170	1.7	25.26	2
343 34		60	160	1.6	23.44	1
353 43		62	156	1.56	25.48	1
627 41		60	162	1.62	22.86	2
5050	45	56	165	1.65	20.57	1
756 53		62	150	1.5	27.56	1
3170	51	50	155	1.55	20.81	1
1287	55	65	150	1.5	28.89	1
241 40		52	158	1.58	20.83	1
165 31		57	158	1.58	22.83	1
607	51	60	163	1.63	22.58	1
1086	45	59	170	1.7	20.42	1
755 56		70	160	1.6	27.34	1
8183	48	65	156	1.56	26.71	1
103 43		55	157	1.57	22.31	1
5133	49	51	160	1.6	19.92	1
1926	44	55	158	1.58	22.03	1
98 28		52	162	1.62	19.81	1
475 31		60	158	1.58	24.03	1
234 44		55	155	1.55	22.89	1
7578	54	67.5	162	1.62	25.72	1
63	50	74	168	1.68	26.22	1
22	39	62	162	1.62	23.62	1
184 33		56	164	1.64	20.82	1
170 36		62	159	1.59	24.52	1
306	37	74	165	1.65	27.18	1
5055	45	65	160	1.6	25.39	1
144	36	60	160	1.6	23.44	1
1358	44	47.5	160	1.6	18.55	1
2690	56	65	169	1.69	22.76	1
845	47	66	162	1.62	25.15	1
955	46	59	164	1.64	21.94	1
5392	51	62	158	1.58	24.84	1
1034	40	70	162	1.62	26.67	1
250 34		61	158	1.58	24.44	1
7783	44	63	166	1.66	22.86	1
156 46		67	162	1.62	25.53	1
2427	42	51	158	1.58	20.43	1
3736	46	65	155	1.55	27.06	1
148	31	55	163	1.63	20.70	1
5905	67	55	162	1.62	20.96	1
1558	50	74	165	1.65	27.18	1
1148	50	56	150	1.5	24.89	1

1575	36	49	151	1.51	21.49	1
3268	50	60	158	1.58	24.03	1
7093	47	60	160	1.6	23.44	1
1405	30	69	170	1.7	23.88	1
3551	48	74	158	1.58	29.64	1
701	31	70	160	1.6	27.34	1
769	35	54	159	1.59	21.36	1
3183	48	62	160	1.6	24.22	1
2296	54	55	158	1.58	22.03	1
674	50	62	167	1.67	22.23	1
1327	46	60	160	1.6	23.44	1
932	55	69	154	1.54	29.09	2
145	36	60	160	1.6	23.44	2
7028	50	50.5	155	1.55	21.02	2
312	42	62	160	1.6	24.22	2
2033	51	70	160	1.6	27.34	2
5051	53	76	166	1.66	27.58	2
414	39	74	160	1.6	28.91	2
6733	42	55	162	1.62	20.96	2
713	48	66	161	1.61	25.46	2
38	48	71	155	1.55	29.55	2
5547	58	52	155	1.55	21.64	2
4466	58	78	163	1.63	29.36	2
7537	47	52	161	1.61	20.06	2
655	48	68	158	1.58	27.24	2
3966	37	64	164	1.64	23.80	2
279	50	71	160	1.6	27.73	2
97	41	60	165	1.65	22.04	2
482	46	65	157	1.57	26.37	2
4449	48	69	165	1.65	25.34	2
320	33	70	160	1.6	27.34	2
937	48	76	159	1.59	30.06	2
5923	44	79	163	1.63	29.73	2
6822	34	64	168	1.68	22.68	2
450	39	60	160	1.6	23.44	2
3350	45	64	156	1.56	26.30	2
2050	46	57.5	158	1.58	23.03	2
7479	44	93	160	1.6	36.33	2
1542	40	65	158	1.58	26.04	2
657	48	79	162	1.62	30.10	2
5975	49	65	168	1.68	23.03	2
1494	62	65	163	1.63	24.46	2
4186	49	62.5	162	1.62	23.81	2
7536	51	65	160	1.6	25.39	2
1177	49	59	150	1.5	26.22	2
6400	47	70	167	1.67	25.10	2
4780	46	73	161	1.61	28.16	2
1893	49	72	164	1.64	26.77	2

1166		49	79.5	155	1.55	33.09	2
6140		40	66	160	1.6	25.78	2
107	40		67	160	1.6	26.17	1
764	25		47	156	1.56	19.31	1
111	39		68	163	1.63	25.59	2
474	41		70	160	1.6	27.34	2
70		53	82	163	1.63	30.86	1
261	41		57.5	159	1.59	22.74	1
661		36	65	160	1.6	25.39	1
172	27		62	168	1.68	21.97	2
1352		44	64	164	1.64	23.80	2
2823		35	65	160	1.6	25.39	2
707	43		60	160	1.6	23.44	2
5287		51	63	165	1.65	23.14	2
4155		41	55	153	1.53	23.50	2
1441		39	53.5	155	1.55	22.27	2
659		45	49	158	1.58	19.63	2
4571		42	59	160	1.6	23.05	2
6969		47	70	160	1.6	27.34	2
2183		50	59	158	1.58	23.63	2
706		49	72	172	1.72	24.34	2
118	42		52	145	1.45	24.73	1
346	41		61	158	1.58	24.44	1
355	39		73	168	1.68	25.86	1
280	42		52	157	1.57	21.10	1
646	44		68	160	1.6	26.56	2
327	39		61	165	1.65	22.41	1
564	40		69	158	1.58	27.64	1
555	48		50	156	1.56	20.55	1
296	38		47	165	1.65	17.26	1
389	39		52	156	1.56	21.37	2
473	34		70	166	1.66	25.40	1
379	39		60	160	1.6	23.44	1
300	45		64	161	1.61	24.69	1
306	32		56	160	1.6	21.88	1
66	44		57	155	1.55	23.73	2
639	44		68	165	1.65	24.98	1
52	46		70	159	1.59	27.69	1
554	44		70	167	1.67	25.10	1
43	36		52	163	1.63	19.57	1
630	44		66	160	1.6	25.78	2
397	41		70	158	1.58	28.04	2
443	37		65	160	1.6	25.39	2
606	42		62	154	1.54	26.14	2
318	46		70	165	1.65	25.71	2
1099		38	65	158	1.58	26.04	1
309	28		51	160	1.6	19.92	1
803		49	53	155	1.55	22.06	1

6204	46	52	152	1.52	22.51	1
225 34		52	162	1.62	19.81	1
238 31		54	160	1.6	21.09	1
214 39		56	158	1.58	22.43	1
1247	28	50	163	1.63	18.82	1
1110	53	81	158	1.58	32.45	1
354 40		65	160	1.6	25.39	1
1860	36	58	160	1.6	22.66	1
1285	50	72	169	1.69	25.21	1
876	28	65.5	170	1.7	22.66	1
5067	33	52	162	1.62	19.81	1
1190	52	69	156	1.56	28.35	1
1852	41	60	155	1.55	24.97	1
1481	56	62	160	1.6	24.22	1
1486	38	51	157	1.57	20.69	1
4886	49	55	150	1.5	24.44	1
6459	33	52	158	1.58	20.83	1
1753	46	58	158	1.58	23.23	1
1196	37	49	162	1.62	18.67	1
91 46		62	165	1.65	22.77	1
79 33		77	165	1.65	28.28	1
244 29		55	162	1.62	20.96	1
558	42	70	163	1.63	26.35	1
1849	40	72.5	160	1.6	28.32	1
135 34		64.5	158	1.58	25.84	1
1775	43	53.5	160	1.6	20.90	1
712 56		60	156	1.56	24.65	1
239 35		57	155	1.55	23.73	1
1403	48	52	160	1.6	20.31	1
4821	42	59	156	1.56	24.24	1
1554	40	74	162	1.62	28.20	1
731	44	52.5	161	1.61	20.25	1
4779	34	64	164	1.64	23.80	1
116	50	64	160	1.6	25.00	1
2601	42	64	160	1.6	25.00	1
454 40		72	158	1.58	28.84	1
1342	47	67	160	1.6	26.17	1
1737	44	52	158	1.58	20.83	1
1877	44	65	163	1.63	24.46	1
352 33		80	155	1.55	33.30	1
194 35		68	163	1.63	25.59	1
3049	46	58	170	1.7	20.07	1
187 26		48	160	1.6	18.75	1
1243	36	50	153	1.53	21.36	1
5503	51	61	165	1.65	22.41	1
3782	30	50	165	1.65	18.37	1
96 42		70	165	1.65	25.71	1
1287	51	70	156	1.56	28.76	1

93	38	65	160	1.6	25.39	1
75	37	59	167	1.67	21.16	1
2030	34	70	163	1.63	26.35	1
1407	35	90	165	1.65	33.06	1
641	33	70	165	1.65	25.71	1
905	51	58	155	1.55	24.14	1
561	49	66	165	1.65	24.24	1
1770	35	69	164	1.64	25.65	1
1198	34	50	158	1.58	20.03	1
2238	48	59	160	1.6	23.05	1
5049	42	62	160	1.6	24.22	1
1802	25	85	170	1.7	29.41	1
2683	48	61	158	1.58	24.44	1
2828	47	64	167	1.67	22.95	1
1137	40	50	152	1.52	21.64	1
264	47	65	163	1.63	24.46	1
5201	49	79	163	1.63	29.73	1
65	52	65	168	1.68	23.03	1
13	38	55	167	1.67	19.72	1
1389	43	55	155	1.55	22.89	1
179	41	63.5	158	1.58	25.44	1
2277	41	55	160	1.6	21.48	1
1892	49	66	170	1.7	22.84	1
228	39	64	160	1.6	25.00	1
7528	57	60	158	1.58	24.03	1
962	47	62	155	1.55	25.81	1
6778	46	61	164	1.64	22.68	1
82	45	67	168	1.68	23.74	1
136	49	59	155	1.55	24.56	1
772	45	67.5	167	1.67	24.20	1
62	50	65	165	1.65	23.88	1
263	38	60	160	1.6	23.44	1
169	44	74	152	1.52	32.03	1
1508	42	74	157	1.57	30.02	1
4511	47	70	156	1.56	28.76	1
4777	55	75	155	1.55	31.22	1
6944	49	62	160	1.6	24.22	1
1161	34	65	170	1.7	22.49	1
2550	32	59	160	1.6	23.05	1
6259	39	70	170	1.7	24.22	1
1368	36	55	163	1.63	20.70	1
16	32	75	158	1.58	30.04	1
3911	54	66	163	1.63	24.84	1
1046	46	60	160	1.6	23.44	1
4088	49	72	165	1.65	26.45	1
3182	37	78	161	1.61	30.09	1
2811	58	73.5	160	1.6	28.71	1
7816	44	75	160	1.6	29.30	1

448		35	55	158	1.58	22.03	1
7421		50	60	155	1.55	24.97	1
419		54	66	162	1.62	25.15	1
325		75	73	163	1.63	27.48	1
1096		44	59	161	1.61	22.76	1
1108		51	45	164	1.64	16.73	1
53		49	74	160	1.6	28.91	1
1961		41	74	156	1.56	30.41	1
1004		55	65	157	1.57	26.37	1
4484		34	55	163	1.63	20.70	1
1832		43	76	162	1.62	28.96	1
1417		58	50	150	1.5	22.22	1
4266		43	82	165	1.65	30.12	1
42		30	64.5	168	1.68	22.85	1
3501		21	96	168	1.68	34.01	1
84		43	58	162	1.62	22.10	1
223	25		66	164	1.64	24.54	2
202	37		52	160	1.6	20.31	2
509	42		79	160	1.6	30.86	2
673	43		61	162	1.62	23.24	2
832		37	80	168	1.68	28.34	2
441	33		61	160	1.6	23.83	2
541	27		65	160	1.6	25.39	2
33	43		73	170	1.7	25.26	2
1180		27	53	168	1.68	18.78	2
5720		43	64	160	1.6	25.00	2
247		48	74	168	1.68	26.22	2
633	40		55	156	1.56	22.60	2
518	50		51.5	157	1.57	20.89	2
951		52	80	161	1.61	30.86	2
1270		47	62	162	1.62	23.62	2
2894		50	55	160	1.6	21.48	2
1122		47	63	159	1.59	24.92	2
867		43	72.5	167	1.67	26.00	2
7486		47	53.5	161	1.61	20.64	2
967		53	57	151	1.51	25.00	2
181	45		61.5	162	1.62	23.43	2
512	41		59	170	1.7	20.42	2
299	34		62	158	1.58	24.84	2
1267		54	70	159	1.59	27.69	2
516	43		75	158	1.58	30.04	2
303	42		68	160	1.6	26.56	2
134	40		62	158	1.58	24.84	2
1481		43	52	158	1.58	20.83	2
1052		54	72	157	1.57	29.21	2
683		35	87.5	167	1.67	31.37	2
899		56	64	160	1.6	25.00	2
651	41		60	160	1.6	23.44	2

619	44	72	165	1.65	26.45	2
178	46	61	161	1.61	23.53	2
825	43	65	155	1.55	27.06	2
8403	39	51	158	1.58	20.43	2
7972	39	65	166	1.66	23.59	2
203	51	69.5	153	1.53	29.69	2
1933	34	47.5	160	1.6	18.55	2
5288	42	58.5	161	1.61	22.57	2
2084	30	53	150	1.5	23.56	2
256	42	65	160	1.6	25.39	2
853	42	56	160	1.6	21.88	2
661	45	68	160	1.6	26.56	2
520	43	65	162	1.62	24.77	2
3960	46	56	158	1.58	22.43	2
215	36	75	164	1.64	27.89	2
521	34	65	168	1.68	23.03	2
7377	36	62.5	168	1.68	22.14	2
999	52	60	160	1.6	23.44	2
3525	45	65	168	1.68	23.03	2
1634	47	58.5	161	1.61	22.57	2
1128	48	67	164	1.64	24.91	2
293	45	72	162	1.62	27.43	2
2044	41	78	165	1.65	28.65	2
649	46	70	162	1.62	26.67	2
959	50	52	158	1.58	20.83	2
4918	39	53.5	160	1.6	20.90	2
8056	44	69	168	1.68	24.45	2
958	51	66	165	1.65	24.24	2
7728	42	68	160	1.6	26.56	2
441	45	58	160	1.6	22.66	2
2266	45	71	159	1.59	28.08	2
675	43	69	160	1.6	26.95	2
217	37	68	165	1.65	24.98	2
671	38	65	167	1.67	23.31	2
1526	43	68.5	160	1.6	26.76	2
4382	48	61	158	1.58	24.44	2
428	44	45	155	1.55	18.73	2
5586	46	60	153	1.53	25.63	2
4739	34	53	160	1.6	20.70	2
1292	47	78	163	1.63	29.36	2
84	49	58	155	1.55	24.14	2
6959	42	86	163	1.63	32.37	2
4147	40	70	162	1.62	26.67	2
210	29	58.5	155	1.55	24.35	2
1553	58	68	163	1.63	25.59	2
1784	45	60	158	1.58	24.03	2
485	39	75	163	1.63	28.23	2
4440	33	40	150	1.5	17.78	2

1600	52	63.5	163	1.63	23.90	2
924	47	70	166	1.66	25.40	2
5591	48	64	163	1.63	24.09	2
960	39	64	160	1.6	25.00	2
787	52	66	158	1.58	26.44	2
4238	40	74	160	1.6	28.91	2
968	44	62.5	160	1.6	24.41	2
333	45	50	155	1.55	20.81	2
229	47	67	163	1.63	25.22	2
5241	47	65	160	1.6	25.39	2
634	41	60	160	1.6	23.44	2
1871	31	55	162	1.62	20.96	2
200	39	60	152	1.52	25.97	2
384	53	56	158	1.58	22.43	2
1415	49	75	159	1.59	29.67	2
4722	51	61	162	1.62	23.24	2
8	36	60	160	1.6	23.44	2
4776	51	57	160	1.6	22.27	2
3329	50	69	168	1.68	24.45	2
808	51	69.5	164	1.64	25.84	2
6070	49	65	158	1.58	26.04	2
148	48	56	160	1.6	21.88	2
3706	35	85	162	1.62	32.39	2
856	52	56	158	1.58	22.43	2
3253	52	86.5	164	1.64	32.16	2
2351	51	74	160	1.6	28.91	2
4868	43	67	161	1.61	25.85	2
733	46	67	161	1.61	25.85	2
7913	45	65	160	1.6	25.39	2
3325	58	64	158	1.58	25.64	2
4521	34	65	165	1.65	23.88	2
4785	50	70	159	1.59	27.69	2
285	30	85	168	1.68	30.12	2
1087	50	57	160	1.6	22.27	2
778	51	67	160	1.6	26.17	2
6532	40	65	165	1.65	23.88	2
4507	50	74	164	1.64	27.51	2
2698	47	68	154	1.54	28.67	2
2301	53	52	160	1.6	20.31	2
1070	41	60	158	1.58	24.03	2
2809	41	75	162	1.62	28.58	2
3031	51	60	162	1.62	22.86	2
1187	50	69	160	1.6	26.95	2
8598	50	79	170	1.7	27.34	2
642	43	60	156	1.56	24.65	1
381	37	68	160	1.6	26.56	1
307	48	60	164	1.64	22.31	1
559	40	90	170	1.7	31.14	2

342	33		67	165	1.65	24.61	1
281	33		60	163	1.63	22.58	1
329	33		59	162	1.62	22.48	1
292	38		65	162	1.62	24.77	1
593	40		64	156	1.56	26.30	2
403	43		55	155	1.55	22.89	2
42	39		63.5	154	1.54	26.78	2
468	37		71	168	1.68	25.16	2
382	39		60	165	1.65	22.04	1
321	31		55	162	1.62	20.96	1
163	41		63	156	1.56	25.89	1
129	37		57	155	1.55	23.73	1
279	42		63	160	1.6	24.61	1
574	35		62	160	1.6	24.22	1
465	44		76	163	1.63	28.60	2
404	42		58	160	1.6	22.66	2
269	40		63	158	1.58	25.24	1
372	43		95.5	165	1.65	35.08	1
317	43		51	155	1.55	21.23	1
491	43		59	163	1.63	22.21	2
390	45		55	160	1.6	21.48	2
613	46		57	158	1.58	22.83	2
348	45		74	165	1.65	27.18	1
590	37		63	166	1.66	22.86	1
243	39		54	158	1.58	21.63	1
2899		37	48	163	1.63	18.07	1
930		40	43	150	1.5	19.11	1
314	41		62	160	1.6	24.22	1
7517		48	66	164	1.64	24.54	1
3370		40	71	160	1.6	27.73	1
4238		31	55	165	1.65	20.20	1
1404		35	65	155	1.55	27.06	1
25	42		61	163	1.63	22.96	2
573	37		57	162	1.62	21.72	2
600	40		58	160	1.6	22.66	2
6948		46	61.5	154	1.54	25.93	2
344	46		68	162	1.62	25.91	1
322	45		70	160	1.6	27.34	1
64	38		70	165	1.65	25.71	1
496	42		55	157	1.57	22.31	1
61	35		84	169	1.69	29.41	1
638	34		55	160	1.6	21.48	1
259	44		61	158	1.58	24.44	1
373	43		64	167	1.67	22.95	2
601	43		55	158	1.58	22.03	2
451	39		58	170	1.7	20.07	2
51	41		67	158	1.58	26.84	1
341	41		76	165	1.65	27.92	1

568	38		60	162	1.62	22.86	2
634	47		60	160	1.6	23.44	2
328	40		82	172	1.72	27.72	1
425	36		70	163	1.63	26.35	1
437	43		56	162	1.62	21.34	1
331	42		64	160	1.6	25.00	1
385	34		60.5	158	1.58	24.23	2
338	41		65	160	1.6	25.39	1
456	35		69	158	1.58	27.64	1
275	48		52.5	159	1.59	20.77	1
648	49		55	150	1.5	24.44	2
435	37		68	164	1.64	25.28	2
2629		45	59	165	1.65	21.67	1
240	40		57	162	1.62	21.72	1
667		34	65	160	1.6	25.39	1
980		34	55	160	1.6	21.48	1
1746		46	65	168	1.68	23.03	1
45	39		65	158	1.58	26.04	1
249	37		55	153	1.53	23.50	1
3951		50	58	168	1.68	20.55	1
83	28		59.5	160	1.6	23.24	1
65	46		75	172	1.72	25.35	1
34	19		58	173	1.73	19.38	1
286	43		65	160	1.6	25.39	1
3648		47	65	160	1.6	25.39	1
213	45		60	165	1.65	22.04	1
7761		71	51	154	1.54	21.50	1
3706		34	53	161	1.61	20.45	1
4144		49	64	165	1.65	23.51	1
5652		33	49	160	1.6	19.14	1
681		45	59	165	1.65	21.67	1
298		49	61	159	1.59	24.13	1
29		46	64	158	1.58	25.64	1
2121		47	53	155	1.55	22.06	1
198	41		56	162	1.62	21.34	1
84	26		53	160	1.6	20.70	1
662	42		55	160	1.6	21.48	1
143		42	66	158	1.58	26.44	1
193	48		66	158	1.58	26.44	1
173	38		70	166	1.66	25.40	1
203	39		70	167	1.67	25.10	1
1695		38	57	158	1.58	22.83	1
766		38	51	160	1.6	19.92	1
1823		38	87	168	1.68	30.82	1
4190		50	68	168	1.68	24.09	1
839		52	85	165	1.65	31.22	1
121	35		54	165	1.65	19.83	1
182	35		83	168	1.68	29.41	1

516	33	55	155	1.55	22.89	1
236	44	44	155	1.55	18.31	1
133	34	76	160	1.6	29.69	1
1301	38	54	160	1.6	21.09	1
5572	33	52	163	1.63	19.57	1
3299	39	55	156	1.56	22.60	1
4257	35	54	165	1.65	19.83	1
2975	40	54	162	1.62	20.58	1
122	35	61	160	1.6	23.83	1
100	39	72	165	1.65	26.45	1
2574	42	50	163	1.63	18.82	1
4717	32	58	161	1.61	22.38	1
47	44	57	160	1.6	22.27	1
971	29	69	161	1.61	26.62	1
546	53	81	159	1.59	32.04	1
5646	38	75	160	1.6	29.30	1
2033	36	63	170	1.7	21.80	1
1596	54	55	160	1.6	21.48	1
19	48	58	152	1.52	25.10	1
1500	35	53	162	1.62	20.20	1
6133	43	56	155	1.55	23.31	1
3112	55	63	167	1.67	22.59	1
4712	47	54	160	1.6	21.09	1
183	39	71	170	1.7	24.57	1
4413	53	57	156	1.56	23.42	1
75	54	62.5	157	1.57	25.36	1
1948	34	59	159	1.59	23.34	1
1171	43	52	160	1.6	20.31	1
6122	39	62.5	165	1.65	22.96	1
179	30	69	162	1.62	26.29	1
1935	50	67	166	1.66	24.31	1
1709	36	65	168	1.68	23.03	1
829	50	56	165	1.65	20.57	1
440	50	76	164	1.64	28.26	1
2028	36	61	160	1.6	23.83	1
2251	51	76	170	1.7	26.30	1
232	34	52	156	1.56	21.37	1
503	31	61	168	1.68	21.61	1
8318	68	70	162	1.62	26.67	1
131	47	62	161	1.61	23.92	1
1230	57	70	164	1.64	26.03	1
1288	49	90	165	1.65	33.06	1
1641	52	71.5	159	1.59	28.28	1
1938	40	62	156	1.56	25.48	1
48	38	76	173	1.73	25.39	1
233	44	71	168	1.68	25.16	1
3030	46	55	158	1.58	22.03	1
190	44	70	165	1.65	25.71	1

3593	38	54	166	1.66	19.60	1
748	38	60	160	1.6	23.44	1
294	35	50	150	1.5	22.22	1
8287	33	82	163	1.63	30.86	1
2440	43	59	155	1.55	24.56	1
1545	32	63	165	1.65	23.14	1
1838	45	64.5	160	1.6	25.20	1
1153	47	67	161	1.61	25.85	1
1439	47	64	150	1.5	28.44	1
601	43	61.5	167	1.67	22.05	1
3543	46	59	163	1.63	22.21	1
1043	43	78	167	1.67	27.97	1
1905	56	63	170	1.7	21.80	1
1933	38	69.5	163	1.63	26.16	1
1453	52	69	160	1.6	26.95	1
2157	35	63	160	1.6	24.61	1
1836	32	73	165	1.65	26.81	1
1347	41	57	157	1.57	23.12	1
701	41	57	166	1.66	20.69	1
3784	27	55	160	1.6	21.48	1
2300	54	72	165	1.65	26.45	1
6087	52	78	165	1.65	28.65	1
216	45	54	168	1.68	19.13	1
1932	40	61	165	1.65	22.41	1
2152	42	65	169.5	1.695	22.62	1
6418	52	58	160	1.6	22.66	1
4037	40	64.5	150	1.5	28.67	1
172	48	60	160	1.6	23.44	1
856	40	56.5	158	1.58	22.63	1
581	41	64	163	1.63	24.09	1
889	51	68	155	1.55	28.30	1
1291	31	61.5	167	1.67	22.05	1
6353	33	64	163	1.63	24.09	1
1233	38	84	164	1.64	31.23	1
267	44	51	157	1.57	20.69	2
222	51	70	163	1.63	26.35	2
522	42	68	166	1.66	24.68	2
16	43	62	159	1.59	24.52	2
7511	45	67	158	1.58	26.84	2
1111	46	60	150	1.5	26.67	2
116	39	61	170	1.7	21.11	2
652	50	60	160	1.6	23.44	2
3932	45	59.5	157	1.57	24.14	2
15	44	55	160	1.6	21.48	2
308	53	72.5	160	1.6	28.32	2
1930	44	70	164	1.64	26.03	2
212	42	54	155	1.55	22.48	2
365	37	70	158	1.58	28.04	2

6916	37	59	160	1.6	23.05	2
1665	33	65	165	1.65	23.88	2
415	50	63	158	1.58	25.24	2
670 49		66	156	1.56	27.12	2
2715	52	52	158	1.58	20.83	2
977	46	80	167	1.67	28.69	2
529	44	48	150	1.5	21.33	2
6721	44	52	165	1.65	19.10	2
200 37		70	160	1.6	27.34	2
417	40	62	162	1.62	23.62	2
3897	53	72	160	1.6	28.13	2
7867	40	50	160	1.6	19.53	2
700	53	69	153	1.53	29.48	2
7941	48	58	158	1.58	23.23	2
1662	49	74	166	1.66	26.85	2
195 38		59	164	1.64	21.94	2
8045	54	55	150	1.5	24.44	2
1414	47	61	160	1.6	23.83	2
1520	44	70	160	1.6	27.34	2
495 31		48	162	1.62	18.29	2
3637	53	67	165	1.65	24.61	2
4882	50	57	160	1.6	22.27	2
184	45	65.5	163	1.63	24.65	2
4513	47	75	162	1.62	28.58	2
168	41	70	158	1.58	28.04	2
3631	55	55.5	156	1.56	22.81	2
2771	52	75	164	1.64	27.89	2
326 29		67	160	1.6	26.17	2
521 37		65	160	1.6	25.39	2
1356	42	50	155	1.55	20.81	2
2155	32	53	163	1.63	19.95	2
3788	42	62	162	1.62	23.62	2
6439	46	60	169	1.69	21.01	2
649	51	67.5	155	1.55	28.10	2
5628	45	67	159	1.59	26.50	2
4148	43	62.5	166	1.66	22.68	2
707	48	69	152	1.52	29.86	2
668 46		60	160	1.6	23.44	2
7996	40	70	158	1.58	28.04	2
130 29		55	160	1.6	21.48	2
2267	49	62	167	1.67	22.23	2
3914	53	65	156	1.56	26.71	2
464 36		50	158	1.58	20.03	2
660	41	60	160	1.6	23.44	2
1379	34	80	170	1.7	27.68	2
1035	46	61	168	1.68	21.61	2
711 42		62	170	1.7	21.45	2
4809	45	67	160	1.6	26.17	2

1524	45	90	172	1.72	30.42	2
8408	42	69	165	1.65	25.34	2
1391	51	65	162	1.62	24.77	2
936	52	60	162	1.62	22.86	2
5289	48	52	150	1.5	23.11	2
2525	42	62	164	1.64	23.05	2
2481	31	78	165	1.65	28.65	2
1547	50	60	162	1.62	22.86	2
2241	52	50.5	156	1.56	20.75	2
294	63	53.5	162	1.62	20.39	2
3362	52	65	155	1.55	27.06	2
3570	41	62	160	1.6	24.22	2
447	55	62	165	1.65	22.77	2
5612	46	80	165	1.65	29.38	2
6652	52	81	165	1.65	29.75	2
1201	50	50	160	1.6	19.53	2
7661	45	60	162	1.62	22.86	2
338	51	61	160	1.6	23.83	2
5046	50	58	158	1.58	23.23	2
5222	28	75	163	1.63	28.23	2
532	48	55.5	156	1.56	22.81	2
1664	53	74	160	1.6	28.91	2
4196	48	72	168	1.68	25.51	2
7249	30	52	156	1.56	21.37	2
1373	56	61	165	1.65	22.41	2
3030	58	66.5	168	1.68	23.56	2
8242	50	63	160	1.6	24.61	2
1663	46	58	161	1.61	22.38	2
1206	49	74	158	1.58	29.64	2
988	54	56	158	1.58	22.43	2
8057	43	75	160	1.6	29.30	2
7398	54	69	160	1.6	26.95	2
3367	51	61	160	1.6	23.83	2
2609	36	78	165	1.65	28.65	2
2782	51	60	158	1.58	24.03	2
6107	60	60	168	1.68	21.26	2
3678	44	58	164	1.64	21.56	2
2905	36	59	160	1.6	23.05	2
5150	51	53	156	1.56	21.78	2
3293	40	60	176	1.76	19.37	2
29	42	67	159	1.59	26.50	2
591	52	72	156	1.56	29.59	2
435	40	49	150	1.5	21.78	2
4769	50	55	155	1.55	22.89	2
7567	34	82	158	1.58	32.85	2
5466	44	57.5	156	1.56	23.63	2
1561	34	42	163	1.63	15.81	2
1543	47	66	153	1.53	28.19	2

4638	28	56	165	1.65	20.57	2
1538	52	69	165	1.65	25.34	2
557	41	65	160	1.6	25.39	1
452	38	75	170	1.7	25.95	1
335	28	72.5	163	1.63	27.29	1
604	40	66	160	1.6	25.78	2
124	41	53	155	1.55	22.06	1
414	49	52	152	1.52	22.51	2
106	39	60	158	1.58	24.03	2
265	39	50	153	1.53	21.36	1
336	46	66	160	1.6	25.78	1
272	41	60	166	1.66	21.77	1
105	45	55	160	1.6	21.48	2
362	35	60	165	1.65	22.04	2
626	41	61	160	1.6	23.83	1
47	46	56	162	1.62	21.34	1
349	41	55	156	1.56	22.60	1
54	23	54	160	1.6	21.09	1
242	34	55	165	1.65	20.20	1
159	35	67	155	1.55	27.89	1
1106	44	67	165	1.65	24.61	1
3946	51	67	160	1.6	26.17	2
719	44	64	163	1.63	24.09	2
2850	52	69	160	1.6	26.95	2
4433	60	60	158	1.58	24.03	2
380	47	73	158	1.58	29.24	2
8316	56	70	165	1.65	25.71	2
3296	26	61	170	1.7	21.11	2
400	45	60	163	1.63	22.58	1
1129	51	84	155	1.55	34.96	1
399	43	61.5	160	1.6	24.02	2
157	37	48.5	161	1.61	18.71	1
398	48	63	165	1.65	23.14	2
645	45	55	155	1.55	22.89	2
378	42	60	151	1.51	26.31	1
162	39	70	167	1.67	25.10	1
268	44	70	169	1.69	24.51	1
570	45	48	158	1.58	19.23	1
313	36	68	166	1.66	24.68	1
324	46	60	165	1.65	22.04	2
5	34	64	165	1.65	23.51	1
1826	42	68	159	1.59	26.90	1
189	36	74	162	1.62	28.20	1
351	45	63	162	1.62	24.01	1
3586	36	69	163	1.63	25.97	1
882	22	45	163	1.63	16.94	1
6770	42	53	160	1.6	20.70	1
8001	44	45	158	1.58	18.03	1

139	45		60	157	1.57	24.34	1
2526		50	60	160	1.6	23.44	1
4397		40	47.5	160	1.6	18.55	1
477	49		73	163	1.63	27.48	1
3947		45	67	159	1.59	26.50	1
8144		46	75	167	1.67	26.89	1
1723		44	62	158	1.58	24.84	1
210		40	55	163	1.63	20.70	1
992		43	57.5	155	1.55	23.93	1
4351		47	75	167	1.67	26.89	1
4992		45	62	163	1.63	23.34	1
186	40		58	162	1.62	22.10	1
176	38		50	155	1.55	20.81	1
73	39		75	165	1.65	27.55	1
526		37	65	168	1.68	23.03	1
1065		39	65	158	1.58	26.04	1
60		31	67.5	165	1.65	24.79	1
152	44		71	163	1.63	26.72	1
247	48		60	160	1.6	23.44	1
631		42	67	165	1.65	24.61	1
3888		52	60	160	1.6	23.44	1
2065		40	63	160	1.6	24.61	1
3347		32	60	165	1.65	22.04	1
2428		48	58	165	1.65	21.30	1
6943		48	65	160	1.6	25.39	1
494		46	51.5	157	1.57	20.89	1
209	42		63	164	1.64	23.42	1
2244		46	55	164	1.64	20.45	1
841		41	79.5	169	1.69	27.84	1
6773		42	70	163	1.63	26.35	1
550		52	54	165	1.65	19.83	1
5736		52	76	158	1.58	30.44	1
4074		26	72	165	1.65	26.45	1
783		45	59	158	1.58	23.63	1
210		42	86	160	1.6	33.59	1
218		43	69	160	1.6	26.95	1
1841		47	55	165	1.65	20.20	1
11		36	76	160	1.6	29.69	1
5130		35	54	160	1.6	21.09	1
5469		40	78	163	1.63	29.36	1
1274		38	59	155	1.55	24.56	1
1103		57	50	152	1.52	21.64	1
3168		30	62	177	1.77	19.79	1
447		37	62	160	1.6	24.22	1
1199		47	58	160	1.6	22.66	1
876		26	54	165	1.65	19.83	1
4217		33	76	163	1.63	28.60	1
3334		50	50	155	1.55	20.81	1

295	50	52	155	1.55	21.64	1
1374	49	57	165	1.65	20.94	1
4114	37	63	165	1.65	23.14	1
1254	48	50	145	1.45	23.78	1
227	38	56	158	1.58	22.43	1
5035	48	50	158	1.58	20.03	1
7423	35	49	155	1.55	20.40	1
174	33	61	159	1.59	24.13	2
519	37	65	160	1.6	25.39	2
2773	43	65	167	1.67	23.31	2
4664	55	66	165	1.65	24.24	2
207	43	50	153	1.53	21.36	2
6800	47	54	160	1.6	21.09	2
5596	52	74	151	1.51	32.45	2
1383	37	57.5	166	1.66	20.87	2
4998	47	72	174	1.74	23.78	2
4635	46	49	163	1.63	18.44	2
1873	49	61.5	160	1.6	24.02	2
2251	50	62	170	1.7	21.45	2
23	49	54	160	1.6	21.09	2
7742	52	50	165	1.65	18.37	2
502	43	70	163	1.63	26.35	2
635	41	71	157	1.57	28.80	2
6860	33	60	163	1.63	22.58	2
1095	48	53	157	1.57	21.50	2
2660	52	56	166	1.66	20.32	2
415	47	58	155	1.55	24.14	2
3081	55	56	151	1.51	24.56	2
168	47	67	166	1.66	24.31	2
2894	38	54	158	1.58	21.63	2
483	37	59	160	1.6	23.05	2
7118	49	57	162	1.62	21.72	2
1730	51	70	166	1.66	25.40	2
796	38	67	166	1.66	24.31	2
637	27	60	170	1.7	20.76	2
1911	47	84	166	1.66	30.48	2
550	40	58	155	1.55	24.14	2
707	43	59	158	1.58	23.63	2
126	41	80	158	1.58	32.05	2
5635	42	75	155	1.55	31.22	2
7768	43	73	168	1.68	25.86	2
2934	45	55	156	1.56	22.60	2
2	38	64	166	1.66	23.23	2
4920	53	51	158	1.58	20.43	2
6871	53	65	160	1.6	25.39	2
1447	50	70	155	1.55	29.14	2
2283	52	64	150	1.5	28.44	2
2435	55	82	162	1.62	31.25	2

3782	32	65	164	1.64	24.17	2
3862	35	60	160	1.6	23.44	2
6089	53	61	164	1.64	22.68	2
6575	24	54	165	1.65	19.83	2
1009	48	66.5	160	1.6	25.98	2
1014	51	71	156	1.56	29.17	2
310	51	74	157.5	1.575	29.83	2
1942	42	80	166	1.66	29.03	2
835	45	61	165	1.65	22.41	2
553	40	67	168	1.68	23.74	2
5203	45	60	168	1.68	21.26	2
4974	41	65	165	1.65	23.88	2
6422	42	73	160	1.6	28.52	2
440	38	63	159	1.59	24.92	2
2407	39	59	155	1.55	24.56	2
4465	45	77.5	170	1.7	26.82	2
4432	50	71	161	1.61	27.39	2
6698	51	71	163	1.63	26.72	2
1739	44	68	165	1.65	24.98	2
2002	41	70	160	1.6	27.34	2
2231	58	80	170	1.7	27.68	2
2404	64	60	158	1.58	24.03	2
1708	50	74	150	1.5	32.89	2
7960	48	70	158	1.58	28.04	2
359	39	63	160	1.6	24.61	1
610	39	67	163	1.63	25.22	2
479	41	80	168	1.68	28.34	2
436	47	65	160	1.6	25.39	1
291	31	52.5	160	1.6	20.51	1
315	42	70	172	1.72	23.66	1
442	39	62	160	1.6	24.22	1
556	42	51	158	1.58	20.43	2
274	44	48	155	1.55	19.98	1
5417	44	60	158	1.58	24.03	1
1168	39	75	164	1.64	27.89	1
6178	53	84	160	1.6	32.81	1
4109	42	69	160	1.6	26.95	2
631	40	57	157	1.57	23.12	1
602	43	59	152	1.52	25.54	2
347	43	60	165	1.65	22.04	1
298	36	67	158	1.58	26.84	1
462	44	57	160	1.6	22.27	1
587	37	66	158	1.58	26.44	2
58	41	58	158	1.58	23.23	2
679	36	60	162	1.62	22.86	1
4349	47	58	160	1.6	22.66	1
822	56	69	153	1.53	29.48	1
340	37	72	169	1.69	25.21	1

3286	52	79	160	1.6	30.86	1
1687	47	62.5	159	1.59	24.72	1
8044	56	59	158	1.58	23.63	1
141	39	54.5	157	1.57	22.11	1
4651	43	80	167	1.67	28.69	1
246	41	60	158	1.58	24.03	1
201	44	59	169	1.69	20.66	1
427	39	80	165	1.65	29.38	1
164	28	87	166	1.66	31.57	1
308	47	69	155	1.55	28.72	1
1794	32	59	162	1.62	22.48	1
379	47	69	156	1.56	28.35	1
1046	49	73.5	157	1.57	29.82	1
531	32	57.5	160	1.6	22.46	1
1953	35	50	160	1.6	19.53	1
1095	38	49	160	1.6	19.14	1
618	48	70	173	1.73	23.39	1
1739	50	63	160	1.6	24.61	1
302	44	51	155	1.55	21.23	1
848	45	55	153	1.53	23.50	1
3028	57	65	155	1.55	27.06	1
5540	34	48	158	1.58	19.23	1
48	37	58	161	1.61	22.38	1
1666	52	50	155	1.55	20.81	1
849	34	52	157	1.57	21.10	1
7491	42	59.5	160	1.6	23.24	1
5358	44	66	165	1.65	24.24	1
1100	47	51	158	1.58	20.43	1
1366	47	51	160	1.6	19.92	1
4371	43	68	164	1.64	25.28	1
3417	48	80	169	1.69	28.01	1
3559	43	51	162	1.62	19.43	1
5497	40	55	150	1.5	24.44	1
351	45	60	166	1.66	21.77	1
1356	25	57	170	1.7	19.72	1
2582	36	67.5	172.5	1.725	22.68	1
7689	40	57	160	1.6	22.27	2
6165	48	63	160	1.6	24.61	2
560	37	70	162	1.62	26.67	2
6670	59	75.5	162	1.62	28.77	2
3390	31	75	168	1.68	26.57	2
81	47	52	160	1.6	20.31	2
5639	47	62	160	1.6	24.22	2
308	44	70	160	1.6	27.34	2
1649	52	65	160	1.6	25.39	2
254	39	51	155	1.55	21.23	2
708	48	64	158	1.58	25.64	2
175	44	60	157	1.57	24.34	2

706	43	58	158	1.58	23.23	2
8394	44	65	164	1.64	24.17	2
3447	42	58	163	1.63	21.83	2
1849	47	58	155	1.55	24.14	2
8058	48	59	160	1.6	23.05	2
1586	52	70	166	1.66	25.40	2
577	46	53	155	1.55	22.06	2
1436	28	59	167	1.67	21.16	2
680	41	64	160	1.6	25.00	2
6244	44	87	160	1.6	33.98	2
603	48	50	158	1.58	20.03	2
713	52	68	155	1.55	28.30	2
1435	56	57.5	155	1.55	23.93	2
1068	40	74	170	1.7	25.61	2
2237	40	76	168	1.68	26.93	2
3865	32	60	170	1.7	20.76	2
4725	43	56.5	158	1.58	22.63	2
5539	31	49	157	1.57	19.88	2
60	43	57.5	160	1.6	22.46	2
2232	52	66	162	1.62	25.15	2
235	45	61	155	1.55	25.39	2
2830	40	64	163	1.63	24.09	2
1011	31	59	158	1.58	23.63	2
1063	54	70	159	1.59	27.69	2
101	48	57	147	1.47	26.38	2
7555	50	67	162	1.62	25.53	2
926	45	58	160	1.6	22.66	2
6672	48	64	155	1.55	26.64	2
8279	55	73	160	1.6	28.52	2
2197	48	79	170	1.7	27.34	2
6609	40	59	158	1.58	23.63	2
2330	60	46	160	1.6	17.97	2
4400	52	60	160	1.6	23.44	2
593	48	51	158	1.58	20.43	2
7241	47	70	160	1.6	27.34	2
4205	48	65	152	1.52	28.13	2
862	53	63.5	158	1.58	25.44	2
453	39	62	158	1.58	24.84	1
158	44	70	150	1.5	31.11	1
487	40	68	165	1.65	24.98	1
480	48	65	164	1.64	24.17	1
265	49	65	167	1.67	23.31	1
287	25	47	167	1.67	16.85	1
2757	66	65	154	1.54	27.41	1
1552	46	57	158	1.58	22.83	1
715	32	64	163	1.63	24.09	2
677	43	62	156	1.56	25.48	2
4801	47	82	162	1.62	31.25	2

4185	52	61.5	163	1.63	23.15	2
374	40	70	160	1.6	27.34	1
603	38	70	155	1.55	29.14	1
253	47	50	160	1.6	19.53	1
3396	47	43	150	1.5	19.11	1
503	36	75	163	1.63	28.23	1
71	50	64	162	1.62	24.39	1
575	52	68	155	1.55	28.30	1
257	50	70	160	1.6	27.34	1
235	38	71	159	1.59	28.08	1
555	39	55	160	1.6	21.48	1
1407	39	64	160	1.6	25.00	1
2840	25	57.5	165	1.65	21.12	1
2193	55	55	155	1.55	22.89	1
501	32	55	155	1.55	22.89	1
6154	36	62	163	1.63	23.34	1
3590	35	60	163	1.63	22.58	1
1998	44	71	170	1.7	24.57	1
6387	49	71	164	1.64	26.40	1
1047	38	65	163	1.63	24.46	2
709	38	55	158	1.58	22.03	2
138	47	67	160	1.6	26.17	2
162	46	95	162	1.62	36.20	2
6222	54	76	168	1.68	26.93	2
2819	44	76	168	1.68	26.93	2
864	29	57	174	1.74	18.83	2
6	47	63	168	1.68	22.32	2
3434	39	55	165	1.65	20.20	2
3928	51	79	161	1.61	30.48	2
758	58	52	149	1.49	23.42	2
599	50	62.5	160	1.6	24.41	2
4851	34	63	161	1.61	24.30	2
805	33	62	160	1.6	24.22	2
1413	53	64	165	1.65	23.51	2
4946	42	72.5	160	1.6	28.32	2
1766	49	64	166	1.66	23.23	2
202	42	80	172	1.72	27.04	2
795	44	60	165	1.65	22.04	2
5033	43	71	160	1.6	27.73	2
392	38	85.5	159	1.59	33.82	1
185	48	68	160	1.6	26.56	1
363	53	65	155	1.55	27.06	1
3363	33	61	163	1.63	22.96	2
1344	43	58	168	1.68	20.55	2
632	37	51	158	1.58	20.43	1
360	43	60	168	1.68	21.26	1
457	39	72	159	1.59	28.48	1
1388	53	64.5	160	1.6	25.20	1

891	41	59	155	1.55	24.56	1
6099	46	70	165	1.65	25.71	1
4480	46	55.5	160	1.6	21.68	1
3632	47	66	161	1.61	25.46	1
3625	34	56	166	1.66	20.32	1
77	32	71	160	1.6	27.73	1
3602	47	59.5	155	1.55	24.77	1
1419	50	70.5	158	1.58	28.24	1
695	49	71	165	1.65	26.08	1
5508	35	59	162	1.62	22.48	1
166	53	63	165	1.65	23.14	1
303	53	58	162	1.62	22.10	1
861	54	55.5	155	1.55	23.10	1
320	47	59	160	1.6	23.05	1
1298	36	51	163	1.63	19.20	1
923	48	72	163	1.63	27.10	1
1026	61	56	158	1.58	22.43	1
1432	42	62	158	1.58	24.84	1
1370	39	65	170	1.7	22.49	1
5284	52	59	160	1.6	23.05	1
35	51	46	160	1.6	17.97	1
1107	29	45	166	1.66	16.33	1
223	39	47	150	1.5	20.89	1
7496	49	57	159	1.59	22.55	1
838	51	54	153	1.53	23.07	1
3589	44	51	158	1.58	20.43	1
1174	45	64	152	1.52	27.70	1
663	49	60	158	1.58	24.03	2
511	46	58	159	1.59	22.94	2
1311	43	55	160	1.6	21.48	2
7627	42	52.5	160	1.6	20.51	2
4697	47	50	158	1.58	20.03	2
394	39	70	158	1.58	28.04	2
447	50	85	168	1.68	30.12	2
575	35	55	162	1.62	20.96	2
1650	45	51	160	1.6	19.92	2
834	48	78	162	1.62	29.72	2
432	37	63	165	1.65	23.14	2
574	37	57	165	1.65	20.94	2
1338	37	60	165	1.65	22.04	2
2961	43	67	160	1.6	26.17	2
2269	47	56	162	1.62	21.34	2
973	49	65	160	1.6	25.39	2
544	45	70	160	1.6	27.34	2
2548	49	68	163	1.63	25.59	2
1728	45	73	164	1.64	27.14	2
822	47	54	153	1.53	23.07	2
4700	40	54	160	1.6	21.09	2

3160	51	45	150	1.5	20.00	2
6286	51	62	153	1.53	26.49	2
415	55	61	155	1.55	25.39	2
926	46	76	156	1.56	31.23	2
1972	43	71	162	1.62	27.05	2
3172	45	63	155	1.55	26.22	2
8081	49	62	163	1.63	23.34	2
4878	47	58	150	1.5	25.78	2
7801	52	85	160	1.6	33.20	2
7410	51	70	160	1.6	27.34	2
563	51	54	159	1.59	21.36	1
388	30	58	160	1.6	22.66	1
463	41	75	168	1.68	26.57	1
449	45	56	154	1.54	23.61	1
1379	58	74	165	1.65	27.18	1
2699	30	71	164	1.64	26.40	2
2666	51	62	150	1.5	27.56	2
714	45	91	170	1.7	31.49	2
391	36	81	163	1.63	30.49	1
599	32	53	160	1.6	20.70	1
885	46	70	164	1.64	26.03	1
8123	43	65	152	1.52	28.13	1
76	47	74	165	1.65	27.18	1
372	46	70.5	163	1.63	26.53	1
1144	36	71	150	1.5	31.56	1
2	39	60	172	1.72	20.28	1
4763	54	58	160	1.6	22.66	1
213	53	78	162	1.62	29.72	1
3029	36	59	172	1.72	19.94	2
1593	44	64	160	1.6	25.00	2
8033	38	67	163	1.63	25.22	2
8090	48	65	162	1.62	24.77	2
533	44	57	162	1.62	21.72	2
580	33	76	158	1.58	30.44	2
6704	54	55	155	1.55	22.89	1
6396	50	65	162	1.62	24.77	2
2505	49	67	160	1.6	26.17	2
1197	47	80	160	1.6	31.25	1
498	50	68	160	1.6	26.56	1
4209	55	49.5	158	1.58	19.83	1
938	48	63	160	1.6	24.61	1
1700	48	59	159	1.59	23.34	1
1115	52	64	165	1.65	23.51	1
2748	48	66	170	1.7	22.84	1
5048	32	67	160	1.6	26.17	1
2555	58	80	160	1.6	31.25	2
5538	53	75	170	1.7	25.95	2
527	33	56	160	1.6	21.88	2

640	44	60	162	1.62	22.86	2
426	26	57	162	1.62	21.72	1
710	51	54	155	1.55	22.48	2
69	29	52	150	1.5	23.11	1
717	46	68	159	1.59	26.90	2
617	34	87	168	1.68	30.82	1
5573	46	92.5	165	1.65	33.98	2
151	45	63	160	1.6	24.61	1
26	55	73	157	1.57	29.62	1
469	50	68	160	1.6	26.56	1
156	27	70	167	1.67	25.10	1
5	35	95	170	1.7	32.87	1
666	45	61	163	1.63	22.96	1
1952	45	68	153	1.53	29.05	1
890	42	64	168	1.68	22.68	1
1955	48	75	162	1.62	28.58	1
1536	50	66	165	1.65	24.24	1
1572	51	48	161	1.61	18.52	1
352	43	75	168	1.68	26.57	1
130	42	70	163	1.63	26.35	1
1056	46	58	155	1.55	24.14	1
136	25	53	165	1.65	19.47	1
828	39	56	158	1.58	22.43	1
41	48	62	168	1.68	21.97	1
675	70	64.5	159	1.59	25.51	1
309	47	59.5	152	1.52	25.75	1
154	56	75	165	1.65	27.55	1
132	38	58	166	1.66	21.05	1
220	51	69	166	1.66	25.04	1
231	35	63	165	1.65	23.14	1
1498	50	66	158	1.58	26.44	1
192	36	65	163	1.63	24.46	1
358	48	59	160	1.6	23.05	1
629	50	59.5	155	1.55	24.77	1
1615	49	52	155	1.55	21.64	1
389	57	54	160	1.6	21.09	1
238	45	58	161	1.61	22.38	1
788	31	62	158	1.58	24.84	1
282	25	62	166	1.66	22.50	1
619	48	69	160	1.6	26.95	1
341	53	68	169	1.69	23.81	1
1608	37	52	155	1.55	21.64	1
1597	42	48	155	1.55	19.98	1
359	58	54	153	1.53	23.07	1
382	44	53	160	1.6	20.70	1
1145	44	73	151	1.51	32.02	2

299	51	76	166	1.66	27.58	2
339	47	74	168	1.68	26.22	2
464	51	52.5	164	1.64	19.52	2
473	32	56	163	1.63	21.08	2
1743	46	60	155	1.55	24.97	2
39	41	62.5	170	1.7	21.63	2
691	58	71	165	1.65	26.08	2
1262	50	60	152	1.52	25.97	2
1233	50	54	155	1.55	22.48	2
299	49	70	160	1.6	27.34	2
1037	52	55	158	1.58	22.03	2
598	53	65.5	170	1.7	22.66	2
258	46	72	168	1.68	25.51	2
1794	40	63	156	1.56	25.89	2
153	57	75	160	1.6	29.30	2
383	49	71	160	1.6	27.73	2
352	49	72	165	1.65	26.45	2
615	50	58	155	1.55	24.14	2
126	47	65	160	1.6	25.39	2
445	50	55	158	1.58	22.03	2
654	54	79	155	1.55	32.88	2
843	55	65	158	1.58	26.04	2
983	50	70	169	1.69	24.51	2
160	50	58	158	1.58	23.23	2
1216	50	65	163	1.63	24.46	2
1059	51	63	165	1.65	23.14	2
172	53	68	158	1.58	27.24	2
139	39	68	158	1.58	27.24	2
454	47	66	155	1.55	27.47	2
1207	45	60	165	1.65	22.04	2
237	50	51	161	1.61	19.68	2
113	52	85	162	1.62	32.39	2
390	55	62	160	1.6	24.22	2
718	50	79	163	1.63	29.73	2
1441	45	79	163	1.63	29.73	2
1003	58	84	164	1.64	31.23	2
1354	50	65	150	1.5	28.89	2
1293	51	63	158	1.58	25.24	2
1085	57	60	160	1.6	23.44	2
464	49	93.5	172	1.72	31.60	2
128	53	75	160	1.6	29.30	2
106	49	54	155	1.55	22.48	2
1143	46	71	160	1.6	27.73	2
263	58	76	158	1.58	30.44	2
530	55	65	162	1.62	24.77	2
1173	50	59	160	1.6	23.05	2

609	46	71.5	164	1.64	26.58	2
338	49	78	163	1.63	29.36	2
1028	70	50	155	1.55	20.81	2
578	56	64	158	1.58	25.64	2
987	45	76	164	1.64	28.26	2
509	47	80	155	1.55	33.30	2
3	48	58	165	1.65	21.30	2
537	49	66	158	1.58	26.44	2
77	46	57.5	162	1.62	21.91	2
1051	49	63	156	1.56	25.89	2
492	48	64	165	1.65	23.51	2
395	52	73	155	1.55	30.39	2
2882	37	52	165	1.65	19.10	1
7431	53	60	160	1.6	23.44	1
2271	49	60	160	1.6	23.44	1
7119	53	72.5	160	1.6	28.32	1
4462	47	68	162	1.62	25.91	1
4094	32	65	157	1.57	26.37	1
3452	34	53.5	170	1.7	18.51	1
1053	41	78	160	1.6	30.47	1
5237	49	57.5	160	1.6	22.46	1
4133	47	60	155	1.55	24.97	1
4962	47	76	172	1.72	25.69	1
3267	45	80	167	1.67	28.69	1
572	49	51	161	1.61	19.68	1
5845	53	59.5	165	1.65	21.85	1
5723	31	66	168	1.68	23.38	1
3017	54	71	155	1.55	29.55	1
5415	62	70	164	1.64	26.03	1
401	56	80	164	1.64	29.74	1
2904	43	53	158	1.58	21.23	1
1028	46	53	160	1.6	20.70	1
1760	44	63	160	1.6	24.61	1
2802	52	72.5	160	1.6	28.32	1
1026	42	50	155	1.55	20.81	1
1260	53	55.5	162	1.62	21.15	1
5217	44	56.5	164	1.64	21.01	1
3378	47	55	156	1.56	22.60	1
7197	52	79	163	1.63	29.73	1
2927	46	53	158	1.58	21.23	1
2233	51	64	157	1.57	25.96	1
4145	45	69.5	165	1.65	25.53	1
1574	49	66	158	1.58	26.44	1
6723	37	69	167	1.67	24.74	1
809	43	70	165	1.65	25.71	1
2563	46	53	156	1.56	21.78	1
376	38	72.5	174	1.74	23.95	1
1925	45	66	165	1.65	24.24	1

5149	42	69	160	1.6	26.95	1
2630	50	78	162	1.62	29.72	1
4353	30	48	162	1.62	18.29	1
4441	43	61	164	1.64	22.68	1
7390	49	76	160	1.6	29.69	1
7502	48	83	160	1.6	32.42	1
6127	74	66	163	1.63	24.84	1
4549	47	68	164	1.64	25.28	1
6049	31	58	160	1.6	22.66	1
1431	32	67	165	1.65	24.61	1
2978	53	67	165	1.65	24.61	1
788	46	79.5	165	1.65	29.20	1
4890	51	85	166	1.66	30.85	1
4142	59	62	157	1.57	25.15	1
2624	39	60	162	1.62	22.86	1
2489	34	56	163	1.63	21.08	1
3576	46	64	160	1.6	25.00	1
4138	47	64	155	1.55	26.64	1
6431	51	64	155	1.55	26.64	1
1337	63	57	147	1.47	26.38	1
4568	44	60	160	1.6	23.44	1
2338	37	60	166	1.66	21.77	1
4512	57	58	161	1.61	22.38	1
4734	29	55	164	1.64	20.45	1
4413	59	70	165	1.65	25.71	1
4678	53	50	150	1.5	22.22	1
3609	46	52	160	1.6	20.31	1
3990	43	80	163	1.63	30.11	1
304	53	60	160	1.6	23.44	1
3764	53	77.5	165	1.65	28.47	1
2317	52	64	155	1.55	26.64	1
1936	44	65	166	1.66	23.59	1
4891	48	50	160	1.6	19.53	1
7074	46	65	158	1.58	26.04	1
7719	53	64	160	1.6	25.00	1
4113	46	69	168	1.68	24.45	1
1762	41	51	160	1.6	19.92	1
690	30	58	167	1.67	20.80	1
5730	47	61	158	1.58	24.44	1
3592	45	69	163	1.63	25.97	1
6795	49	66	172	1.72	22.31	1
2449	46	55.5	160	1.6	21.68	1
2484	48	75	156	1.56	30.82	1
1161	49	64	151	1.51	28.07	1
3945	34	62	159	1.59	24.52	1
3494	47	70	155	1.55	29.14	1
342	49	60	155	1.55	24.97	1
8510	57	70	159	1.59	27.69	1

3968	40	93	168	1.68	32.95	1
3380	26	55	163	1.63	20.70	1
3831	55	79	162	1.62	30.10	1
4176	33	70	160	1.6	27.34	1
3047	39	59	155	1.55	24.56	1
3448	80	56	158	1.58	22.43	1
212	50	50	150	1.5	22.22	1
6419	45	78	160	1.6	30.47	1
3072	35	53	166	1.66	19.23	1
2800	56	60	164	1.64	22.31	1
5139	35	76	162	1.62	28.96	1
2886	22	69	160	1.6	26.95	1
6713	34	72.5	148	1.48	33.10	1
299	52	75	163	1.63	28.23	1
1504	40	61	160	1.6	23.83	1
1861	39	69	168	1.68	24.45	1
3768	41	76	163	1.63	28.60	1
5212	49	52.5	160	1.6	20.51	1
1626	68	71	160	1.6	27.73	1
2671	49	85	170	1.7	29.41	1
7115	39	70	163	1.63	26.35	1
2460	41	56	160	1.6	21.88	1
5298	50	61	162	1.62	23.24	1
7291	48	62	159	1.59	24.52	1
3829	45	70	164	1.64	26.03	1
4505	50	70	160	1.6	27.34	1
1612	53	53	160	1.6	20.70	1
3390	54	64	160	1.6	25.00	1
2320	35	82.5	158	1.58	33.05	1
8005	51	50	163	1.63	18.82	1
2122	48	53	158	1.58	21.23	1
4363	40	60	163	1.63	22.58	1
1039	40	75.5	162	1.62	28.77	1
1221	45	60	159	1.59	23.73	1
2566	39	65	158	1.58	26.04	1
4160	73	66	163	1.63	24.84	1
5081	52	60	160	1.6	23.44	1
1391	33	51	160	1.6	19.92	1
992	42	52	160	1.6	20.31	1
1102	51	70	167	1.67	25.10	1
5494	45	76	165	1.65	27.92	1
4822	42	61.5	165	1.65	22.59	1
2755	29	58	166	1.66	21.05	1
695	51	66	162	1.62	25.15	2
6596	51	55	160	1.6	21.48	2
2056	35	54	164	1.64	20.08	2
2519	43	68	162	1.62	25.91	2
321	43	71	162	1.62	27.05	2

1898	55	64	158	1.58	25.64	2
3078	41	70	155	1.55	29.14	2
1315	44	59	155	1.55	24.56	2
7227	53	75	163	1.63	28.23	2
2491	57	67.5	162	1.62	25.72	2
1554	45	55	158	1.58	22.03	2
4495	59	67	160	1.6	26.17	2
3496	52	72.5	162	1.62	27.63	2
4860	50	56	160	1.6	21.88	2
3090	60	73	168	1.68	25.86	2
4152	44	56	160	1.6	21.88	2
7045	43	88	161	1.61	33.95	2
1188	55	58	160	1.6	22.66	2
1957	56	71.5	160	1.6	27.93	2
3347	49	52	158	1.58	20.83	2
2482	36	95	164	1.64	35.32	2
3196	63	63	155	1.55	26.22	2
4198	55	60	158	1.58	24.03	2
851	53	63	150	1.5	28.00	2
1776	43	57	153	1.53	24.35	2
2476	46	61	160	1.6	23.83	2
545	47	74	170	1.7	25.61	2
2242	53	57	156	1.56	23.42	2
7968	44	70	168	1.68	24.80	2
3224	50	54	163	1.63	20.32	2
3169	45	68	163	1.63	25.59	2
4296	47	60	160	1.6	23.44	2
7389	48	66	162	1.62	25.15	2
2662	37	58	152	1.52	25.10	2
1052	55	74	160	1.6	28.91	2
8299	51	49	158	1.58	19.63	2
5593	40	50	156	1.56	20.55	2
3754	49	80	172	1.72	27.04	2
6661	49	47	150	1.5	20.89	2
3210	46	78	162	1.62	29.72	2
3417	41	72	165	1.65	26.45	2
730	56	70	162	1.62	26.67	2
4032	55	59	160	1.6	23.05	2
1747	49	70	163	1.63	26.35	2
5142	46	85	163	1.63	31.99	2
4402	57	66	160	1.6	25.78	2
2423	42	68	164	1.64	25.28	2
4495	48	72	162	1.62	27.43	2
2895	44	52	163	1.63	19.57	2
4837	45	61	157	1.57	24.75	2
2867	51	80	158	1.58	32.05	2
2098	45	70	173	1.73	23.39	2
5706	28	49	160	1.6	19.14	2

3881	50	60	160	1.6	23.44	2
1875	45	58	158	1.58	23.23	2
7498	55	47	150	1.5	20.89	2
3033	39	58	158	1.58	23.23	2
3557	52	73	162	1.62	27.82	2
5103	45	70	161	1.61	27.01	2
4565	50	75	158	1.58	30.04	2
3734	51	71	165	1.65	26.08	2
5341	57	70	158	1.58	28.04	2
811	49	66	158	1.58	26.44	2
1714	51	55	155	1.55	22.89	2
5472	50	64	162	1.62	24.39	2
5060	50	56	157	1.57	22.72	2
3147	36	51	157	1.57	20.69	2
4609	53	62	165	1.65	22.77	2
1745	60	65	160	1.6	25.39	2
3639	47	74	163	1.63	27.85	2
2617	53	55	170	1.7	19.03	2
4671	52	54.5	165	1.65	20.02	2
751	48	75	164	1.64	27.89	2
5487	51	65	165	1.65	23.88	2
4110	51	64	162	1.62	24.39	2
3535	49	61	163	1.63	22.96	2
4366	42	70	155	1.55	29.14	2
3547	52	60	162	1.62	22.86	2
5354	51	65	168	1.68	23.03	2
4871	48	55	157	1.57	22.31	2
1971	46	68	160	1.6	26.56	2
4842	52	60	160	1.6	23.44	2
1660	46	60	159	1.59	23.73	2
8674	50	67	160	1.6	26.17	2
3343	55	70	165	1.65	25.71	2
1390	46	55	160	1.6	21.48	2
4908	38	45	160	1.6	17.58	2
6223	47	67	168	1.68	23.74	2
4103	48	67	163	1.63	25.22	2
4708	56	56	158	1.58	22.43	2
2731	51	55	160	1.6	21.48	2
3108	49	51	152	1.52	22.07	2
234	46	63	158	1.58	25.24	2
4468	51	74.5	164	1.64	27.70	2
2551	44	57	162	1.62	21.72	2
1950	54	57	166	1.66	20.69	2
2653	41	69	162	1.62	26.29	2
3714	51	60	153	1.53	25.63	2
2676	44	82	163	1.63	30.86	2
2957	53	70	162	1.62	26.67	2
7230	52	66	156	1.56	27.12	2

4395	44	60	160	1.6	23.44	2
4159	38	55	164	1.64	20.45	2
4090	50	74	168	1.68	26.22	2
3290	47	64	158	1.58	25.64	2
1416	51	65	160	1.6	25.39	2
3181	47	60	155	1.55	24.97	2
6655	56	69.5	166	1.66	25.22	2
5516	49	71	163	1.63	26.72	2
4547	52	58	164	1.64	21.56	2
1004	49	63	164	1.64	23.42	2
4831	46	69	163	1.63	25.97	2
2261	56	56	154	1.54	23.61	2
4171	44	60	163	1.63	22.58	2
1510	50	59	160	1.6	23.05	2
6671	34	69	171	1.71	23.60	2
3095	57	58	160	1.6	22.66	2
2130	50	64	160	1.6	25.00	2
5258	50	68	163	1.63	25.59	2
4206	68	80	155	1.55	33.30	2
4292	42	74	168	1.68	26.22	2
4538	55	73	165	1.65	26.81	2
5589	41	74	162	1.62	28.20	2
5225	37	54	164	1.64	20.08	2
3836	44	70	163	1.63	26.35	2
2547	53	63	155	1.55	26.22	2
902	55	66	158	1.58	26.44	2
542	53	69.5	165	1.65	25.53	2
7615	52	64	165	1.65	23.51	2
4869	55	65	157	1.57	26.37	2
2039	47	60	152	1.52	25.97	2
2287	44	58	160	1.6	22.66	2
4151	52	52	155	1.55	21.64	2
3965	50	66	163	1.63	24.84	2
2357	31	65.5	165	1.65	24.06	2
1989	49	60	159	1.59	23.73	2
3379	41	66	167	1.67	23.67	2
5274	52	74	163	1.63	27.85	2
742	47	74	160	1.6	28.91	2
7886	51	58	153	1.53	24.78	2
1521	49	71.5	164	1.64	26.58	2
4223	44	72	171	1.71	24.62	2
1217	54	75	160	1.6	29.30	2
1021	47	76	160	1.6	29.69	2
3675	55	65	162	1.62	24.77	2
3380	43	65	155	1.55	27.06	2
1017	37	64	173	1.73	21.38	2
4188	52	63	160	1.6	24.61	2
4324	34	104	167	1.67	37.29	2

786	48	71	160	1.6	27.73	2
5384	50	62	163	1.63	23.34	2
913	50	72.5	160	1.6	28.32	2
5496	45	64	162	1.62	24.39	2
1970	53	64	150	1.5	28.44	2
8231	51	47	163	1.63	17.69	2
819	54	72	165	1.65	26.45	2
1173	55	59	169	1.69	20.66	2
3056	48	55	163	1.63	20.70	2
3610	50	60	160	1.6	23.44	2
5525	42	53	153	1.53	22.64	2
2891	46	65	160	1.6	25.39	2
2328	41	55	165	1.65	20.20	2
5311	51	70	158	1.58	28.04	2
457	47	74.5	165	1.65	27.36	2
2974	50	60	160	1.6	23.44	2
1183	46	70	160	1.6	27.34	2
4304	38	47	157	1.57	19.07	2
4463	48	74.5	170	1.7	25.78	2
1514	43	60	155	1.55	24.97	2
3035	53	73	159	1.59	28.88	2
5579	43	76	173	1.73	25.39	2
1196	45	70	156	1.56	28.76	2
1142	49	85	170	1.7	29.41	2
1999	73	85	164	1.64	31.60	2
999	51	74	160	1.6	28.91	2
5153	45	60	160	1.6	23.44	2
3156	50	60	155	1.55	24.97	2
1389	51	62	156	1.56	25.48	2
3607	51	64	158	1.58	25.64	2
2445	33	53	163	1.63	19.95	2
5238	50	55	155	1.55	22.89	2
7187	50	80	170	1.7	27.68	2
3600	45	74	165	1.65	27.18	2
4746	39	73	160	1.6	28.52	2
3827	52	69	153	1.53	29.48	2
5948	43	69	162	1.62	26.29	2
2196	48	63	168	1.68	22.32	2
4865	51	65	155	1.55	27.06	2
2573	46	63	150	1.5	28.00	2
5160	49	88	170	1.7	30.45	2
5384	66	75	160	1.6	29.30	2
2688	43	60	168	1.68	21.26	2
5351	43	66	160	1.6	25.78	2
3189	37	69	156	1.56	28.35	2
1054	30	60	158	1.58	24.03	2
3254	45	70	160	1.6	27.34	2
4826	46	82.5	164	1.64	30.67	2

5090	51	71	155	1.55	29.55	2
4699	44	64	158	1.58	25.64	2
3658	51	68	170	1.7	23.53	2
3339	57	67	155	1.55	27.89	2
4663	49	48	155	1.55	19.98	2
974	52	60	153	1.53	25.63	2
4969	49	58	155	1.55	24.14	2
3431	45	67	168	1.68	23.74	2
5338	52	64	170	1.7	22.15	2
4187	57	65	155	1.55	27.06	2
6694	49	55	157	1.57	22.31	2
5062	35	63	165	1.65	23.14	2
2337	53	65	160	1.6	25.39	2
5716	37	55	155	1.55	22.89	2
4477	48	68	165	1.65	24.98	2
3509	41	55	156	1.56	22.60	2
7358	53	61	158	1.58	24.44	2
2810	52	60	153	1.53	25.63	2
5582	48	53	155	1.55	22.06	2
2619	41	62	160	1.6	24.22	2
2527	49	61	155	1.55	25.39	2
5650	50	76	157	1.57	30.83	2
4016	51	72	158	1.58	28.84	2
678	41	55	156	1.56	22.60	2
3428	50	62.5	157	1.57	25.36	2
6912	63	80	160	1.6	31.25	2
3858	45	79	163	1.63	29.73	2
4798	57	60	165	1.65	22.04	2
3325	43	64	158	1.58	25.64	2
2657	55	58	155	1.55	24.14	2
2644	45	56	155	1.55	23.31	2
5651	45	77	168	1.68	27.28	2
2508	50	57	160	1.6	22.27	2
5333	54	71	165	1.65	26.08	2
4588	45	70	158	1.58	28.04	2
3791	49	62	160	1.6	24.22	2
2124	46	72	165	1.65	26.45	2
1212	57	67	158	1.58	26.84	2
3369	56	80	170	1.7	27.68	2
3413	46	82	158	1.58	32.85	2
181	47	58.5	155	1.55	24.35	2
4150	52	71	160	1.6	27.73	2
2190	45	56	158	1.58	22.43	2
7207	49	55.5	156	1.56	22.81	2
3774	44	65	165	1.65	23.88	2
5765	31	51	163	1.63	19.20	2
2293	48	65	160	1.6	25.39	2
941	53	67	163	1.63	25.22	2

5009	40	51	164	1.64	18.96	2
3375	50	59	155	1.55	24.56	2
1520	53	55	162	1.62	20.96	2
554	46	67	160	1.6	26.17	2
5052	48	60.5	155	1.55	25.18	2
4124	46	65	164	1.64	24.17	2
2542	60	66	162	1.62	25.15	2
3447	48	80	165	1.65	29.38	2
5552	51	75	160	1.6	29.30	2
206	49	65	159	1.59	25.71	2
3156	50	65	160	1.6	25.39	2
2522	42	55	158	1.58	22.03	2
2750	46	46	152	1.52	19.91	2
5047	45	55	155	1.55	22.89	2
3674	49	52	154	1.54	21.93	2
3520	41	60	160	1.6	23.44	2
3261	47	59	160	1.6	23.05	2
5666	51	60	158	1.58	24.03	2
6940	50	65	160	1.6	25.39	2
1820	44	60	162	1.62	22.86	2
2427	41	55	160	1.6	21.48	2
4307	40	60	163	1.63	22.58	2
3563	53	55	160	1.6	21.48	2
4586	45	64	163	1.63	24.09	2
6881	40	71	165	1.65	26.08	2
2916	41	65	150	1.5	28.89	2
1717	32	58	156	1.56	23.83	2
1224	51	74	160	1.6	28.91	2
5240	51	72	160	1.6	28.13	2
4276	47	75	160	1.6	29.30	2
5664	46	72	166	1.66	26.13	2
1720	44	69	163	1.63	25.97	2
6731	49	60	164	1.64	22.31	2
817	62	81	160	1.6	31.64	2
7213	51	84	168	1.68	29.76	2
4017	51	67	160	1.6	26.17	2
3239	50	72	169	1.69	25.21	2
1944	42	62	168	1.68	21.97	2
2179	48	62	155	1.55	25.81	2
5005	51	61	155	1.55	25.39	2
3246	59	62	156	1.56	25.48	2
6208	43	54	160	1.6	21.09	2
5686	33	86	162	1.62	32.77	2
5798	50	55	155	1.55	22.89	2
681	55	69	160	1.6	26.95	2
8043	52	79	165	1.65	29.02	2
1895	54	80	160	1.6	31.25	2
2027	48	71	155	1.55	29.55	2

949	45	65	162	1.62	24.77	2
4006	53	62	165	1.65	22.77	2
59	51	69	154	1.54	29.09	2
6318	55	72	162	1.62	27.43	2
2860	54	59	155	1.55	24.56	2
2113	44	59	148	1.48	26.94	2
5009	51	52	155	1.55	21.64	2

JLZDZJ	JWS	YJGL	JL	TJ	Platelet	Lymphocyte
0.3	0	1	2	0	209	1.24
0.3	0	1	2	1	369	2.59
0.4	0	0	2	0	280	1.51
0.4	0	0	2	1	214	1.42
0.5	0	1	2	0	274	1.61
0.5	0	1	2	0	355	2.16
0.5	0	0	2	0	273	1.7
0.5	0	1	2	0	276	1.47
0.5	0	0	2	1	291	2.91
0.5	0	1	2	0	362	1.29
0.5	0	1	2	0	253	2.01
0.5	0	1	2	0	273	1.78
0.6	0	0	3	1	222	1.94
0.8	0	1	2	0	432	1.88
0.8	0	1	2	0	200	1.99
0.8	0	1	2	0	175	1.14
0.8	0	1	2	0	377	2.35
0.8	0	1	2	0	304	2.17
0.8	0	1	2	0	294	2.08
0.9	0	0	2	0	292	1.49
1	0	1	2	0	239	1.77
1	0	1	2	0	253	1.78
1	0	1	2	0	222	0.92
1	0	0	2	0	243	1.65
1	0	1	2	0	305	2.09
1	0	1	2	0	232	2.15
1	0	1	2	0	226	2.1
1	0	1	3	0	236	2.96
1	0	0	2	0	265	2.34
1	0	1	2	0	154	1.7
1	0	1	2	0	357	1.3
1	0	1	3	0	282	1.81
1	0	0	2	0	394	2.48
1	0	1	2	0	170	2.19
1	0	1	2	0	227	2.19
1	0	1	2	0	285	2.57
1	0	1	2	0	295	1.91
1	0	1	3	0	237	1.26
1	0	1	2	0	311	1.55
1	0	1	2	0	280	1.5
1	0	1	2	0	314	1.87
1	0	1	2	0	364	2.19
1	0	1	2	1	330	1.9
1	0	1	3	0	164	1.35
1	0	1	2	0	252	2.07
1	0	1	2	0	239	4.16
1	0	1	3	0	316	1.64

1	0	1	2	0	235	2.36
1	0	0	2	0	246	1.36
1	0	0	2	1	384	1.97
1	0	1	2	0	231	3.11
1	0	1	2	1	285	1.26
1	0	1	2	0	219	1.31
1	0	1	2	0	220	2.09
1	0	1	2	0	352	1.85
1	0	1	3	0	378	1.33
1	0	1	2	0	251	1.21
1	0	1	2	0	222	1.27
1	0	1	1	0	259	1.46
1	0	1	3	1	239	0.69
1	0	1	2	0	295	3.16
1	0	1	2	0	221	2.1
1.2	0	0	3	0	216	1.3
1.5	0	1	2	0	338	1.13
1.5	0	0	2	1	308	2
1.5	0	1	2	0	233	2.22
1.5	0	1	2	0	246	1.16
1.5	0	1	2	0	232	1.39
1.5	0	1	2	0	348	1.36
1.5	0	1	2	0	193	1.29
1.5	0	1	2	0	217	1.62
1.5	0	1	2	0	265	1.55
1.5	0	1	2	0	242	1.56
1.5	0	1	2	0	216	0.94
1.5	0	1	2	1	303	2.16
1.5	0	1	2	0	234	2.54
1.5	0	1	2	0	169	1.54
1.5	0	1	2	0	304	1.88
1.5	0	1	2	0	231	2.04
1.5	0	1	2	0	160	1.85
1.5	0	1	2	0	292	1.08
1.5	0	1	2	0	380	1.9
1.5	0	1	3	0	425	2.03
1.5	0	1	2	0	216	1.95
1.5	0	1	2	0	304	2.8
1.5	0	1	2	0	241	2.69
1.5	0	1	2	0	85	0.94
1.5	0	1	2	0	299	2.76
1.5	0	1	2	0	306	1.97
1.5	0	1	2	0	242	1.32
1.5	0	1	2	0	133	1.45
1.5	0	1	2	0	251	2.72
1.5	0	1	2	0	225	2.22
1.5	0	1	2	0	246	1.22
1.5	0	1	2	0	259	2.94

1.5	0	1	2	0	272	2.2
1.8	0	0	3	1	265	1.11
1.8	0	1	2	0	154	1.43
1.9	0	0	2	0	206	1.95
1.9	0	0	2	0	399	1.96
2	0	0	2	0	216	1.35
2	0	1	3	1	276	2.07
2	0	1	2	0	171	1.67
2	0	0	2	0	296	2.32
2	0	0	2	0	211	2.06
2	0	0	2	0	213	1.64
2	0	1	2	0	261	1.44
2	0	0	2	1	254	2.25
2	0	1	2	1	230	1.4
2	0	1	2	0	437	1.64
2	0	1	2	1	265	2.05
2	0	1	2	0	157	1.78
2	0	1	2	0	227	1.36
2	0	1	2	0	350	1.97
2	0	1	2	0	288	2.11
2	0	1	2	1	271	2.36
2	0	1	2	1	282	1.54
2	0	1	2	0	301	2.18
2	0	1	2	0	189	2.03
2	0	0	1	0	268	2.77
2	0	1	1	0	146	2.56
2	0	1	2	0	270	2.53
2	0	0	2	1	246	1.92
2	0	1	2	0	296	1.45
2	0	1	2	0	228	1.45
2	0	1	2	0	250	1.97
2	0	1	2	0	461	1.79
2	0	1	2	0	280	1.27
2	0	1	2	0	256	2.15
2	0	1	2	0	321	2.33
2	0	1	2	0	296	2.95
2	0	1	2	1	315	2.5
2	0	1	2	1	338	1.7
2	0	1	2	0	213	1.68
2	0	1	2	0	312	1.3
2	0	1	2	0	266	1.44
2	0	1	2	0	363	2.13
2	0	1	2	0	192	1.98
2	0	1	2	0	283	2.49
2	0	1	2	0	235	1.28
2	0	1	2	0	310	1.72
2	0	1	2	1	381	1.66
2	0	1	3	1	302	2.6

2	0	1	2	0	402	2.63
2	0	1	2	0	148	1.17
2	0	1	2	0	225	1.6
2	0	0	2	1	338	1.77
2	0	1	2	0	328	2.27
2	0	1	2	0	131	0.72
2	0	1	2	1	324	2.08
2	0	1	2	0	326	1.33
2	0	1	2	0	239	1.1
2	0	1	3	0	287	1.44
2	0	1	2	0	170	1.32
2	0	1	1	0	120	1.89
2	0	1	2	0	298	1.08
2	0	1	2	0	186	1.6
2	0	1	2	0	282	3.24
2	0	1	3	0	367	1.7
2	0	1	1	0	294	1.31
2	0	1	2	0	359	1.36
2	0	0	2	0	294	1.6
2	0	1	2	1	391	1.77
2.2	0	0	2	0	249	2.6
2.5	0	1	2	0	204	1.1
2.5	0	1	2	0	200	1.62
2.5	0	1	2	0	338	1.66
2.5	0	1	2	0	235	1.86
2.5	0	1	2	0	226	1.29
2.5	0	1	2	0	315	1.74
2.5	0	1	2	0	228	1.39
2.5	0	1	3	0	343	1.54
2.5	0	1	2	1	255	1.95
2.5	0	1	2	0	228	1.13
2.5	0	1	2	0	309	3.29
2.5	0	1	2	0	187	1.21
2.5	0	0	2	0	237	2.13
2.5	0	1	2	0	232	1.85
2.5	0	1	2	0	172	1.98
2.5	0	1	2	0	280	2.74
2.5	0	1	2	0	309	0.97
2.5	0	1	2	0	237	2.02
2.5	0	1	2	0	441	2.63
2.5	0	1	2	0	301	1.62
2.5	0	1	2	1	184	1.96
2.5	0	0	2	0	249	1.11
2.5	0	1	2	0	216	2.07
2.5	0	1	2	0	248	1.84
2.5	0	1	2	0	232	1.54
2.5	0	1	2	0	228	1.39
2.5	0	1	2	0	275	1.72

2.5	0	1	2	0	277	1.76
2.5	0	1	2	0	247	1.59
2.6	0	0	2	0	260	2.55
2.9	0	0	2	0	260	1.67
2.9	0	0	2	0	317	1.3
3	0	1	2	0	256	1.78
3	0	0	2	1	268	1.88
3	0	1	2	0	238	1.18
3	0	1	2	0	295	1.54
3	0	1	2	1	376	1.81
3	0	1	2	0	224	2.33
3	0	1	2	1	177	1.3
3	0	1	2	1	348	2.11
3	0	1	2	0	273	1.7
3	0	1	2	0	279	1.71
3	0	1	2	0	297	1
3	0	1	2	0	332	3.37
3	0	1	2	1	431	1.89
3	0	1	2	1	174	2.3
3	0	1	2	0	261	1.39
3	0	1	2	0	178	2.4
3	0	1	2	0	268	1
3	0	1	2	0	218	1.65
3	0	1	2	1	265	1.47
3	0	1	3	1	219	1.05
3	0	1	2	0	246	1.11
3	0	1	2	0	323	2.11
3	0	1	2	0	251	3.11
3	0	1	3	0	278	1.28
3	0	1	2	0	226	1.61
3	0	1	2	0	298	1.44
3	0	1	2	0	436	2.21
3	0	1	2	0	268	2.13
3	0	1	2	0	317	1.68
3	0	1	3	0	295	2.05
3	0	1	2	0	402	3.14
3	0	1	2	0	369	0.85
3	0	1	2	0	361	1.52
3	0	1	2	0	293	2.63
3	0	1	2	0	184	2.25
3	0	1	2	0	283	3.27
3	0	1	2	0	314	4.14
3	0	1	2	0	277	0.95
3	0	1	2	0	447	2.38
3	0	1	2	0	183	1.01
3	0	1	2	0	419	2.52
3	0	1	2	0	364	1.27
3	0	1	2	1	267	2.94

3	0	1	2	0	310	1.84
3	0	1	2	0	440	2.57
3	0	1	2	0	193	2.59
3	0	1	2	0	203	1.76
3	0	1	2	0	250	1.41
3	0	1	3	1	368	1.95
3	0	1	2	0	326	1.27
3	0	1	2	0	241	2.6
3	0	1	2	1	274	2.81
3	0	1	2	0	288	2.13
3	0	1	2	0	209	1.42
3	0	1	2	0	195	1.78
3	0	1	3	0	229	1.11
3	0	1	2	0	307	1.38
3	0	1	2	0	296	1.73
3	0	1	2	0	379	2.06
3	0	1	2	0	213	1.75
3	0	1	2	1	205	1.75
3	0	1	2	0	272	3.16
3	0	1	2	0	196	1.57
3	0	1	2	0	320	2.63
3	0	1	2	1	349	1.7
3	0	1	2	0	153	0.53
3	0	1	3	1	280	2.26
3	0	1	2	0	423	3.45
3	0	1	2	0	289	1.83
3	0	1	2	0	235	1.46
3	0	1	2	0	308	2.03
3	0	1	2	0	230	1.81
3.2	0	0	2	0	445	1.38
3.2	0	0	2	0	273	2.12
3.3	0	0	3	0	308	2.94
3.5	0	1	2	1	304	1.19
3.5	0	0	2	0	252	1.09
3.5	0	1	2	0	263	1.86
3.5	0	1	2	0	236	3.89
3.5	0	1	3	1	356	2.54
3.5	0	1	2	0	159	0.94
3.5	0	1	2	1	369	3.11
3.5	0	1	2	0	328	2.1
3.5	0	1	2	0	266	2.23
3.5	0	1	2	0	231	1.82
3.5	0	1	2	0	246	1.95
3.5	0	1	2	0	210	1.81
3.5	0	1	2	0	392	1.85
3.5	0	0	3	0	235	1.45
3.5	0	1	2	0	180	1.7
3.5	0	0	2	0	259	1.54

3.5	0	1	2	1	471	2.45
3.5	0	1	2	0	253	1.23
3.5	0	1	2	0	302	2.41
3.6	0	0	2	1	261	2.34
3.6	0	0	2	0	205	1.58
3.6	0	0	2	0	370	2.31
3.7	0	0	2	0	289	1.86
3.8	0	0	2	1	196	1.55
3.8	0	0	1	1	227	3.47
4	0	1	2	0	134	1.16
4	0	1	2	0	279	1.96
4	0	1	2	0	133	1.48
4	0	1	2	0	266	2.51
4	0	0	3	1	189	1.71
4	0	0	2	1	426	2.63
4	0	1	2	0	223	1.9
4	0	1	3	1	266	1.34
4	0	0	2	0	239	2.86
4	0	1	1	0	203	1.67
4	0	0	3	0	254	1.8
4	0	1	2	0	330	1.2
4	0	1	2	0	196	1.44
4	0	0	2	0	207	1.96
4	0	0	2	1	261	2.1
4	0	0	2	0	357	2.24
4	0	1	2	0	367	0.73
4	0	1	2	0	157	1.72
4	0	1	2	0	184	1.96
4	0	0	2	0	202	1.6
4	0	0	2	0	318	1.4
4	0	1	3	0	192	1.53
4	0	1	2	0	316	1.6
4	0	1	2	0	166	2.03
4	0	1	2	0	255	1.36
4	0	1	2	0	325	1.96
4	0	1	3	0	257	2.37
4	0	1	2	0	246	2.1
4	0	1	2	0	335	1.73
4	0	1	2	0	391	1.84
4	0	0	2	0	239	1.89
4	0	1	3	1	301	1.31
4	0	0	2	0	230	1.6
4	0	1	2	0	297	1.85
4	0	1	2	0	271	1.17
4	0	1	2	0	231	2.66
4	0	1	2	0	144	1.61
4	0	1	2	0	238	1.78
4	0	1	2	0	277	1.56

4	0	1	2	0	346	1.97
4	0	1	2	0	51	1.05
4	0	1	2	0	397	1.37
4	0	1	2	0	273	2.43
4	0	1	2	0	247	2.15
4	0	1	2	0	208	2.65
4	0	1	2	0	222	2.88
4	0	1	3	0	306	1.48
4	0	1	2	0	167	1.34
4	0	1	2	0	283	1.36
4	0	1	2	0	237	1.2
4	0	1	2	0	264	1.87
4	0	0	2	0	194	2.53
4	0	1	2	0	206	2.06
4	0	0	2	0	349	2.03
4	0	1	3	1	337	1.31
4	0	1	2	0	240	1.59
4	0	1	2	0	301	0.57
4	0	1	2	0	312	1.88
4	0	0	2	0	184	1.84
4	0	1	2	0	297	1.08
4	0	1	3	0	321	2.64
4	0	1	2	0	199	2.18
4	0	1	2	0	344	1.8
4	0	0	2	0	305	2.27
4	0	1	2	1	263	1.57
4	0	1	2	0	247	1.57
4	0	0	2	0	237	2.22
4	0	1	3	0	406	1.62
4	0	1	2	1	349	0.5
4	0	1	2	0	216	1.57
4	0	1	2	0	226	0.87
4	0	1	2	0	274	1.38
4	0	1	2	0	303	2.2
4	0	0	2	0	202	1.47
4	0	1	2	0	188	2.08
4	0	1	2	1	334	1.09
4	0	1	2	0	297	1.96
4	0	1	2	0	307	2.69
4	0	1	2	0	284	1.89
4	0	1	2	0	218	1.3
4	0	1	2	0	243	1.34
4	0	1	3	0	308	1.25
4	0	1	2	0	296	2.27
4	0	1	2	0	256	1.2
4	0	1	2	0	240	1.25
4	0	1	2	0	228	2.81
4	0	1	2	0	231	3.16

4	0	1	2	0	226	1.85
4	0	1	2	1	270	2.58
4.3	0	0	2	0	207	1.41
4.3	0	0	2	0	265	2.54
4.3	0	0	2	0	261	1.84
4.4	0	0	3	1	272	2.47
4.5	0	1	2	0	321	1.71
4.5	0	0	3	0	319	2.47
4.5	0	1	2	1	332	2.77
4.5	0	0	2	1	317	2.09
4.5	0	1	2	0	131	1.26
4.5	0	1	3	1	353	2.31
4.5	0	0	3	0	234	1.81
4.5	0	1	2	0	284	1.86
4.5	0	1	2	0	343	1.49
4.5	0	1	2	0	343	1.74
4.5	0	1	2	0	185	1.66
4.5	0	1	2	1	470	3.31
4.5	0	1	2	0	326	1.51
4.5	0	1	3	1	228	2.14
4.5	0	1	2	0	258	2.08
4.6	0	0	2	0	223	1.88
4.6	0	1	2	0	324	1.45
4.6	0	0	2	1	206	2.38
4.6	0	0	2	0	258	1.61
4.6	0	0	2	0	414	2.15
4.7	0	0	2	0	284	2.81
4.7	0	0	3	1	332	1.72
4.7	0	0	2	0	205	1.94
4.7	0	0	2	0	197	2.14
4.7	0	0	2	0	364	1.46
4.8	0	0	3	0	371	1.71
4.8	0	0	2	0	328	1.39
4.8	0	0	2	0	267	3.04
4.8	0	0	2	1	202	1.61
4.8	0	0	2	0	190	1.61
4.9	0	0	2	0	249	2.74
4.9	0	0	2	0	359	1.29
4.9	0	0	2	0	224	1.51
4.9	0	0	2	0	216	1.61
4.9	0	0	2	1	209	2.61
4.9	0	0	2	0	244	1.48
4.9	0	0	1	1	274	1.67
4.9	0	0	2	1	301	1.79
4.9	0	0	2	0	458	1.47
5	0	1	2	0	145	1.77
5	0	0	2	1	208	2.3
5	0	1	2	0	304	0.97

5	0	1	2	0	314	2.72
5	0	0	2	0	243	1.47
5	0	0	2	0	362	1.65
5	0	0	2	1	178	1.97
5	0	1	2	0	206	1.58
5	0	1	2	0	216	1.95
5	0	0	2	1	267	2.04
5	0	1	2	0	192	1.15
5	0	1	2	1	173	1.78
5	0	1	2	0	222	2.14
5	0	1	2	0	274	2.29
5	0	1	2	0	313	1.97
5	0	1	2	0	307	2.17
5	0	1	2	0	191	1.81
5	0	1	2	0	184	1.29
5	0	1	2	0	170	2
5	0	1	2	0	208	1.68
5	0	1	2	0	396	1.99
5	0	1	2	0	265	1.92
5	0	0	2	0	134	1.83
5	0	0	2	0	245	2.48
5	0	0	2	0	376	1.86
5	0	1	2	0	307	1.35
5	0	1	2	0	205	2.39
5	0	0	2	0	240	1.7
5	0	1	2	0	395	1.86
5	0	0	2	1	234	1.54
5	0	0	2	0	377	3.34
5	0	1	2	0	263	1.1
5	0	1	2	0	497	2.38
5	0	1	2	0	295	1.45
5	0	1	2	0	193	1.73
5	0	1	2	0	294	1.26
5	0	1	3	0	423	2.41
5	0	1	2	0	482	2.28
5	0	0	2	0	290	1.76
5	0	1	2	0	207	0.68
5	0	1	2	0	126	1.98
5	0	1	2	0	227	1.62
5	0	0	2	1	344	2.88
5	0	0	2	1	252	1.36
5	0	1	2	0	183	1.19
5	0	0	2	0	260	1.01
5	0	1	2	1	303	1.13
5	0	1	2	0	270	2.01
5	0	1	3	0	270	2.07
5	0	0	2	0	194	1.67
5	0	1	2	0	366	1.35

5	0	0	2	0	275	2.1
5	0	0	3	1	277	2.43
5	0	1	2	1	287	2.81
5	0	1	2	0	261	2.72
5	0	0	2	1	215	1.95
5	0	1	2	0	220	1.93
5	0	1	2	0	165	1.69
5	0	1	2	0	262	1.94
5	0	1	2	0	218	1.66
5	0	1	2	0	350	2.3
5	0	1	2	0	235	1.89
5	0	1	2	0	94	1.25
5	0	1	2	0	288	1.98
5	0	1	2	1	181	1.21
5	0	1	2	0	250	1.33
5	0	1	2	1	288	1.84
5	0	1	2	0	375	1.94
5	0	1	2	1	263	2.01
5	0	0	1	0	268	1.58
5	0	1	2	0	253	1.99
5	0	1	3	0	298	1.73
5	0	1	1	0	229	1.93
5	0	1	2	1	394	1.66
5	0	1	2	0	266	1.3
5	0	1	2	0	144	2.18
5	0	1	1	0	239	2.16
5	0	1	2	0	194	1.37
5	0	0	3	0	214	2.33
5	0	0	2	0	350	1.2
5	0	1	2	0	284	2.08
5	0	1	2	0	261	2.38
5	0	0	2	0	163	2.38
5	0	1	1	0	319	3.43
5	0	1	2	0	292	1.55
5	0	1	2	0	238	2.1
5	0	1	2	0	266	1.38
5	0	1	2	0	227	1.29
5	0	1	2	0	271	1.21
5	0	1	2	0	197	1.95
5	0	1	2	0	204	2.04
5	0	1	2	0	323	2.37
5	0	1	2	0	212	2.15
5	0	1	2	0	211	2.01
5	0	1	2	0	360	1.87
5	0	1	2	0	359	1.96
5	0	1	2	0	335	2.39
5	0	1	2	0	315	2.32
5	0	1	2	0	291	3.07

5	0	1	2	0	366	2.41
5	0	1	2	0	254	2.27
5	0	1	2	0	272	1.57
5	0	1	2	0	249	2.85
5	0	1	2	0	284	1.58
5	0	1	2	0	233	2.1
5	0	1	2	0	390	2.2
5	0	1	1	0	370	2.41
5	0	1	2	0	263	2.16
5	0	1	2	0	174	1.66
5	0	1	2	1	225	1.94
5	0	1	2	0	325	1.4
5	0	1	2	0	497	1.77
5	0	1	2	0	195	2.16
5	0	1	3	1	365	2.76
5	0	1	2	0	234	1.36
5	0	0	2	1	296	2.05
5	0	0	2	0	251	1.88
5	0	0	2	0	231	1.48
5	0	0	3	1	323	1.16
5	0	1	2	0	274	1.88
5	0	0	2	0	223	1.61
5	0	0	2	1	384	1.51
5	0	0	2	0	181	1.55
5	0	1	2	0	271	1.64
5	0	1	3	0	247	1.23
5	0	1	2	1	227	1.18
5	0	0	1	0	206	2.22
5	0	0	2	1	202	1.38
5	0	1	2	0	178	1.34
5	0	1	2	0	233	1.97
5	0	1	2	0	209	1.11
5	0	1	2	0	231	1.84
5	0	1	2	0	223	0.87
5	0	1	3	0	104	1.43
5	0	1	2	0	306	2.06
5	0	0	2	0	226	1.34
5	0	0	2	0	254	1.84
5	0	0	2	0	320	2.22
5	0	1	2	0	264	2.53
5	0	0	2	0	260	2.25
5	0	0	2	1	207	1.89
5	0	0	2	0	416	2.23
5	0	1	2	1	158	2.44
5	0	1	2	0	228	1.13
5	0	1	2	0	240	2.04
5	0	1	2	0	264	3.08
5	0	0	2	0	243	2.27

5	0	0	2	0	282	1.59
5	0	0	2	0	218	1.94
5	0	1	2	1	199	1.005
5	0	1	2	0	514	1.11
5	0	1	2	0	308	1.32
5	0	1	2	0	267	1.81
5	0	1	2	0	249	1.65
5	0	1	2	0	293	1.56
5	0	1	2	0	292	2.41
5	0	0	3	0	386	1.46
5	0	1	2	0	260	2
5	0	0	2	0	247	2.57
5	0	0	2	0	230	1.68
5	0	1	2	1	241	1.96
5	0	0	2	0	417	3.17
5	0	1	2	0	215	2.13
5	0	1	2	1	329	1.6
5	0	1	2	0	207	1.31
5	0	1	2	0	289	1.28
5	0	1	2	0	194	2.36
5	0	1	2	0	189	1.01
5	0	0	2	1	325	3.36
5	0	1	2	0	257	1.26
5	0	0	2	0	399	2.17
5	0	1	2	0	290	1.26
5	0	1	1	0	234	2.31
5	0	1	2	1	320	2.26
5	0	1	2	0	278	2.25
5	0	1	2	0	243	2.25
5	0	1	2	0	282	1.57
5	0	1	2	0	294	1.47
5	0	0	3	1	453	1.4
5	0	0	2	0	312	2.5
5	0	0	3	1	260	1.27
5	0	1	2	0	320	2.18
5	0	1	2	0	275	1.04
5	0	0	2	0	400	2.53
5	0	1	2	0	307	3.31
5	0	1	2	0	195	1.34
5	0	1	3	0	291	1.33
5	0	1	2	0	338	1.31
5	0	1	3	1	273	1.24
5	0	1	2	0	182	1.71
5	0	0	2	0	278	1.5
5	0	1	2	0	283	2.41
5	0	1	2	0	238	1.16
5	0	1	2	0	271	2.34
5	0	1	2	1	304	2.36

5	0	1	1	0	300	1.67
5	0	1	2	0	226	2.5
5	0	1	3	1	405	1.49
5	0	1	2	0	384	1.39
5	0	1	2	0	192	2.64
5	0	1	2	1	302	2.46
5	0	1	2	0	244	1.56
5	0	1	2	0	244	1.54
5	0	1	2	1	223	1.67
5	0	1	2	1	333	2.21
5	0	1	2	0	303	2.04
5	0	1	2	0	262	2.81
5	0	1	2	1	418	2.18
5	0	1	2	0	442	2.2
5	0	1	2	0	282	1.73
5	0	1	2	0	271	1.39
5	0	1	2	0	150	1.26
5	0	1	2	0	331	1.22
5	0	1	2	1	265	1.89
5	0	1	2	0	215	2.63
5	0	1	3	1	329	0.63
5	0	0	3	1	277	2.16
5	0	1	2	0	233	2.56
5	0	1	2	0	274	2.21
5	0	1	2	1	257	1.14
5	0	1	2	0	291	2.27
5	0	1	2	0	345	1.7
5	0	1	2	1	219	1.37
5	0	1	2	0	242	0.9
5	0	1	2	0	302	1.48
5	0	1	2	0	446	1.6
5	0	1	2	0	395	2.96
5	0	1	2	0	354	2.8
5	0	1	2	0	386	2.26
5	0	1	2	0	350	1.69
5	0	1	2	0	314	2.21
5	0	1	2	0	306	1.55
5	0	1	2	0	426	1.4
5	0	1	2	0	257	1.7
5	0	1	2	0	206	0.96
5	0	1	2	0	305	2.75
5	0	1	2	0	240	2.19
5	0	1	2	0	182	1.16
5	0	1	2	1	306	2.85
5.1	0	0	2	0	209	2.59
5.1	0	0	2	0	315	2.43
5.1	0	0	2	0	385	1.8
5.1	0	0	2	1	176	2.52

5.2	0	0	2	0	140	2.41
5.2	0	0	2	0	259	1.41
5.2	0	0	2	1	180	1.05
5.2	0	0	2	1	174	2.01
5.2	0	0	2	0	236	1.56
5.2	0	0	2	0	356	1.5
5.2	0	0	2	0	233	1.73
5.2	0	0	2	0	218	1.75
5.3	0	0	2	0	252	2.46
5.3	0	0	2	0	182	2.79
5.3	0	0	2	0	198	1.98
5.3	0	0	3	0	368	2.78
5.3	0	1	3	0	456	1.84
5.3	0	0	3	0	380	1.3
5.3	0	0	2	0	241	2.46
5.3	0	0	2	0	396	1.8
5.4	0	0	2	0	441	1.51
5.4	0	0	3	1	259	2.19
5.4	0	0	2	0	332	2.33
5.4	0	0	2	0	350	1.11
5.4	0	0	3	0	387	1.44
5.4	0	0	2	0	295	2.43
5.5	0	0	2	0	225	2.29
5.5	0	0	2	0	352	1.02
5.5	0	0	2	0	299	2.42
5.5	0	1	2	0	243	1.59
5.5	0	1	2	0	284	1.81
5.5	0	0	2	0	280	1.89
5.5	0	1	2	0	259	1.54
5.5	0	1	2	0	389	2.59
5.5	0	1	2	0	217	1.23
5.5	0	1	1	0	206	2.31
5.5	0	0	2	1	208	2.46
5.5	0	0	2	0	308	1.32
5.5	0	0	2	0	249	1.09
5.5	0	1	2	0	345	2.18
5.6	0	0	2	0	345	1.26
5.6	0	0	3	0	188	1.72
5.6	0	0	2	0	230	1.56
5.6	0	0	2	1	444	1.9
5.6	0	1	2	0	247	1.46
5.6	0	0	2	0	284	1.6
5.6	0	0	2	0	258	1.3
5.6	0	0	2	1	281	1.48
5.6	0	0	2	0	202	1.03
5.6	0	0	2	0	396	1.69
5.7	0	0	2	0	239	1.64
5.7	0	0	3	1	233	1.84

5.7	0	0	2	0	260	2.2
5.7	0	0	2	0	215	2.97
5.8	0	0	2	0	328	2.34
5.8	0	0	2	0	198	1.38
5.8	0	0	2	0	389	1.45
5.8	0	1	2	0	425	1.92
5.8	0	0	2	0	315	1.57
5.9	0	0	2	0	394	2.7
5.9	0	0	2	0	237	1.36
5.9	0	0	2	1	355	1.69
5.9	0	0	3	0	329	1.55
5.9	0	0	3	0	192	2.62
6	0	1	3	0	406	1.22
6	0	0	2	0	351	1.18
6	0	1	2	0	248	2.2
6	0	1	2	0	305	1.55
6	0	1	2	0	217	1.63
6	0	0	2	0	341	1.52
6	0	0	3	0	325	1.81
6	0	1	2	0	227	0.89
6	0	0	2	0	220	1.64
6	0	0	2	0	331	1.58
6	0	1	1	1	279	2.63
6	0	0	2	0	287	2.03
6	0	1	2	1	263	2.81
6	0	0	3	1	308	1.84
6	0	1	2	1	160	1.29
6	0	1	2	0	331	1.88
6	0	1	2	1	354	2.32
6	0	1	3	1	270	1.93
6	0	1	2	0	226	3.77
6	0	1	2	0	299	1.73
6	0	1	2	0	310	2.82
6	0	1	2	1	264	1.87
6	0	0	2	0	246	1.97
6	0	0	2	0	224	1.98
6	0	0	2	0	232	2.17
6	0	1	2	0	126	1.68
6	0	0	2	0	224	1.91
6	0	0	3	0	370	1.39
6	0	0	2	0	332	1.77
6	0	1	2	0	248	1.91
6	0	1	2	0	324	2.81
6	0	1	2	0	228	1.54
6	0	1	2	0	270	2.51
6	0	1	2	0	351	1.68
6	0	0	2	0	243	1.73
6	0	0	2	0	276	1.8

6	0	1	2	0	297	1.77
6	0	0	2	0	338	2.17
6	0	0	2	0	272	1.93
6	0	1	2	1	198	1.59
6	0	1	2	1	209	1.8
6	0	1	2	0	206	3.34
6	0	1	2	0	240	1.28
6	0	1	2	0	309	2.05
6	0	0	2	0	270	1.5
6	0	0	2	0	251	2.18
6	0	1	2	0	274	0.69
6	0	1	2	0	231	2.16
6	0	1	2	0	416	1.81
6	0	1	2	0	188	1.38
6	0	0	2	0	200	1.84
6	0	1	2	0	181	1.41
6	0	1	2	0	246	1.13
6	0	1	2	0	260	1.19
6	0	1	2	0	308	2.34
6	0	1	2	0	247	2.25
6	0	1	3	1	198	1.55
6	0	1	2	0	292	1.18
6	0	1	2	0	236	1.6
6	0	0	2	0	207	1.17
6	0	1	2	0	289	2.18
6	0	1	2	0	302	1.91
6	0	1	2	0	250	0.76
6	0	1	2	0	270	1.19
6	0	1	2	1	391	1.45
6	0	0	2	0	398	3.26
6	0	1	2	0	281	2.37
6	0	1	2	0	269	1.52
6	0	1	2	0	352	1.31
6	0	1	3	1	227	2.47
6	0	1	2	0	316	2.38
6	0	1	2	1	285	1.92
6	0	1	2	0	137	1.34
6	0	1	2	0	272	1.58
6	0	1	2	0	290	2.74
6	0	0	2	0	345	1.72
6	0	1	2	0	231	1.89
6	0	1	3	1	338	2.89
6	0	1	2	0	275	1.87
6	0	1	2	0	217	2.79
6	0	0	2	0	248	2.37
6	0	0	3	0	192	2.72
6	0	1	2	0	303	2.12
6	0	0	2	1	217	1.93

6	0	1	2	0	317	3.2
6	0	1	2	0	286	2.02
6	0	1	2	0	301	1.53
6	0	1	2	0	379	2.26
6	0	1	2	0	324	2.36
6	0	1	2	0	318	2.05
6	0	1	2	0	339	1.92
6	0	1	2	0	473	1.75
6	0	1	2	0	216	2.33
6	0	1	2	0	268	0.58
6	0	1	2	0	381	2.34
6	0	1	2	0	274	2.73
6	0	1	2	0	163	1.38
6	0	1	2	0	290	1.53
6	0	1	2	0	195	0.88
6	0	1	2	1	355	1.93
6	0	1	2	0	191	2.43
6	0	1	2	0	212	2.24
6	0	0	1	1	180	1.48
6	0	1	2	1	173	1.94
6	0	1	2	0	318	2.32
6	0	1	2	0	268	2.35
6	0	1	2	0	342	2.26
6	0	1	2	0	309	2.47
6	0	1	2	0	316	1.29
6	0	1	2	0	329	3.71
6	0	1	2	0	369	1.58
6	0	1	2	0	319	1.71
6	0	1	2	0	274	1.76
6	0	1	2	0	337	2.61
6	0	1	2	0	174	2.1
6	0	1	2	0	256	1.57
6	0	1	2	0	326	3.37
6	0	1	2	0	317	0.75
6	0	0	2	0	377	1.27
6	0	1	3	1	372	1.18
6	0	0	3	0	208	1.6
6	0	0	2	0	292	1.99
6	0	1	2	0	226	1.16
6	0	1	2	0	353	1.67
6	0	1	2	0	325	1.5
6	0	1	2	0	218	1.67
6	0	1	2	0	263	2.19
6	0	1	2	1	163	1.57
6	0	1	2	0	306	1.55
6	0	1	2	0	255	1.83
6	0	0	2	0	252	2.03
6	0	0	2	0	290	1.73

6	0	1	2	1	201	1.74
6	0	1	2	0	275	2.04
6	0	1	2	0	294	0.89
6	0	0	2	1	274	1.83
6	0	1	3	1	227	2.35
6	0	1	2	0	325	2
6	0	1	2	0	154	1.05
6	0	1	2	0	215	1.97
6	0	0	2	1	265	2.06
6	0	1	2	0	344	1.75
6	0	1	2	0	309	2.32
6	0	1	3	0	200	2.14
6	0	1	2	0	178	1.7
6	0	1	2	0	206	1.33
6	0	1	2	0	285	1.55
6	0	0	2	0	240	1.98
6	0	1	2	0	442	2.29
6	0	1	3	0	409	1.93
6	0	1	2	0	216	1.65
6	0	0	2	1	276	1.79
6	0	1	2	0	203	1.82
6	0	1	2	0	267	1.72
6	0	1	3	0	454	1.87
6	0	1	2	0	446	2.14
6	0	1	2	0	281	1.95
6	0	1	2	0	243	1.99
6	0	1	2	1	200	1.67
6	0	0	3	0	372	1.83
6	0	0	2	1	305	1.77
6	0	1	2	0	315	1.4
6	0	1	2	1	258	2.27
6	0	1	3	0	351	1.97
6	0	1	2	0	173	0.84
6	0	1	2	0	230	1.24
6	0	1	2	0	350	1.9
6	0	1	2	1	313	2.17
6	0	1	2	0	343	1.76
6	0	0	2	0	338	1.77
6	0	1	2	1	254	1.46
6	0	0	2	0	196	2.42
6	0	1	2	0	308	1.11
6	0	1	3	1	254	1.65
6	0	0	2	0	221	2.16
6	0	1	2	0	221	1.44
6	0	1	2	0	261	2.82
6	0	1	2	1	450	2.48
6	0	0	2	0	213	1.29
6	0	1	2	0	161	1.85

6	0	1	2	0	340	1.98
6	0	1	2	0	283	2.26
6	0	1	2	0	299	1.46
6	0	1	2	0	223	2.06
6	0	1	2	0	214	1.72
6	0	1	2	0	461	1.96
6	0	1	3	0	228	1.67
6	0	1	2	1	241	0.73
6	0	1	2	0	352	1.79
6	0	1	3	1	219	0.73
6	0	1	3	1	230	1.83
6	0	1	2	0	358	2.1
6	0	1	2	0	304	2.02
6	0	1	2	0	176	1.84
6	0	1	2	1	209	2.66
6	0	1	2	0	98	0.48
6	0	1	2	0	374	2.46
6	0	1	2	0	288	1.15
6	0	1	2	0	255	2.39
6	0	1	3	0	277	3.06
6	0	1	3	1	299	2.30
6	0	1	2	0	224	2.04
6	0	1	3	0	409	1.95
6	0	1	2	0	515	1.85
6	0	1	2	0	285	1.89
6	0	1	3	0	244	1.32
6	0	1	2	0	367	1.4
6	0	1	2	0	228	1.6
6	0	1	2	0	174	0.73
6	0	1	2	0	313	2.89
6	0	1	2	0	206	1.74
6	0	1	2	0	315	2.97
6	0	1	2	0	371	2.06
6	0	1	2	0	366	1.27
6	0	1	2	1	460	2.28
6	0	1	3	0	243	2.41
6	0	1	2	0	177	1.2
6	0	1	2	1	252	2.11
6	0	1	2	0	267	1.87
6	0	1	1	0	200	1.81
6	0	1	2	1	300	1.97
6	0	1	2	0	263	1.84
6	0	1	2	1	138	0.81
6	0	1	2	0	331	2.01
6	0	1	2	0	211	1.88
6	0	1	2	0	458	1.82
6	0	1	2	0	313	3.77
6	0	1	1	0	345	3.09

6	0	1	2	1	245	3.94
6	0	1	2	0	338	0.84
6.1	0	0	2	0	279	2.05
6.1	0	0	2	1	291	1.86
6.1	0	0	2	0	336	2.9
6.1	0	0	2	0	491	2.03
6.2	0	0	2	0	223	1.48
6.2	0	0	3	0	409	2.98
6.2	0	0	2	0	281	1.87
6.3	0	0	2	0	238	2.21
6.3	0	0	3	0	323	1.2
6.3	0	0	2	0	228	2.08
6.3	0	0	2	0	259	1.77
6.3	0	0	2	0	215	1.82
6.4	0	0	2	1	366	1.31
6.4	0	0	3	0	322	1.49
6.4	0	1	2	0	344	2.35
6.5	0	1	2	0	363	3.75
6.5	0	0	2	0	292	1.42
6.5	0	0	2	0	230	2.63
6.5	0	1	2	0	275	2.05
6.5	0	1	2	0	229	1.96
6.5	0	0	2	0	180	2.2
6.5	0	1	2	0	252	2.32
6.5	0	1	2	0	210	2.28
6.5	0	1	2	0	307	1.61
6.5	0	1	2	0	239	1.82
6.5	0	1	1	1	345	2.47
6.6	0	0	2	0	376	2.49
6.6	0	1	2	0	414	2.16
6.6	0	0	2	0	205	1.69
6.7	0	0	2	0	220	1.53
6.7	0	0	3	0	239	1.55
6.7	0	0	2	0	250	2.65
6.8	0	0	2	0	190	1.81
6.8	0	0	3	0	384	1.92
6.8	0	0	2	0	306	1.44
6.8	0	0	2	1	276	1.83
6.8	0	0	2	0	223	1.86
6.8	0	0	2	0	166	1.75
6.9	0	0	2	0	287	2.37
7	0	1	3	0	244	1.65
7	0	0	2	0	289	1.86
7	0	0	2	1	199	1.99
7	0	1	2	0	256	1.19
7	0	1	2	0	205	1.46
7	0	1	2	0	234	1.5
7	0	1	2	0	152	1.17

7	0	0	1	1	250	1.45
7	0	1	2	0	334	1.63
7	0	1	2	0	307	1.05
7	0	0	2	0	200	0.7
7	0	1	2	0	359	2.09
7	0	1	2	0	348	1.67
7	0	1	2	0	91	0.67
7	0	1	2	0	195	1.31
7	0	1	2	0	278	1.72
7	0	1	2	1	385	2.06
7	0	1	2	1	434	0.92
7	0	0	2	0	300	1.18
7	0	0	2	0	191	2.32
7	0	0	3	0	235	1.95
7	0	1	2	0	237	1.29
7	0	1	2	0	160	1.51
7	0	1	2	0	140	2.12
7	0	0	2	0	287	1.72
7	0	0	2	0	375	2.4
7	0	1	2	0	176	1.72
7	0	1	2	1	216	2.54
7	0	1	2	0	185	1.59
7	0	1	2	0	199	1.97
7	0	1	2	0	366	1.6
7	0	1	2	0	198	1.53
7	0	1	2	0	390	2.84
7	0	0	2	0	227	1.2
7	0	1	3	0	128	1.56
7	0	1	2	0	322	2.38
7	0	1	3	1	364	1.93
7	0	1	2	0	160	1.7
7	0	1	2	0	248	2.44
7	0	1	2	0	197	2.16
7	0	1	3	1	292	1.73
7	0	1	3	1	321	2.1
7	0	1	2	0	176	1.3
7	0	1	2	0	204	2.11
7	0	1	2	1	406	1.96
7	0	1	2	0	301	3.09
7	0	1	2	0	257	2.23
7	0	1	2	1	262	1.42
7	0	1	2	0	171	1.52
7	0	1	2	1	268	1.54
7	0	1	2	0	263	1.99
7	0	1	2	0	208	2.75
7	0	1	2	0	315	2.37
7	0	1	2	0	255	3.23
7	0	1	2	0	337	1.36

7	0	1	2	1	470	2.75
7	0	1	2	0	161	1.29
7	0	1	2	0	289	2.26
7	0	1	2	0	270	2.09
7	0	1	2	0	303	0.87
7	0	1	2	0	219	2
7	0	1	2	1	375	1.63
7	0	0	2	0	252	2.9
7	0	0	2	0	224	2.21
7	0	1	2	0	281	1.78
7	0	1	2	0	169	0.79
7	0	0	2	0	250	2.06
7	0	1	2	1	260	2.12
7	0	1	2	0	272	2.54
7	0	1	2	0	204	2.29
7	0	1	2	0	397	0.88
7	0	1	2	0	278	2.5
7	0	1	2	1	267	1.66
7	0	1	2	0	168	1.5
7	0	1	2	0	181	1.27
7	0	1	3	0	220	1.33
7	0	0	2	0	215	1.91
7	0	0	2	0	234	1.88
7	0	1	2	0	344	1.85
7	0	1	2	0	291	1.61
7	0	1	2	0	349	2.71
7	0	0	2	1	420	1.72
7	0	1	2	1	222	1.18
7	0	0	2	0	139	1.68
7	0	1	3	0	207	2.4
7	0	0	2	0	204	1.05
7	0	1	2	0	202	1.74
7	0	1	2	0	280	1.63
7	0	1	2	0	261	2.48
7	0	1	3	1	334	1.46
7	0	1	2	0	285	1.88
7	0	0	2	0	336	1
7	0	1	2	0	329	2.45
7	0	0	2	0	237	1.44
7	0	1	2	0	184	1.61
7	0	1	2	0	150	1.36
7	0	1	2	0	248	1.55
7	0	1	2	0	265	2.43
7	0	1	2	0	236	1.1
7	0	1	2	0	243	1.77
7	0	1	2	0	250	0.84
7	0	1	2	0	256	1.72
7	0	1	2	0	278	2.57

7	0	1	2	0	412	1.97
7	0	1	2	0	272	3.23
7	0	1	2	0	276	2.33
7	0	1	3	1	202	1.49
7	0	1	2	0	288	1.71
7	0	1	2	0	376	2.79
7	0	1	2	0	252	1.71
7	0	1	2	1	247	2.28
7	0	1	2	0	266	1.96
7	0	1	2	0	338	2
7	0	1	2	0	185	1.69
7	0	1	2	0	383	1.39
7	0	1	2	0	420	2.01
7	0	0	2	0	271	1.94
7	0	1	2	0	394	1.08
7	0	1	2	0	233	2.75
7	0	1	2	0	282	2.34
7	0	1	2	0	270	2.44
7	0	1	2	0	193	1.14
7	0	1	2	0	364	1.14
7	0	1	2	0	427	1.5
7	0	1	1	0	225	2.73
7	0	1	2	0	293	3.23
7	0	1	2	1	291	2.3
7.1	0	0	2	1	256	1.87
7.1	0	0	2	0	199	1.66
7.1	0	0	2	0	260	1.64
7.2	0	0	2	0	320	2.57
7.2	0	0	3	0	270	1.59
7.2	0	1	1	0	301	2.34
7.3	0	0	2	0	334	1.63
7.3	0	0	2	0	271	2.48
7.4	0	1	2	0	200	1.4
7.5	0	1	2	0	235	1.72
7.5	0	1	2	0	329	1.84
7.5	0	1	2	0	163	1.77
7.5	0	1	3	0	349	1.51
7.7	0	0	2	0	219	2.47
7.7	0	0	2	1	400	1.19
7.8	0	0	2	0	263	2.08
7.8	0	0	3	0	353	1.46
7.8	0	0	2	0	288	2.24
7.9	0	0	1	0	451	2.26
7.9	0	0	2	0	374	2.08
8	0	0	2	0	206	1.91
8	0	1	2	0	312	2.53
8	0	1	2	0	246	1.9
8	0	0	2	0	221	1.65

8	0	1	2	0	165	1.22
8	0	1	2	0	319	1.65
8	0	1	2	0	249	1.85
8	0	1	2	0	281	1.71
8	0	1	2	0	310	2.06
8	0	0	2	0	419	2.17
8	0	0	2	1	230	1.9
8	0	0	2	0	338	2.25
8	0	0	2	0	165	2.01
8	0	0	2	0	282	2.24
8	0	1	2	0	325	1.23
8	0	1	2	0	245	1.73
8	0	1	2	0	266	1.73
8	0	1	2	0	515	1.7
8	0	1	1	0	270	2.35
8	0	1	2	0	235	1.9
8	0	0	2	0	371	1.42
8	0	1	2	0	276	2.42
8	0	0	2	0	229	2.08
8	0	1	2	0	248	1.66
8	0	1	2	0	214	2.63
8	0	1	2	0	285	2.15
8	0	1	2	1	296	2.77
8	0	1	2	0	229	1.99
8	0	1	2	0	379	2.78
8	0	1	2	0	200	1.05
8	0	1	3	1	350	1.25
8	0	1	2	0	209	1.24
8	0	1	2	0	259	2.13
8	0	1	2	1	342	1.24
8	0	1	2	0	332	1.6
8	0	1	2	0	266	1.02
8	0	1	2	1	382	2.39
8	0	1	2	0	202	1.63
8	0	1	2	0	299	1.36
8	0	1	2	0	266	2.34
8	0	1	1	0	227	2.43
8	0	1	2	0	316	2.1
8	0	0	2	1	353	1.58
8	0	1	2	0	280	1.49
8	0	1	2	0	242	1.85
8	0	0	2	0	213	1.79
8	0	1	2	0	242	1.9
8	0	1	2	0	372	1.5
8	0	1	2	1	214	2.26
8	0	0	2	0	197	2.59
8	0	1	3	1	333	0.98
8	0	0	2	0	198	2.08

8	0	1	2	0	221	1.56
8	0	1	2	0	292	1.95
8	0	1	2	0	311	2.74
8	0	1	2	0	325	0.87
8	0	1	2	1	204	2.78
8	0	1	2	0	309	1.92
8	0	0	2	0	357	1.73
8	0	1	2	0	162	1.99
8	0	0	2	1	325	1.35
8	0	1	2	0	164	1.64
8	0	1	2	0	187	2.03
8	0	1	2	0	298	2.12
8	0	1	2	0	213	1.76
8	0	1	2	0	370	1.91
8	0	1	3	0	352	1.12
8	0	1	2	1	207	1.94
8	0	1	2	0	453	1.4
8	0	1	2	0	266	1.73
8	0	0	3	0	391	1.36
8	0	1	2	0	319	2.21
8	0	1	2	0	289	2.35
8	0	1	2	0	213	1.87
8	0	1	3	0	329	2.69
8	0	1	2	0	293	2.29
8	0	1	2	0	221	2.30
8	0	1	2	0	282	1.26
8	0	1	2	0	459	1.67
8	0	1	3	0	318	1.69
8	0	1	3	0	287	2.5
8	0	1	3	0	248	1.45
8	0	1	3	0	166	2.29
8	0	1	2	0	209	1.3
8	0	1	2	0	212	2.14
8	0	1	2	0	243	1.27
8	0	1	2	0	418	2.86
8	0	1	2	1	299	2.87
8	0	1	2	0	288	1.82
8.1	0	0	2	0	278	2.33
8.2	0	0	3	0	191	1.38
8.3	0	0	2	0	228	2
8.3	0	0	2	1	324	1.99
8.5	0	1	3	1	170	1.68
8.5	0	0	2	1	325	2.48
8.5	0	1	2	0	240	3.7
8.5	0	1	2	1	210	2.07
8.5	0	1	2	0	487	1.8
8.5	0	0	3	0	490	2.42
8.5	0	1	2	0	473	1.77

8.5	0	1	2	0	308	2.05
8.7	0	0	2	0	276	1.88
8.8	0	0	2	0	284	1.81
8.9	0	0	2	0	248	1.06
9	0	1	3	0	176	1.42
9	0	0	2	0	213	1.73
9	0	0	2	0	303	2.28
9	0	1	2	0	244	0.95
9	0	1	2	0	241	1.97
9	0	1	2	0	258	1.48
9	0	1	2	0	265	1.62
9	0	1	2	1	248	1.19
9	0	1	2	0	213	2.53
9	0	1	2	0	274	1.53
9	0	0	2	1	235	2.46
9	0	1	2	1	231	1.74
9	0	1	2	0	233	2.17
9	0	1	2	0	387	1.34
9	0	1	2	1	281	1.98
9	0	1	2	0	249	2.16
9	0	0	2	0	255	1.22
9	0	0	2	0	264	2.04
9	0	1	2	0	344	1.99
9	0	1	2	0	228	1.22
9	0	1	2	0	186	1.76
9	0	1	2	1	217	1.84
9	0	1	2	0	289	2.32
9	0	1	2	0	241	1.64
9	0	1	3	1	531	1.14
9	0	1	2	0	202	1.85
9	0	1	2	0	200	1.76
9	0	1	2	0	476	1.01
9	0	1	2	0	332	1.85
9	0	1	2	0	371	1.86
9	0	1	1	0	274	3.31
9	0	1	2	0	173	1.55
9	0	1	2	0	290	1.24
9	0	1	2	0	225	1.62
9	0	1	2	0	367	0.83
9.2	0	0	2	0	271	1.91
9.5	0	0	2	0	347	1.62
9.5	0	0	1	0	210	2.55
9.5	0	1	2	0	213	1.9
9.5	0	1	2	0	242	1.5
9.6	0	1	3	0	442	1.9
9.7	0	0	2	0	214	2
10	0	0	2	0	354	2.09
10	0	1	2	0	228	1.02

10	0	1	2	0	258	1.75
10	0	1	2	0	340	2.02
10	0	1	2	0	224	1.49
10	0	1	2	0	246	1.73
10	0	1	2	0	302	2.16
10	0	0	2	0	286	2.43
10	0	1	2	0	268	1.6
10	0	1	2	0	479	0.78
10	0	1	2	0	212	2.01
10	0	1	2	0	414	1.29
10	0	1	2	1	399	1.08
10	0	1	2	0	225	1.77
10	0	1	2	0	230	1.23
10	0	1	2	0	314	1.45
10	0	1	2	0	204	0.97
10	0	1	2	0	150	1.75
10	0	1	2	0	199	1.48
10	0	1	2	0	255	2.28
10	0	1	2	0	302	2.42
10	0	1	2	0	286	2.38
10	0	1	2	0	344	0.69
10	0	1	2	0	493	1.62
10	0	1	2	0	286	1.53
10	0	1	2	0	396	2.09
10	0	1	2	0	246	1.34
10	0	1	2	0	255	1.65
10	0	1	2	1	293	2.15
10	0	0	2	0	333	1.71
10	0	0	3	0	491	1.4
10	0	1	2	1	231	1.02
10	0	1	2	1	289	1.49
10	0	1	2	0	360	2.1
10	0	0	2	0	261	2.17
10	0	0	2	0	421	2.06
10	0	1	2	0	121	1.11
10	0	1	2	0	297	1.66
10	0	1	2	0	248	0.77
10	0	0	2	1	308	1.87
10	0	1	2	0	303	2.23
10	0	1	2	0	270	1.05
10	0	1	2	0	303	1.8
10	0	1	2	0	194	2.11
10	0	1	2	0	280	1.14
10	0	0	2	0	174	1.01
10	0	1	2	1	248	1.39
10	0	1	2	0	387	1.86
10	0	1	3	1	480	1.05
10	0	1	2	0	259	1.86

10	0	1	2	0	300	1.78
10	0	1	2	0	276	1.9
10	0	1	2	0	356	1.36
10	0	1	2	0	463	0.98
10	0	1	3	0	281	2.57
10	0	1	2	0	359	1.95
10	0	1	2	0	229	0.91
10	0	1	2	1	331	2.13
10	0	1	2	0	356	2.55
10	0	1	2	0	368	2.05
10.1	0	0	2	0	244	1.57
10.3	0	0	2	0	339	1.43
10.5	0	0	2	0	314	3
10.6	0	0	2	0	200	1.96
11	0	1	2	0	216	2.11
11	0	1	2	1	226	1.65
11	0	1	2	1	304	2.2
11	0	0	2	0	452	2.89
11.5	0	0	2	0	352	1.7
12	0	0	2	0	289	2.03
12	0	1	2	0	350	2.18
12	0	1	2	0	411	1.57
12	0	0	2	1	250	1.92
12	0	1	2	0	304	2.06
12	0	1	2	0	227	1.45
12	0	1	2	0	265	1.75
12	0	1	2	1	375	1.33
12	0	1	2	0	304	1.55
12	0	1	2	1	208	1.55
12	0	1	2	0	164	1.45
12	0	1	2	0	271	1.68
12	0	1	2	0	381	1.59
12	0	0	2	0	249	1.65
12.7	0	0	2	0	286	2.53
13	0	1	3	0	472	1.28
13	0	1	2	0	388	2.77
13	0	1	2	0	218	0.84
14	0	1	2	0	346	2.32
14	0	0	2	0	280	2.69
15	0	1	2	0	373	2.51
15	0	1	2	0	246	1.95
15	0	1	2	0	297	1.09
15	0	1	2	0	315	2.22
15	0	1	2	0	216	1.45
15	0	1	2	0	260	2.08
15	0	1	2	0	352	3
15	0	1	2	0	220	2.3
15	0	0	2	0	299	1.12

15	0	0	2	0	326	1.86
15.7	0	0	2	1	328	2.1
18	0	0	2	1	199	2.25
20	0	0	2	1	248	2.2
20	0	0	2	0	396	1.24
22.3	0	0	2	0	296	2.03
9	0	1	1	0	338	2.55
NA	0	1	2	0	216	1.05
NA	0	1	2	0	169	1.45
NA	0	1	2	0	407	1.68
NA	0	1	2	0	184	1.67
NA	0	1	2	0	239	1.41
NA	0	1	2	0	208	1.39
NA	0	1	2	0	225	1.96
NA	0	1	2	0	346	1.54
NA	0	1	2	0	262	1.67
NA	0	1	2	0	205	1.58
NA	0	1	2	0	306	2
NA	0	1	2	0	360	1.48
NA	0	1	2	0	286	1.91
NA	0	1	2	0	374	1.44
NA	0	1	2	0	265	2.29
NA	0	1	2	0	302	1.8
NA	0	1	2	0	232	1.26
NA	0	1	2	0	289	2.42
NA	0	1	2	0	223	2.13
NA	0	1	2	0	213	1.65
NA	0	1	2	0	276	1.25
NA	0	1	2	0	158	0.5
NA	0	1	2	0	243	3.26
NA	0	1	2	0	251	1.69
NA	0	1	2	0	194	1.89
NA	0	1	2	0	320	2.12
NA	0	1	2	0	255	2.37
NA	0	1	2	0	327	1.47
NA	0	1	2	0	222	1.75
NA	0	1	2	0	279	1.52
NA	0	1	2	0	188	1.53
NA	0	1	2	0	298	2.33
NA	0	1	2	1	236	0.9
NA	0	1	3	1	386	1.32
NA	0	1	2	0	321	0.53
NA	0	1	2	0	335	1.76
NA	0	1	2	0	216	1.36
NA	0	1	2	0	56	0.77
NA	0	1	2	0	255	1.92

NA	0	1	2	0	311	2.65
NA	0	1	3	1	239	1.37
NA	0	1	2	0	337	1.58
NA	0	1	2	0	297	1.04
NA	0	1	2	0	214	1.31
NA	0	1	2	0	254	2.64
NA	0	1	2	0	237	1.71
NA	0	1	2	0	216	1.47
NA	0	1	2	0	200	2.06
NA	0	1	2	0	335	1.97
NA	0	1	2	0	207	1.25
NA	0	1	2	0	202	2.18
NA	0	1	2	1	465	2.53
NA	0	1	2	0	359	2.09
NA	0	1	2	0	330	2.46
NA	0	1	2	0	225	1.51
NA	0	1	2	0	350	2.60
NA	0	1	3	1	322	1.39
NA	0	1	3	0	239	1.12
NA	0	1	2	0	353	1.79
NA	0	1	2	0	501	2.87
NA	0	1	2	0	268	2.57
NA	0	1	2	0	307	1.65
NA	0	1	1	0	405	1.12
NA	0	1	2	0	211	0.4
NA	0	1	2	0	239	1.46
NA	0	1	2	1	381	1.47
NA	0	1	2	0	301	1.27
NA	0	1	2	0	173	1.23
NA	0	1	2	0	255	1.78
NA	0	1	2	0	408	1.27
NA	0	1	2	0	148	1.02
NA	0	1	2	1	334	1.88
NA	0	1	2	0	260	2.07
NA	0	1	2	0	241	1.27
NA	0	1	2	0	242	1.81
NA	0	1	2	0	163	1.92
NA	0	1	2	0	416	2.24
NA	0	1	2	0	274	2.74
NA	0	1	2	0	332	1.16
NA	0	1	2	0	336	2.64
NA	0	1	2	0	86	2.23
NA	0	1	2	0	349	1.71
NA	0	1	3	0	269	1.79
NA	0	1	3	1	443	2.20
NA	0	1	2	0	242	2.44

NA	0	1	2	0	346	3.04
NA	0	1	2	1	323	1.54
NA	0	1	2	0	182	1.51
NA	0	1	2	0	382	1.61
NA	0	1	2	0	273	1.23
NA	0	1	3	1	258	1.98
NA	0	1	2	0	239	1.07
NA	0	1	2	0	355	2.15
NA	0	1	2	0	278	2.85
NA	0	1	2	0	257	1.61
NA	0	1	2	1	301	1.84
NA	0	1	2	1	339	0.75
NA	0	1	2	0	303	1.17
NA	0	1	2	0	239	2.01
NA	0	1	2	0	336	1.68
NA	0	1	2	0	237	1.89
NA	0	1	2	0	431	1.48
NA	0	1	2	0	399	1.85
NA	0	1	1	0	250	1.97
NA	0	1	3	0	305	1.15
NA	0	1	3	1	320	1.73
NA	0	1	3	0	294	1.22
NA	0	1	2	0	231	2.03
NA	0	1	2	0	236	1.05
NA	0	1	2	0	360	0.89
NA	0	1	3	0	155	0.78
NA	0	1	2	0	217	2.53
NA	0	1	2	0	227	2.3
NA	0	1	2	0	216	1.6
NA	0	1	2	0	210	1.61
NA	0	1	2	1	279	1.91
NA	0	1	2	1	261	1.18
NA	0	1	2	0	306	2.01
NA	0	1	2	0	228	1.8
NA	0	1	3	1	318	1.87
NA	0	1	2	0	156	1.26
NA	0	1	2	0	136	1.03
NA	0	1	3	0	139	1.41
NA	0	1	2	0	379	1.65
NA	0	1	2	0	203	1.09
NA	0	1	2	0	282	2.35
NA	0	1	3	0	367	2.23
NA	0	1	2	0	230	2.12
NA	0	1	2	0	248	1.05
NA	0	1	2	1	277	2.41
NA	0	1	2	0	195	0.84
NA	0	1	2	0	294	1.28
NA	0	1	3	0	230	2.19

NA	0	1	2	0	245	1.55
NA	0	1	3	0	380	1.52
NA	0	1	2	1	245	1.6
NA	0	1	1	0	257	1.99
NA	0	1	2	0	201	1.6
NA	0	1	3	1	319	1.65
NA	0	1	2	0	315	2.72
NA	0	1	3	0	307	2
NA	0	1	2	0	320	1.14
NA	0	1	2	0	287	1.39
NA	0	1	3	0	319	1.98
NA	0	1	3	1	370	1.77
NA	0	1	2	0	337	1.54
NA	0	1	2	0	282	2.42
NA	0	1	2	0	217	1.83
NA	0	1	2	0	425	2.2
NA	0	1	2	1	186	1.65
NA	0	1	2	0	247	1.83
NA	0	1	2	0	371	1.98
NA	0	1	1	0	264	1.99
NA	0	1	2	0	336	1.19
NA	0	1	2	0	374	1.42
NA	0	1	1	0	320	2.62
NA	0	1	2	0	214	1.75
NA	0	1	2	0	187	1.12
NA	0	1	2	0	280	1.84
NA	0	1	2	0	442	1.42
NA	0	1	2	0	272	1.95
NA	0	1	2	0	255	1.88
NA	0	1	2	0	207	1.07
NA	0	1	2	0	183	1.85
NA	0	1	2	0	286	2.64
NA	0	1	2	0	142	1.62
NA	0	1	2	1	266	1.76
NA	0	1	2	0	173	1.37
NA	0	1	3	0	275	2.84
NA	0	1	2	0	185	2.06
NA	0	1	2	0	360	1.39
NA	0	1	2	0	487	1.9
NA	0	1	2	0	286	1.7
NA	0	1	3	1	176	1.3
NA	0	1	3	1	301	1.6
NA	0	1	2	0	197	1.29
NA	0	1	2	0	281	1.37
NA	0	1	2	0	283	2.46
NA	0	1	2	1	238	2.21
NA	0	1	2	0	542	2.65
NA	0	1	3	0	190	2.08

NA	0	1	2	1	272	2
NA	0	1	2	0	263	1.29
NA	0	1	3	1	240	1.39
NA	0	1	2	0	299	2.3
NA	0	1	2	0	308	1.33
NA	0	1	2	0	231	1.5
NA	0	1	3	0	242	1.46
NA	0	1	2	0	281	2.44
NA	0	1	2	0	326	1.77
NA	0	1	2	0	203	2.8
NA	0	1	2	0	323	1.31
NA	0	1	2	0	392	1.94
NA	0	1	2	0	280	1.31
NA	0	1	2	0	268	2.08
NA	0	1	2	0	338	1.92
NA	0	1	2	1	370	3.11
NA	0	1	2	0	278	1.91
NA	0	1	2	1	247	1.92
NA	0	1	2	1	243	1.37
NA	0	1	2	0	240	2.11
NA	0	1	2	1	413	1.38
NA	0	1	3	1	338	2.38
NA	0	1	3	1	284	1.88
NA	0	1	2	0	221	1.36
NA	0	1	2	0	506	1.57
NA	0	1	2	1	236	2.42
NA	0	1	2	0	329	1.56
NA	0	1	3	0	282	2.11
NA	0	1	2	1	255	2.23
NA	0	1	2	0	342	2.49
NA	0	1	3	1	253	0.85
NA	0	1	2	0	261	1.99
NA	0	1	2	1	370	1.81
NA	0	1	2	0	255	2.87
NA	0	1	2	0	257	1.62
NA	0	1	2	0	209	2.32
NA	0	1	2	0	330	1.7
NA	0	1	2	0	249	2.56
NA	0	1	2	0	236	1.04
NA	0	1	2	0	263	1.53
NA	0	1	2	0	287	3.32
NA	0	1	2	0	405	1.47
NA	0	1	2	1	346	2.74
NA	0	1	2	0	175	1.72
NA	0	1	3	1	302	1.31
NA	0	1	2	0	238	1.76
NA	0	1	1	0	255	1.59
NA	0	1	2	0	361	1.19

NA	0	1	2	0	354	1.94
NA	0	1	2	0	335	1.65
NA	0	1	3	1	334	1.39
NA	0	1	3	1	273	1.96
NA	0	1	2	0	263	2.06
NA	0	1	2	0	463	2.39
NA	0	1	2	0	269	1.54
NA	0	1	3	0	241	1.02
NA	0	1	2	0	222	1.73
NA	0	1	2	0	241	1.41
NA	0	1	2	0	220	1.17
NA	0	1	2	0	302	1.53
NA	0	1	2	0	371	1.83
NA	0	1	2	0	226	2.09
NA	0	1	2	0	380	1.39
NA	0	1	2	0	341	1.16
NA	0	1	2	0	128	1.35
NA	0	1	3	1	314	0.62
NA	0	1	2	0	308	2.1
NA	0	1	2	1	320	2.3
NA	0	1	2	0	270	1.88
NA	0	1	3	1	155	1.27
NA	0	1	2	0	358	2.54
NA	0	1	2	0	235	1.62
NA	0	1	2	0	264	1.55
NA	0	1	2	0	292	1.35
NA	0	1	2	0	243	1.69
NA	0	1	3	1	538	2.29
NA	0	1	2	0	280	2.15
NA	0	1	3	1	210	2.28
NA	0	1	3	0	211	1.58
NA	0	1	2	1	392	1.96
NA	0	1	2	0	443	1.94
NA	0	1	3	0	381	1.63
NA	0	1	2	1	283	1.22
NA	0	1	2	0	342	1.3
NA	0	1	3	0	441	1.93
NA	0	1	2	0	264	2.09
NA	0	1	2	0	299	1.41
NA	0	1	2	0	293	1.85
NA	0	1	3	0	350	1.51
NA	0	1	3	0	287	2.21
NA	0	1	3	0	483	1.55
NA	0	1	2	0	244	1.79
NA	0	1	3	0	217	1.16
NA	0	1	2	0	343	2.82
NA	0	1	3	1	194	1.37
NA	0	1	1	1	236	2.05

NA	0	1	2	0	348	2.28
NA	0	1	2	0	129	0.57
NA	0	1	2	0	237	1.43
NA	0	1	3	0	262	2.28
NA	0	1	2	0	276	1.63
NA	0	1	2	0	228	2.1
NA	0	1	2	1	197	1.57
NA	0	1	3	0	438	1.5
NA	0	1	2	0	241	2.1
NA	0	1	2	1	207	1.75
NA	0	1	2	0	266	1.44
NA	0	1	2	0	191	0.54
NA	0	1	3	0	294	1.96
NA	0	1	2	0	427	1.22
NA	0	1	2	1	246	2.92
NA	0	1	2	0	131	2.41
NA	0	1	3	1	454	1.46
NA	0	1	2	0	165	1.32
NA	0	1	2	0	233	1.99
NA	0	1	2	0	312	1.48
NA	0	1	2	0	192	1.37
NA	0	1	2	0	270	1.41
NA	0	1	2	1	321	2.93
NA	0	1	2	0	305	1.68
NA	0	1	2	0	277	2
NA	0	1	3	1	258	2.46
NA	0	1	2	0	271	1.22
NA	0	1	2	0	257	1.34
NA	0	1	3	0	311	1.53
NA	0	1	2	0	255	1.66
NA	0	1	2	0	184	0.88
NA	0	1	2	0	221	1.73
NA	0	1	2	1	243	1.5
NA	0	1	2	0	259	2.78
NA	0	1	2	0	271	1.14
NA	0	1	2	0	253	1.94
NA	0	1	2	0	223	2.61
NA	0	1	2	0	299	1.92
NA	0	1	2	1	283	1.65
NA	0	1	2	1	276	2.32
NA	0	1	2	0	354	1.4
NA	0	1	2	1	321	1.97
NA	0	1	3	0	210	1.32
NA	0	1	2	0	307	0.77
NA	0	1	2	0	391	1.46
NA	0	1	3	1	295	1.54
NA	0	1	2	0	536	1.74
NA	0	1	2	0	264	2.21

NA	0	1	2	0	257	2.04
NA	0	1	2	0	192	3.06
NA	0	1	3	1	261	1.33
NA	0	1	2	1	283	2.3
NA	0	1	2	0	214	1.85
NA	0	1	3	1	343	1.39
NA	0	1	2	0	246	1.53
NA	0	1	2	0	321	1.01
NA	0	1	3	0	268	2.62
NA	0	1	2	0	295	1.58
NA	0	1	3	1	323	2.44
NA	0	1	2	0	228	1.39
NA	0	1	3	0	248	1.42
NA	0	1	2	0	502	1.98
NA	0	1	2	0	248	1.57
NA	0	1	2	0	246	2
NA	0	1	2	0	218	1.74
NA	0	1	2	0	251	1.8
NA	0	1	3	0	233	1.84
NA	0	1	3	1	401	1.51
NA	0	1	3	0	321	1.8
NA	0	1	2	1	280	1.71
NA	0	1	2	0	252	1.12
NA	0	1	2	1	229	0.99
NA	0	1	2	0	258	1.53
NA	0	1	3	0	329	1.82
NA	0	1	2	0	167	1.16
NA	0	1	2	0	258	1.08
NA	0	1	2	0	202	1.94
NA	0	1	3	1	389	2.48
NA	0	1	2	0	270	1.61
NA	0	1	2	0	293	0.9
NA	0	1	3	0	237	1.26
NA	0	1	2	0	284	1.71
NA	0	1	2	0	147	1.54
NA	0	1	2	0	329	1.68
NA	0	1	2	0	357	1.85
NA	0	1	2	0	295	1.5
NA	0	1	2	0	334	2.15
NA	0	1	2	1	314	2.1
NA	0	1	2	0	302	0.93
NA	0	1	2	0	277	3.97
NA	0	1	2	0	372	2.21
NA	0	1	2	0	240	1.94
NA	0	1	2	0	239	1
NA	0	1	2	0	315	0.85
NA	0	1	3	0	207	2.49
NA	0	1	2	0	331	3.05

NA	0	1	2	0	313	1.73
NA	0	1	2	0	230	2.2
NA	0	1	2	0	289	2.02
NA	0	1	1	0	254	1.4
NA	0	1	3	0	264	0.84
NA	0	1	2	1	252	1.77
NA	0	1	2	0	269	1.46
NA	0	1	2	0	213	2.19
NA	0	1	2	0	307	1.53
NA	0	1	2	0	311	1.2
NA	0	1	2	1	338	2.29
NA	0	1	2	0	212	0.9
NA	0	1	3	1	281	1.32
NA	0	1	2	1	203	1.96
NA	0	1	2	0	228	1.87
NA	0	1	2	0	307	2.52
NA	0	1	2	0	345	1.91
NA	0	1	2	1	232	1.82
NA	0	1	3	0	337	2.33
NA	0	1	2	0	439	1.85
NA	0	1	2	0	354	1.62
NA	0	1	2	1	264	1.7
NA	0	1	2	1	394	2.85
NA	0	1	3	0	465	1.68
NA	0	1	2	0	160	2.23
NA	0	1	2	0	189	1.53
NA	0	1	2	1	269	1.83
NA	0	1	2	1	275	1.48
NA	0	1	2	1	310	2.03
NA	0	1	2	0	355	1.56
NA	0	1	2	0	306	1.62
NA	0	1	2	1	451	1.29
NA	0	1	2	0	219	1.95
NA	0	1	3	1	286	1.54
NA	0	1	3	1	350	2.49
NA	0	1	2	1	252	0.99
NA	0	1	2	0	263	1.22
NA	0	1	2	0	159	1.51
NA	0	1	2	0	322	2.62
NA	0	1	2	0	360	2.07
NA	0	1	3	0	376	1.25
NA	0	1	2	0	134	1.46
NA	0	1	2	0	362	2.02
NA	0	1	2	0	424	1.48
NA	0	1	2	0	310	1.49
NA	0	1	2	1	300	2.8
NA	0	1	2	0	287	2.21
NA	0	1	2	1	323	2.7

NA	0	1	3	0	362	1.61
NA	0	1	2	1	210	2.16
NA	0	1	3	1	342	1.59
NA	0	1	3	1	307	1.57
NA	0	1	3	1	295	2.49
NA	0	1	3	0	454	1.57
NA	0	1	1	0	240	2.05
NA	0	1	2	0	234	0.96
NA	0	1	2	0	375	1.54
NA	0	1	2	0	360	1.83
NA	0	1	2	0	253	1.24
NA	0	1	2	0	314	1.81
NA	0	1	2	0	273	2.17
NA	0	1	2	0	348	1.65
NA	0	1	2	1	247	1.73
NA	0	1	3	0	386	1.72
NA	0	1	2	0	248	2.15
NA	0	1	2	0	330	2.2
NA	0	1	2	0	345	1.84
NA	0	1	2	0	210	1.18
NA	0	1	2	0	228	1.25
NA	0	1	2	1	423	1.44
NA	0	1	2	0	246	2.11
NA	0	1	2	0	280	1.78
NA	0	1	1	1	386	1.92
NA	0	1	2	1	337	3.13
NA	0	1	2	0	486	2.45
NA	0	1	2	0	332	1.09
NA	0	1	2	0	307	1.7
NA	0	1	2	0	281	3.4
NA	0	1	2	1	183	2.06
NA	0	1	2	0	207	2.07
NA	0	1	2	0	284	2.54
NA	0	1	2	0	127	2.24
NA	0	1	2	0	300	2.3
NA	0	1	2	0	297	2.67
NA	0	1	2	0	283	2.24
NA	0	1	2	0	235	1.68
NA	0	1	2	0	215	1.49
NA	0	1	3	0	453	2.21
NA	0	1	2	1	248	0.91
NA	0	1	2	0	430	2.08
NA	0	1	2	0	111	1.25
NA	0	1	2	1	246	2.02
NA	0	1	2	0	321	3.29
NA	0	1	2	0	194	1.13
NA	0	1	2	0	310	1.46
NA	0	1	2	1	298	0.53

NA	0	1	2	1	269	1.92
NA	0	1	2	0	88	1.27
NA	0	1	2	1	361	1.43
NA	0	1	2	0	280	1.29
NA	0	1	2	0	383	1.86
NA	0	1	3	1	419	2.43
NA	0	1	2	0	362	2.24
NA	0	1	2	0	286	2.22
NA	0	1	2	0	281	3.41
NA	0	1	1	0	351	1.59
NA	0	1	2	0	369	1.59
NA	0	1	3	0	265	2.37
NA	0	1	2	0	230	1.67
NA	0	1	2	0	314	1.64
NA	0	1	2	0	416	2.39
NA	0	1	2	0	325	1.49
NA	0	1	2	0	290	1.79
NA	0	1	2	1	160	1.78
NA	0	1	2	0	213	2.69
NA	0	1	2	1	202	3.27
NA	0	1	2	0	288	1.57
NA	0	1	2	0	231	0.71
NA	0	1	3	1	281	1.68
NA	0	1	2	0	497	1.56
NA	0	1	3	1	276	1.37
NA	0	1	2	1	331	1.62
NA	0	1	2	0	404	3.58
NA	0	1	3	0	361	2.1
NA	0	1	2	1	133	1.37
NA	0	1	2	1	207	1.28
NA	0	1	3	1	362	1.7
NA	0	1	2	0	261	1.91
NA	0	1	2	0	328	2.45
NA	0	1	3	1	218	1.66
NA	0	1	2	0	350	2.19
NA	0	1	3	0	227	1.98
NA	0	1	2	0	351	3.2
NA	0	1	3	0	371	1.41
NA	0	1	2	0	382	1.86
NA	0	1	2	0	352	2.74
NA	0	1	3	1	417	2.47
NA	0	1	2	0	344	2.16
NA	0	1	2	0	374	2.2
NA	0	1	3	1	359	1.07
NA	0	1	2	0	238	2.33
NA	0	1	2	0	302	1.52
NA	0	1	2	0	328	2.76
NA	0	1	2	0	309	2.42

NA	0	1	2	0	381	2.07
NA	0	1	2	0	287	3.05
NA	0	1	3	1	377	2.72
NA	0	1	2	0	313	2.43
NA	0	1	3	0	349	2.76
NA	0	1	3	0	445	2.82
NA	0	1	3	1	257	1.38

Neutrophil	Monocyte	PLR	NLR	MLR	SII	TG
3.83	0.3	168.55	3.09	0.24	645.54	0.87
4.44	0.37	142.47	1.71	0.14	632.57	1.08
6.36	0.34	185.43	4.21	0.23	1179.34	0.91
1.9	0.17	150.70	1.34	0.12	286.34	1.25
3.37	0.31	170.19	2.09	0.19	573.53	0.87
4.72	0.58	164.35	2.19	0.27	775.74	1
3.46	0.29	160.59	2.04	0.17	555.64	1.16
2.83	0.24	187.76	1.93	0.16	531.35	1.24
4.45	0.94	100.00	1.53	0.32	445.00	1.38
3.86	0.38	280.62	2.99	0.29	1083.19	0.41
3.46	0.3	125.87	1.72	0.15	435.51	0.88
4.77	0.47	153.37	2.68	0.26	731.58	0.98
2.98	0.27	114.43	1.54	0.14	341.01	0.98
2.63	0.41	229.79	1.40	0.22	604.34	0.62
3.55	0.52	100.50	1.78	0.26	356.78	0.72
2.42	0.26	153.51	2.12	0.23	371.49	0.72
4.31	0.34	160.43	1.83	0.14	691.43	1.94
2.43	0.34	140.09	1.12	0.16	340.42	0.7
3.33	0.45	141.35	1.60	0.22	470.68	1.87
1.94	0.5	195.97	1.30	0.34	380.19	0.98
2.03	0.34	135.03	1.15	0.19	274.11	0.48
1.97	0.26	142.13	1.11	0.15	280.01	0.59
5.38	0.3	241.30	5.85	0.33	1298.22	0.62
2.85	0.37	147.27	1.73	0.22	419.73	0.63
3.1	0.33	145.93	1.48	0.16	452.39	0.64
3.3	0.4	107.91	1.53	0.19	356.09	0.7
3.66	0.24	107.62	1.74	0.11	393.89	0.73
2.95	0.42	79.73	1.00	0.14	235.20	0.76
3.95	0.6	113.25	1.69	0.26	447.33	0.78
3.17	0.21	90.59	1.86	0.12	287.16	0.81
2.57	0.73	274.62	1.98	0.56	705.76	0.84
3.09	0.34	155.80	1.71	0.19	481.43	0.85
3.83	0.53	158.87	1.54	0.21	608.48	0.88
2.75	0.38	77.63	1.26	0.17	213.47	0.91
2.15	0.32	103.65	0.98	0.15	222.85	0.91
4.84	0.45	110.89	1.88	0.18	536.73	0.95
3.11	0.35	154.45	1.63	0.18	480.34	1.01
2.73	0.4	188.10	2.17	0.32	513.50	1.02
2.95	0.42	200.65	1.90	0.27	591.90	1.06
2.23	0.42	186.67	1.49	0.28	416.27	1.07
3.15	0.39	167.91	1.68	0.21	528.93	1.18
3.3	0.25	166.21	1.51	0.11	548.49	1.39
2.87	0.23	173.68	1.51	0.12	498.47	1.47
1.88	0.20	121.48	1.39	0.15	228.39	1.52
2.99	0.46	121.74	1.44	0.22	364.00	1.64
3.68	0.43	57.45	0.88	0.10	211.42	1.8
4.29	0.35	192.68	2.62	0.21	826.61	2.06

2.83	0.56	99.58	1.20	0.24	281.80	2.15
3.01	0.37	180.88	2.21	0.27	544.46	0.51
5.23	0.42	194.92	2.65	0.21	1019.45	0.61
1.57	0.46	74.28	0.50	0.15	116.61	0.64
3.52	0.29	226.19	2.79	0.23	796.19	0.78
3.4	0.4	167.18	2.60	0.31	568.40	0.80
3.21	0.25	105.26	1.54	0.12	337.89	0.81
3.98	0.24	190.27	2.15	0.13	757.28	0.81
3.6	0.3	284.21	2.71	0.23	1023.16	0.84
1.71	0.25	207.44	1.41	0.21	354.72	1.08
3.7	0.23	174.80	2.91	0.18	646.77	1.09
1.92	0.33	177.40	1.32	0.23	340.60	1.21
5.62	0.5	346.38	8.14	0.72	1946.64	1.31
4.07	1.04	93.35	1.29	0.33	379.95	1.5
2.88	0.44	105.24	1.37	0.21	303.09	1.6
1.36	0.26	166.15	1.05	0.20	225.97	0.71
3.33	0.44	299.12	2.95	0.39	996.05	0.65
3.34	0.3	154.00	1.67	0.15	514.36	0.75
2.67	0.19	104.95	1.20	0.09	280.23	0.77
2.42	0.22	212.07	2.09	0.19	513.21	0.81
2.98	0.4	166.91	2.14	0.29	497.38	0.82
2.99	0.29	255.88	2.20	0.21	765.09	0.86
2.56	0.4	149.61	1.98	0.31	383.01	0.88
2.18	0.42	133.95	1.35	0.26	292.01	0.97
4.68	0.44	170.97	3.02	0.28	800.13	0.97
3.76	0.38	155.13	2.41	0.24	583.28	1.01
7.44	0.33	229.79	7.91	0.35	1709.62	1.03
4.26	0.32	140.28	1.97	0.15	597.58	1.13
3.36	0.42	92.13	1.32	0.17	309.54	1.16
7.27	0.75	109.74	4.72	0.49	797.81	1.26
2.39	0.3	161.70	1.27	0.16	386.47	1.29
4.57	0.66	113.24	2.24	0.32	517.49	1.55
3.55	0.69	86.49	1.92	0.37	307.03	1.74
5.39	0.47	270.37	4.99	0.44	1457.30	1.74
3.33	0.35	200.00	1.75	0.18	666.00	1.76
5.01	0.81	209.36	2.47	0.40	1048.89	1.81
8.2	1.05	110.77	4.21	0.54	908.31	2.75
10.03	0.25	108.57	3.58	0.09	1088.97	2.8
3.8	0.58	89.59	1.41	0.22	340.45	3.2
8.03	0.26	90.43	8.54	0.28	726.12	3.59
8.14	0.53	108.33	2.95	0.19	881.83	5.03
2.27	0.28	155.33	1.15	0.14	352.60	0.58
2.45	0.17	183.33	1.86	0.13	449.17	0.59
3.73	0.21	91.72	2.57	0.14	342.13	0.71
2.34	0.51	92.28	0.86	0.19	215.93	0.77
3.32	0.41	101.35	1.50	0.18	336.49	1.02
3.04	0.27	201.64	2.49	0.22	612.98	1.11
4.21	0.29	88.10	1.43	0.10	370.88	1.62

1.43	0.59	123.64	0.65	0.27	176.80	3.11
1.93	0.39	238.74	1.74	0.35	460.77	0.53
2.87	0.33	107.69	2.01	0.23	309.08	1.05
4.04	0.41	105.64	2.07	0.21	426.79	0.68
3.39	0.24	203.57	1.73	0.12	690.11	0.53
2.99	0.19	160.00	2.21	0.14	478.40	0.33
1.95	0.33	133.33	0.94	0.16	260.00	0.49
3.2	0.48	102.40	1.92	0.29	327.66	0.49
2.69	0.51	127.59	1.16	0.22	343.21	0.5
2.53	0.58	102.43	1.23	0.28	259.14	0.54
2.36	0.56	129.88	1.44	0.34	306.51	0.54
4.43	0.36	181.25	3.08	0.25	802.94	0.57
4.4	0.51	112.89	1.96	0.23	496.71	0.58
3.26	0.31	164.29	2.33	0.22	535.57	0.58
3.22	0.47	266.46	1.96	0.29	858.01	0.6
2.26	0.33	129.27	1.10	0.16	292.15	0.62
4.97	0.28	88.20	2.79	0.16	438.37	0.64
3.12	0.21	166.91	2.29	0.15	520.76	0.64
2.19	0.62	177.66	1.11	0.31	389.09	0.65
2.94	0.38	136.49	1.39	0.18	401.29	0.67
3.03	0.31	114.83	1.28	0.13	347.94	0.7
4.16	0.32	183.12	2.70	0.21	761.77	0.72
1.91	0.4	138.07	0.88	0.18	263.72	0.74
4.46	0.37	93.10	2.20	0.18	415.24	0.75
5.41	0.71	96.75	1.95	0.26	523.42	0.79
1.84	0.46	57.03	0.72	0.18	104.94	0.82
4.33	0.54	106.72	1.71	0.21	462.09	0.84
2.73	0.28	128.13	1.42	0.15	349.78	0.87
2.79	0.27	204.14	1.92	0.19	569.54	0.87
2.24	0.49	157.24	1.54	0.34	352.22	0.88
2.58	0.47	126.90	1.31	0.24	327.41	0.89
3.33	0.44	257.54	1.86	0.25	857.61	0.99
2.28	0.2	220.47	1.80	0.16	502.68	1.02
3.44	0.41	119.07	1.60	0.19	409.60	1.03
5.21	0.61	137.77	2.24	0.26	717.77	1.03
3.51	0.39	100.34	1.19	0.13	352.19	1.05
2.81	0.42	126.00	1.12	0.17	354.06	1.09
3.2	0.5	198.82	1.88	0.29	636.24	1.14
4.16	0.25	126.79	2.48	0.15	527.43	1.18
4.73	0.31	240.00	3.64	0.24	1135.20	1.24
3.57	0.53	184.72	2.48	0.37	659.46	1.31
1.82	0.37	170.42	0.85	0.17	310.17	1.32
2.83	0.37	96.97	1.43	0.19	274.42	1.44
3.17	0.69	113.65	1.27	0.28	360.29	2.11
4.77	0.22	183.59	3.73	0.17	875.74	2.11
9.36	0.61	180.23	5.44	0.35	1686.98	2.54
4.53	0.55	229.52	2.73	0.33	1039.72	2.84
4.84	0.8	116.15	1.86	0.31	562.18	2.87

6.89	0.45	152.85	2.62	0.17	1053.15	3.01
8.95	0.5	126.50	7.65	0.43	1132.14	3.83
2.22	0.21	140.63	1.39	0.13	312.19	0.49
6.56	0.38	190.96	3.71	0.21	1252.70	0.51
4.43	0.47	144.49	1.95	0.21	640.11	0.64
1.78	0.3	181.94	2.47	0.42	323.86	0.65
3.78	0.32	155.77	1.82	0.15	588.81	0.66
2.86	0.54	245.11	2.15	0.41	701.02	0.66
2.32	0.33	217.27	2.11	0.30	504.07	0.67
2.97	0.27	199.31	2.06	0.19	591.94	0.78
1.42	0.23	128.79	1.08	0.17	182.88	0.8
4.77	0.23	63.49	2.52	0.12	302.86	0.84
3.18	0.57	275.93	2.94	0.53	877.44	0.88
1.65	0.28	116.25	1.03	0.18	191.81	0.9
3.41	0.57	87.04	1.05	0.18	296.80	0.9
3.48	0.39	215.88	2.05	0.23	751.27	0.92
2.94	0.29	224.43	2.24	0.22	659.82	0.97
2.83	0.3	263.97	2.08	0.22	747.04	1
2.62	0.47	183.75	1.64	0.29	481.43	1.05
3.75	0.24	220.90	2.12	0.14	828.39	1.81
2.51	0.54	95.77	0.97	0.21	240.38	0.67
2.3	0.36	185.45	2.09	0.33	426.55	0.4
2.49	0.35	123.46	1.54	0.22	307.41	0.59
3.69	0.42	203.61	2.22	0.25	751.34	0.65
2.27	0.19	126.34	1.22	0.10	286.80	0.66
4.85	0.37	175.19	3.76	0.29	849.69	0.67
2.25	0.26	181.03	1.29	0.15	407.33	0.69
2.27	0.18	164.03	1.63	0.13	372.35	0.73
2.4	0.27	222.73	1.56	0.18	534.55	0.75
2.61	0.38	130.77	1.34	0.19	341.31	0.78
3.01	0.43	201.77	2.66	0.38	607.33	0.79
3.81	0.35	93.92	1.16	0.11	357.84	0.9
3.63	0.3	154.55	3.00	0.25	561.00	1.06
4.94	0.49	111.27	2.32	0.23	549.66	1.1
3.5	0.59	125.41	1.89	0.32	438.92	1.3
1.89	0.22	86.87	0.95	0.11	164.18	1.39
4.17	0.39	102.19	1.52	0.14	426.13	1.46
4.42	0.44	318.56	4.56	0.45	1408.02	1.57
4.27	0.28	117.33	2.11	0.14	500.99	1.6
4.81	0.45	167.68	1.83	0.17	806.54	2.74
8.39	0.47	185.80	5.18	0.29	1558.88	5.24
2.17	0.32	93.88	1.11	0.16	203.71	0.46
2.52	0.23	224.32	2.27	0.21	565.30	0.54
2.58	0.37	104.35	1.25	0.18	269.22	0.58
2.4	0.25	134.78	1.30	0.14	323.48	0.69
2.56	0.24	150.65	1.66	0.16	385.66	0.71
3.3	0.31	164.03	2.37	0.22	541.29	0.75
2.67	0.36	159.88	1.55	0.21	426.89	1.08

2.89	0.25	157.39	1.64	0.14	454.85	1.16
6.46	0.9	155.35	4.06	0.57	1003.53	2.53
1.66	0.25	101.96	0.65	0.10	169.25	0.48
1.91	0.27	155.69	1.14	0.16	297.37	0.45
3.94	0.47	243.85	3.03	0.36	960.75	0.66
4.2	0.45	143.82	2.36	0.25	604.04	0.43
3.33	0.34	142.55	1.77	0.18	474.70	0.44
2.89	0.26	201.69	2.45	0.22	582.90	0.49
2.1	0.32	191.56	1.36	0.21	402.27	0.52
2.44	0.42	207.73	1.35	0.23	506.87	0.52
4.06	0.51	96.14	1.74	0.22	390.32	0.55
4.56	0.34	136.15	3.51	0.26	620.86	0.63
1.76	0.25	164.93	0.83	0.12	290.27	0.67
7.19	0.59	160.59	4.23	0.35	1154.63	0.67
2.44	0.3	163.16	1.43	0.18	398.11	0.72
3.61	0.2	297.00	3.61	0.20	1072.17	0.72
3.89	0.5	98.52	1.15	0.15	383.23	0.81
4.59	0.44	228.04	2.43	0.23	1046.71	0.83
3.44	0.27	75.65	1.50	0.12	260.24	0.85
5.97	0.35	187.77	4.29	0.25	1120.99	0.85
2.09	0.29	74.17	0.87	0.12	155.01	0.86
1.96	0.23	268.00	1.96	0.23	525.28	0.87
2.78	0.27	132.12	1.68	0.16	367.30	0.91
3.01	0.57	180.27	2.05	0.39	542.62	0.91
3.09	0.36	208.57	2.94	0.34	644.49	0.92
2.83	0.25	221.62	2.55	0.23	627.19	1.01
3.84	0.27	153.08	1.82	0.13	587.83	1.02
3.6	0.4	80.71	1.16	0.13	290.55	1.07
1.43	0.26	217.19	1.12	0.20	310.58	1.09
3	0.15	140.37	1.86	0.09	421.12	1.13
4.14	0.26	206.94	2.88	0.18	856.75	1.13
2.78	0.47	197.29	1.26	0.21	548.45	1.14
4.52	0.37	125.82	2.12	0.17	568.71	1.14
2.05	0.36	188.69	1.22	0.21	386.82	1.17
2.91	0.35	143.90	1.42	0.17	418.76	1.19
5.7	0.52	128.03	1.82	0.17	729.75	1.39
4.01	0.4	434.12	4.72	0.47	1740.81	1.4
2.67	0.29	237.50	1.76	0.19	634.13	1.53
4.5	0.46	111.41	1.71	0.17	501.33	1.54
2.9	0.36	81.78	1.29	0.16	237.16	1.59
3	0.54	86.54	0.92	0.17	259.63	1.61
3.25	0.48	75.85	0.79	0.12	246.50	1.62
8.38	0.21	291.58	8.82	0.22	2443.43	1.86
3.95	0.35	187.82	1.66	0.15	741.87	1.97
8.69	0.51	181.19	8.60	0.50	1574.52	2.03
4.3	0.43	166.27	1.71	0.17	714.96	2.59
8.55	0.83	286.61	6.73	0.65	2450.55	2.93
4.61	0.63	90.82	1.57	0.21	418.66	3.03

6.59	0.4	168.48	3.58	0.22	1110.27	3.14
6.09	0.35	171.21	2.37	0.14	1042.65	3.7
3.53	0.63	74.52	1.36	0.24	263.05	0.44
2.77	0.43	115.34	1.57	0.24	319.49	0.46
3.1	0.28	177.30	2.20	0.20	549.65	0.49
1.99	0.46	188.72	1.02	0.24	375.55	0.53
2.63	0.41	256.69	2.07	0.32	675.10	0.56
2.22	0.42	92.69	0.85	0.16	205.78	0.68
3.34	0.42	97.51	1.19	0.15	325.68	0.69
4.43	0.23	135.21	2.08	0.11	598.99	0.69
3.83	0.24	147.18	2.70	0.17	563.71	0.74
3.88	0.27	109.55	2.18	0.15	425.06	0.84
1.58	0.46	206.31	1.42	0.41	325.96	0.91
2.24	0.28	222.46	1.62	0.20	498.32	0.96
4.37	0.31	171.10	2.53	0.18	747.70	0.97
2.2	0.47	183.98	1.07	0.23	404.76	0.99
4.05	0.31	121.71	2.31	0.18	492.94	1.09
1.81	0.39	117.14	1.03	0.22	212.03	1.12
4.47	0.44	86.08	1.41	0.14	384.76	1.12
2.46	0.38	124.84	1.57	0.24	307.11	1.16
4.59	0.64	121.67	1.75	0.24	558.48	1.19
2.9	0.31	205.29	1.71	0.18	595.35	1.39
5.21	0.38	288.68	9.83	0.72	1504.02	1.47
2.84	0.48	123.89	1.26	0.21	351.86	1.5
4.27	0.72	122.61	1.24	0.21	523.54	1.71
4.27	0.42	157.92	2.33	0.23	674.33	1.72
3.64	0.53	160.96	2.49	0.36	585.89	1.84
2.93	0.32	151.72	1.44	0.16	444.55	2.08
4.63	0.37	127.07	2.56	0.20	588.34	2.18
2.99	0.43	322.46	2.17	0.31	964.17	0.46
3.08	0.3	128.77	1.45	0.14	396.62	1.05
2.39	0.49	104.76	0.81	0.17	250.38	0.93
3.86	0.26	255.46	3.24	0.22	986.08	0.42
3.02	0.41	231.19	2.77	0.38	698.20	0.58
1.78	0.23	141.40	0.96	0.12	251.69	0.66
3.3	0.47	60.67	0.85	0.12	200.21	0.69
2.08	0.39	140.16	0.82	0.15	291.53	0.73
5.62	0.34	169.15	5.98	0.36	950.62	1.13
3.95	0.43	118.65	1.27	0.14	468.67	1.19
3.98	0.38	156.19	1.90	0.18	621.64	1.29
3.86	0.42	119.28	1.73	0.19	460.43	1.35
3.44	0.50	126.92	1.89	0.27	436.62	1.51
1.83	0.43	126.15	0.94	0.22	230.86	1.52
5.24	0.26	116.02	2.90	0.14	607.96	2.67
1.83	0.32	211.89	0.99	0.17	387.76	0.46
2.4	0.43	162.07	1.66	0.30	388.97	0.46
3.34	0.41	105.88	1.96	0.24	353.65	0.86
3.14	0.38	168.18	2.04	0.25	528.09	1.1

3.72	0.24	192.24	1.52	0.10	715.15	1.31
4	0.45	205.69	3.25	0.37	822.76	1.65
5.95	0.45	125.31	2.47	0.19	745.60	2.66
3.88	0.55	111.54	1.66	0.24	432.77	0.49
2.73	0.31	129.75	1.73	0.20	354.21	0.9
4.4	0.53	160.17	1.90	0.23	704.76	1.08
2.49	0.61	155.38	1.34	0.33	386.89	0.66
2.41	0.36	126.45	1.55	0.23	304.75	0.84
4.46	0.59	65.42	1.29	0.17	291.76	0.66
2.07	0.42	115.52	1.78	0.36	239.12	0.44
3.03	0.33	142.35	1.55	0.17	431.31	0.48
1.6	0.3	89.86	1.08	0.20	143.78	0.51
1.92	0.3	105.98	0.76	0.12	203.47	0.52
1.66	0.23	110.53	0.97	0.13	183.47	0.53
4.38	0.55	161.98	1.67	0.21	709.46	0.54
2.29	0.25	117.37	1.21	0.13	268.77	0.56
2.15	0.48	198.51	1.60	0.36	426.79	0.56
3.77	0.62	83.57	1.32	0.22	315.05	0.57
2.7	0.46	121.56	1.62	0.28	328.20	0.59
4.26	0.32	141.11	2.37	0.18	601.13	0.62
3.89	0.28	275.00	3.24	0.23	1069.75	0.64
2.65	0.35	136.11	1.84	0.24	360.69	0.65
1.75	0.43	105.61	0.89	0.22	184.82	0.68
3.13	0.46	124.29	1.49	0.22	389.01	0.69
3.69	0.51	159.38	1.65	0.23	588.09	0.69
1.18	0.32	502.74	1.62	0.44	593.23	0.7
3.88	0.27	91.28	2.26	0.16	354.16	0.74
2.48	0.53	93.88	1.27	0.27	232.82	0.75
2.11	0.38	126.25	1.32	0.24	266.39	0.8
3.7	0.39	227.14	2.64	0.28	840.43	0.8
1.57	0.35	125.49	1.03	0.23	197.02	0.81
2.95	0.21	197.50	1.84	0.13	582.63	0.81
4.44	0.31	81.77	2.19	0.15	363.07	0.82
2.64	0.3	187.50	1.94	0.22	495.00	0.83
3.31	0.55	165.82	1.69	0.28	548.85	0.87
2.49	0.57	108.44	1.05	0.24	270.01	0.91
2.85	0.26	117.14	1.36	0.12	333.86	0.92
2.64	0.24	193.64	1.53	0.14	511.21	0.96
3.09	0.57	212.50	1.68	0.31	656.63	0.97
3.34	0.48	126.46	1.77	0.25	422.36	1.04
3.02	0.32	229.77	2.31	0.24	693.91	1.04
2.9	0.36	143.75	1.81	0.23	416.88	1.06
1.09	0.28	160.54	0.59	0.15	174.99	1.1
4.34	0.34	231.62	3.71	0.29	1005.25	1.12
2.38	0.54	86.84	0.89	0.20	206.68	1.13
3.08	0.33	89.44	1.91	0.20	275.48	1.13
3.62	0.27	133.71	2.03	0.15	484.02	1.16
1.37	0.24	177.56	0.88	0.15	243.26	1.2

3.25	0.28	175.63	1.65	0.14	570.81	1.27
2.32	0.29	48.57	2.21	0.28	112.69	1.29
2.38	0.27	289.78	1.74	0.20	689.68	1.3
3.3	0.39	112.35	1.36	0.16	370.74	1.34
5.06	0.54	114.88	2.35	0.25	581.31	1.61
2.23	0.2	78.49	0.84	0.08	175.03	1.62
6.56	0.64	77.08	2.28	0.22	505.67	1.73
4.82	0.33	206.76	3.26	0.22	996.57	2.16
4.18	0.3	124.63	3.12	0.22	520.94	2.21
10.66	0.76	208.09	7.84	0.56	2218.22	2.81
11.47	0.49	197.50	9.56	0.41	2265.33	4.3
4.53	0.35	141.18	2.42	0.19	639.53	0.37
3.04	0.5	76.68	1.20	0.20	233.11	0.44
2.93	0.45	100.00	1.42	0.22	293.00	0.5
2.07	0.3	171.92	1.02	0.15	355.88	0.5
3.76	0.48	257.25	2.87	0.37	967.27	0.55
2.83	0.31	150.94	1.78	0.19	427.17	0.56
1.5	0.45	528.07	2.63	0.79	792.11	0.56
2.13	0.72	165.96	1.13	0.38	353.49	0.58
2.88	0.52	100.00	1.57	0.28	288.00	0.61
1.97	0.42	275.00	1.82	0.39	541.75	0.63
3.38	0.33	121.59	1.28	0.13	410.98	0.64
1.57	0.4	91.28	0.72	0.18	143.32	0.74
2.91	0.34	191.11	1.62	0.19	556.13	0.8
1.9	0.41	134.36	0.84	0.18	255.29	0.81
2.71	0.34	167.52	1.73	0.22	453.97	0.81
2.45	0.35	157.32	1.56	0.22	385.45	0.84
3.25	0.34	106.76	1.46	0.15	346.96	0.86
3.72	0.20	250.62	2.30	0.12	932.30	0.87
6.54	0.34	698.00	13.08	0.68	4564.92	0.98
5.03	0.29	137.58	3.20	0.18	692.03	1.09
5.83	0.36	259.77	6.70	0.41	1514.46	1.12
3.19	0.25	198.55	2.31	0.18	633.38	1.14
2.82	0.36	137.73	1.28	0.16	388.39	1.22
3.49	0.44	137.41	2.37	0.30	479.58	1.26
5.58	0.43	90.38	2.68	0.21	504.35	1.3
1.48	0.15	306.42	1.36	0.14	453.50	1.32
4.25	0.38	151.53	2.17	0.19	644.01	1.33
3.95	0.32	114.13	1.47	0.12	450.80	1.4
3.08	0.43	150.26	1.63	0.23	462.81	1.53
5.54	0.4	167.69	4.26	0.31	929.02	1.54
3.73	0.44	181.34	2.78	0.33	676.41	1.58
4.48	0.3	246.40	3.58	0.24	1103.87	1.61
3.77	0.42	130.40	1.66	0.19	491.59	1.83
5.54	0.37	213.33	4.62	0.31	1181.87	2.12
5.22	0.63	192.00	4.18	0.50	1002.24	2.34
5.32	0.52	81.14	1.89	0.19	431.66	2.45
4.84	0.35	73.10	1.53	0.11	353.81	2.68

8.49	0.5	122.16	4.59	0.27	1037.16	2.93
5.27	0.46	104.65	2.04	0.18	551.51	3.4
4.37	0.5	146.81	3.10	0.35	641.55	0.5
4.22	0.43	104.33	1.66	0.17	440.28	0.51
2.98	0.52	141.85	1.62	0.28	422.71	0.85
3.73	0.65	110.12	1.51	0.26	410.75	0.77
2.46	0.37	187.72	1.44	0.22	461.79	0.89
2.68	0.31	129.15	1.09	0.13	346.12	0.93
2.41	0.43	119.86	0.87	0.16	288.85	1.84
4.25	0.38	151.67	2.03	0.18	644.62	0.5
1.64	0.23	103.97	1.30	0.18	170.51	0.64
4.06	0.57	152.81	1.76	0.25	620.42	0.8
2.67	0.33	129.28	1.48	0.18	345.18	1
2.04	0.26	152.69	1.10	0.14	311.48	1.03
1.9	0.36	230.20	1.28	0.24	437.38	1.05
3.63	0.22	197.13	2.09	0.13	715.57	1.1
1.66	0.16	111.45	1.00	0.10	185.00	1.19
3.39	0.47	141.99	1.02	0.14	481.36	1.27
7.18	0.56	215.89	4.75	0.37	1550.12	1.44
2.9	0.64	106.54	1.36	0.30	308.97	1.9
8.04	0.6	124.04	3.87	0.29	997.27	2.73
2.92	0.58	118.62	1.55	0.31	346.36	0.38
3.58	0.39	223.45	2.47	0.27	799.94	0.54
3.82	0.46	86.55	1.61	0.19	330.64	0.7
3.07	0.22	160.25	1.91	0.14	491.96	0.96
3.16	0.59	192.56	1.47	0.27	608.48	0.61
2.45	0.54	101.07	0.87	0.19	247.62	0.84
1.82	0.28	193.02	1.06	0.16	351.30	0.86
4.1	0.37	105.67	2.11	0.19	433.25	0.93
2.51	0.45	92.06	1.17	0.21	231.06	0.94
3.93	0.3	249.32	2.69	0.21	979.81	0.62
3.29	0.66	216.96	1.92	0.39	713.80	0.59
4.76	0.33	235.97	3.42	0.24	1123.22	0.62
3.39	0.51	87.83	1.12	0.17	297.74	0.81
2.96	0.26	125.47	1.84	0.16	371.38	1.10
2.48	0.33	118.01	1.54	0.20	292.67	0.43
4.06	0.61	90.88	1.48	0.22	368.96	0.66
3.6	0.55	278.29	2.79	0.43	1001.86	0.67
1.3	0.35	148.34	0.86	0.23	192.85	0.7
1.43	0.3	134.16	0.89	0.19	191.85	0.84
4.38	0.64	80.08	1.68	0.25	350.74	0.4
3.18	0.47	164.86	2.15	0.32	524.27	0.49
1.71	0.28	164.07	1.02	0.17	280.56	0.58
3.82	0.3	168.16	2.13	0.17	642.36	0.78
2.34	0.29	311.56	1.59	0.20	729.06	0.9
1.83	0.21	81.92	1.03	0.12	149.92	0.35
4.22	0.53	90.43	1.83	0.23	381.63	0.4
1.99	0.28	313.40	2.05	0.29	623.67	0.42

3.29	0.48	115.44	1.21	0.18	379.80	0.44
2.64	0.19	165.31	1.80	0.13	436.41	0.45
4.43	0.21	219.39	2.68	0.13	971.92	0.46
2.51	0.3	90.36	1.27	0.15	226.79	0.47
1.7	0.19	130.38	1.08	0.12	221.65	0.48
3	0.62	110.77	1.54	0.32	332.31	0.49
3.99	0.58	130.88	1.96	0.28	522.22	0.49
5.2	0.26	166.96	4.52	0.23	868.17	0.5
1.83	0.4	97.19	1.03	0.22	177.86	0.51
2.51	0.27	103.74	1.17	0.13	260.38	0.51
2.48	0.54	119.65	1.08	0.24	296.73	0.53
3.52	0.25	158.88	1.79	0.13	559.27	0.53
4.18	0.42	141.47	1.93	0.19	591.36	0.53
1.66	0.25	105.52	0.92	0.14	175.17	0.54
1.63	0.18	142.64	1.26	0.14	232.50	0.54
4.52	0.44	85.00	2.26	0.22	384.20	0.54
1.76	0.43	123.81	1.05	0.26	217.90	0.55
5.73	0.4	198.99	2.88	0.20	1140.24	0.55
4.5	0.42	138.02	2.34	0.22	621.09	0.56
2.74	0.35	73.22	1.50	0.19	200.63	0.59
2.23	0.52	98.79	0.90	0.21	220.30	0.61
2.53	0.21	202.15	1.36	0.11	511.44	0.63
3.63	0.41	227.41	2.69	0.30	825.49	0.65
2.65	0.32	85.77	1.11	0.13	227.30	0.67
3.78	0.43	141.18	2.22	0.25	533.65	0.67
4.94	0.56	212.37	2.66	0.30	1049.09	0.69
2.21	0.23	151.95	1.44	0.15	335.81	0.7
3.84	0.72	112.87	1.15	0.22	433.44	0.71
2.31	0.3	239.09	2.10	0.27	552.30	0.71
4.82	0.47	208.82	2.03	0.20	1006.53	0.71
5.65	0.46	203.45	3.90	0.32	1149.48	0.71
2.05	0.35	111.56	1.18	0.20	228.70	0.72
1.47	0.38	233.33	1.17	0.30	343.00	0.73
3.19	0.52	175.52	1.32	0.22	559.90	0.73
3.24	0.46	211.40	1.42	0.20	684.95	0.73
4.16	0.33	164.77	2.36	0.19	685.45	0.73
9.35	0.64	304.41	13.75	0.94	2846.25	0.73
3.06	0.25	63.64	1.55	0.13	194.73	0.74
2.25	0.2	140.12	1.39	0.12	315.28	0.74
3.6	0.54	119.44	1.25	0.19	430.00	0.74
2.69	0.48	185.29	1.98	0.35	498.44	0.75
3.23	0.3	153.78	2.71	0.25	496.71	0.76
1.34	0.2	257.43	1.33	0.20	344.95	0.78
3.1	0.33	268.14	2.74	0.29	831.24	0.78
4.06	0.29	134.33	2.02	0.14	545.37	0.8
2.1	0.49	130.43	1.01	0.24	273.91	0.82
3.99	0.55	116.17	2.39	0.33	463.51	0.82
2.73	0.46	271.11	2.02	0.34	740.13	0.83

3.43	0.4	130.95	1.63	0.19	449.17	0.84
3.64	0.68	113.99	1.50	0.28	414.93	0.85
2.51	0.36	102.14	0.89	0.13	256.36	0.86
4.67	0.54	95.96	1.72	0.20	448.11	0.87
4.94	0.47	110.26	2.53	0.24	544.67	0.89
1.89	0.38	113.99	0.98	0.20	215.44	0.9
2.45	0.22	97.63	1.45	0.13	239.20	0.92
1.92	0.45	135.05	0.99	0.23	259.30	0.92
2.37	0.4	131.33	1.43	0.24	311.24	0.92
3.59	0.47	152.17	1.56	0.20	546.30	0.92
4.55	0.5	124.34	2.41	0.26	565.74	0.92
2.11	0.3	75.20	1.69	0.24	158.67	0.93
6.14	0.26	145.45	3.10	0.13	893.09	0.93
2.3	0.41	149.59	1.90	0.34	344.05	0.94
2.5	0.32	187.97	1.88	0.24	469.92	0.95
3.06	0.43	156.52	1.66	0.23	478.96	0.97
3.04	0.4	193.30	1.57	0.21	587.63	0.97
2.21	0.23	130.85	1.10	0.11	289.17	0.98
1.85	0.42	169.62	1.17	0.27	313.80	0.98
3.46	0.32	127.14	1.74	0.16	439.89	0.98
2.80	0.55	172.25	1.62	0.32	482.31	0.98
5.56	0.27	118.65	2.88	0.14	659.71	1.02
2.53	0.32	237.35	1.52	0.19	600.49	1.04
7.6	0.54	204.62	5.85	0.42	1555.08	1.05
2.76	0.45	66.06	1.27	0.21	182.31	1.07
2.65	0.44	110.65	1.23	0.20	293.22	1.08
3.05	0.29	141.61	2.23	0.21	431.90	1.12
5.62	0.44	91.85	2.41	0.19	516.17	1.12
2.19	0.47	291.67	1.83	0.39	638.75	1.14
2.52	0.25	136.54	1.21	0.12	344.08	1.17
4.27	0.41	109.66	1.79	0.17	468.26	1.18
2.32	0.36	68.49	0.97	0.15	158.89	1.2
4.91	0.27	93.00	1.43	0.08	456.64	1.2
3.65	0.39	188.39	2.35	0.25	687.61	1.2
2.9	0.3	113.33	1.38	0.14	328.67	1.26
5.99	0.66	192.75	4.34	0.48	1154.59	1.26
6.23	0.52	175.97	4.83	0.40	1096.29	1.27
3.98	0.31	223.97	3.29	0.26	891.39	1.28
1.75	0.38	101.03	0.90	0.19	176.79	1.33
1.92	0.47	100.00	0.94	0.23	192.00	1.35
1.51	0.22	136.29	0.64	0.09	205.79	1.36
3.27	0.54	98.60	1.52	0.25	322.44	1.41
3.44	0.49	104.98	1.71	0.24	361.11	1.41
5.22	0.23	192.51	2.79	0.12	1004.92	1.42
4.06	0.3	183.16	2.07	0.15	743.64	1.45
5.52	0.64	140.17	2.31	0.27	773.72	1.54
5.82	0.48	135.78	2.51	0.21	790.22	1.55
3.54	0.51	94.79	1.15	0.17	335.55	1.65

2.7	0.29	151.87	1.12	0.12	410.04	1.65
6.48	0.48	111.89	2.85	0.21	725.07	1.65
4.04	0.38	173.25	2.57	0.24	699.92	1.69
5.09	0.7	87.37	1.79	0.25	444.71	1.81
7.82	0.81	179.75	4.95	0.51	1405.62	1.87
2.98	0.42	110.95	1.42	0.20	330.64	1.9
5.52	0.49	177.27	2.51	0.22	978.55	1.9
4.25	0.41	153.53	1.76	0.17	652.49	1.92
4.75	0.36	121.76	2.20	0.17	578.36	1.93
1.86	0.42	104.82	1.12	0.25	194.96	1.97
4.38	0.6	115.98	2.26	0.31	507.99	2.27
8.07	0.51	232.14	5.76	0.36	1873.39	2.36
5.46	0.48	280.79	3.08	0.27	1533.12	2.51
2.19	0.34	90.28	1.01	0.16	197.71	2.68
6.55	0.57	132.25	2.37	0.21	866.21	3.14
10.34	0.18	172.06	7.60	0.13	1779.09	4.85
1.9	0.21	144.39	0.93	0.10	274.34	0.27
4.78	0.33	133.51	2.54	0.18	638.18	0.36
1.66	0.39	156.08	1.12	0.26	259.09	0.37
2.4	0.13	278.45	2.07	0.11	668.28	0.37
2.55	0.33	145.74	1.36	0.18	371.65	0.41
3.14	0.35	138.51	1.95	0.22	434.92	0.43
4.71	0.23	254.30	3.12	0.15	1197.77	0.44
2.3	0.4	116.77	1.48	0.26	268.58	0.45
2.5	0.37	165.24	1.52	0.23	413.11	0.45
5.26	0.26	200.81	4.28	0.21	1056.28	0.45
1.68	0.35	192.37	1.42	0.30	323.19	0.46
3.8	0.52	92.79	1.71	0.23	352.61	0.46
2.12	0.13	146.38	1.54	0.09	310.32	0.49
2.81	0.34	132.84	2.10	0.25	373.27	0.5
2.65	0.41	118.27	1.35	0.21	313.43	0.51
2.71	0.43	188.29	2.44	0.39	510.26	0.52
3.9	0.2	125.54	2.12	0.11	489.62	0.53
2.78	0.36	256.32	3.20	0.41	712.57	0.53
1.89	0.28	72.73	1.32	0.20	137.45	0.54
6.01	0.43	148.54	2.92	0.21	892.75	0.54
2.2	0.25	168.66	1.64	0.19	371.04	0.55
2.29	0.53	138.04	1.24	0.29	316.12	0.56
2.23	0.48	144.14	1.00	0.22	321.44	0.58
3.95	0.46	104.35	1.56	0.18	412.17	0.58
3.75	0.33	115.56	1.67	0.15	433.33	0.58
2.96	0.31	109.52	1.57	0.16	324.19	0.59
1.9	0.73	186.55	0.85	0.33	354.44	0.59
3.22	0.41	64.75	1.32	0.17	208.51	0.6
3.04	0.31	201.77	2.69	0.27	613.38	0.6
2.52	0.4	117.65	1.24	0.20	296.47	0.63
3.35	0.27	85.71	1.09	0.09	287.14	0.66
3.54	0.32	107.05	1.56	0.14	378.95	0.67

2.85	0.44	177.36	1.79	0.28	505.47	0.67
3.65	0.7	112.37	1.88	0.36	410.15	0.69
5.57	0.77	198.01	5.54	0.77	1102.92	0.7
2.93	0.29	463.06	2.64	0.26	1356.77	0.70
3.08	0.21	233.33	2.33	0.16	718.67	0.72
1.83	0.27	147.51	1.01	0.15	269.95	0.73
4.01	0.75	150.91	2.43	0.45	605.15	0.75
2.13	0.24	187.82	1.37	0.15	400.06	0.76
2	0.42	121.16	0.83	0.17	242.32	0.77
2.26	0.53	264.38	1.55	0.36	597.51	0.77
6.58	0.77	130.00	3.29	0.39	855.40	0.78
3.2	0.47	96.11	1.25	0.18	307.55	0.8
2.81	0.21	136.90	1.67	0.13	384.70	0.8
2.88	0.18	122.96	1.47	0.09	354.12	0.81
5.07	0.8	131.55	1.60	0.25	666.94	0.81
2.05	0.38	100.94	0.96	0.18	206.92	0.83
1.61	0.35	205.63	1.01	0.22	331.06	0.83
2.24	0.27	158.02	1.71	0.21	353.95	0.84
2.43	0.43	225.78	1.90	0.34	548.65	0.84
1.84	0.33	82.20	0.78	0.14	151.25	0.85
2.38	0.2	187.13	2.36	0.20	445.37	0.86
2.38	0.31	96.73	0.71	0.09	230.21	0.87
3.46	0.27	203.97	2.75	0.21	705.73	0.87
3.67	0.53	183.87	1.69	0.24	674.81	0.88
2.52	0.38	230.16	2.00	0.30	580.00	0.89
4.03	0.6	101.30	1.74	0.26	408.23	0.9
3.45	0.64	141.59	1.53	0.28	488.50	0.9
2.63	0.38	123.56	1.17	0.17	324.95	0.91
2.53	0.45	108.00	1.12	0.20	273.24	0.92
2.54	0.33	179.62	1.62	0.21	456.23	0.93
3.79	0.33	200.00	2.58	0.22	758.00	0.93
2.83	0.35	323.57	2.02	0.25	915.71	0.93
4.39	0.57	124.80	1.76	0.23	547.87	0.94
2.88	0.39	204.72	2.27	0.31	589.61	0.94
2.57	0.16	146.79	1.18	0.07	377.25	0.96
1.51	0.46	264.42	1.45	0.44	399.28	0.96
2.7	0.39	158.10	1.07	0.15	426.88	0.96
3.3	0.8	92.75	1.00	0.24	306.07	0.97
2.08	0.27	145.52	1.55	0.20	302.69	1
1.79	0.26	218.80	1.35	0.20	391.65	1
3.07	0.35	258.02	2.34	0.27	792.11	1
2.28	0.39	220.16	1.84	0.31	501.97	1.02
5.65	0.45	106.43	3.30	0.26	601.35	1.02
1.69	0.19	185.33	1.13	0.13	313.21	1.04
2.72	0.29	117.43	1.13	0.12	319.40	1.04
6.34	0.65	205.17	5.47	0.56	1300.79	1.05
3.65	0.18	115.81	1.56	0.08	422.71	1.07
2.31	0.34	128.81	0.98	0.14	297.56	1.09

3.12	0.38	179.64	1.87	0.23	560.48	1.12
2.69	0.33	90.40	1.08	0.13	243.18	1.16
7.52	0.31	271.81	5.05	0.21	2044.03	1.17
3.53	0.39	276.26	2.54	0.28	975.19	1.18
2.56	0.42	72.73	0.97	0.16	186.18	1.19
3.14	0.48	122.76	1.28	0.20	385.48	1.2
2.77	0.36	156.41	1.78	0.23	433.26	1.2
2.91	0.24	158.44	1.89	0.16	461.06	1.22
6.32	0.60	133.53	3.78	0.36	843.93	1.23
5.19	0.59	150.68	2.35	0.27	782.02	1.25
3.61	0.52	148.53	1.77	0.25	536.19	1.29
3.99	0.37	93.24	1.42	0.13	372.02	1.37
3.76	0.42	191.74	1.72	0.19	720.95	1.38
2.63	0.47	200.91	1.20	0.21	528.39	1.41
2.42	0.38	163.01	1.40	0.22	394.47	1.43
2.45	0.4	194.96	1.76	0.29	477.66	1.43
6.15	0.41	119.05	4.88	0.33	732.14	1.43
3.03	0.55	271.31	2.48	0.45	822.07	1.44
4.3	0.38	140.21	2.28	0.20	602.91	1.48
1.92	0.41	81.75	0.73	0.16	156.96	1.51
8.36	0.48	522.22	13.27	0.76	4365.78	1.57
2.48	0.55	128.24	1.15	0.25	318.04	1.58
4.66	0.54	91.02	1.82	0.21	424.13	1.58
3.69	0.27	123.98	1.67	0.12	457.49	1.62
3.91	0.3	225.44	3.43	0.26	881.46	1.63
2.93	0.44	128.19	1.29	0.19	375.61	1.67
2.19	0.18	202.94	1.29	0.11	444.44	1.73
2.12	0.43	159.85	1.55	0.31	338.89	1.76
5.26	0.55	268.89	5.84	0.61	1414.36	1.8
1.79	0.25	204.05	1.21	0.17	365.26	1.81
2.9	0.27	278.75	1.81	0.17	808.38	1.82
3.22	0.71	133.45	1.09	0.24	429.70	1.85
5.16	0.52	126.43	1.84	0.19	652.37	1.85
9.17	1.08	170.80	4.06	0.48	1566.20	1.9
2.2	0.74	207.10	1.30	0.44	455.62	1.92
5.73	0.55	142.08	2.59	0.25	814.13	1.94
5.64	0.41	197.42	3.64	0.26	1113.45	2.1
4.61	0.36	304.29	3.29	0.26	1402.76	2.18
6.28	0.8	151.18	3.69	0.47	949.39	2.43
5.77	0.44	214.58	6.01	0.46	1238.15	2.81
4.8	0.3	110.91	1.75	0.11	532.36	3.24
3.32	0.5	109.59	1.52	0.23	363.84	4.09
12.47	0.81	156.90	10.75	0.70	1956.50	4.34
4.35	0.29	107.37	1.53	0.10	467.05	4.39
4.99	0.58	80.69	1.93	0.22	402.67	0.66
6.08	0.65	129.63	2.50	0.27	788.15	0.71
2.83	0.47	213.89	1.57	0.26	605.31	0.79
6.03	1	69.84	2.39	0.40	421.14	0.7

3.89	0.41	58.09	1.61	0.17	225.98	0.49
1.94	0.34	183.69	1.38	0.24	356.35	0.53
3.65	0.38	171.43	3.48	0.36	625.71	0.55
3.31	0.27	86.57	1.65	0.13	286.54	0.71
4.23	0.41	151.28	2.71	0.26	639.92	0.47
4.2	0.38	237.33	2.80	0.25	996.80	0.75
2.95	0.63	134.68	1.71	0.36	397.31	0.87
3.83	0.36	124.57	2.19	0.21	477.11	0.94
3.44	0.56	102.44	1.40	0.23	352.39	0.39
1.76	0.3	65.23	0.63	0.11	114.81	0.52
2.93	0.35	100.00	1.48	0.18	293.00	0.85
2.6	0.57	132.37	0.94	0.21	344.17	0.86
3	0.55	247.83	1.63	0.30	743.48	0.86
2.31	0.36	292.31	1.78	0.28	675.23	1.02
3.39	0.51	97.97	1.38	0.21	332.11	0.5
3.46	0.26	220.00	1.92	0.14	761.20	0.73
3.2	0.35	292.05	2.12	0.23	934.57	0.59
3.86	0.39	118.26	1.76	0.18	456.50	0.7
3.76	0.45	142.49	1.61	0.19	535.76	0.89
2.6	0.26	315.32	2.34	0.23	819.82	0.51
2.59	0.34	268.75	1.80	0.24	696.06	0.7
4.54	0.7	121.40	1.87	0.29	551.15	1.06
5.24	0.38	98.25	2.29	0.17	514.85	0.41
3.52	0.26	345.10	3.45	0.25	1214.75	0.6
3.64	0.57	123.55	1.50	0.24	449.74	0.76
3.47	0.24	152.83	2.18	0.15	530.32	1.04
6.18	0.35	156.91	3.41	0.19	969.68	1.04
1.87	0.43	148.15	0.99	0.23	277.04	1.25
2.13	0.43	168.18	1.38	0.28	358.23	1.6
5.17	0.48	150.19	2.00	0.19	776.50	1.62
4.18	0.61	176.42	3.40	0.50	737.45	1.92
3.99	0.35	89.18	1.73	0.15	355.82	2.08
3.17	0.31	84.55	1.29	0.13	268.03	0.74
2.68	0.51	233.33	2.03	0.39	625.33	0.75
3.26	0.29	228.44	2.99	0.27	744.72	0.85
5.12	0.5	158.26	2.35	0.23	810.28	1.73
2.49	0.31	273.81	1.98	0.25	681.79	0.4
3.45	0.38	109.30	2.01	0.22	377.09	0.41
2.64	0.28	147.44	1.69	0.18	389.23	0.48
4.55	0.46	233.68	2.39	0.24	1063.26	0.51
3.04	0.56	169.18	2.08	0.38	514.30	0.54
2.83	0.28	177.50	1.77	0.18	502.33	0.7
1.08	0.29	198.46	0.83	0.22	214.34	0.72
1.57	0.27	189.86	1.06	0.18	298.09	0.53
2.31	0.28	196.12	2.24	0.27	453.03	0.59
1.98	0.32	234.32	1.17	0.19	463.95	0.67
3.51	0.47	145.73	2.14	0.29	511.52	0.62
2.19	0.4	126.63	1.19	0.22	277.32	0.75

3.12	0.66	118.18	1.42	0.30	368.73	0.72
3.69	0.96	72.39	1.24	0.32	267.12	0.81
2.87	0.66	140.17	1.23	0.28	402.29	0.46
5.77	0.39	143.48	4.18	0.28	827.87	0.6
2.62	0.3	268.28	1.81	0.21	702.88	0.73
2.13	0.31	221.35	1.11	0.16	471.48	0.77
1.99	0.36	200.64	1.27	0.23	399.27	0.73
4.8	0.43	145.93	1.78	0.16	700.44	0.87
2.75	0.19	174.26	2.02	0.14	479.23	0.88
2.83	0.37	210.06	1.67	0.22	594.47	1.7
1.74	0.32	212.26	1.12	0.21	369.33	0.73
2.18	0.32	73.28	0.83	0.12	159.76	0.79
2.27	0.32	332.79	1.86	0.26	755.43	0.42
3.2	0.22	297.46	2.71	0.19	951.86	0.44
4.61	0.34	112.73	2.10	0.15	519.67	0.46
1.57	0.29	196.77	1.01	0.19	308.94	0.49
2.71	0.27	133.13	1.66	0.17	360.78	0.49
3.44	0.28	224.34	2.26	0.18	771.74	0.5
1.1	0.42	179.56	0.61	0.23	197.51	0.53
2.39	0.22	255.06	2.69	0.25	609.58	0.53
3.81	0.52	134.15	2.32	0.32	511.10	0.54
4.43	0.24	209.49	2.80	0.15	928.06	0.54
3.56	0.69	106.08	1.35	0.26	377.66	0.55
3.05	0.27	141.38	1.50	0.13	431.21	0.55
3.39	0.44	93.59	1.21	0.16	317.28	0.57
1.48	0.47	167.39	0.80	0.26	247.74	0.6
2.42	0.47	124.03	1.88	0.36	300.16	0.61
4.32	0.36	176.06	2.30	0.19	760.60	0.61
0.83	0.26	152.59	0.36	0.11	126.65	0.63
3.96	0.46	139.90	2.05	0.24	553.99	0.63
3.36	0.51	59.95	0.89	0.14	201.42	0.65
5.55	0.45	172.83	3.21	0.26	959.22	0.65
3.46	0.33	109.93	1.23	0.12	380.35	0.66
3.13	0.35	141.18	1.67	0.19	441.88	0.66
2.42	0.34	124.87	1.23	0.17	302.19	0.69
3.64	0.65	113.13	1.84	0.33	411.80	0.69
3.56	0.5	106.91	1.64	0.23	380.61	0.7
2.2	0.35	75.00	1.31	0.21	165.00	0.71
2.93	0.53	117.28	1.53	0.28	343.62	0.72
1.76	0.23	266.19	1.27	0.17	468.49	0.72
3.1	0.35	187.57	1.75	0.20	581.47	0.72
2.32	0.4	129.84	1.21	0.21	301.24	0.73
2.97	0.26	115.30	1.06	0.09	342.45	0.74
2.53	0.21	148.05	1.64	0.14	374.57	0.74
3.44	0.37	107.57	1.37	0.15	370.04	0.75
4.19	0.4	208.93	2.49	0.24	875.41	0.75
2.42	0.32	140.46	1.40	0.18	339.92	0.76
3.77	0.2	153.33	2.09	0.11	578.07	0.76

3.83	0.31	167.80	2.16	0.18	642.66	0.77
4.34	0.35	155.76	2.00	0.16	676.00	0.77
2.04	0.27	140.93	1.06	0.14	287.50	0.78
3.21	0.68	124.53	2.02	0.43	399.74	0.79
3.29	0.54	116.11	1.83	0.30	382.01	0.8
2.39	0.42	61.68	0.72	0.13	147.41	0.81
3.98	0.25	187.50	3.11	0.20	746.25	0.81
3.13	0.31	150.73	1.53	0.15	471.79	0.82
2.7	0.39	180.00	1.80	0.26	486.00	0.82
5.51	0.35	115.14	2.53	0.16	634.41	0.83
2.57	0.23	397.10	3.72	0.33	1020.55	0.83
2.04	0.27	106.94	0.94	0.13	218.17	0.89
3.56	0.23	229.83	1.97	0.13	818.21	0.89
2.56	0.21	136.23	1.86	0.15	348.75	0.9
4.67	0.58	108.70	2.54	0.32	507.61	0.9
3.17	0.42	128.37	2.25	0.30	406.93	0.92
1.63	0.19	217.70	1.44	0.17	354.85	0.94
2.82	0.43	218.49	2.37	0.36	616.13	0.94
3.21	0.5	131.62	1.37	0.21	422.51	0.95
1.84	0.29	109.78	0.82	0.13	201.99	0.96
2.11	0.23	127.74	1.36	0.15	269.54	0.96
1.63	0.2	247.46	1.38	0.17	403.36	0.99
2.85	0.29	147.50	1.78	0.18	420.38	1
3.15	0.32	176.92	2.69	0.27	557.31	1
4.07	0.36	132.57	1.87	0.17	539.56	1.01
2.44	0.33	158.12	1.28	0.17	385.80	1.02
6.74	0.32	328.95	8.87	0.42	2217.11	1.02
2.41	0.23	226.89	2.03	0.19	546.81	1.03
3.44	0.22	269.66	2.37	0.15	927.61	1.03
3.09	0.58	122.09	0.95	0.18	377.25	1.05
3.37	0.44	118.57	1.42	0.19	399.57	1.07
2.44	0.38	176.97	1.61	0.25	431.82	1.07
6.08	0.42	268.70	4.64	0.32	1633.71	1.07
2.61	0.37	91.90	1.06	0.15	239.87	1.09
3.8	0.23	132.77	1.60	0.10	504.54	1.12
3.62	0.51	148.44	1.89	0.27	537.34	1.13
5.46	0.44	102.24	4.07	0.33	558.22	1.13
2.01	0.25	172.15	1.27	0.16	346.03	1.14
3.77	0.45	105.84	1.38	0.16	399.01	1.14
2.82	0.27	200.58	1.64	0.16	565.64	1.17
2.57	0.25	122.22	1.36	0.13	314.11	1.18
4.4	0.69	116.96	1.52	0.24	514.60	1.19
3.71	0.36	147.06	1.98	0.19	545.59	1.2
2.27	0.46	77.78	0.81	0.16	176.56	1.21
3.44	0.66	104.64	1.45	0.28	359.97	1.21
1.96	0.47	70.59	0.72	0.17	138.35	1.24
4.49	0.31	142.92	2.12	0.15	641.73	1.25
3.41	0.64	112.44	1.77	0.33	383.40	1.26

2.72	0.62	99.06	0.85	0.19	269.45	1.28
2.59	0.24	141.58	1.28	0.12	366.70	1.32
4.05	0.36	196.73	2.65	0.24	796.76	1.33
3.32	0.32	167.70	1.47	0.14	556.76	1.34
5.41	0.28	137.29	2.29	0.12	742.73	1.42
3.86	0.4	155.12	1.88	0.20	598.77	1.43
2.67	0.57	176.56	1.39	0.30	471.42	1.45
7.45	0.4	270.29	4.26	0.23	2013.63	1.45
2.85	0.35	92.70	1.22	0.15	264.21	1.47
9.25	0.31	462.07	15.95	0.53	4274.14	1.48
3.87	0.31	162.82	1.65	0.13	630.12	1.49
5.44	0.67	100.37	1.99	0.25	545.99	1.5
2.72	0.49	118.12	1.97	0.36	321.28	1.51
2.57	0.38	189.54	1.68	0.25	487.12	1.52
5.01	0.41	221.59	5.69	0.47	1110.17	1.52
4.27	0.46	183.94	2.21	0.24	785.41	1.55
2.95	0.61	78.60	1.21	0.25	231.87	1.59
6.91	0.54	94.64	3.08	0.24	653.98	1.63
1.71	0.41	121.62	1.16	0.28	207.97	1.68
4	0.46	89.18	2.06	0.24	356.70	1.72
2.64	0.67	137.07	1.14	0.29	361.86	1.77
6.07	0.39	114.04	2.58	0.17	692.24	1.81
6.64	0.6	151.33	2.94	0.27	1004.81	1.87
4.89	0.38	125.10	1.98	0.15	611.74	1.89
2.44	0.42	244.96	1.89	0.33	597.71	1.92
4.59	0.27	88.68	1.24	0.07	407.04	1.93
3.66	0.54	233.54	2.32	0.34	854.77	2.02
4.42	0.4	186.55	2.58	0.23	824.55	2.03
3.26	0.35	155.68	1.85	0.20	507.52	2.06
5.79	0.48	129.12	2.22	0.18	747.60	2.16
2.69	0.4	82.86	1.28	0.19	222.89	2.26
6.18	0.42	163.06	3.94	0.27	1007.69	2.39
3.76	0.65	96.74	1.12	0.19	363.73	3.46
10.65	0.72	422.67	14.20	0.96	4501.40	4.31
3.51	0.52	296.85	2.76	0.41	1041.94	0.28
1.9	0.25	315.25	1.61	0.21	598.98	0.33
2.47	0.2	130.00	1.54	0.13	321.10	0.37
2.71	0.37	146.73	1.36	0.19	397.65	0.44
2.19	0.52	194.83	1.89	0.45	426.67	0.44
2.3	0.3	211.38	1.38	0.18	486.17	0.46
3.71	0.11	216.67	2.47	0.07	803.83	0.51
3.37	0.5	130.54	2.02	0.30	439.92	0.52
3.82	0.55	120.09	1.74	0.25	458.75	0.52
2.97	0.26	103.82	1.89	0.17	308.35	0.54
2.02	0.45	197.42	1.30	0.29	398.79	0.55
1.68	0.36	139.34	0.92	0.20	234.10	0.56
3.18	0.22	124.14	1.57	0.11	394.76	0.56
3.6	0.28	167.63	2.08	0.16	603.47	0.56

3.75	0.37	115.52	2.16	0.21	433.19	0.58
5.52	0.42	134.80	2.71	0.21	744.12	0.61
1.79	0.27	330.34	2.01	0.30	591.30	0.62
4.18	0.54	149.73	2.28	0.30	625.86	0.62
2.59	0.44	96.60	1.10	0.19	250.18	0.64
4.16	0.34	162.50	2.08	0.17	676.00	0.64
3.31	0.24	146.67	3.15	0.23	485.47	0.66
1.98	0.31	109.14	1.01	0.16	216.09	0.67
5.28	0.34	128.64	2.56	0.17	679.22	0.67
1.99	0.35	196.57	1.14	0.20	391.18	0.68
2.66	0.42	133.19	1.15	0.18	354.28	0.69
6.46	0.42	93.46	3.02	0.20	603.74	0.69
1.83	0.15	104.71	1.08	0.09	191.61	0.7
3.9	0.35	154.89	2.93	0.26	604.06	0.74
5.42	0.28	183.87	3.50	0.18	996.58	0.74
3.35	0.65	121.21	1.69	0.33	406.06	0.75
2.61	0.35	193.01	1.14	0.15	503.76	0.75
4.52	0.5	211.92	2.34	0.26	957.87	0.75
2.76	0.36	130.91	1.67	0.22	361.31	0.77
3.96	0.52	154.19	2.21	0.29	610.59	0.77
1.24	0.28	111.54	0.68	0.15	138.31	0.79
2	0.25	155.23	1.16	0.15	310.47	0.79
3.86	0.47	242.78	2.06	0.25	937.13	0.8
2.67	0.45	208.41	1.25	0.21	556.46	0.82
1.73	0.38	144.10	0.89	0.19	249.30	0.83
3.02	0.45	122.11	1.52	0.23	368.77	0.83
3.3	0.57	119.76	1.98	0.34	395.21	0.83
4.5	0.4	203.28	2.46	0.22	914.75	0.83
1.86	0.42	172.32	1.05	0.24	320.51	0.86
5.88	0.3	225.00	4.20	0.21	1323.00	0.86
2.84	0.57	113.66	1.25	0.25	322.78	0.87
2.26	0.32	178.17	1.15	0.16	402.67	0.88
2.76	0.6	205.95	3.29	0.71	568.43	0.88
2.12	0.28	185.48	1.71	0.23	393.23	0.89
2.88	0.34	184.21	1.52	0.18	530.53	0.89
3.2	0.33	144.24	1.47	0.15	461.57	0.9
3.35	0.26	194.89	1.90	0.15	652.87	0.9
3.84	0.29	190.96	2.17	0.16	733.29	0.92
3.69	0.46	173.97	2.53	0.32	641.96	0.94
2.12	0.51	80.99	0.88	0.21	171.70	0.95
4.98	0.39	277.48	4.49	0.35	1381.84	0.95
1.64	0.38	153.94	0.99	0.23	252.46	0.96
2.71	0.74	102.31	1.25	0.34	277.27	0.98
5.09	0.76	153.47	3.53	0.53	781.17	1
3.56	0.34	92.55	1.26	0.12	329.49	1.02
3.18	0.25	181.45	1.28	0.10	577.02	1.02
3.09	0.31	165.12	2.40	0.24	510.21	1.05
3.47	0.43	87.03	1.88	0.23	301.98	1.09

3.29	0.59	171.72	1.66	0.30	564.95	1.11
2.78	0.31	125.22	1.23	0.14	348.12	1.13
3.49	0.29	204.79	2.39	0.20	714.73	1.13
3.64	0.48	108.25	1.77	0.23	394.04	1.14
3.01	0.21	124.42	1.75	0.12	374.50	1.18
2.82	0.38	235.20	1.44	0.19	663.28	1.19
2.92	0.26	136.53	1.75	0.16	398.66	1.2
3.13	0.17	330.14	4.29	0.23	1033.33	1.2
3.21	0.35	196.65	1.79	0.20	631.24	1.22
5.83	0.26	300.00	7.99	0.36	1749.00	1.22
3.69	0.32	125.68	2.02	0.17	463.77	1.24
3.09	0.3	170.48	1.47	0.14	526.77	1.24
2.12	0.36	150.50	1.05	0.18	319.05	1.27
3.27	0.35	95.65	1.78	0.19	312.78	1.28
3.17	0.67	78.57	1.19	0.25	249.07	1.3
1.37	0.12	204.17	2.85	0.25	279.71	1.31
4.2	0.33	152.03	1.71	0.13	638.54	1.36
5.26	0.28	250.43	4.57	0.24	1317.29	1.38
3.21	0.48	106.69	1.34	0.20	342.49	1.41
3.7	0.5	90.52	1.21	0.16	334.93	1.43
3.91	0.47	130.00	1.70	0.20	508.30	1.44
3.96	0.44	109.80	1.94	0.22	434.82	1.48
4.08	0.43	209.74	2.09	0.22	855.75	1.51
3.74	0.75	278.38	2.02	0.41	1041.14	1.52
3.95	0.24	150.79	2.09	0.13	595.63	1.56
4.25	0.31	184.85	3.22	0.23	785.61	1.56
4.85	0.62	262.14	3.46	0.44	1271.39	1.58
3.42	0.65	142.50	2.14	0.41	487.35	1.64
2.33	0.37	238.36	3.19	0.51	555.37	1.66
3.82	0.62	108.30	1.32	0.21	413.72	1.69
4.73	0.79	118.39	2.72	0.45	559.99	1.73
5.43	0.46	106.06	1.83	0.15	575.91	1.79
2.99	0.45	180.10	1.45	0.22	538.49	1.8
3.34	0.37	288.19	2.63	0.29	962.55	1.84
4.92	0.39	201.75	2.16	0.17	992.63	1.84
3.13	0.36	100.83	1.30	0.15	315.60	1.87
5.28	0.66	147.50	4.40	0.55	778.80	1.89
2.79	0.31	119.43	1.32	0.15	333.21	1.93
4.93	0.49	142.78	2.64	0.26	703.91	1.96
3.99	0.48	110.50	2.20	0.27	440.88	2.01
3.03	0.37	152.28	1.54	0.19	461.42	2.13
4.72	0.58	142.93	2.57	0.32	674.65	2.37
11.28	0.33	170.37	13.93	0.41	1921.78	2.48
4.92	0.52	164.68	2.45	0.26	810.21	2.55
6.6	0.7	112.23	3.51	0.37	740.74	2.6
5.94	0.55	251.65	3.26	0.30	1494.79	3.15
3.91	0.38	83.02	1.04	0.10	324.62	3.72
8.41	0.65	111.65	2.72	0.21	938.98	4.53

10.7	1.11	62.18	2.72	0.28	665.36	5.29
10.04	0.4	402.38	11.95	0.48	4039.90	6.04
2.34	0.22	136.10	1.14	0.11	318.47	0.80
3.91	0.35	156.45	2.10	0.19	611.73	0.82
3.57	0.75	115.86	1.23	0.26	413.63	0.98
1.84	0.32	241.87	0.91	0.16	445.04	1.33
2.12	0.32	150.68	1.43	0.22	319.43	0.89
5	0.46	137.25	1.68	0.15	686.24	0.36
3.3	0.54	150.27	1.76	0.29	495.88	0.94
3.68	0.44	107.69	1.67	0.20	396.31	0.55
1.91	0.29	269.17	1.59	0.24	514.11	0.93
3.14	0.52	109.62	1.51	0.25	344.19	1.1
2.49	0.23	146.33	1.41	0.13	364.36	0.7
1.73	0.61	118.13	0.95	0.34	204.37	0.79
3.06	0.39	279.39	2.34	0.30	854.93	0.63
2.34	0.3	216.11	1.57	0.20	505.69	0.91
4.44	0.46	146.38	1.89	0.20	649.94	1.04
3.47	0.59	96.80	0.93	0.16	335.90	0.65
1.97	0.12	205.63	1.39	0.08	405.10	0.76
3.63	0.34	87.45	1.38	0.13	317.45	0.77
3.03	0.34	134.15	1.48	0.17	406.46	0.79
1.75	0.29	116.84	0.89	0.15	204.46	0.69
2.97	0.55	81.82	1.35	0.25	243.00	0.72
2.21	0.45	108.62	0.95	0.19	240.05	0.9
2.41	0.24	92.11	1.06	0.11	221.97	1.19
3.98	0.3	190.68	2.47	0.19	758.92	1.28
5.62	0.71	131.32	3.09	0.39	738.01	1.39
3.49	0.41	139.68	1.41	0.17	487.47	1.54
3.6	0.35	151.00	1.45	0.14	543.61	0.53
3.19	0.33	191.67	1.48	0.15	611.42	0.67
2.72	0.25	121.30	1.61	0.15	329.94	0.62
2.59	0.52	143.79	1.69	0.34	372.42	0.91
3.22	0.47	154.19	2.08	0.30	496.50	0.55
4.69	0.5	94.34	1.77	0.19	442.45	0.64
2.61	0.37	104.97	1.44	0.20	273.98	0.3
3.11	0.49	200.00	1.62	0.26	622.00	0.49
4.28	0.38	212.50	2.97	0.26	909.50	0.53
2.72	0.62	150.82	1.49	0.34	410.23	1.01
1.79	0.62	119.89	0.96	0.33	214.61	1.09
2.51	0.38	94.86	1.43	0.22	238.09	0.78
3.79	0.7	121.10	1.60	0.30	458.96	0.52
1.12	0.24	147.88	0.68	0.15	165.62	0.34
4.09	0.62	155.38	2.20	0.33	635.49	0.49
1.44	0.2	100.00	0.72	0.10	144.00	0.5
3.36	0.48	215.13	2.82	0.40	722.82	0.5
1.59	0.26	140.41	1.09	0.18	223.25	0.52
1.77	0.28	156.00	1.18	0.19	276.12	0.52
3.52	0.35	129.91	3.01	0.30	457.30	0.54

4.53	0.27	172.41	3.12	0.19	781.03	0.56
1.89	0.27	204.91	1.16	0.17	387.28	0.61
2.86	0.23	292.38	2.72	0.22	836.21	0.61
0.97	0.15	285.71	1.39	0.21	277.14	0.66
2.89	0.62	171.77	1.38	0.30	496.42	0.69
4.53	0.23	208.38	2.71	0.14	943.98	0.7
1.09	0.19	135.82	1.63	0.28	148.04	0.73
1.46	0.2	148.85	1.11	0.15	217.33	0.76
2.59	0.25	161.63	1.51	0.15	418.62	0.76
3.11	0.4	186.89	1.51	0.19	581.24	0.76
4.53	0.16	471.74	4.92	0.17	2136.98	0.77
2.36	0.37	254.24	2.00	0.31	600.00	0.79
2.83	0.46	82.33	1.22	0.20	232.99	0.8
3.69	0.51	120.51	1.89	0.26	444.69	0.8
2.31	0.2	183.72	1.79	0.16	424.40	0.81
4.67	0.37	105.96	3.09	0.25	494.83	0.83
1.82	0.21	66.04	0.86	0.10	120.19	0.86
2.82	0.37	166.86	1.64	0.22	470.55	0.87
3.56	0.49	156.25	1.48	0.20	556.25	0.87
1.41	0.28	102.33	0.82	0.16	144.28	0.88
3.91	0.32	85.04	1.54	0.13	332.50	0.88
3.09	0.26	116.35	1.94	0.16	359.53	0.89
1.67	0.36	101.02	0.85	0.18	168.70	0.9
4.81	0.31	228.75	3.01	0.19	1100.29	0.94
2.41	0.34	129.41	1.58	0.22	311.88	0.96
4.41	0.39	137.32	1.55	0.14	605.60	0.96
4.07	0.38	189.17	3.39	0.32	769.91	0.98
4.57	0.68	82.05	2.93	0.44	374.97	0.99
3.7	0.43	135.29	1.55	0.18	500.59	1.06
3.59	0.61	188.60	1.86	0.32	677.08	1.08
3.27	0.51	94.12	1.92	0.30	307.76	1.09
2.71	0.48	101.64	1.11	0.20	275.44	1.1
2.34	0.37	91.20	1.08	0.17	213.42	1.12
3.76	0.51	168.79	2.17	0.29	634.64	1.18
2.77	0.32	152.86	1.32	0.15	423.41	1.2
4.13	0.4	135.38	3.18	0.31	559.14	1.32
4.16	0.6	96.68	1.97	0.28	402.20	1.34
5.65	0.34	207.14	2.88	0.17	1170.36	1.42
2.03	0.42	97.41	0.66	0.14	197.74	1.47
4.25	0.27	115.25	1.91	0.12	489.80	1.48
4	0.24	184.51	2.82	0.17	738.03	1.49
4.11	0.31	112.50	2.70	0.20	462.38	1.5
5.93	0.56	174.03	3.85	0.36	1031.97	1.52
3.49	0.47	132.16	1.75	0.24	461.24	1.58
3.05	0.34	75.64	1.11	0.12	230.69	1.59
2.52	0.38	132.91	1.06	0.16	334.94	1.71
4.83	0.62	78.95	1.50	0.19	381.32	1.79
3.46	0.18	247.79	2.54	0.13	857.37	1.95

2.97	0.59	170.91	1.08	0.21	507.60	2
2.43	0.18	124.81	1.88	0.14	303.28	2.08
2.99	0.34	127.88	1.32	0.15	382.35	2.1
7.54	0.64	129.19	3.61	0.31	974.07	2.18
5.81	0.39	348.28	6.68	0.45	2023.48	2.19
3.11	0.57	109.50	1.56	0.29	340.55	2.29
3.52	0.31	230.06	2.16	0.19	809.82	4.38
4.37	0.58	86.90	1.51	0.20	379.74	0.45
5	0.31	101.36	2.26	0.14	506.79	0.45
3.86	0.35	157.87	2.17	0.20	609.36	0.49
2.11	0.33	213.92	2.67	0.42	451.38	0.52
3.74	0.42	121.36	1.82	0.20	453.88	0.53
2.8	0.33	122.64	1.32	0.16	343.40	0.6
2.46	0.76	107.09	0.97	0.30	263.43	0.62
2.98	0.35	89.08	1.30	0.15	265.47	0.63
1.3	0.22	451.14	1.48	0.25	586.48	0.69
2.86	0.54	111.20	1.14	0.22	318.03	0.71
2.43	0.42	160.84	1.46	0.25	390.85	0.71
2.13	0.31	112.00	1.42	0.21	238.56	0.72
1.81	0.49	142.52	1.43	0.39	257.96	0.76
1.77	0.29	165.41	1.33	0.22	292.78	0.78
3.02	0.44	112.57	1.58	0.23	339.95	0.79
3.23	0.36	124.47	1.72	0.19	402.03	0.79
3.27	0.23	185.95	1.77	0.12	608.04	0.8
5.58	0.57	180.75	3.47	0.35	1008.56	0.8
3.12	0.28	128.78	1.15	0.10	401.80	0.81
2.54	0.4	244.19	1.48	0.23	620.23	0.82
4.11	0.42	188.14	3.48	0.36	773.24	0.82
1.67	0.37	82.74	0.99	0.22	138.17	0.83
2.73	0.32	86.25	1.14	0.13	235.46	0.83
3.73	0.25	194.29	3.55	0.24	724.69	0.83
1.92	0.37	116.09	1.10	0.21	222.90	0.85
3.01	0.37	171.78	1.85	0.23	517.06	0.85
3.06	0.31	105.24	1.23	0.13	322.04	0.88
2.02	0.49	228.77	1.38	0.34	462.11	0.88
2.8	0.37	151.60	1.49	0.20	424.47	0.91
3.42	0.36	336.00	3.42	0.36	1149.12	0.92
2.23	0.24	134.29	0.91	0.10	299.46	0.94
3.73	0.23	164.58	2.59	0.16	613.90	0.96
2.77	0.39	114.29	1.72	0.24	316.57	0.99
1.66	0.29	110.29	1.22	0.21	183.09	1.01
2.89	0.48	160.00	1.86	0.31	462.40	1.05
1.97	0.28	109.05	0.81	0.12	214.84	1.16
2.1	0.63	214.55	1.91	0.57	450.55	1.16
4.07	0.62	137.29	2.30	0.35	558.76	1.29
9.1	0.72	297.62	10.83	0.86	2708.33	1.34
5.56	0.48	148.84	3.23	0.28	827.53	1.37
2.9	0.52	108.17	1.13	0.20	313.70	1.45

3.67	0.26	209.14	1.86	0.13	767.53	1.45
1.73	0.41	84.21	0.54	0.13	145.68	1.47
3.11	0.35	118.45	1.33	0.15	368.39	1.52
2.7	0.48	135.57	1.81	0.32	366.04	1.53
4.68	0.25	168.42	2.74	0.15	788.21	1.58
3.19	0.22	134.77	1.14	0.08	429.91	1.59
1.9	0.78	147.37	1.11	0.46	280.00	1.61
4.7	0.55	108.33	2.06	0.24	509.17	1.61
4.76	0.4	135.71	2.43	0.20	646.00	1.62
4.94	0.25	169.00	2.47	0.13	834.86	1.62
3.71	0.45	109.47	2.20	0.27	406.12	1.65
9.87	0.82	275.54	7.10	0.59	2719.58	1.71
3.72	0.46	208.96	1.85	0.23	777.31	1.78
3.74	0.38	139.69	1.93	0.20	522.44	1.82
10.33	0.9	364.81	9.56	0.83	3768.54	1.94
3.98	0.39	84.73	1.45	0.14	337.21	1.97
5.96	0.27	120.51	2.55	0.12	718.26	2.02
4.28	0.41	110.66	1.75	0.17	473.61	2.25
2.82	0.21	169.30	2.47	0.18	477.42	2.32
10.4	0.35	319.30	9.12	0.31	3320.70	2.33
7.14	0.75	284.67	4.76	0.50	2032.52	2.8
4.14	0.4	82.42	1.52	0.15	341.21	2.86
5.88	0.27	90.71	1.82	0.08	533.39	2.88
5.13	0.55	126.52	2.23	0.24	649.06	3.87
1.69	0.52	136.90	0.90	0.28	231.36	0.53
3.73	0.6	119.88	2.25	0.36	447.15	0.73
3.76	0.3	158.54	2.29	0.18	596.10	1.12
2.44	0.51	124.51	0.95	0.20	303.81	0.59
1.92	0.29	169.81	1.21	0.18	326.04	0.84
2.78	0.46	128.63	1.19	0.20	357.60	0.85
3.89	0.68	204.91	2.39	0.42	797.09	1.17
3.96	0.43	109.27	1.60	0.17	432.73	0.48
2.8	0.33	142.86	2.00	0.24	400.00	0.91
3.87	0.43	136.63	2.25	0.25	528.75	0.89
3.47	0.45	178.80	1.89	0.24	620.45	1.17
4.33	0.43	92.09	2.45	0.24	398.75	2.24
3.46	0.48	231.13	2.29	0.32	799.70	0.95
3.03	0.55	88.66	1.23	0.22	268.65	0.6
3.07	0.18	336.13	2.58	0.15	1031.93	0.39
2.22	0.39	126.44	1.07	0.19	280.70	0.42
2.86	0.52	241.78	1.96	0.36	691.49	0.54
3.06	0.54	128.57	1.37	0.24	393.43	0.86
3.75	0.46	199.56	1.66	0.20	748.34	0.85
3.53	0.37	179.81	1.70	0.18	634.72	1.1
4.2	0.32	107.85	2.20	0.17	452.98	0.34
4.09	0.33	123.32	1.62	0.13	504.38	0.47
2.72	0.26	129.47	1.43	0.14	352.17	0.48
2.05	0.39	133.94	1.24	0.24	274.58	0.52

3.5	0.49	135.25	2.87	0.40	473.36	0.52
3.65	0.3	193.33	2.21	0.18	705.67	0.52
2.93	0.37	134.59	1.58	0.20	394.36	0.54
2.04	0.29	164.33	1.19	0.17	335.23	0.58
3.66	0.39	150.49	1.78	0.19	550.78	0.61
2.17	0.4	193.09	1.00	0.18	419.00	0.62
5	0.66	121.05	2.63	0.35	605.26	0.66
4.08	0.72	150.22	1.81	0.32	612.91	0.71
2.89	0.3	82.09	1.44	0.15	237.24	0.76
3.14	0.55	125.89	1.40	0.25	395.30	0.8
3.98	0.16	264.23	3.24	0.13	1051.63	0.84
3.12	0.25	141.62	1.80	0.14	441.85	0.85
2.58	0.59	153.76	1.49	0.34	396.69	0.9
2.96	0.35	302.94	1.74	0.21	896.71	0.93
2.3	0.52	114.89	0.98	0.22	264.26	0.95
3.38	0.4	123.68	1.78	0.21	418.05	0.95
2.24	0.31	261.27	1.58	0.22	585.24	0.95
2.3	0.27	114.05	0.95	0.11	262.31	0.96
2.76	0.58	110.10	1.33	0.28	303.87	0.97
2.7	0.85	149.40	1.63	0.51	403.37	1.04
3.66	0.5	81.37	1.39	0.19	297.81	1.06
3.15	0.23	132.56	1.47	0.11	417.56	1.06
3.43	0.59	106.86	1.24	0.21	366.53	1.13
5.19	0.53	115.08	2.61	0.27	597.24	1.15
4.05	0.4	136.33	1.46	0.14	552.14	1.18
3.31	0.41	190.48	3.15	0.39	630.48	1.3
7.12	0.33	280.00	5.70	0.26	1993.60	1.35
3.13	0.16	168.55	2.52	0.13	527.56	1.37
2.56	0.27	121.60	1.20	0.13	311.29	1.38
2.65	0.33	275.81	2.14	0.27	730.89	1.53
5.81	0.36	207.50	3.63	0.23	1205.58	1.57
4.97	0.63	260.78	4.87	0.62	1296.10	1.68
3.39	0.25	159.83	1.42	0.10	541.83	1.72
2.99	0.31	123.93	1.83	0.19	370.54	2.22
9.1	0.47	219.85	6.69	0.35	2000.66	2.6
8.26	0.67	113.68	3.53	0.29	938.96	2.95
2.76	0.55	93.42	1.14	0.23	257.83	0.37
2.36	0.41	150.48	1.12	0.20	355.12	0.41
3	0.23	223.42	1.90	0.15	670.25	0.41
3.41	0.41	187.92	2.29	0.28	640.81	0.47
3.06	0.38	130.81	1.65	0.21	400.28	0.49
2.44	0.39	118.99	1.36	0.22	290.35	0.52
2.61	0.36	127.37	1.37	0.19	332.43	0.54
2.42	0.49	248.00	1.61	0.33	600.16	0.54
3.18	0.23	94.69	1.41	0.10	301.12	0.6
4.14	0.35	76.06	1.60	0.14	314.90	0.6
4.26	0.21	339.80	4.35	0.21	1447.53	0.67
2.81	0.84	95.19	1.35	0.40	267.49	0.69

2.25	0.18	141.67	1.44	0.12	318.75	0.72
3.46	0.41	149.74	1.77	0.21	518.11	0.76
2.9	0.55	113.50	1.06	0.20	329.16	0.77
2.61	0.25	373.56	3.00	0.29	975.00	0.78
2.63	0.54	73.38	0.95	0.19	192.99	0.79
4.91	0.37	160.94	2.56	0.19	790.20	0.79
4.17	0.3	206.36	2.41	0.17	860.51	0.82
1.58	0.27	81.41	0.79	0.14	128.62	0.83
1.81	0.41	240.74	1.34	0.30	435.74	0.85
3.84	0.59	100.00	2.34	0.36	384.00	0.87
2.33	0.28	92.12	1.15	0.14	214.64	0.89
3.91	0.34	140.57	1.84	0.16	549.61	0.93
2.74	0.34	121.02	1.56	0.19	331.60	0.97
2.68	0.34	193.72	1.40	0.18	519.16	0.97
3.7	0.4	314.29	3.30	0.36	1162.86	1.01
3.29	0.33	106.70	1.70	0.17	351.05	1.05
3.43	0.27	323.57	2.45	0.19	1109.85	1.12
2.72	0.31	153.76	1.57	0.18	418.22	1.14
5.23	0.42	287.50	3.85	0.31	1503.63	1.14
3.11	0.36	144.34	1.41	0.16	448.91	1.2
5.66	0.47	122.98	2.41	0.20	696.06	1.26
3.83	0.49	113.90	2.05	0.26	436.25	1.28
3.12	0.48	122.30	1.16	0.18	381.59	1.29
4.64	0.37	127.95	2.03	0.16	593.68	1.37
3.42	0.37	96.09	1.49	0.16	328.62	1.49
5.27	0.42	223.81	4.18	0.33	1179.48	1.49
2.94	0.3	274.85	1.76	0.18	808.06	1.51
5.07	0.74	188.17	3.00	0.44	954.00	1.55
6.36	0.45	114.80	2.54	0.18	730.13	1.62
2.46	0.43	171.03	1.70	0.30	420.74	1.68
2.99	0.36	72.49	1.31	0.16	216.74	1.76
5.56	0.23	160.77	4.28	0.18	893.88	1.86
3.96	0.66	99.07	1.85	0.31	392.30	1.94
5.03	0.41	191.34	3.96	0.32	962.43	2.09
7.14	0.65	146.15	2.50	0.23	1043.54	2.26
8.06	0.78	104.18	2.81	0.27	839.70	2.49
9.63	0.54	158.24	5.29	0.30	1523.87	5.15
2.15	0.53	119.31	0.92	0.23	256.52	0.94
3.6	0.3	138.41	2.61	0.22	498.26	1.1
5.42	0.41	114.00	2.71	0.21	617.88	0.5
4.01	0.25	162.81	2.02	0.13	652.88	0.84
2.34	0.33	101.19	1.39	0.20	236.79	0.37
4.96	0.51	131.05	2.00	0.21	650.00	0.55
4.03	0.67	64.86	1.09	0.18	261.41	0.66
2.44	0.49	101.45	1.18	0.24	247.54	0.98
3.55	0.24	270.56	1.97	0.13	960.47	0.78
6.05	0.5	202.48	2.50	0.21	1225.00	0.78
4.85	0.33	267.23	2.74	0.19	1296.07	0.88

4.12	0.35	150.24	2.01	0.17	619.00	2.1
2.23	0.33	146.81	1.19	0.18	327.38	0.93
2.91	0.2	156.91	1.61	0.11	456.60	0.7
2.21	0.21	233.96	2.08	0.20	517.06	1.04
3.5	0.22	123.94	2.46	0.15	433.80	0.48
3.12	0.31	123.12	1.80	0.18	384.14	0.51
2.5	0.66	132.89	1.10	0.29	332.24	0.62
3.33	0.34	256.84	3.51	0.36	855.28	0.66
1.68	0.24	122.34	0.85	0.12	205.52	0.72
1.95	0.38	174.32	1.32	0.26	339.93	0.78
3.13	0.2	163.58	1.93	0.12	512.01	0.96
3.21	0.65	208.40	2.70	0.55	668.97	0.99
2.85	0.36	84.19	1.13	0.14	239.94	1
5.06	0.6	179.08	3.31	0.39	906.17	1.1
2.81	0.51	95.53	1.14	0.21	268.43	1.2
3.48	0.52	132.76	2.00	0.30	462.00	1.23
3.06	0.61	107.37	1.41	0.28	328.56	1.36
6.08	0.23	288.81	4.54	0.17	1755.94	1.38
2.89	0.31	141.92	1.46	0.16	410.15	1.59
2.58	0.44	115.28	1.19	0.20	297.42	0.38
3.48	0.15	209.02	2.85	0.12	727.38	0.51
3.75	0.39	129.41	1.84	0.19	485.29	0.63
3.43	0.42	172.86	1.72	0.21	592.92	0.7
3.09	0.36	186.89	2.53	0.30	577.48	0.71
2.32	0.39	105.68	1.32	0.22	245.18	0.77
2.51	0.49	117.93	1.36	0.27	296.02	0.79
3.91	0.54	124.57	1.69	0.23	487.06	0.84
2.79	0.24	146.95	1.70	0.15	409.99	0.88
2.58	0.33	465.79	2.26	0.29	1201.74	0.98
2.4	0.32	109.19	1.30	0.17	262.05	1.01
2.93	0.6	113.64	1.66	0.34	332.95	1.18
4.43	0.26	471.29	4.39	0.26	2087.80	1.29
3.01	0.31	179.46	1.63	0.17	540.17	1.31
4.57	0.36	199.46	2.46	0.19	911.54	1.36
4.54	0.39	82.78	1.37	0.12	375.82	1.82
3.29	0.23	111.61	2.12	0.15	367.21	1.93
6.95	0.39	233.87	5.60	0.31	1625.40	2.04
3.97	0.34	138.89	2.45	0.21	551.39	2.05
6.99	0.75	442.17	8.42	0.90	3090.76	2.62
3.2	0.36	141.88	1.68	0.19	454.03	0.96
3.56	0.47	214.20	2.20	0.29	762.54	0.4
1.78	0.3	82.35	0.70	0.12	146.59	0.57
2.61	0.28	112.11	1.37	0.15	292.59	0.63
4.61	0.5	161.33	3.07	0.33	743.75	1.54
2.15	0.44	232.63	1.13	0.23	500.16	0.9
6.09	0.52	107.00	3.05	0.26	651.63	0.98
4.41	0.67	169.38	2.11	0.32	746.96	0.45
5.4	0.48	223.53	5.29	0.47	1207.06	0.47

2.17	0.3	147.43	1.24	0.17	319.92	0.49
3.31	0.28	168.32	1.64	0.14	557.13	0.5
6.48	0.46	150.34	4.35	0.31	974.17	0.52
2.31	0.35	142.20	1.34	0.20	328.47	0.54
2.35	0.38	139.81	1.09	0.18	328.56	0.59
2.9	0.52	117.70	1.19	0.21	341.32	0.61
3.37	0.28	167.50	2.11	0.18	564.48	0.63
1.71	0.15	614.10	2.19	0.19	1050.12	0.72
2.6	0.36	105.47	1.29	0.18	274.23	0.8
2.5	0.76	320.93	1.94	0.59	802.33	0.8
4.19	0.48	369.44	3.88	0.44	1547.97	0.82
2.63	0.3	127.12	1.49	0.17	334.32	0.83
1.92	0.29	186.99	1.56	0.24	359.02	0.85
2.08	0.22	216.55	1.43	0.15	450.43	0.87
4.02	0.25	210.31	4.14	0.26	845.44	0.89
4.92	0.4	85.71	2.81	0.23	421.71	0.99
3.32	0.43	134.46	2.24	0.29	446.41	1.04
3.03	0.33	111.84	1.33	0.14	338.88	1.1
2.93	0.18	124.79	1.21	0.07	365.64	1.17
3.81	0.36	120.17	1.60	0.15	457.84	1.26
2.29	0.24	498.55	3.32	0.35	1141.68	1.33
5.19	0.37	304.32	3.20	0.23	1579.43	1.46
6.55	0.37	186.93	4.28	0.24	1224.38	1.55
5.1	0.55	189.47	2.44	0.26	966.32	1.8
3.78	0.41	183.58	2.82	0.31	693.94	1.84
2.87	0.41	154.55	1.74	0.25	443.55	2.04
4.49	0.29	136.28	2.09	0.13	611.89	2.09
4.29	0.54	194.74	2.51	0.32	835.42	0.40
4.7	0.27	350.71	3.36	0.19	1648.36	0.41
1.6	0.2	226.47	1.57	0.20	362.35	0.48
2.65	0.33	193.96	1.78	0.22	513.99	0.48
3.19	0.37	171.43	1.52	0.18	546.86	0.51
4.28	0.39	120.28	1.97	0.18	514.78	0.57
2.85	0.41	204.37	1.38	0.20	582.45	0.57
1.55	0.26	109.01	1.40	0.23	168.96	0.59
2.21	0.35	178.92	1.33	0.21	395.40	0.64
3.68	0.32	322.08	4.78	0.42	1185.25	0.76
3.99	0.49	164.71	2.13	0.26	657.18	0.81
4.55	0.29	135.87	2.04	0.13	618.23	0.82
4.61	0.31	257.14	4.39	0.30	1185.43	0.84
3.1	0.37	168.33	1.72	0.21	521.83	0.86
3.64	0.51	91.94	1.73	0.24	334.67	0.95
1.8	0.3	245.61	1.58	0.26	442.11	0.96
3.71	0.38	172.28	3.67	0.38	639.15	1.01
2.99	0.32	178.42	2.15	0.23	533.47	1.08
2.17	0.42	208.06	1.17	0.23	451.50	1.12
7.11	0.3	457.14	6.77	0.29	3250.29	1.18
2.02	0.29	139.25	1.09	0.16	281.28	1.23

3.96	0.44	168.54	2.22	0.25	667.42	1.24
4.1	0.72	145.26	2.16	0.38	595.58	1.38
4.68	0.49	261.76	3.44	0.36	1225.06	1.53
6.34	0.62	472.45	6.47	0.63	2995.33	1.55
5.51	0.33	109.34	2.14	0.13	602.46	1.63
4.71	0.41	184.10	2.42	0.21	867.12	1.67
5.24	0.33	251.65	5.76	0.36	1318.64	1.71
4.78	0.7	155.40	2.24	0.33	742.81	1.99
3.7	0.23	139.61	1.45	0.09	516.55	2.14
6.57	0.91	179.51	3.20	0.44	1179.40	2.24
2.26	0.39	155.41	1.44	0.25	351.24	0.52
3.08	0.49	237.06	2.15	0.34	730.15	0.72
2.68	0.52	104.67	0.89	0.17	280.51	1.5
2.42	0.35	102.04	1.23	0.18	246.94	0.69
1.7	0.3	102.37	0.81	0.14	174.03	0.83
1.97	0.16	136.97	1.19	0.10	269.83	0.43
2.75	0.44	138.18	1.25	0.20	380.00	0.8
3	0.5	156.40	1.04	0.17	469.20	1.31
3.37	0.32	207.06	1.98	0.19	697.79	0.33
2.65	0.48	142.36	1.31	0.24	377.27	0.49
1.74	0.29	160.55	0.80	0.13	279.36	0.72
3.43	0.34	261.78	2.18	0.22	897.92	0.87
2.73	0.34	130.21	1.42	0.18	355.47	0.9
2.1	0.35	147.57	1.02	0.17	309.90	0.95
1.95	0.26	156.55	1.34	0.18	305.28	1.09
2.74	0.17	151.43	1.57	0.10	414.91	1.17
3.23	0.46	281.95	2.43	0.35	910.71	1.17
4.85	0.35	196.13	3.13	0.23	951.23	1.47
3.9	0.3	134.19	2.52	0.19	523.35	0.67
2.71	0.27	113.10	1.87	0.19	306.51	0.81
3.26	0.81	161.31	1.94	0.48	525.87	0.9
3.08	0.49	239.62	1.94	0.31	738.04	1.45
2.49	0.11	150.91	1.51	0.07	375.76	1.48
5.65	0.6	113.04	2.23	0.24	638.70	0.77
6.78	0.65	368.75	5.30	0.51	2500.13	2.39
4.63	0.65	140.07	1.67	0.23	648.53	1.78
10.06	0.59	259.52	11.98	0.70	2610.81	2.66
3.21	0.59	149.14	1.38	0.25	478.73	0.45
4.15	0.64	104.09	1.54	0.24	431.97	0.91
1.23	0.39	148.61	0.49	0.16	182.78	0.71
3.22	0.32	126.15	1.65	0.16	406.22	0.97
6.37	0.73	272.48	5.84	0.67	1735.68	0.97
2.95	0.26	141.89	1.33	0.12	418.58	1.29
4.86	0.63	148.97	3.35	0.43	723.97	1.69
5.49	0.63	125.00	2.64	0.30	686.25	3.76
4.66	0.5	117.33	1.55	0.17	546.77	0.43
3.48	0.28	95.65	1.51	0.12	332.87	1.38
2.44	0.41	266.96	2.18	0.37	651.39	1.4

4.85	0.52	175.27	2.61	0.28	850.05	1.54
2.71	0.47	156.19	1.29	0.22	423.28	0.43
3.06	0.61	88.44	1.36	0.27	270.64	0.48
2.57	0.34	112.73	1.17	0.15	289.71	0.59
3.66	0.31	319.35	2.95	0.25	1168.84	0.6
5	0.59	145.81	2.46	0.29	729.06	0.6
7.04	0.51	132.55	2.76	0.20	933.15	1.72
2.32	0.3	205.71	2.21	0.29	477.26	0.4
1.54	0.31	116.55	1.06	0.21	179.49	0.51
4.04	0.44	242.26	2.40	0.26	978.74	0.53
2.08	0.19	110.18	1.25	0.11	229.17	0.58
1.99	0.28	169.50	1.41	0.20	337.31	0.6
3.42	0.39	149.64	2.46	0.28	511.77	0.61
3.07	0.34	114.80	1.57	0.17	352.42	0.68
4.14	0.37	224.68	2.69	0.24	930.16	0.74
3.2	0.36	156.89	1.92	0.22	502.04	0.78
3.27	0.35	129.75	2.07	0.22	424.27	0.79
2	0.27	153.00	1.00	0.14	306.00	0.84
2.41	0.22	243.24	1.63	0.15	586.22	0.86
2.55	0.23	149.74	1.34	0.12	381.83	0.89
2.64	0.42	259.72	1.83	0.29	685.67	0.91
1.87	0.58	115.72	0.82	0.25	216.40	0.93
3.1	0.25	167.78	1.72	0.14	520.11	0.96
2.95	0.3	184.13	2.34	0.24	543.17	0.99
4.48	0.33	119.42	1.85	0.14	535.01	1
6.02	0.4	104.69	2.83	0.19	630.26	1.07
4.54	0.31	129.09	2.75	0.19	586.07	1.09
2.06	0.16	220.80	1.65	0.13	454.85	1.19
3.19	0.11	316.00	6.38	0.22	1008.04	1.22
3.04	0.42	74.54	0.93	0.13	226.60	1.28
4.42	0.3	148.52	2.62	0.18	656.46	1.32
3.87	0.29	102.65	2.05	0.15	397.24	1.33
2.56	0.4	150.94	1.21	0.19	386.42	1.38
2.68	0.49	107.59	1.13	0.21	288.35	1.39
3.38	0.39	222.45	2.30	0.27	751.88	1.41
2.98	0.33	126.86	1.70	0.19	378.03	1.46
4.07	0.46	183.55	2.68	0.30	747.06	1.46
2.82	0.25	122.88	1.84	0.16	346.51	1.48
4.21	0.41	127.90	1.81	0.18	538.45	1.54
1.58	0.17	262.22	1.76	0.19	414.31	1.72
3.81	0.30	292.42	2.89	0.23	1114.14	1.81
7.63	0.44	605.66	14.40	0.83	4621.19	1.96
5.57	0.44	190.34	3.16	0.25	1060.20	1.99
4.97	0.51	158.82	3.65	0.38	789.35	2.08
11.91	0.63	72.73	15.47	0.82	866.18	3.69
3.4	0.61	132.81	1.77	0.32	451.56	0.32

3.47	0.36	117.36	1.31	0.14	407.23	0.42
2.35	0.37	174.45	1.72	0.27	409.96	0.45
2.27	0.36	213.29	1.44	0.23	484.17	0.46
3.07	0.34	285.58	2.95	0.33	876.72	0.48
4.19	0.28	163.36	3.20	0.21	684.47	0.52
3.52	0.44	96.21	1.33	0.17	338.67	0.55
3.19	0.22	138.60	1.87	0.13	442.12	0.57
2.8	0.29	146.94	1.90	0.20	411.43	0.6
2	0.22	97.09	0.97	0.11	194.17	0.63
1.95	0.37	170.05	0.99	0.19	331.60	0.64
2.41	0.34	165.60	1.93	0.27	399.10	0.64
2.14	0.27	92.66	0.98	0.12	198.29	0.66
5.39	0.58	183.79	2.13	0.23	990.65	0.66
2.55	0.38	171.77	1.22	0.18	438.01	0.67
2.42	0.47	134.15	0.98	0.19	324.63	0.69
2.24	0.19	149.01	1.48	0.13	333.77	0.71
3.42	0.43	134.62	1.32	0.17	460.38	0.75
1.52	0.20	231.65	1.09	0.14	352.12	0.76
2.41	0.40	213.39	2.15	0.36	514.28	0.78
3.86	0.38	197.21	2.16	0.21	761.22	0.8
2.93	0.56	174.56	1.02	0.20	511.47	0.81
1.73	0.36	104.28	0.67	0.14	180.40	0.82
3.27	0.61	186.06	1.98	0.37	608.42	0.82
3.88	0.28	361.61	3.46	0.25	1403.04	0.89
5.91	0.1	527.50	14.78	0.25	3117.53	0.93
8.75	0.33	163.70	5.99	0.23	1432.36	0.95
3.38	0.17	259.18	2.30	0.12	876.04	0.98
2.39	0.29	237.01	1.88	0.23	566.45	0.99
4.32	0.3	140.65	3.51	0.24	607.61	1
1.98	0.27	143.26	1.11	0.15	283.65	1.03
3.47	0.33	321.26	2.73	0.26	1114.77	1.03
5.84	0.05	145.10	5.73	0.05	847.37	1.04
3.25	0.22	177.66	1.73	0.12	577.39	1.11
5.28	0.32	125.60	2.55	0.15	663.19	1.11
2.8	0.28	189.76	2.20	0.22	531.34	1.12
1.72	0.27	133.70	0.95	0.15	229.97	1.15
1.97	0.3	84.90	1.03	0.16	167.24	1.17
4.36	0.58	185.71	1.95	0.26	809.71	1.17
4.49	0.42	100.00	1.64	0.15	449.00	1.18
2.86	0.29	286.21	2.47	0.25	818.55	1.22
4.17	0.37	127.27	1.58	0.14	530.73	1.23
2.15	0.29	38.57	0.96	0.13	82.91	1.25
3.6	0.26	204.09	2.11	0.15	734.74	1.25
3.68	0.29	150.28	2.06	0.16	553.03	1.26
3.80	0.37	201.36	1.73	0.17	765.18	1.32
2.11	0.33	99.18	0.86	0.14	209.27	1.34

4.89	0.34	113.82	1.61	0.11	556.56	1.34
5.68	0.64	209.74	3.69	0.42	1191.32	1.34
2.89	0.33	120.53	1.91	0.22	348.33	1.37
6.73	0.4	237.27	4.18	0.25	1596.81	1.42
1.83	0.33	221.95	1.49	0.27	406.17	1.43
3.45	0.45	130.30	1.74	0.23	449.55	1.49
6.06	0.69	223.36	5.66	0.64	1353.59	1.65
2.27	0.26	165.12	1.06	0.12	374.81	1.81
3.76	0.43	97.54	1.32	0.15	366.76	1.92
4.24	0.53	159.63	2.63	0.33	676.82	2.23
5.21	0.52	163.59	2.83	0.28	852.29	2.49
8.51	0.49	452.00	11.35	0.65	3846.52	3.33
3.51	0.33	258.97	3.00	0.28	909.00	0.25
1.9	0.52	118.91	0.95	0.26	225.92	0.33
2.31	0.35	200.00	1.38	0.21	462.00	0.37
2.77	0.42	125.40	1.47	0.22	347.35	0.42
1.91	0.25	291.22	1.29	0.17	556.22	0.42
4.9	0.37	215.68	2.65	0.20	1056.81	0.43
2.89	0.16	126.90	1.47	0.08	366.75	0.44
2.97	0.25	265.22	2.58	0.22	787.70	0.44
2.4	0.3	184.97	1.39	0.17	443.93	0.48
4.52	0.38	240.98	3.70	0.31	1089.25	0.48
3.73	0.45	113.79	1.84	0.22	424.45	0.51
3.29	0.27	224.76	3.13	0.26	739.47	0.54
5.32	0.18	404.49	5.98	0.20	2151.91	0.55
2.11	0.34	198.72	2.71	0.44	419.29	0.56
5.31	0.39	85.77	2.10	0.15	455.44	0.57
3.01	0.39	98.70	1.31	0.17	297.07	0.58
2.22	0.37	135.00	1.39	0.23	299.70	0.58
2.71	0.29	130.43	1.68	0.18	353.48	0.58
3.06	0.46	146.07	1.60	0.24	446.98	0.6
3.59	0.26	221.19	3.04	0.22	794.06	0.62
4.89	0.28	152.24	2.43	0.14	744.45	0.63
2.45	0.37	126.67	1.36	0.21	310.33	0.64
2.47	0.21	170.05	1.32	0.11	420.03	0.64
3.3	0.34	123.81	2.62	0.27	408.57	0.66
2.12	0.35	132.04	2.06	0.34	279.92	0.67
2.2	0.35	98.58	1.56	0.25	216.88	0.68
2.82	0.36	229.70	1.71	0.22	647.75	0.69
1.48	0.23	186.24	1.36	0.21	275.63	0.7
3.63	0.62	120.00	1.54	0.26	435.60	0.7
1.98	0.21	164.57	0.89	0.09	325.86	0.71
3.2	0.32	108.49	1.51	0.15	347.17	0.73
3	0.23	236.19	2.86	0.22	708.57	0.73
3.44	0.48	114.94	1.43	0.20	395.39	0.74
3.51	0.3	232.14	4.18	0.36	814.82	0.74
2.47	0.21	229.69	1.93	0.16	567.33	0.76
2.86	0.32	105.02	1.31	0.15	300.37	0.78

1.94	0.31	158.06	1.25	0.20	306.65	0.78
2.53	0.37	250.00	1.66	0.24	632.50	0.78
3.61	0.29	153.13	2.26	0.18	552.78	0.79
4.57	0.73	129.15	2.30	0.37	590.20	0.79
2.98	0.44	125.63	1.86	0.28	374.36	0.8
2.41	0.58	193.33	1.46	0.35	465.93	0.8
4.86	0.46	115.81	1.79	0.17	562.83	0.8
2.32	0.28	153.50	1.16	0.14	356.12	0.81
2.19	0.51	280.70	1.92	0.45	614.74	0.82
2.61	0.31	206.47	1.88	0.22	538.90	0.83
3.64	0.49	161.11	1.84	0.25	586.44	0.84
3.77	0.27	209.04	2.13	0.15	788.08	0.84
5.99	0.48	218.83	3.89	0.31	1310.80	0.84
2.05	0.31	116.53	0.85	0.13	238.88	0.85
3.28	0.4	118.58	1.79	0.22	388.94	0.86
4.12	0.24	193.18	1.87	0.11	795.91	0.86
2.75	0.43	112.73	1.67	0.26	310.00	0.87
3.43	0.36	134.97	1.87	0.20	462.96	0.87
2.57	0.36	187.37	1.30	0.18	481.55	0.87
3.15	0.34	132.66	1.58	0.17	417.89	0.88
2	0.32	282.35	1.68	0.27	564.71	0.88
3.84	0.24	263.38	2.70	0.17	1011.38	0.89
2.15	0.19	122.14	0.82	0.07	262.60	0.94
1.71	0.26	122.29	0.98	0.15	209.11	0.96
1.77	0.36	166.96	1.58	0.32	295.53	0.96
3.57	0.25	152.17	1.94	0.14	543.26	0.97
2.28	0.33	311.27	1.61	0.23	709.69	0.97
4.13	0.64	139.49	2.12	0.33	576.08	0.98
3.41	0.47	135.64	1.81	0.25	462.53	0.99
1.19	0.49	193.46	1.11	0.46	230.21	1.01
3.41	0.4	98.92	1.84	0.22	337.31	1.01
3.4	0.37	108.33	1.29	0.14	368.33	1.01
1.11	0.25	87.65	0.69	0.15	97.30	1.02
2.69	0.48	151.14	1.53	0.27	406.56	1.02
3.63	0.48	126.28	2.65	0.35	458.39	1.02
3.49	0.35	96.83	1.23	0.12	337.94	1.03
2.22	0.32	89.81	1.08	0.16	199.37	1.05
3.68	0.22	258.99	2.65	0.16	953.09	1.05
5.73	0.51	256.32	3.02	0.27	1468.69	1.07
2.66	0.2	168.24	1.56	0.12	447.51	1.09
4.22	0.43	135.38	3.25	0.33	571.32	1.1
3.15	0.78	188.13	1.97	0.49	592.59	1.12
4.09	0.31	152.71	3.17	0.24	624.60	1.12
10.03	0.74	205.11	7.32	0.54	2057.25	1.13
5.38	0.49	115.04	2.19	0.20	618.92	1.16
3.31	0.41	107.69	1.50	0.19	356.46	1.17
3.51	0.64	204.53	1.32	0.24	717.89	1.18
2.86	0.44	91.35	1.38	0.21	261.25	1.19

4.09	0.42	136.00	2.05	0.21	556.24	1.2
5.66	0.39	203.88	4.39	0.30	1153.94	1.2
1.55	0.18	172.66	1.12	0.13	267.63	1.25
6.35	0.65	130.00	2.76	0.28	825.50	1.26
5.1	0.26	231.58	3.83	0.20	1181.05	1.3
4.6	0.54	154.00	3.07	0.36	708.40	1.33
3.72	0.55	165.75	2.55	0.38	616.60	1.35
2.55	0.55	115.16	1.05	0.23	293.67	1.39
2.52	0.3	184.18	1.42	0.17	464.14	1.39
6.02	0.87	72.50	2.15	0.31	436.45	1.4
3.46	0.31	246.56	2.64	0.24	853.11	1.42
6.48	0.91	202.06	3.34	0.47	1309.36	1.44
4.99	0.26	213.74	3.81	0.20	1066.56	1.45
4	0.48	128.85	1.92	0.23	515.38	1.48
3.47	0.35	176.04	1.81	0.18	610.86	1.48
3.57	0.37	118.97	1.15	0.12	424.73	1.51
6.03	0.2	145.55	3.16	0.10	877.66	1.51
3.93	0.41	128.65	2.05	0.21	505.58	1.52
4.92	0.51	177.37	3.59	0.37	872.67	1.52
4.3	0.43	113.74	2.04	0.20	489.10	1.54
5.05	0.52	299.28	3.66	0.38	1511.34	1.57
2.49	0.42	142.02	1.05	0.18	353.62	1.6
3.22	0.52	151.06	1.71	0.28	486.43	1.62
5.84	0.33	162.50	4.29	0.24	949.00	1.68
4.25	0.72	322.29	2.71	0.46	1369.75	1.69
2.68	0.46	97.52	1.11	0.19	261.36	1.73
3.33	0.42	210.90	2.13	0.27	702.29	1.73
3.61	0.51	133.65	1.71	0.24	482.47	1.79
4.49	0.27	114.35	2.01	0.12	513.43	1.79
3.47	0.6	137.35	1.39	0.24	476.60	1.8
6.68	0.28	297.65	7.86	0.33	1988.28	1.9
2.9	0.28	131.16	1.46	0.14	380.35	1.97
4.51	0.32	204.42	2.49	0.18	921.93	2.05
4.67	0.54	88.85	1.63	0.19	414.93	2.27
5.38	0.77	158.64	3.32	0.48	853.49	2.43
3.92	0.53	90.09	1.69	0.23	353.14	2.49
4.43	0.33	194.12	2.61	0.19	859.94	2.74
7.16	0.48	97.27	2.80	0.19	696.42	2.79
4.96	0.42	226.92	4.77	0.40	1125.54	2.96
10.81	0.82	171.90	7.07	0.54	1858.19	3.48
5.32	0.39	86.45	1.60	0.12	459.89	3.7
3.41	0.32	275.51	2.32	0.22	939.49	4.15
5.67	0.49	126.28	2.07	0.18	715.99	5.07
2.23	0.25	101.74	1.30	0.15	226.89	0.29
2.5	0.3	230.53	1.91	0.23	576.34	0.33
2.64	0.4	135.23	1.50	0.23	357.00	0.38
3.76	0.51	160.38	2.36	0.32	603.02	0.41
2.88	0.36	303.36	2.42	0.30	873.68	0.41

3.31	0.21	182.47	1.71	0.11	603.99	0.42
3.43	0.3	203.03	2.08	0.18	696.39	0.42
2.2	0.31	240.29	1.58	0.22	528.63	0.44
2.68	0.42	139.29	1.37	0.21	373.29	0.45
2.95	0.42	127.67	1.43	0.20	376.63	0.45
3.11	0.23	193.72	1.30	0.10	602.48	0.45
2.74	0.34	174.68	1.78	0.22	478.61	0.48
4.38	0.29	236.27	4.29	0.28	1034.88	0.48
2.66	0.33	128.32	1.54	0.19	341.34	0.49
2.69	0.19	170.92	1.91	0.13	459.78	0.49
3	0.3	188.03	2.56	0.26	564.10	0.49
4.48	0.25	197.39	2.93	0.16	884.29	0.49
1.95	0.34	202.73	1.07	0.19	395.33	0.5
2.2	0.41	108.13	1.05	0.20	237.89	0.51
3	0.24	273.38	2.16	0.17	820.14	0.51
3.39	0.26	293.97	2.92	0.22	996.54	0.51
4.75	0.35	94.81	3.52	0.26	450.37	0.52
4.13	0.07	506.45	6.66	0.11	2091.65	0.52
2.97	0.22	146.67	1.41	0.10	435.60	0.53
3.25	0.48	139.13	1.41	0.21	452.17	0.53
3.51	0.5	143.62	1.87	0.27	504.10	0.53
2.02	0.47	122.05	1.59	0.37	246.54	0.54
1.96	0.36	140.94	0.77	0.14	276.25	0.54
2.81	0.34	145.06	1.73	0.21	407.62	0.54
2.84	0.35	170.32	1.83	0.23	483.72	0.54
3.18	0.38	216.30	2.36	0.28	687.82	0.54
4.85	0.34	143.79	2.87	0.20	697.37	0.55
4.68	0.46	234.93	2.04	0.20	1099.49	0.556
2.8	0.27	130.23	1.30	0.13	364.65	0.56
2.58	0.51	92.11	1.13	0.22	237.63	0.58
2.55	0.46	133.54	1.61	0.29	340.54	0.58
2.04	0.4	200.00	1.04	0.20	408.00	0.58
2.5	0.3	228.35	1.29	0.15	570.88	0.58
3.72	0.25	233.74	2.28	0.15	869.52	0.58
4.17	0.29	231.97	3.42	0.24	967.30	0.58
1.82	0.34	263.08	1.40	0.26	478.80	0.59
5.41	0.41	228.50	2.80	0.21	1236.17	0.59
3.19	0.53	126.32	1.53	0.25	402.95	0.6
1.94	0.31	212.06	1.38	0.22	411.39	0.6
3.65	0.39	158.38	1.97	0.21	578.08	0.6
1.7	0.23	231.79	1.13	0.15	394.04	0.61
3.77	0.62	129.86	1.71	0.28	489.59	0.61
2.1	0.23	311.61	1.35	0.15	654.39	0.61
2.23	0.21	136.31	1.25	0.12	303.98	0.62
2.12	0.38	187.07	1.83	0.33	396.59	0.62
3.67	0.4	121.63	1.30	0.14	446.39	0.62
1.08	0.23	141.61	0.79	0.17	152.93	0.63
1.61	0.44	115.12	0.79	0.21	185.35	0.63

2.24	0.4	152.63	0.98	0.18	341.89	0.63
2.17	0.32	226.32	3.81	0.56	491.11	0.63
1.63	0.29	165.73	1.14	0.20	270.15	0.64
2.59	0.47	114.91	1.14	0.21	297.62	0.65
2.58	0.26	169.33	1.58	0.16	436.86	0.65
2.48	0.4	108.57	1.18	0.19	269.26	0.66
2.17	0.4	125.48	1.38	0.25	272.29	0.66
5.11	0.67	292.00	3.41	0.45	1492.12	0.66
2.41	0.28	114.76	1.15	0.13	276.58	0.67
2.59	0.45	118.29	1.48	0.26	306.36	0.67
2.27	0.38	184.72	1.58	0.26	419.32	0.67
3.88	0.34	353.70	7.19	0.63	1372.37	0.67
2.69	0.26	150.00	1.37	0.13	403.50	0.68
1.74	0.3	350.00	1.43	0.25	609.00	0.68
2.95	0.39	84.25	1.01	0.13	248.53	0.69
2.72	0.41	54.36	1.13	0.17	147.85	0.7
2.72	0.18	310.96	1.86	0.12	845.81	0.7
2.51	0.3	125.00	1.90	0.23	313.75	0.71
3.25	0.58	117.09	1.63	0.29	380.53	0.71
2.87	0.5	210.81	1.94	0.34	605.03	0.71
3.65	0.3	140.15	2.66	0.22	511.53	0.72
2.65	0.29	191.49	1.88	0.21	507.45	0.73
2.42	0.35	109.56	0.83	0.12	265.13	0.74
2.97	0.25	181.55	1.77	0.15	539.20	0.74
1.9	0.27	138.50	0.95	0.14	263.15	0.75
2.69	0.47	104.88	1.09	0.19	282.12	0.75
4.28	0.25	222.13	3.51	0.20	950.72	0.75
5.78	0.52	191.79	4.31	0.39	1108.55	0.76
2.88	0.32	203.27	1.88	0.21	585.41	0.77
3.82	0.62	153.61	2.30	0.37	586.81	0.78
4.37	0.51	209.09	4.97	0.58	913.73	0.79
2.13	0.29	127.75	1.23	0.17	272.10	0.8
1.92	0.33	162.00	1.28	0.22	311.04	0.8
3.83	0.63	93.17	1.38	0.23	356.82	0.8
1.9	0.18	237.72	1.67	0.16	451.67	0.8
3.09	0.41	130.41	1.59	0.21	402.97	0.81
2.83	0.58	85.44	1.08	0.22	241.80	0.82
2.59	0.32	155.73	1.35	0.17	403.34	0.82
2.63	0.4	171.52	1.59	0.24	451.08	0.82
3.93	0.42	118.97	1.69	0.18	467.53	0.82
2.06	0.57	252.86	1.47	0.41	520.89	0.82
3.83	0.28	162.94	1.94	0.14	624.08	0.82
4.08	0.49	159.09	3.09	0.37	649.09	0.82
8.03	0.25	398.70	10.43	0.32	3201.57	0.82
2.54	0.34	267.81	1.74	0.23	680.23	0.83
3.72	0.64	191.56	2.42	0.42	712.60	0.83
3.1	0.31	308.05	1.78	0.18	954.94	0.83
2.54	0.53	119.46	1.15	0.24	303.42	0.84

4.01	0.41	125.98	1.97	0.20	505.18	0.84
2.77	0.47	62.75	0.91	0.15	173.80	0.85
2.98	0.32	196.24	2.24	0.24	584.80	0.85
1.78	0.27	123.04	0.77	0.12	219.02	0.86
2.51	0.24	115.68	1.36	0.13	290.35	0.86
3.83	0.4	246.76	2.76	0.29	945.10	0.86
5.96	0.27	160.78	3.90	0.18	958.27	0.86
4.04	0.35	317.82	4.00	0.35	1284.00	0.86
3.38	0.3	102.29	1.29	0.11	345.74	0.87
2.63	0.28	186.71	1.66	0.18	491.04	0.88
3.77	0.43	132.38	1.55	0.18	499.06	0.88
1.6	0.27	164.03	1.15	0.19	262.45	0.89
3.44	0.31	174.65	2.42	0.22	600.79	0.91
4.23	0.66	253.54	2.14	0.33	1072.45	0.91
1.69	0.28	157.96	1.08	0.18	266.96	0.92
2.48	0.24	123.00	1.24	0.12	305.04	0.92
2.81	0.47	125.29	1.61	0.27	352.06	0.92
2.98	0.46	139.44	1.66	0.26	415.54	0.92
3.87	0.52	126.63	2.10	0.28	490.06	0.92
2.04	0.33	265.56	1.35	0.22	541.75	0.92
3.27	0.33	178.33	1.82	0.18	583.15	0.92
3.99	0.33	163.74	2.33	0.19	653.33	0.92
3.78	0.45	225.00	3.38	0.40	850.50	0.92
1.79	0.19	231.31	1.81	0.19	414.05	0.93
1.77	0.38	168.63	1.16	0.25	298.47	0.94
2.64	0.73	180.77	1.45	0.40	477.23	0.94
3.84	0.37	143.97	3.31	0.32	552.83	0.94
5.83	0.46	238.89	5.40	0.43	1392.72	0.95
2.95	0.25	104.12	1.52	0.13	307.16	0.96
3.64	0.51	156.85	1.47	0.21	570.95	0.96
3.8	0.36	167.70	2.36	0.22	637.27	0.96
2.03	0.47	325.56	2.26	0.52	660.88	0.96
1.76	0.24	188.10	1.40	0.19	331.05	0.97
2.3	0.23	166.08	1.35	0.13	381.99	0.97
6.12	0.31	95.45	3.97	0.20	584.18	0.97
4.82	0.45	195.83	2.87	0.27	943.92	0.97
3.02	0.39	192.97	1.63	0.21	582.78	0.98
3.18	0.44	196.67	2.12	0.29	625.40	0.98
4.21	0.24	155.35	1.96	0.11	654.02	0.98
4.85	0.43	149.52	2.31	0.20	725.19	0.98
1.03	0.45	324.73	1.11	0.48	334.47	0.99
4.93	0.38	69.77	1.24	0.10	343.98	0.99
4.49	0.27	168.33	2.03	0.12	755.78	0.99
3.84	0.32	123.71	1.98	0.16	475.05	1
2.53	0.3	239.00	2.53	0.30	604.67	1
2.57	0.18	370.59	3.02	0.21	952.41	1
4.3	0.42	83.13	1.73	0.17	357.47	1.01
5.47	0.34	108.52	1.79	0.11	593.63	1.01

3.72	0.48	180.92	2.15	0.28	673.04	1.01
2.55	0.38	104.55	1.16	0.17	266.59	1.02
2.9	0.3	143.07	1.44	0.15	414.90	1.02
5.06	0.41	181.43	3.61	0.29	918.03	1.02
3.17	0.38	314.29	3.77	0.45	996.29	1.02
3.03	0.32	142.37	1.71	0.18	431.39	1.03
3.09	0.5	184.25	2.12	0.34	569.32	1.03
2.44	0.47	97.26	1.11	0.21	237.32	1.04
2.83	0.17	200.65	1.85	0.11	567.85	1.04
4.65	0.62	259.17	3.88	0.52	1205.13	1.04
3.73	0.4	147.60	1.63	0.17	550.54	1.05
2.41	0.45	235.56	2.68	0.50	567.69	1.05
3.97	0.32	212.88	3.01	0.24	845.13	1.05
2.87	0.43	103.57	1.46	0.22	297.25	1.06
5.66	0.26	121.93	3.03	0.14	690.10	1.06
5.08	0.54	121.83	2.02	0.21	618.87	1.07
3.69	0.86	180.63	1.93	0.45	666.52	1.07
2.46	0.29	127.47	1.35	0.16	313.58	1.08
3.64	0.36	144.64	1.56	0.15	526.47	1.08
2.47	0.5	237.30	1.34	0.27	586.12	1.09
3.33	0.25	218.52	2.06	0.15	727.67	1.09
2.71	0.45	155.29	1.59	0.26	420.85	1.1
5.63	0.6	138.25	1.98	0.21	778.32	1.11
3.58	0.41	276.79	2.13	0.24	990.89	1.13
2.13	0.48	71.75	0.96	0.22	152.83	1.14
2.22	0.36	123.53	1.45	0.24	274.24	1.14
2.63	0.36	146.99	1.44	0.20	386.60	1.14
2.73	0.35	185.81	1.84	0.24	507.26	1.14
3.48	0.29	152.71	1.71	0.14	531.43	1.14
3.85	0.27	227.56	2.47	0.17	876.12	1.14
4.35	0.38	188.89	2.69	0.23	821.67	1.15
2.4	0.22	349.61	1.86	0.17	839.07	1.16
4.48	0.55	112.31	2.30	0.28	503.14	1.17
2.72	0.37	185.71	1.77	0.24	505.14	1.17
4.1	0.34	140.56	1.65	0.14	576.31	1.18
2.83	0.19	254.55	2.86	0.19	720.36	1.18
3.75	0.43	215.57	3.07	0.35	808.40	1.18
1.96	0.5	105.30	1.30	0.33	206.38	1.2
2.76	0.44	122.90	1.05	0.17	339.21	1.2
4.09	0.53	173.91	1.98	0.26	711.30	1.2
2.98	0.26	300.80	2.38	0.21	896.38	1.2
4.41	0.35	91.78	3.02	0.24	404.75	1.21
2.52	0.33	179.21	1.25	0.16	451.60	1.21
3.48	0.24	286.49	2.35	0.16	996.97	1.21
3.58	0.27	208.05	2.40	0.18	744.83	1.22
2.7	0.53	107.14	0.96	0.19	289.29	1.23
5.37	0.44	129.86	2.43	0.20	697.37	1.23
3.89	0.47	119.63	1.44	0.17	465.36	1.24

2.94	0.34	224.84	1.83	0.21	661.04	1.24
3	0.25	97.22	1.39	0.12	291.67	1.25
2.47	0.27	215.09	1.55	0.17	531.28	1.25
3.94	0.2	195.54	2.51	0.13	770.43	1.25
2.53	0.67	118.47	1.02	0.27	299.74	1.27
4.05	0.66	289.17	2.58	0.42	1171.15	1.27
3.63	0.53	117.07	1.77	0.26	424.98	1.28
4.25	0.4	243.75	4.43	0.42	1035.94	1.28
4.56	0.45	243.51	2.96	0.29	1110.39	1.28
5.11	0.23	196.72	2.79	0.13	1005.25	1.29
3.96	0.5	204.03	3.19	0.40	807.97	1.31
2.86	0.25	173.48	1.58	0.14	496.15	1.32
3.29	0.26	125.81	1.52	0.12	413.90	1.34
3.19	0.29	210.91	1.93	0.18	672.80	1.34
2.75	0.51	142.77	1.59	0.29	392.63	1.35
3.86	0.36	224.42	2.24	0.21	866.26	1.35
3.83	0.44	115.35	1.78	0.20	441.79	1.37
2.92	0.44	150.00	1.33	0.20	438.00	1.38
7.35	0.46	187.50	3.99	0.25	1378.13	1.38
2.32	0.26	177.97	1.97	0.22	412.88	1.39
5.03	0.24	182.40	4.02	0.19	917.47	1.39
3.14	0.45	293.75	2.18	0.31	922.38	1.39
3.26	0.36	116.59	1.55	0.17	380.08	1.4
3.18	0.65	157.30	1.79	0.37	500.22	1.4
3.32	0.38	201.04	1.73	0.20	667.46	1.41
2.81	0.48	107.67	0.90	0.15	302.55	1.43
4.14	0.52	198.37	1.69	0.21	821.24	1.44
3.58	0.23	304.59	3.28	0.21	1090.42	1.45
7.96	0.41	180.59	4.68	0.24	1437.48	1.45
4.04	0.54	82.65	1.19	0.16	333.89	1.47
2.76	0.36	88.83	1.34	0.17	245.18	1.48
3.79	0.35	100.00	1.83	0.17	379.00	1.48
3.63	0.67	111.81	1.43	0.26	405.87	1.48
4.53	0.41	56.70	2.02	0.18	256.83	1.49
3.54	0.63	130.43	1.54	0.27	461.74	1.49
5.57	0.42	111.24	2.09	0.16	619.58	1.49
4.13	0.49	126.34	1.84	0.22	521.78	1.51
4.47	0.47	139.88	2.66	0.28	625.27	1.52
4.39	0.41	144.30	2.95	0.28	633.46	1.54
3.62	0.26	204.98	1.64	0.12	742.02	1.54
2.95	0.6	272.53	3.24	0.66	803.96	1.55
4.54	0.45	206.73	2.18	0.22	938.56	1.55
2.1	0.33	88.80	1.68	0.26	186.48	1.57
2.43	0.32	121.78	1.20	0.16	295.93	1.57
4.7	0.58	97.57	1.43	0.18	458.57	1.58
3.25	0.44	171.68	2.88	0.39	557.96	1.6
5.86	0.75	212.33	4.01	0.51	1244.25	1.6
6.41	0.41	562.26	12.09	0.77	3604.11	1.6

0.88	0.5	140.10	0.46	0.26	123.29	1.61
1.79	0.34	69.29	1.41	0.27	124.03	1.61
3.33	0.31	252.45	2.33	0.22	840.65	1.61
6.32	0.3	217.05	4.90	0.23	1371.78	1.64
3.21	0.33	205.91	1.73	0.18	660.98	1.66
5.55	0.44	172.43	2.28	0.18	956.98	1.67
4.51	0.27	161.61	2.01	0.12	728.85	1.69
7.09	0.73	128.83	3.19	0.33	913.40	1.69
3.94	0.35	82.40	1.16	0.10	324.67	1.71
3.47	0.46	220.75	2.18	0.29	766.02	1.71
3.99	0.31	232.08	2.51	0.19	925.98	1.74
3.51	0.34	111.81	1.48	0.14	392.47	1.77
3.07	0.38	137.72	1.84	0.23	422.81	1.78
4.44	0.32	191.46	2.71	0.20	850.10	1.79
4.8	0.39	174.06	2.01	0.16	835.48	1.8
2.35	0.26	218.12	1.58	0.17	512.58	1.83
5.81	0.29	162.01	3.25	0.16	941.28	1.83
3.87	0.35	89.89	2.17	0.20	347.87	1.84
2.96	0.37	79.18	1.10	0.14	234.38	1.85
3.26	0.45	61.77	1.00	0.14	201.38	1.88
4.64	0.27	183.44	2.96	0.17	851.16	1.88
6.23	0.26	325.35	8.77	0.37	2026.94	1.89
5.03	0.39	167.26	2.99	0.23	841.33	1.93
4.92	0.59	318.59	3.15	0.38	1567.46	1.95
3.59	0.44	201.46	2.62	0.32	723.24	2.03
3.56	0.25	204.32	2.20	0.15	727.38	2.03
3.24	0.27	112.85	0.91	0.08	365.63	2.04
7.86	0.55	171.90	3.74	0.26	1351.17	2.04
3.79	0.52	97.08	2.77	0.38	367.93	2.06
4.85	0.27	161.72	3.79	0.21	784.34	2.11
4.33	0.42	212.94	2.55	0.25	922.04	2.11
4.99	0.5	136.65	2.61	0.26	681.88	2.14
4.37	0.47	133.88	1.78	0.19	585.04	2.2
3.79	0.36	131.33	2.28	0.22	497.72	2.21
4.57	0.42	159.82	2.09	0.19	730.37	2.23
3.17	0.32	114.65	1.60	0.16	363.43	2.25
2	0.31	109.69	0.63	0.10	219.38	2.31
2.08	0.28	263.12	1.48	0.20	547.29	2.39
4.5	0.38	205.38	2.42	0.20	924.19	2.4
5.88	0.65	128.47	2.15	0.24	755.39	2.47
4.66	0.37	168.83	1.89	0.15	786.73	2.47
7.47	0.53	159.26	3.46	0.25	1189.67	2.59
4.37	0.35	170.00	1.99	0.16	742.90	2.6
5.59	0.56	335.51	5.22	0.52	1875.52	2.75
4.14	0.29	102.15	1.78	0.12	422.88	2.88
7.57	0.76	198.68	4.98	0.50	1504.04	2.94
7.6	0.44	118.84	2.75	0.16	903.19	3.03
4.47	0.39	127.69	1.85	0.16	570.76	3.05

4.27	0.49	184.06	2.06	0.24	785.93	3.09
4.9	0.41	94.10	1.61	0.13	461.08	3.27
7.24	0.66	138.60	2.66	0.24	1003.49	4.13
6.39	0.33	128.81	2.63	0.14	823.07	4.29
2.6	0.37	126.45	0.94	0.13	328.77	5.46
9.11	0.61	157.80	3.23	0.22	1437.57	5.82
8.76	0.68	186.23	6.35	0.49	1631.39	5.91

TC	HDL	LDL	VLDL	Lipoprotein
3.79	1.3	2.07	0.42	104
5.18	1.56	2.91	0.71	333
4.82	1.47	3.2	0.15	35
4.34	1.17	3.15	0.02	219
5.28	1.56	3.32	0.4	72
3.64	1.36	1.56	0.72	257
5.28	1.59	3.61	0.08	26
3.7	1.42	1.97	0.31	128
4.21	1.2	2.82	0.19	125
5.14	1.77	2.92	0.45	169
5.07	1.75	2.86	0.46	126
4.59	1.25	2.7	0.64	479
3.21	1.15	2.02	0.04	308
4.34	1.94	2.05	0.35	87
4.94	1.72	2.72	0.5	376
3.72	1.6	1.91	0.21	128
7.12	1.76	4.7	0.66	112
4.18	1.37	2.46	0.35	447
4.04	0.93	2.64	0.47	18
3.3	0.79	2.31	0.2	187
5.22	1.85	3.1	0.27	465
3.43	1.16	2	0.27	107
4.18	1	2.58	0.6	103
3.15	0.79	2.21	0.15	131
4.3	1.7	2.16	0.44	833
4.32	1.07	2.78	0.47	3
4.51	1.63	2.4	0.48	233
4.68	1.78	2.37	0.53	175
4.65	1.98	2.5	0.17	30
4.42	1.57	2.45	0.4	186
4.98	1.57	3.09	0.32	472
4.64	1.38	2.87	0.39	95
3.93	1.13	2.67	0.13	639
3.69	1.3	1.97	0.42	357
5.35	1.43	3.59	0.33	79
4.2	0.86	2.54	0.8	193
4.92	1.58	2.93	0.41	231
3.44	1.09	2.06	0.29	42
3.71	1.39	1.96	0.36	149
4.68	1.24	3.17	0.27	240
5.2	1.66	3.12	0.42	795
4.39	1.22	2.69	0.48	292
5.03	1.13	3.53	0.37	363
4.64	1.18	3.23	0.23	361
5.58	1.28	3.81	0.49	347
4.35	0.9	2.51	0.94	946
5.76	1.62	3.61	0.53	90

4.17	1.28	2.43	0.46	341
3.79	1.73	1.88	0.18	94
3.46	1.49	1.84	0.13	105
4.16	1.17	2.61	0.38	159
5.04	1.38	3.29	0.37	106
5.16	1.37	3.42	0.37	191
4.68	1.4	2.85	0.43	264
4.36	1.54	2.32	0.5	58
4.23	1.98	1.84	0.41	70
3.89	1.49	2.18	0.22	167
5.52	1.36	3.77	0.39	181
4.52	1.69	2.59	0.24	174
5.22	1.24	3.39	0.59	443
5.54	1.43	3.28	0.83	78
4.73	1.17	3.14	0.42	72
4.24	1.37	2.75	0.12	129
5.01	1.81	2.85	0.35	25
5.09	1.95	3.09	0.05	95
3.56	1.19	1.98	0.39	208
3.36	1.22	1.89	0.25	284
5.04	1.62	3.08	0.34	83
3.21	1.17	1.72	0.32	176
4.2	1.24	2.67	0.29	132
3.96	1.09	2.58	0.29	59
5	1.26	3.16	0.58	222
4.98	1.44	3.09	0.45	116
3.62	1.44	1.26	0.92	3
3.48	1.03	1.86	0.59	114
5.65	1.88	3.23	0.54	90
4.81	1.28	2.47	1.06	177
4.34	1.81	2.22	0.31	71
4.72	1.41	2.6	0.71	191
3.26	0.89	1.87	0.5	
4.44	1.13	2.68	0.63	106
5.18	1.09	3.65	0.44	230
5.35	1.23	3.35	0.77	91
4.68	1.02	2.29	1.37	20
5.87	1.27	3.08	1.52	142
5.07	1.52	2.82	0.73	3
6.01	1.74	3.27	1	65
7.69	1.46	4.81	1.42	73
3.29	1.19	1.79	0.31	244
3.43	1.35	1.83	0.25	248
4.55	1.25	2.89	0.41	100
5.21	1.73	3.04	0.44	253
3.44	1.04	1.92	0.48	31
4.24	1.22	2.71	0.31	239
5.93	1.21	3.98	0.74	297

3.7	0.92	2.49	0.29	135
4.91	1.23	3.48	0.2	109
4.73	1.39	3.13	0.21	127
5.63	1.86	3.64	0.13	85
4.24	1.71	2.51	0.02	308
2.72	1.13	1.55	0.04	174
3.81	1.75	1.77	0.29	116
3.77	1.22	2.14	0.41	89
4.39	1.62	2.63	0.14	345
4.49	1.49	2.82	0.18	217
4.14	1.55	2.39	0.2	156
3.37	1	1.84	0.53	151
3.01	1.07	1.79	0.15	96
5.54	1.89	3.29	0.36	51
4.74	1.92	2.42	0.4	154
4.31	1.54	2.45	0.32	3
4.05	1.14	2.27	0.64	358
4.46	1.8	2.33	0.33	99
4.06	1.39	2.34	0.33	239
3.46	1.23	1.82	0.41	71
4.91	1.46	3	0.45	347
5.81	1.65	3.67	0.49	526
3.36	1.29	1.77	0.3	49
4.83	1.35	2.86	0.62	641
3.65	1.1	2.38	0.17	102
4.02	1.45	2.23	0.34	589
5.37	1.88	2.77	0.72	235
3.56	1.14	2.37	0.05	19
4.16	1.17	2.68	0.31	335
2.98	1.18	1.52	0.28	63
3.82	1	2.45	0.37	63
5.14	1.24	3.46	0.44	201
4.08	1.3	2.54	0.24	143
5.53	1.21	3.81	0.51	111
4.57	1.42	2.33	0.82	517
5.07	1.25	3.15	0.67	135
2.42	1.23	0.68	0.51	144
4.42	1.45	2.56	0.41	26
4.95	1.67	2.79	0.49	3
4.23	0.93	2.75	0.55	81
3.48	1	2.05	0.43	67
3.56	1.22	2.04	0.3	149
4.81	1.02	3.4	0.39	166
4.56	1.05	2.96	0.55	70
4.83	1.09	2.72	1.02	380
5.52	1.18	3.79	0.55	192
4.98	1.37	3.01	0.6	133
6.71	1.25	4.64	0.82	52

4.54	1.2	2.23	1.11	59
6.54	1.28	4.07	1.19	410
3.76	1.58	1.92	0.26	53
4.17	1.67	2.37	0.13	76
4.52	1.3	2.56	0.66	76
4.57	1.86	2.49	0.22	915
3.09	1.08	1.48	0.53	224
3.61	1.19	2.07	0.35	381
4.49	1.27	2.96	0.26	124
3.74	1.39	2.03	0.32	357
4.07	1.46	2.4	0.21	885
3.78	1.02	2.14	0.62	257
4.86	2.26	2.25	0.35	198
4.06	1.42	2.4	0.24	305
3.8	0.86	2.22	0.72	313
4.61	1.54	2.64	0.43	212
4.23	1.49	2.4	0.34	147
4.81	1.56	2.94	0.31	67
3.31	1.23	1.89	0.19	100
4.12	1.14	2.52	0.46	108
4.17	1.35	2.69	0.13	394
3.62	1.26	2.11	0.25	313
4.3	1.57	2.39	0.34	76
4.84	1.7	2.66	0.48	324
4.79	1.55	2.95	0.29	532
4.88	1.48	2.84	0.56	126
3.83	1.22	2.32	0.29	47
3.27	1.05	1.97	0.25	238
4.12	1.25	2.59	0.28	89
3.55	0.97	2.22	0.36	33
4.24	1.03	2.9	0.31	135
4.76	1.12	2.91	0.73	178
4.15	1.16	2.61	0.38	57
5.97	1.41	4.41	0.15	98
5.09	1.67	2.92	0.5	171
5.07	1.57	3.24	0.26	52
5.26	1.42	3.14	0.7	283
3.92	1.22	2.24	0.46	185
4.88	1.01	3.3	0.57	110
6.17	1.56	3.84	0.77	3
3.85	0.72	1.96	1.17	41
4.98	1.86	2.82	0.3	60
3.07	1.52	1.42	0.13	164
4.27	1.41	2.5	0.36	111
4.51	1.43	2.78	0.30	3
3.51	1.06	2.15	0.3	188
3.45	1.46	1.62	0.37	26
4.33	1.4	2.59	0.34	119

4.62	1.35	2.88	0.39	106
5.24	1.4	2.86	0.98	236
4.04	1.53	2.5	0.01	125
3.67	1.92	1.69	0.06	102
4.75	1.99	2.56	0.2	122
4.93	1.68	2.68	0.57	101
3.48	1.59	1.8	0.09	122
3.56	1.37	1.91	0.28	145
4.38	2.01	2.09	0.28	57
5.04	1.57	3.13	0.34	182
5.22	2.28	2.32	0.62	226
5.02	1.55	2.96	0.51	124
6.1	1.48	4.35	0.27	1159
4.92	1.93	2.72	0.27	276
3.86	1.42	2.13	0.31	33
5.48	1.6	3.53	0.35	1063
5.76	2.11	2.88	0.77	3
4.18	1.32	2.23	0.63	365
5.82	1.6	3.72	0.5	247
4.45	1.42	2.29	0.74	128
4.02	1.27	2.42	0.33	31
4.2	1.18	2.8	0.22	52
3.76	1.22	2.21	0.33	27
3.91	1.31	2.23	0.37	110
3.53	0.97	2.23	0.33	223
3.96	1.37	2.32	0.27	271
4.24	1.01	2.72	0.51	284
4.5	1.48	2.37	0.65	187
3.08	0.87	1.99	0.22	80
4.72	1.52	2.87	0.33	159
4.38	1.31	2.61	0.46	257
4.79	1.11	3.2	0.48	66
6.22	1.79	3.8	0.63	181
4.64	1.48	2.87	0.29	76
4.51	0.99	3.11	0.41	366
5.84	1.46	3.33	1.05	462
5.1	1.78	2.87	0.45	170
4.15	1.58	2.26	0.31	71
4.93	1.15	3.04	0.74	43
4.2	0.74	3.03	0.43	166
5.35	1.63	3.11	0.61	88
5.1	0.99	3.34	0.77	34
5.51	1.29	3.16	1.06	247
5.05	1.38	3.07	0.6	18
5.87	1.35	3.37	1.15	1121
3.68	0.83	2.16	0.69	32
4.29	0.83	2.19	1.27	841
3.58	0.99	1.75	0.84	434

4.84	0.98	2.92	0.94	68
3.31	0.97	1.38	0.96	3
4.28	1.41	2.24	0.63	11
5.97	2.13	3.45	0.39	80
4.03	1.26	2.41	0.36	64
5.68	1.75	3.16	0.77	501
2.71	0.82	1.6	0.29	185
4.25	1.42	2.44	0.39	439
5.4	1.81	3.02	0.57	188
5.13	1.43	3.1	0.6	13
4.31	1.66	2.24	0.41	541
3.79	1.2	2.11	0.48	561
4.82	1.49	3.11	0.22	118
4.74	1.44	3.05	0.25	63
5.9	1.42	3.93	0.55	820
3.16	0.78	2.05	0.33	1131
5.51	2	3	0.51	193
4.78	1.2	3.02	0.26	141
4.09	0.81	2.46	0.82	322
4.77	1.36	3.11	0.3	358
6.56	1.37	4.32	0.87	938
4.97	1.67	2.95	0.35	326
6.15	1.31	4.33	0.51	776
3.15	1.08	1.62	0.45	263
4.11	0.97	2.27	0.87	107
4.53	1.12	2.84	0.57	182
3.44	1.03	1.97	0.44	109
3.03	1.03	1.6	0.4	88
6.3	1.69	4	0.61	531
2.8	1.7	0.91	0.19	157
4.4	1.16	3.2	0.04	107
5.23	1.81	3.35	0.07	292
4.69	1.32	2.97	0.4	44
4.07	1.2	2.67	0.2	331
4.52	1.56	2.71	0.25	317
4.7	1.51	2.44	0.75	575
3.91	1.45	2.1	0.36	138
5.56	1.28	3.67	0.61	255
4.13	1.51	1.9	0.72	887
4.78	1.37	2.86	0.55	219
3.75	1.09	2.05	0.61	55
4.96	1.18	3.32	0.46	217
4.04	0.91	2.85	0.28	133
3.12	1.08	1.36	0.68	442
4.11	1.32	2.53	0.26	77
2.73	1.2	1.34	0.19	247
6.19	2.26	3.51	0.42	533
3.15	1.18	1.8	0.17	237

2.96	0.69	1.71	0.56	96
3.94	1.09	2.37	0.48	154
4.77	1.28	2.41	1.08	376
4.53	2.19	2.19	0.15	89
3.41	1.1	2.2	0.11	58
3.38	0.94	2.29	0.15	257
4.01	1.7	2.11	0.2	80
3.99	1.49	2.35	0.15	227
3.03	1.14	1.81	0.08	312
3.88	1.57	2.06	0.25	142
3.54	1.46	2	0.08	170
3.77	1.44	2.08	0.25	20
4.03	1.29	2.41	0.33	61
4.33	1.78	2.51	0.04	133
3.84	1.84	1.87	0.13	159
5.91	1.8	3.8	0.31	97
4.85	1.54	3.05	0.26	144
4.72	1.8	2.78	0.14	116
5.57	2.03	3.19	0.35	151
3.89	1.17	2.64	0.08	207
4.61	1.47	2.74	0.40	341
4.67	1.85	2.52	0.3	97
4.26	1.32	2.8	0.14	81
4.43	1.49	2.8	0.14	600
5.62	2.11	3.36	0.15	86
3.65	1.49	1.95	0.21	59
5.12	1.65	2.99	0.48	35
4.29	1.39	2.53	0.37	122
2.95	1.04	1.75	0.16	55
5.05	1.67	3.2	0.18	840
3.25	1.43	1.59	0.23	126
5.43	1.59	3.5	0.34	158
4.42	0.96	2.78	0.68	449
4.55	1.68	2.58	0.29	196
5.01	2.05	2.49	0.47	59
4.25	1.33	2.5	0.42	82
4.82	1.13	3.27	0.42	161
3.52	1.35	1.84	0.33	167
3.51	1.34	1.74	0.43	41
6.39	1.71	4.51	0.17	787
5.72	2.04	3.35	0.33	264
3.05	0.87	2.03	0.15	73
3.58	1.26	2.1	0.22	145
4.07	0.98	2.63	0.46	611
5.02	1.18	3.4	0.44	211
3.05	1.03	1.66	0.36	31
4.02	1.21	2.36	0.45	367
5.45	1.39	3.84	0.22	616

4.27	1.37	2.47	0.43	773
4.87	1.48	3.15	0.24	236
3.67	1.31	2.1	0.26	833
4.1	0.98	2.60	0.52	75
5.71	0.98	3.97	0.76	188
4	0.82	2.81	0.37	98
5.56	1.63	3.35	0.58	69
3.9	1.03	2.3	0.57	65
5.28	1.25	3.55	0.48	144
4.62	0.83	2.28	1.51	78
5.92	1.42	2.85	1.65	74
6.2	2.09	3.52	0.59	218
1.76	0.74	0.9	0.12	481
5.82	2.15	3.26	0.41	412
3.5	1.1	2.35	0.05	247
4.9	2.01	2.46	0.43	53
4.36	1.78	2.24	0.34	3
4.15	1.76	2.15	0.24	211
3.07	0.94	1.78	0.35	197
3.42	1.14	2.1	0.18	152
3.98	1.7	2.04	0.24	150
5.17	1.86	2.76	0.55	614
3.3	1.54	1.48	0.28	45
4.55	1.36	2.81	0.38	241
4.14	1.97	2.08	0.09	195
4.5	1.66	2.52	0.32	212
3.95	1.35	2.29	0.31	109
4.66	1.25	3.35	0.06	459
4.6	1.23	2.94	0.43	102
5.48	1.4	3.39	0.69	136
5.18	1.88	3.04	0.26	889
4.08	1.85	1.92	0.31	247
3.51	1.49	1.68	0.34	3
4.29	1.24	2.64	0.41	568
5.44	1.28	3.97	0.19	263
6.06	1.15	4.11	0.8	68
5.85	1.36	4.28	0.21	22
4.66	1.49	2.58	0.59	26
4.8	1.11	3.06	0.63	429
4.86	1.34	3.11	0.41	233
4.64	1.24	2.71	0.69	55
5.26	1.35	3.49	0.42	44
5.99	1.49	4.01	0.49	115
5.05	1.4	3.1	0.55	326
3.67	1.16	1.86	0.65	197
3.93	1.05	2.2	0.68	52
4.11	1.3	1.89	0.92	64
8.39	1.04	6.35	1	969

5.98	1.03	3.65	1.3	380
5.07	1.29	2.97	0.81	139
4.24	1.44	2.6	0.2	115
4.27	1.52	2.67	0.08	162
3.65	1.34	2.13	0.18	143
3.99	1.37	2.44	0.18	73
3.56	1.24	2.02	0.3	310
5.93	1.41	4.49	0.03	127
5.1	1.19	3.45	0.46	912
3.13	1.22	1.82	0.09	226
4.17	1.31	2.64	0.22	551
4.5	1.67	2.2	0.63	94
6.5	1.72	4.69	0.09	253
3.32	1.55	1.50	0.27	40
3.78	1.12	2.41	0.25	218
3.89	1.35	2.09	0.45	200
4.92	1.38	3.31	0.23	72
4.05	1.53	1.84	0.68	169
5.65	0.84	3.81	1	193
4.36	1.16	2.75	0.45	36
3.37	0.9	1.22	1.25	61
4.24	1.05	3	0.19	111
4.69	2.2	2.31	0.18	102
3.88	1.62	2.15	0.11	298
3.59	0.91	2.64	0.04	196
3.44	1.11	2.15	0.18	56
3.66	1.18	2.37	0.11	243
3.78	0.84	2.87	0.07	223
4.83	1.28	3.44	0.11	112
4.15	1.72	2.3	0.13	35
1.68	0.49	1.06	0.13	98
3.1	1.25	1.64	0.21	148
5.35	1.39	3.8	0.16	813
3.21	1.25	1.88	0.08	35
3.23	1.14	2.03	0.06	3
4.35	1.82	2.41	0.12	427
4.12	1.4	2.57	0.15	184
3.51	1.16	2.14	0.21	334
4.32	1.37	2.8	0.15	45
3.95	1.28	2.57	0.1	171
3.53	1.68	1.69	0.16	251
2.77	1.48	1.1	0.19	121
2.92	1.13	1.72	0.07	76
3.82	1.11	2.64	0.07	103
4.12	1.71	2.3	0.11	189
3.69	1.49	1.94	0.26	78
3.17	1.31	1.71	0.15	7
4.07	1.47	2.37	0.23	113

3.84	1.42	1.86	0.56	174
3.49	1.09	2.37	0.03	108
3.45	1.33	2.09	0.03	40
3.39	0.98	2.35	0.06	537
5.2	1.96	3.01	0.23	917
3.34	0.99	1.89	0.46	97
3.75	1.66	1.91	0.18	73
4.02	1.37	2.08	0.57	17
4.07	1.3	2.5	0.27	97
4.16	1.31	2.5	0.35	360
4.76	1.4	2.96	0.4	101
3.99	1.46	2.05	0.48	164
4.09	2.19	1.3	0.6	203
3.32	1.15	1.92	0.25	115
4.21	1.6	2.39	0.22	187
4.38	1.69	2.06	0.63	191
4.76	1.07	3.44	0.25	168
4.33	1.64	1.89	0.8	42
5.63	1.57	3.53	0.53	227
3.12	0.99	2.02	0.11	196
4.37	1	3.24	0.13	345
4.11	1.16	2.93	0.02	194
4.05	1.53	2.26	0.26	109
4.29	1.27	2.62	0.4	332
4.19	1.77	2.25	0.17	509
3.6	1.23	1.65	0.72	177
3.81	1.76	2	0.05	16
3.84	1.58	2.13	0.13	169
4.65	2.35	2.05	0.25	488
4.38	1.39	2.23	0.76	29
4.22	1.1	2.39	0.73	572
4.94	1.77	2.89	0.28	50
4.02	1.22	2.58	0.22	831
4.77	1.79	2.46	0.52	103
4.78	1.68	2.55	0.55	40
3.12	1	2.02	0.1	173
3.73	1.09	2.4	0.24	313
4.98	1.32	3.26	0.4	128
4.66	2.55	1.85	0.26	29
3.77	1.12	2.55	0.1	22
3.48	1.39	1.89	0.2	180
3.24	0.97	1.93	0.34	50
3.01	0.97	1.92	0.12	317
3.52	1.49	1.71	0.32	437
4.44	1.51	2.38	0.55	45
5.3	1.6	3.38	0.32	141
4.5	1.62	2.68	0.2	152
3.39	1.37	1.69	0.33	368

4.54	1.46	2.97	0.11	274
4.13	1.74	2.21	0.18	252
4.48	1.31	2.73	0.44	133
4.77	1.77	2.2	0.8	85
3.46	1.34	1.96	0.16	42
5.41	1.74	3.39	0.28	290
5.44	1.79	3.36	0.29	157
3.4	1.23	1.88	0.29	17
4.71	1.74	2.67	0.3	405
3.37	1.27	1.56	0.54	61
4.16	1.09	2.45	0.62	87
3.89	1.57	2.08	0.24	193
4.52	1.31	2.37	0.84	58
3.89	1.67	1.97	0.25	873
2.9	1.18	1.44	0.28	74
3.63	1.47	1.76	0.4	173
5.68	1.55	3.73	0.4	76
4.81	1.65	2.86	0.3	100
3.15	1.05	1.92	0.18	177
5.98	1.41	4.09	0.48	57
4.04	1.01	2.63	0.4	3
3.84	1.09	2	0.75	173
3.64	1.3	2.03	0.31	44
4.87	1.56	2.28	1.03	148
4.46	1.4	2.65	0.41	175
3.57	1.28	1.9	0.39	19
4.2	0.98	2.89	0.33	508
3.99	1.16	2.73	0.1	145
3.46	1.48	1.77	0.21	234
5.17	1.28	3.54	0.35	184
3.66	1.3	1.72	0.64	17
2.95	0.97	1.93	0.05	12
3.72	0.86	2.25	0.61	119
3.57	0.97	2.17	0.43	374
5.12	1.31	3.41	0.4	78
4.07	1.08	2.2	0.79	229
5.13	1.54	2.8	0.79	162
5.2	1.21	3.56	0.43	605
3.85	1.27	2.31	0.27	436
4.1	1.18	2.62	0.3	3
4.43	1.51	2.65	0.27	57
4.37	1.09	2.8	0.48	72
4.07	1.14	2.43	0.5	95
3.86	1.05	2.12	0.69	98
4.73	1.72	2.47	0.54	230
4.72	1.13	2.66	0.93	15
4.46	1.08	2.46	0.92	185
5.68	1.35	3.67	0.66	168

4.33	1.03	2.9	0.4	183
5.36	1.08	3.28	1	210
4.06	0.98	2.6	0.48	115
5.01	1.02	3.04	0.95	154
4.45	1.17	2.12	1.16	71
3.8	1.16	2.2	0.44	74
5.52	1.65	3.06	0.81	229
4.68	1.26	2.77	0.65	62
4.27	0.9	2.67	0.7	181
4.87	1.24	3.38	0.25	240
4.28	0.8	2.87	0.61	165
4.48	1.08	2.29	1.11	467
6.26	1.37	4.14	0.75	61
4.78	1.68	2.75	0.35	234
4.69	0.81	2.77	1.11	96
3.56	0.7	1.46	1.4	59
3.86	1.31	2.54	0.01	100
3.92	1.62	2.22	0.08	158
3.94	1.62	2.14	0.18	124
3.1	1.39	1.69	0.02	181
3.72	1.16	2.22	0.34	40
3.88	1.66	2.08	0.14	80
3.73	1.49	2.18	0.06	254
3.67	1.87	1.63	0.17	3
5.88	1.92	3.66	0.3	121
6.52	1.97	3.97	0.58	314
3.48	1.24	2.02	0.22	0
3.33	1.18	2	0.15	3
3.53	1.5	2.02	0.01	50
5.68	2.08	3.29	0.31	213
3.56	1.31	2.03	0.22	98
3.46	1.15	2.02	0.29	9
4.08	1.56	2.04	0.48	133
3.3	1.16	1.88	0.26	198
4.84	2.02	2.58	0.24	98
5.07	2.07	2.1	0.9	88
3.49	1.13	2.26	0.1	94
3.75	1.31	2.26	0.18	94
3.5	1.34	2.03	0.13	134
4.52	1.47	2.35	0.7	899
4.58	1.41	3.12	0.05	131
3.92	1.25	2.6	0.07	693
3.52	1.38	1.95	0.19	156
4.14	1.56	2.09	0.49	75
5.68	2.3	3.07	0.31	327
4.6	1.38	2.85	0.37	984
4.83	1.86	2.37	0.6	822
4.37	1.53	2.8	0.04	147

4.3	1.19	2.93	0.18	322
4.72	2.48	2.04	0.2	37
5.12	1.63	2.79	0.7	85
4.19	1.54	2.36	0.29	66
4.2	1.5	2.38	0.32	33
3.34	1.55	1.54	0.25	53
4.47	1.32	2.6	0.55	65
3.78	1.27	2.26	0.25	145
3.62	1.24	2.04	0.34	217
4.95	1.52	3.23	0.2	132
5.48	1.62	2.82	1.04	584
4.07	1.49	2.49	0.09	100
4.31	0.96	3.32	0.03	59
3.38	1.06	1.96	0.36	123
4.12	1.89	2.06	0.17	145
4.18	1.43	2.43	0.32	3
4.23	1.64	2.35	0.24	245
3.87	1	2.62	0.25	335
3.19	0.98	1.92	0.29	91
3.99	1.23	2.44	0.32	83
3.3	0.99	2.07	0.24	209
4.42	1.14	2.44	0.84	141
4.61	1.58	2.66	0.37	46
3.35	1.04	2.15	0.16	106
4.73	1.17	3.28	0.28	63
5.14	1.12	3.33	0.69	229
5.3	1.64	3.12	0.54	133
4.37	1.41	2.54	0.42	68
3.56	1.06	2.1	0.4	55
5.06	1.67	3.09	0.3	241
4	1.46	2.11	0.43	140
4.02	1.33	2.52	0.17	194
4.07	1.07	2.85	0.15	333
2.97	0.89	1.89	0.19	234
4.11	1.22	2.54	0.35	67
3.3	1.33	1.75	0.22	85
3.11	1.1	1.95	0.06	29
4.92	1.23	2.96	0.73	209
4.62	1.54	2.84	0.24	86
3.19	1.02	1.94	0.23	28
4.34	1.32	2.69	0.33	118
3.97	1.56	2.15	0.26	84
4.69	1.34	2.6	0.75	334
4.23	1.5	2.7	0.03	144
4.57	1.28	2.88	0.41	129
5.65	1.82	3.04	0.79	121
4.26	1.21	2.54	0.51	106
5.43	1.69	3.37	0.37	96

3.26	0.94	1.93	0.39	428
4.2	1.08	2.69	0.43	43
5.09	1.39	2.65	1.05	440
4.76	1.37	2.93	0.46	791
5.47	1.41	3.61	0.45	479
5.19	1.3	3.39	0.5	69
4	1.06	2.62	0.32	140
3.78	1.29	2.16	0.33	135
4.73	1.25	2.556	0.92	159
4.38	0.98	2.61	0.79	94
6.12	1.29	4.2	0.63	292
5.72	1.54	3.49	0.69	83
4.4	1.46	2.39	0.55	178
4.48	1.09	2.98	0.41	75
4.32	1.38	2.63	0.31	134
3.34	1.14	1.91	0.29	119
4.34	1.01	2.57	0.76	39
5.52	1.48	3.68	0.36	36
4.21	1.16	2.47	0.58	181
3.59	0.94	2.26	0.39	5
6.96	1.79	4.14	1.03	73
3.74	1.13	2.44	0.17	82
4.7	1.13	2.81	0.76	760
3.57	0.96	2.1	0.51	242
5.34	1.24	3.69	0.41	298
3.84	1.14	2.26	0.44	38
3.2	1.07	1.87	0.26	1128
5.55	1.49	3.81	0.25	37
4.88	1.42	2.88	0.58	873
4.34	1.2	2.48	0.66	363
4.91	1.37	3.19	0.35	35
5.82	1.27	3.89	0.66	272
5.46	1.39	3.18	0.89	69
3.92	1.15	1.28	1.49	33
6.12	1.5	4.28	0.34	703
4.74	1.43	2.41	0.9	68
4.09	1.43	1.92	0.74	215
3.76	1	2.23	0.53	30
5.01	1.21	2.86	0.94	105
4.37	1.07	2.64	0.66	428
4.85	1.06	3.03	0.76	90
1.32	1.07	2.53	0.49	92
4.61	0.91	1.8	1.9	28
4.59	1.37	2.5	0.72	107
3.05	1.63	1.27	0.15	72
4.42	1.67	2.57	0.18	85
3.85	1.55	2.13	0.17	438
5.1	1.63	3.26	0.21	45

3.83	1.19	2.56	0.08	22
3.24	1.41	1.67	0.16	179
4.24	1.67	2.37	0.2	356
5.48	1.36	4.08	0.04	94
3.73	1.64	1.91	0.18	327
4.21	1.5	2.54	0.17	45
4.49	1.82	2.47	0.2	184
3.89	1.31	2.45	0.13	98
3.13	1.18	1.8	0.15	94
3.51	1.31	2.18	0.02	762
4.68	1.31	3.29	0.08	168
2.28	0.85	1.3	0.13	76
4.78	1.52	3.07	0.19	312
4.61	1.4	3.03	0.18	251
3.98	1.32	2.53	0.13	277
2.9	1.2	1.65	0.05	85
3.18	1.11	1.92	0.15	48
4.05	1.37	2.6	0.08	37
3.33	1.04	2.18	0.11	55
3.49	1.23	2.11	0.15	136
3.4	1.05	2.19	0.16	204
3.39	1.18	2.02	0.19	4
4.19	1.6	2.52	0.07	70
3.71	1.6	1.94	0.17	124
3.55	1.48	1.91	0.16	152
3.85	1.3	2.16	0.39	107
5.18	0.98	3.34	0.86	770
3.26	1.29	1.82	0.15	3
3.57	1.2	2.08	0.29	53
4.24	1.07	2.33	0.84	339
3.48	0.86	2.12	0.5	272
5.08	1.32	3.18	0.58	228
3.99	1.38	2.58	0.03	1114
4.7	1.4	3.09	0.21	65
3.45	1.07	2.2	0.18	123
4.03	0.92	2.35	0.76	53
3.29	1.26	1.86	0.17	1028
4.23	1.63	2.46	0.14	65
4.49	1.37	3.03	0.09	296
3.52	1.57	1.79	0.16	31
3.75	1.72	1.82	0.21	133
4.97	1.68	3.21	0.08	73
4.05	1.52	2.38	0.15	138
4.59	1.52	2.98	0.09	1690
3.44	1.48	1.78	0.18	132
4.48	1.67	2.71	0.1	294
4.32	1.52	2.62	0.18	692
4.4	1.49	2.77	0.14	125

4.21	1.59	2.43	0.19	59
4.12	1.46	2.47	0.19	216
4.1	1.76	2.16	0.18	307
4.44	1.76	2.5	0.18	78
4.73	1.33	3.27	0.13	671
5.06	1.43	3.57	0.06	129
5.58	1.68	3.75	0.15	44
3.77	1.45	2.26	0.06	177
4.46	1.42	3	0.04	624
4.12	1.16	2.82	0.14	626
4.38	2.07	2.18	0.13	464
3.79	1.42	2.35	0.02	211
3.94	1.85	1.84	0.25	255
3.99	0.99	2.9	0.1	190
5.48	1.8	3.01	0.67	948
3.79	1.76	1.8	0.23	26
4.48	1.49	2.67	0.32	156
3.58	1.23	2.26	0.09	74
3.48	1.09	2.24	0.15	630
5.6	1.92	3.44	0.24	246
3.84	1.29	2.36	0.19	109
3.4	1.39	1.95	0.06	37
3.63	1.4	2.05	0.18	198
4.21	1.8	2.38	0.03	201
6.4	2	3.8	0.6	280
3.73	1.34	2.22	0.17	108
3.93	1.33	2.31	0.29	146
4.12	1.38	2.16	0.58	217
4.56	1.59	2.74	0.23	102
3.04	0.91	1.6	0.53	136
4.45	1.54	2.17	0.74	59
4.3	1.71	2.1	0.49	78
4.19	1.55	2.06	0.58	382
4.55	1.29	2.85	0.41	475
3.57	1.02	2.47	0.08	399
4.02	1.38	2.44	0.2	126
4.06	1.26	2.65	0.15	101
3.87	1.36	2.23	0.28	82
3.81	1.08	2.55	0.18	152
4.8	1.63	3.1	0.07	276
4.32	1.53	2.67	0.12	435
4.66	1.73	2.61	0.32	334
4.77	1.99	2.29	0.49	89
4.77	1.8	2.68	0.29	286
3.73	1.15	2.02	0.56	238
4.97	1.63	2.81	0.53	502
3.5	1.62	1.79	0.09	72
3.64	1.4	2.22	0.02	139

6.1	1.61	4.01	0.48	689
3	0.92	2.02	0.06	19
4.45	1.47	2.94	0.04	236
3.75	1.31	1.91	0.53	30
5.78	2.04	3.29	0.45	151
5.33	1.52	3.3	0.51	263
3.81	1.4	1.99	0.42	197
4.39	1.61	2.35	0.43	103
3.53	1.71	1.65	0.17	101
3.67	1.05	2.56	0.06	202
3.34	1.28	1.83	0.23	57
3.38	1.19	1.88	0.31	194
4.34	1.42	2.47	0.45	30
4.34	1.41	2.66	0.27	251
3.2	0.96	2.05	0.19	90
4.28	1.23	2.67	0.38	75
5.4	1.67	3.51	0.22	187
4.57	1.45	2.81	0.31	54
3.12	0.92	1.7	0.5	120
3.89	1.48	2.11	0.3	334
3.66	1.28	2.12	0.26	19
4.46	1.22	3.02	0.22	34
4.25	1.37	2.54	0.34	204
3.8	1.63	1.99	0.18	3
5.79	2.09	3.13	0.57	1022
3.24	1.06	1.85	0.33	84
5.57	1.49	3.33	0.75	278
3.57	1.04	2.28	0.25	200
4.3	1.23	2.69	0.38	955
3.71	1.51	2.12	0.08	61
5.08	1.85	2.72	0.51	126
3.31	0.98	2.02	0.31	44
4.68	1.29	2.9	0.49	192
5.35	0.95	3.97	0.43	408
4.29	0.96	2.79	0.54	498
5.2	1.59	3.1	0.51	87
4.77	1.41	2.68	0.68	316
3.3	0.9	2.15	0.25	38
4.55	1.1	2.78	0.67	176
4.44	1.31	3.07	0.06	97
5.1	1.28	3.48	0.34	94
4.6	1.34	2.48	0.78	130
4.7	2.13	2.09	0.48	112
4.74	1.63	2.69	0.42	299
4.69	1.27	3.24	0.18	124
5.25	1.47	3.7	0.08	107
5.31	1.3	3.39	0.62	143
3.76	1.17	2.39	0.2	204

4.57	1.17	2.71	0.69	304
4.12	1.37	2.4	0.35	129
3.66	0.85	2.33	0.48	43
5.55	1.77	3.26	0.52	442
4.72	1.35	2.58	0.79	108
4.39	1.33	2.51	0.55	150
3.95	1.22	2.34	0.39	223
6.08	1.37	3.65	1.06	581
4.64	1.26	2.94	0.44	36
3.63	0.86	2.38	0.39	47
9.37	2.09	6.69	0.59	983
5.42	1.8	2.66	0.96	50
6.48	1.5	4.65	0.33	159
3.44	1.16	1.98	0.3	72
3.76	0.91	2.32	0.53	277
6.55	2.06	3.9	0.59	144
5.8	1.99	3.22	0.59	103
4.77	1.07	2.6	1.1	197
4.64	0.94	3.52	0.18	512
4.98	1.11	3.3	0.57	60
4.74	1.15	3.15	0.44	204
4.98	1.22	2.78	0.98	73
6.07	1.32	3.65	1.1	281
3.66	1.09	1.82	0.75	73
3.91	1.02	2.6	0.29	252
5.78	1.59	3.29	0.9	207
5.19	1.23	3.49	0.47	155
5.32	1.1	3.66	0.56	442
4.92	1.27	3.24	0.41	107
4.58	0.81	2.83	0.94	301
3.52	0.96	2.16	0.4	935
3.96	1.23	1.92	0.81	132
3.45	1.07	1.6	0.78	51
4.15	0.96	1.73	1.46	281
4.31	1.53	2.57	0.21	138
3.69	1.74	1.72	0.23	18
4.06	1.41	2.62	0.03	177
3.6	1.89	1.61	0.1	370
3.67	1.7	1.72	0.25	13
3.34	1.33	1.72	0.29	54
3.48	1.52	1.56	0.4	59
6.01	1.56	4.02	0.43	240
4.17	1.34	2.26	0.57	27
4.11	1.27	2.51	0.33	136
3.43	1.62	1.55	0.26	76
3.61	1.46	1.9	0.25	53
4.08	1.55	2.51	0.02	194
3.49	1.53	1.89	0.07	252

5.22	1.77	2.98	0.47	184
4.84	1.16	2.88	0.8	119
3.38	1.34	1.83	0.21	329
5.46	1.94	3.33	0.19	126
4.65	1.41	2.84	0.4	652
3.54	1.61	1.37	0.56	163
4.17	1.61	2.24	0.32	46
3.85	1.37	2.2	0.28	108
3.67	1.53	2.07	0.07	212
3.98	1.61	2.11	0.26	32
4.47	1.91	2.12	0.44	606
4.78	1.23	2.58	0.97	152
3.46	1.16	2.06	0.24	93
4.07	1.39	2.24	0.44	34
5.39	1.76	2.96	0.67	704
3.91	0.91	2.8	0.2	144
4.48	1.22	2.87	0.39	422
4.58	1.5	2.46	0.62	247
3.91	1.25	2.29	0.37	130
3.42	1.38	1.85	0.19	95
3.7	1.16	2.31	0.23	269
4.13	1.63	2.24	0.26	162
3.8	1.12	2.16	0.52	306
3.99	1.06	2.53	0.4	267
4.53	1.34	2.93	0.26	176
4.36	1.19	2.75	0.42	89
4.76	1.71	2.6	0.45	130
3.09	0.97	1.98	0.14	173
4.45	1.35	2.94	0.16	71
5.51	1.63	3.15	0.73	365
4.09	1.43	2.21	0.45	64
4.31	0.93	3.07	0.31	186
4.42	1.4	2.74	0.28	95
4.77	1.83	2.7	0.24	130
3.75	1.08	2.29	0.38	264
4.84	1.63	2.76	0.45	269
5.3	1.49	3.4	0.41	690
4.09	1.94	2.08	0.07	27
3.97	1.38	2.11	0.48	22
4.72	1.88	2.71	0.13	215
4.22	1.2	2.46	0.56	125
4.66	1.75	2.66	0.25	108
3.63	1.08	2.35	0.2	333
5.54	1.5	3.54	0.5	552
4.77	1.27	2.87	0.63	107
4.94	1.59	2.88	0.47	238
4.07	1.46	2.45	0.16	370
5.8	1.6	3.74	0.46	98

4.56	1.34	2.73	0.49	108
3.49	0.85	2.23	0.41	91
4.44	1.46	2.6	0.38	159
4.29	1.23	2.54	0.52	63
4.29	1.53	2.41	0.35	135
4.53	1.28	2.86	0.39	75
4.19	1.28	2.56	0.35	73
4.07	1.38	2.43	0.26	145
4.08	1.02	2.61	0.45	95
4.91	1.06	3.26	0.59	160
4.64	1.14	3.02	0.48	847
4.03	1.2	2.41	0.42	59
4.18	1.46	2.41	0.31	163
5.04	1.11	3.52	0.41	623
4.43	1.03	2.84	0.56	40
4.13	1.53	2.39	0.21	767
3.98	1.19	2.14	0.65	247
3.36	0.78	2	0.58	62
5.44	1.46	3.47	0.51	3
4.67	1.03	2.95	0.69	141
6.65	1.83	4.23	0.59	539
5.22	1.2	3.47	0.55	188
4.99	1.22	3.21	0.56	3
4.3	1.32	2.42	0.56	55
3.6	1.22	1.88	0.5	117
3.95	1.22	2.26	0.47	76
3.9	0.88	2.4	0.62	75
3.93	1.04	2.43	0.46	23
4.1	0.97	2.61	0.52	627
5.21	1.41	3.09	0.71	237
5.64	1.11	3.86	0.67	84
5.74	1.18	3.62	0.94	3
3.75	1	2.33	0.42	206
3.89	0.89	2.65	0.35	117
4.52	1.58	2.21	0.73	110
4.29	0.79	3.01	0.49	230
6.24	1.29	4.3	0.65	527
4.54	1.19	2.96	0.39	641
4.24	0.91	2.64	0.69	813
5.37	1.21	3.63	0.53	88
4.87	0.91	3.56	0.4	251
3.6	0.9	2.03	0.67	81
4.3	1.08	1.73	1.49	30
4.36	1.25	2.37	0.74	55
4.33	1.26	2.01	1.06	212
4.04	0.83	2.38	0.83	224
4.78	1.02	2.97	0.79	404
4.55	0.98	2.09	1.48	3

4.7	0.94	1.59	2.17	38
4.44	1.12	1.96	1.36	204
3.43	1.46	1.96	0.01	100
3.22	1.19	1.93	0.1	49
3.53	1.36	2.00	0.17	73
4.13	1.36	2.71	0.06	243
3.59	1.5	1.95	0.14	492
2.76	1.06	1.64	0.06	50
4.42	1.46	2.77	0.19	50
3.08	1.14	1.82	0.12	114
3.98	0.96	2.86	0.16	549
3.72	1.35	2.2	0.17	47
4.26	1.46	2.77	0.03	78
3.63	1.73	1.7	0.2	85
5	1.31	3.5	0.19	303
3.84	1.63	2.09	0.12	131
5.2	1.54	3.55	0.11	1822
2.94	1	1.88	0.06	100
5.36	1.58	3.03	0.75	61
4.36	1.62	2.71	0.03	117
3.24	1.21	1.62	0.41	111
4.08	1.26	2.56	0.26	415
4.21	1.65	2.39	0.17	355
4.27	1.55	2.36	0.36	306
5.4	1.44	3.6	0.36	220
4.64	1.45	2.72	0.47	60
5.15	1.32	3.4	0.43	592
2.92	1.06	1.32	0.54	147
3.62	1.35	2.23	0.04	80
2.67	1.06	1.17	0.44	242
2.6	0.9	1.65	0.05	185
4.35	1.67	2.48	0.2	747
4.48	1.32	2.97	0.19	149
5.50	1.9	3.50	0.10	786
2.95	1.18	1.65	0.12	62
5.33	1.28	3.88	0.17	484
3.79	1.53	2.08	0.18	144
3.15	1.17	1.78	0.2	55
3.36	1.18	1.98	0.2	83
3.31	0.83	2.34	0.14	21
3.6	1.18	2.23	0.19	204
3.19	1.4	1.57	0.22	84
3.43	1.68	1.55	0.2	3
3.99	1.32	2.66	0.01	69
4.54	2.17	2	0.37	132
3.59	1.56	1.8	0.23	214
3.72	1.36	2.11	0.25	570
4.01	1.43	2.21	0.37	158

3.56	1.43	2.03	0.1	68
3.13	1.21	1.67	0.25	53
4.31	1.86	2.18	0.27	171
3.48	1.44	1.91	0.13	50
5.45	1.52	3.48	0.45	138
5	1.45	3.01	0.54	12
3.52	1.18	2.13	0.21	124
3.95	1.08	2.66	0.21	488
3.49	1.29	1.89	0.31	117
5.65	1.64	3.57	0.44	329
6.15	2.22	3.5	0.43	430
4.7	1.14	3.37	0.19	795
4.39	1.21	3.06	0.12	313
3.64	1.57	1.9	0.17	88
4.48	1.72	2.51	0.25	70
3.9	1.04	2.29	0.57	71
4.2	1.42	2.51	0.27	309
4.53	1.34	3.06	0.13	74
2.39	0.92	1.35	0.12	120
4.45	1.84	2.38	0.23	74
4.76	1.25	2.91	0.6	144
3.76	1.03	2.36	0.37	20
3.9	1.29	2.35	0.26	6
3.49	1.62	1.28	0.59	57
3.35	1.41	1.65	0.29	42
6.03	1.54	3.75	0.74	25
4.73	1.19	3.35	0.19	100
4.42	1.18	2.64	0.6	93
5.02	1.12	3.28	0.62	161
4.66	1.43	2.68	0.55	138
4.15	1.04	2.67	0.44	70
5.49	1.6	3.44	0.45	327
3.57	1.3	1.92	0.35	142
3.25	0.89	1.83	0.53	131
2.4	0.76	1.26	0.38	136
4.42	1.3	2.63	0.49	191
4.45	1.18	2.87	0.4	45
4.8	1.21	2.82	0.77	68
4.38	1.06	2.89	0.43	100
3.36	1.02	1.75	0.59	69
2.95	0.84	1.66	0.45	155
6.08	1.69	3.91	0.48	329
6.03	1.61	3.64	0.78	803
4.78	1.58	2.71	0.49	89
3.82	1.08	2.19	0.55	535
4.49	1.21	2.88	0.4	185
5.27	1.24	3.09	0.94	87
4.51	1.08	3.07	0.36	246

6.15	1.67	3.95	0.53	207
4.13	1.33	2.54	0.26	3
4.28	1.08	2.77	0.43	158
4.8	0.91	2.75	1.14	277
5.33	1.3	3.4	0.63	66
4.03	0.86	2.71	0.46	89
3.16	0.84	1.47	0.85	31
3.78	1.24	2.42	0.12	3
5.46	2.11	3.31	0.04	331
4.48	1.23	2.76	0.49	207
3.89	1.75	1.92	0.22	60
3.21	1.21	1.88	0.12	59
4.91	1.62	2.88	0.41	86
3.89	1.25	2.17	0.47	91
4.77	1.68	2.63	0.46	892
4.18	1.42	2.55	0.21	1011
4.96	1.87	2.62	0.47	133
4.55	1.8	2.44	0.31	719
4.92	1.58	3.09	0.25	353
3.14	1.57	1.33	0.24	100
4.84	1.65	2.96	0.23	549
3.44	1.29	2	0.15	45
3.11	0.89	2.11	0.11	31
3.99	1.18	2.4	0.41	80
4.2	1.16	2.29	0.75	368
5.03	1.36	3.16	0.51	347
2.66	0.6	1.91	0.15	62
5.85	2.16	3.24	0.45	3
3.23	1.11	1.98	0.14	201
4.29	1.47	2.4	0.42	333
3.48	1.15	2.17	0.16	256
3.82	1.5	2.05	0.27	230
5.72	1.67	3.68	0.37	377
4.54	1.5	2.56	0.48	188
4.08	1.59	2.29	0.2	120
3.66	1.63	1.65	0.38	344
3.65	1.07	2.38	0.2	181
4.59	2.01	2.21	0.37	635
3.91	1.25	2.6	0.06	29
3.99	1.48	2.16	0.35	64
5.13	1.52	3.38	0.23	928
4.2	1.21	2.64	0.35	166
3.84	1.2	2.31	0.33	462
3.96	1.33	2.38	0.25	151
3.93	0.93	2.43	0.57	203
4.76	1.27	3.17	0.32	227
6.67	1.55	4.37	0.75	158
6.36	1.93	3.95	0.48	106

3.99	1.48	2	0.51	181
4.39	1.3	2.68	0.41	130
4.36	0.98	2.92	0.46	204
5.15	1.58	3.24	0.33	58
4.47	0.95	2.95	0.57	181
5.47	1.29	3.67	0.51	145
6.25	1.26	4.68	0.31	183
3.73	0.93	2.07	0.73	136
4.51	1.14	2.72	0.65	121
4.37	1.23	2.43	0.71	267
5.23	1.45	3.31	0.47	82
7.52	1.2	4.84	1.48	158
4.89	0.88	3.49	0.52	227
3.39	1.47	1.81	0.11	90
5.88	1.47	2.93	1.48	47
4.75	1.33	2.77	0.65	269
4.86	1.17	2.81	0.88	92
5.8	1.44	3.7	0.66	3
4.9	0.99	1.62	2.29	73
6.2	1.6	3.2	1.4	56
4.83	1.09	2.68	1.06	67
4.53	0.89	2.94	0.7	124
4.88	1.53	2.77	0.58	43
4.47	1.03	2.66	0.78	70
3.82	1.87	1.77	0.18	189
4.59	1.6	2.79	0.2	110
3.74	1.5	2.15	0.09	354
3.71	1.2	2.39	0.12	202
4.37	0.98	3.3	0.09	226
3.99	1.25	2.63	0.11	101
3.61	1.35	2.05	0.21	52
5.83	2.07	3.68	0.08	193
4.33	2.09	2.08	0.16	189
5.38	1.48	3.41	0.49	635
4.27	1.32	2.49	0.46	276
3.99	1.02	2.4	0.57	71
6.03	1.6	4.01	0.42	264
2.61	0.93	1.53	0.15	31
2.96	1.52	1.39	0.05	320
3.62	1.52	2	0.1	283
4.4	1.53	2.67	0.2	61
2.63	1.42	1.05	0.16	112
4.35	1.13	3.1	0.12	487
5.48	1.33	4.07	0.08	267
4.37	1.3	3.00	0.07	105
4.77	1.88	2.27	0.62	29
3.83	1.42	2.06	0.35	136
3.92	1.55	2.21	0.16	171

3.78	1.43	1.97	0.38	59
5.73	1.78	3.52	0.43	330
4.32	1.58	2.35	0.39	190
4.79	1.56	2.97	0.26	61
3.54	1.09	1.94	0.51	158
3.37	1.22	2.06	0.09	23
2.74	1	1.54	0.2	268
3.93	1.32	2.42	0.19	134
3.63	1.19	2.39	0.05	138
3.84	1.31	2.37	0.16	252
5.84	1.87	3.57	0.4	169
3.47	1.09	2.01	0.37	87
4.03	1.1	2.58	0.35	3
5.21	1.71	3.14	0.36	190
4.61	1.41	2.82	0.38	133
5.25	1.15	3.64	0.46	61
2.71	1.36	1.21	0.14	139
4.43	1.46	2.61	0.36	107
4.47	1.06	3.23	0.18	223
5.54	1.27	3.89	0.38	342
3.67	0.79	2.27	0.61	110
3.71	1.41	1.88	0.42	311
5.44	1.39	3.45	0.6	114
3.53	0.73	2.04	0.76	218
5.03	1.43	2.85	0.75	241
4.09	1.01	2.73	0.35	157
4.86	1.27	2.65	0.94	104
4.36	1.47	2.59	0.3	295
3.85	1.11	2.35	0.39	173
4.07	1.38	2.42	0.27	240
4.56	0.96	2.85	0.75	204
5.08	1.59	2.89	0.6	260
5.15	1.32	3.33	0.5	3
4.21	1.09	2.77	0.35	125
4.1	0.82	1.97	1.31	41
5.39	1.27	2.75	1.37	246
3.21	1.54	1.21	0.46	121
3.48	1.48	1.65	0.35	103
4.03	1.37	2.61	0.05	130
3.98	1.64	1.93	0.41	46
3.29	1.4	1.48	0.41	29
3.28	1.13	2.01	0.14	49
4.42	2.08	1.99	0.35	163
4.66	1.41	2.96	0.29	62
3.93	1.42	2.06	0.45	237
3.81	1.3	2.47	0.04	774
5	1.56	3.02	0.42	150
2.83	1.14	1.48	0.21	140

3.58	1.56	1.76	0.26	263
4.37	1.25	2.66	0.46	722
4.34	1.59	2.22	0.53	39
4.36	1.29	2.83	0.24	215
4.43	1.7	2.24	0.49	60
6.2	1.53	3.96	0.71	916
4.8	1.19	3.53	0.08	116
4.88	1.6	3.03	0.25	331
3.87	1.29	2.39	0.19	97
4.16	1.31	2.34	0.51	67
3.37	1.18	1.87	0.32	3
4.7	1.4	2.76	0.54	16
5.5	1.33	3.83	0.34	44
4.52	1.71	2.45	0.36	116
4.62	1.62	2.61	0.39	35
4.5	1.64	2.42	0.44	186
3.91	1.19	2.35	0.37	261
3.16	0.91	1.91	0.34	77
4.21	1.49	2.53	0.19	214
3.85	1.16	2.23	0.46	287
5.29	1.51	2.9	0.88	52
4.24	1.49	2.24	0.51	59
3.29	1.01	1.75	0.53	91
4.53	1.33	2.5	0.7	148
4.91	1.2	3.2	0.51	159
4.49	1.28	2.57	0.64	138
3.89	0.89	2.65	0.35	142
6.76	1.88	4.16	0.72	291
5.65	1.64	2.99	1.02	308
4.67	1.17	3.21	0.29	115
3.85	1.11	2.29	0.45	44
6.22	1.49	4.09	0.64	459
4.03	1.07	2.36	0.6	145
4.44	1.3	2.61	0.53	34
4.83	0.99	2.63	1.21	219
5.96	1.09	3.42	1.45	142
4.91	1.03	2.46	1.42	3
5.3	1.64	3.51	0.15	73
4.25	1.34	2.77	0.14	264
3.46	1.15	2.19	0.12	42
6.16	1.73	4.4	0.03	168
3.86	1.78	1.79	0.29	200
3.48	1.19	2.16	0.13	82
5.39	1.94	2.56	0.89	167
4.87	1.21	3.29	0.37	221
4.19	1.04	2.68	0.47	71
5.16	1.77	3.26	0.13	139
4.86	1.28	2.94	0.64	436

5.81	1.36	3.87	0.58	183
4.01	1.36	2.57	0.08	105
3.65	1.48	2.15	0.02	102
4.4	1.32	2.96	0.12	159
4.76	1.88	2.51	0.37	107
3.17	1.27	1.81	0.09	14
3.46	1.59	1.68	0.19	404
3.59	1.26	2.01	0.32	3
4.5	1.37	2.87	0.26	295
4.29	1.17	2.87	0.25	91
3.62	0.96	2.3	0.36	167
4.76	1.37	3.02	0.37	281
4.23	0.98	2.79	0.46	109
4.84	1.06	3.1	0.68	522
3.98	1.14	2.71	0.13	368
3.27	1.06	1.94	0.27	79
4.88	1.62	2.8	0.46	612
5.01	1.89	2.39	0.73	81
5.67	1.79	3.5	0.38	107
4.57	1.97	2.2	0.4	604
3.23	1.41	1.79	0.03	132
2.98	0.7	2.17	0.11	32
4.64	1.3	2.86	0.48	65
3.32	1.35	1.65	0.32	229
4.62	1.52	2.8	0.3	129
4.78	1.36	3.07	0.35	510
3.73	1.32	1.91	0.5	54
5.46	1.27	3.87	0.32	308
5.46	1.37	3.83	0.26	135
3.41	1.19	1.9	0.32	125
3.66	1.04	2.23	0.39	195
4.48	1.25	2.76	0.47	141
2.96	1.13	1.45	0.38	3
5.27	1.53	3.1	0.64	179
4.71	1.22	2.62	0.87	115
5.42	2.07	2.98	0.37	54
6.15	1.31	3.94	0.9	134
3.56	0.87	2.21	0.48	82
4.5	1.02	2.6	0.88	57
3.64	1.3	2.24	0.1	314
3.91	1.46	2.26	0.19	221
3.35	1.25	2.08	0.02	79
4.07	1.23	2.5	0.34	19
3.96	1.1	2.23	0.63	37
3.45	1.21	2.09	0.15	103
3.01	1.62	1.22	0.17	371
4.28	1.76	2.33	0.19	3
4.98	1.91	2.42	0.65	130

3.38	1.56	1.55	0.27	40
4.33	1.34	2.55	0.44	38
4.46	1.53	2.07	0.86	97
3.69	1.46	1.93	0.3	144
2.96	1.19	1.41	0.36	90
3.68	1.29	2.26	0.13	20
3.27	1.23	1.64	0.4	106
3.12	1.24	1.67	0.21	3
3.9	1.39	2.15	0.36	314
3.55	1.34	1.9	0.31	3
4.81	1.55	2.8	0.46	151
3.81	1.48	2.00	0.33	75
3.93	1.46	2.24	0.23	154
3.31	1.22	1.84	0.25	97
5.52	1.62	3.5	0.4	198
4.33	1.35	2.33	0.65	40
5.33	1.54	3.41	0.38	3
5.14	1.11	3.58	0.45	326
3.72	0.97	2.32	0.43	226
5.65	1.38	3.7	0.57	50
3.48	1.35	1.91	0.22	45
6.67	1.3	4.69	0.68	38
4.83	1.28	3	0.55	560
4.61	1.09	2.68	0.84	111
3.99	1.15	2.39	0.45	77
5.13	1.88	2.88	0.37	98
4.76	1.22	2.92	0.62	446
3.70	1.5	2.01	0.19	54
3.15	1.4	1.64	0.11	16
4.48	1.49	2.78	0.21	137
5.96	2.38	3.26	0.32	116
4.58	1.87	2.26	0.45	72
4.27	1.53	2.65	0.09	91
3.66	1.31	2.23	0.12	166
3.96	1.54	2.21	0.21	142
2.85	0.91	1.66	0.28	161
5.74	1.88	3.52	0.34	345
3.99	1.04	2.77	0.18	39
4.59	1.46	2.48	0.65	129
3.95	1.22	2.24	0.49	69
4.65	1.44	2.82	0.39	43
5	1.58	2.89	0.53	103
4.14	1.58	2.33	0.23	253
2.4	0.77	1.42	0.21	126
3.62	1.41	1.89	0.32	76
5.15	1.39	3.37	0.39	125
4.98	1.13	2.99	0.86	324
3.78	1.31	2.15	0.32	20

3.14	1.16	1.46	0.52	400
5.15	1.17	3.37	0.61	113
3.83	0.87	2.38	0.58	86
5.82	1.73	3.33	0.76	962
3.96	0.99	2.11	0.86	529
4.47	1.29	2.52	0.66	55
5.38	1.66	3.17	0.55	549
3.97	1	2.23	0.74	305
4.98	1.39	3.03	0.56	187
4.9	1.17	2.67	1.06	94
2.95	1.13	1.65	0.17	77
3.43	1.33	1.9	0.2	73
5.46	1.83	3.55	0.08	390
3.7	1.47	2.15	0.08	201
3.77	1.16	2.34	0.27	211
3.08	1.3	1.54	0.24	116
4.63	1.67	2.55	0.41	3
4.78	1.4	3.3	0.08	122
2.85	1.47	1.28	0.1	3
3.72	1.42	2.14	0.16	92
4.59	1.7	2.61	0.28	444
3.72	1.04	2.2	0.48	95
4.8	1.53	3.19	0.08	445
3.86	0.97	2.58	0.31	172
4.21	1.29	2.68	0.24	260
3.2	0.94	1.92	0.34	130
4.61	1.67	2.58	0.36	153
5.62	1.14	3.89	0.59	275
3.7	1.21	2.03	0.46	30
4.23	1.4	2.53	0.3	100
4.05	1.38	2.2	0.47	153
4.41	1.2	2.83	0.38	91
4.67	1.49	2.53	0.65	773
5	1.7	3.14	0.16	40
4.5	1.1	2.47	0.93	233
2.95	0.79	1.37	0.79	61
5.38	1.01	2.99	1.38	28
5.11	2.18	2.42	0.51	394
4.99	1.26	3.57	0.16	221
5.04	1.86	2.91	0.27	358
2.8	0.98	1.39	0.43	133
4.3	1.39	2.08	0.83	515
4.48	1.42	2.63	0.43	44
5.34	1.16	3.55	0.63	118
5.8	1.31	3.69	0.8	35
5.67	1.85	2.97	0.85	104
3.83	1.29	2.04	0.5	118
5.45	1.54	3.71	0.2	256

4.32	1.41	2.73	0.18	23
2.96	1.48	1.33	0.15	131
4.5	1.77	2.55	0.18	191
3.9	1.22	2.62	0.06	577
3.7	1.64	1.89	0.17	180
3.78	1.06	2.53	0.19	699
5.75	1.3	3.3	1.15	172
4.54	1.45	2.85	0.24	292
3.78	1.85	1.71	0.22	90
5.03	1.65	2.87	0.51	551
3.72	1.38	2.09	0.25	40
3.29	1.33	1.72	0.24	134
4.31	2.02	1.91	0.38	54
3.4	1.32	1.66	0.42	46
2.88	1.08	1.3	0.5	117
3.92	1.14	2.39	0.39	99
5.96	1.79	3.79	0.38	180
3.68	1.46	1.94	0.28	390
2.67	1.07	1.33	0.27	48
5.38	1.9	3.15	0.33	251
3.77	1.07	2.39	0.31	82
6.26	1.76	4.14	0.36	339
4.52	1.4	2.74	0.38	157
4.09	1.33	2.46	0.3	155
4.76	1.14	2.95	0.67	536
6.09	1.32	3.86	0.91	492
3.29	1.16	1.57	0.56	201
3.02	0.9	1.89	0.23	202
4.46	1.22	3	0.24	63
5.18	1.52	3.07	0.59	173
4.18	1.55	2.08	0.55	276
5.39	1.07	3.83	0.49	487
4.23	1.3	2.56	0.37	101
4.42	1.15	2.82	0.45	146
5.26	1.15	3.72	0.39	555
5.58	1.44	3.77	0.37	781
4.29	1.04	2.75	0.5	144
5.31	1.53	3.46	0.32	169
5.24	1.26	3.34	0.64	751
4.88	1.15	3.52	0.21	40
5.36	1.42	3.53	0.41	180
4.3	1.02	2.4	0.88	115
4.44	1.09	2.59	0.76	217
6.15	1.39	4.16	0.6	164
4.68	1.06	2	1.62	536
4.27	2.16	1.57	0.54	186

4.2	1.66	1.97	0.57	353
3.78	1.34	2.18	0.26	487
4.45	1.74	2.43	0.28	104
3.71	1.33	2.08	0.3	325
4.59	1.68	2.33	0.58	549
3.68	1.47	1.64	0.57	194
4.48	1.6	2.5	0.38	138
3.63	1.03	2.27	0.33	83
3.81	1.19	2.34	0.28	80
3.79	1.2	2.3	0.29	371
3.65	1.22	2.17	0.26	43
3.8	1.57	1.91	0.32	79
6.34	1.86	3.6	0.88	151
3.96	1.44	2.15	0.37	95
5.25	1.61	3.24	0.4	153
3.73	1.27	2.21	0.25	533
4.19	1.68	2.07	0.44	749
4.89	1.33	3.34	0.22	421
3.67	1.49	1.93	0.25	109
5.49	2.09	2.9	0.5	812
5.75	1.36	3.86	0.53	136
4.72	1.51	2.87	0.34	43
4.98	2.06	2.48	0.44	41
3.94	1.52	2.03	0.39	128
3.76	1.31	1.92	0.53	25
5.33	1.53	2.61	1.19	193
4.26	1.75	2.14	0.37	54
4.51	1.35	2.9	0.26	176
3.98	1.27	2.39	0.32	373
4.35	1.18	2.91	0.26	69
5.05	1.62	3.06	0.37	19
4.84	1.5	2.93	0.41	842
3.65	1.16	2.09	0.4	208
4.19	1.05	2.36	0.78	198
5.19	1.55	3.35	0.29	258
4.23	1.37	2.62	0.24	56
3.4	1.48	1.64	0.28	141
3.88	1.11	2.18	0.59	256
5.51	1.75	3.02	0.74	69
3.77	1.06	2.41	0.3	79
4.15	1.18	2.3	0.67	150
5.1	1.68	3.1	0.32	130
4.54	1.25	2.84	0.45	362
4.52	1.15	2.92	0.45	572
4.86	1.42	2.9	0.54	46
5.37	1.27	3.75	0.35	854

6.04	1.45	3.75	0.84	126
4.29	1.1	2.43	0.76	638
6.22	1.86	4.03	0.33	958
5.83	1.49	3.29	1.05	236
4.21	1.36	2.62	0.23	253
5.58	1.77	3.32	0.49	263
5.79	1.56	3.48	0.75	261
3.64	1.09	2.23	0.32	144
6.4	1.21	4.55	0.64	69
4.17	1.18	2.45	0.54	137
4.69	1.01	2.95	0.73	87
5.81	1.16	3.55	1.1	116
4.43	2.17	1.85	0.41	283
4.17	2.21	1.64	0.32	102
3.64	1.62	1.71	0.31	25
5.44	2.31	2.75	0.38	129
3.72	1.98	1.5	0.24	273
4.05	1.41	1.99	0.65	284
4.09	1.76	1.97	0.36	3
3.62	1.52	1.81	0.29	195
4.77	1.47	3	0.3	56
5.56	1.43	3.62	0.51	81
5.71	1.54	3.63	0.54	182
3.37	1.33	1.71	0.33	65
3.75	1.45	1.75	0.55	100
4.47	1.82	2.43	0.22	116
4.09	1.34	1.94	0.81	486
4.65	1.5	2.69	0.46	161
3.53	1.54	1.71	0.28	175
4.02	1.56	2.14	0.32	79
4.34	1.94	1.99	0.41	91
4.03	1.74	1.93	0.36	120
4.18	2.37	1.11	0.7	77
5.43	1.84	3.27	0.32	353
3.68	1.6	1.77	0.31	55
3.85	1.47	2	0.38	70
4.38	1.39	2.76	0.23	100
4.36	1.19	2.91	0.26	77
3.76	1.31	2.1	0.35	126
3.58	1.25	2.12	0.21	237
5.61	1.4	3.63	0.58	71
5.17	2.02	2.85	0.3	317
6.19	1.81	3.94	0.44	495
4.19	1.64	2.25	0.3	121
4.99	1.81	2.62	0.56	205
4.58	1.31	2.95	0.32	55
3.96	1.77	1.92	0.27	112
4.67	1.61	2.65	0.41	111

5.17	1.83	3.09	0.25	136
3.25	1.22	1.71	0.32	247
5.45	1.65	3.35	0.45	282
5.27	1.51	3.03	0.73	567
4.96	1.68	2.92	0.36	125
3.64	1.28	2.04	0.32	3
4.98	1.41	2.73	0.84	185
4.82	1.55	2.95	0.32	93
4.67	1.8	2.59	0.28	51
5.17	1.16	3.72	0.29	206
4.83	1.76	2.56	0.51	83
4.11	1.5	2.15	0.46	341
6.46	3.9	1.77	0.79	65
3.85	1.08	2.43	0.34	113
5.74	1.68	3.62	0.44	279
4.25	1.34	2.34	0.57	37
3.74	1.29	2.11	0.34	76
5.79	1.45	3.91	0.43	178
4.43	1.08	3	0.35	262
5.09	1.33	3.27	0.49	414
4.05	1.45	2.37	0.23	346
4.25	1.12	2.71	0.42	301
4.46	1.41	2.7	0.35	142
4.36	1.51	2.6	0.25	491
3.58	1.27	2.08	0.23	142
4.76	1.3	3.02	0.44	184
3.9	1.25	2.39	0.26	261
3.74	1.23	1.92	0.59	140
4.2	1.48	2.25	0.47	45
4.75	1.48	3.06	0.21	93
3.53	1.1	1.97	0.46	29
4.28	1.14	2.57	0.57	232
4.09	1.2	2.68	0.21	233
4.28	1.34	2.56	0.38	125
3.78	1.11	2.26	0.41	172
4.86	1.19	3.09	0.58	3
5.16	1.29	3.54	0.33	240
3.65	1.02	2.23	0.4	136
4.86	1.31	2.75	0.8	602
3.46	1.35	1.8	0.31	74
4.4	1.12	2.76	0.52	228
3.72	0.99	2.3	0.43	467
3.84	1.08	2.31	0.45	135
5.52	1.33	3.72	1.47	131
4.86	1.29	2.73	0.84	77
3.46	1.34	1.62	0.5	76
4.41	1.09	2.7	0.62	87
4.49	1.22	2.86	0.41	77

5.4	1.66	3.15	0.59	355
4.99	1.36	2.92	0.71	310
4.51	1.19	3.1	0.22	469
5.97	1.47	3.5	1	94
4.43	1.1	2.75	0.58	116
4.43	2.07	1.78	0.58	250
7.61	1.22	5.37	1.02	239
4.3	2.02	1.86	0.42	80
3.83	1.35	2.16	0.32	68
6.29	1.41	3.78	1.1	184
4.27	1.32	2.59	0.36	498
4.92	1.03	2.83	1.06	379
4.28	1.06	2.66	0.56	401
4.7	1.32	2.81	0.57	197
4.08	0.95	2.67	0.46	76
4.6	1.11	2.84	0.65	521
3.93	1.17	1.97	0.79	32
4.33	0.98	2.82	0.53	119
3.9	0.97	2.34	0.59	277
5.66	1.87	3.18	0.61	45
4.28	1.3	2.36	0.62	85
4.96	1.41	3.16	0.39	3
6.72	1.83	4.44	0.45	94
7.26	1.68	4.86	0.72	134
4.36	1.07	2.73	0.56	364
7.65	1.61	5.61	0.43	115
5.24	1.2	3.64	0.4	282
2.87	0.93	1.42	0.52	14
6.17	1.38	4.16	0.63	363
4.4	1.33	2.68	0.39	33
6.08	1.15	4.18	0.75	208
3.95	0.89	2.68	0.38	597
5.57	1.31	3.68	0.58	729
5.87	1.5	3.58	0.79	72
5.2	1.04	3.4	0.76	100
5.03	1.09	3.34	0.6	134
4.09	1.11	2.41	0.57	93
5.98	1.25	3.59	1.14	260
4.24	0.88	2.81	0.55	249
4.57	0.99	1.97	1.61	44
6.08	1.53	3.56	0.99	170
5.57	0.84	2.38	2.35	158
5.02	0.95	3.11	0.96	107
4.35	2.07	1.99	0.29	32
2.99	1.18	1.54	0.27	52
3.39	1.18	1.87	0.34	217
3.37	1.49	1.4	0.48	177
4.39	1.9	2.19	0.3	270

4.47	1.85	2.2	0.42	153
2.51	1.33	0.77	0.41	12
4.13	1.36	2.51	0.26	53
3.92	1.23	2.31	0.38	76
6.38	2.44	3.52	0.42	35
5.18	2.2	2.5	0.48	333
2.99	1.45	1.2	0.34	149
5.32	2.51	2.36	0.45	131
4.59	1.71	2.55	0.33	174
4.4	1.05	3.05	0.3	207
4.4	1.28	2.82	0.3	706
5.28	1.66	3.1	0.52	21
4.06	1.52	2.26	0.28	104
3.96	1.5	2.13	0.33	30
4.04	1.7	2.02	0.32	69
3.88	1.57	1.92	0.39	134
4.28	1.92	1.79	0.57	104
4.35	1.47	2.57	0.31	192
4.97	1.67	2.9	0.4	86
3.81	1.35	1.97	0.49	99
5.05	1.84	2.71	0.5	478
3.72	1.54	1.94	0.24	68
3.29	1.55	1.39	0.35	123
4.21	1.4	2.47	0.34	325
4.16	1.16	2.67	0.33	155
4.19	1.73	2.11	0.35	235
4.04	1.3	2.12	0.62	117
4.82	2	2.1	0.72	618
4.83	2.21	2.23	0.39	99
5.61	2.22	2.97	0.42	122
4.42	1.41	2.68	0.33	192
3.91	1.13	2.48	0.3	350
4.07	1.93	1.81	0.33	349
4.84	1.22	3.19	0.43	221
5.15	1.64	3.06	0.45	349
5	1.86	2.9	0.24	223
4.68	2.04	1.89	0.75	64
4.51	1.34	2.7	0.47	168
3.89	1.52	2.13	0.24	116
3.66	1.99	1.19	0.48	63
3.3	1.4	1.67	0.23	157
3.44	1.09	1.76	0.59	103
3.5	1.22	2.03	0.25	625
3.4	1.36	1.77	0.27	83
4.65	1.58	2.83	0.24	137
5.42	1.53	3.25	0.64	210
4.48	1.64	2.63	0.21	157
2.49	0.94	1.28	0.27	432

4.62	1.13	3.11	0.38	356
3.36	1.41	1.73	0.22	180
5.07	1.5	3.34	0.23	122
3.53	1.06	2.06	0.41	342
3.66	1.43	1.93	0.3	110
5.5	1.81	3.33	0.36	503
4.73	1.8	2.66	0.27	48
4.05	1.33	2.04	0.68	91
3.38	1.69	1.35	0.34	31
4.27	1.44	2.48	0.35	140
4.04	1.44	2.33	0.27	281
3.46	1.46	1.67	0.33	81
4.59	1.72	2.52	0.35	712
3.35	1.11	2.01	0.23	44
4.05	1.26	2.26	0.53	259
3.69	1.25	2.01	0.43	541
4.05	1.4	2.36	0.29	137
5.41	1.53	3.61	0.27	60
4.96	2.16	2.33	0.47	6
3.66	1.71	1.6	0.35	154
5.66	1.86	3.41	0.39	125
4.58	1.4	2.87	0.31	443
4.09	1.24	2.39	0.46	218
4.46	1.62	2.47	0.37	535
3.79	1.46	2.05	0.28	111
5.21	1.65	3.09	0.47	50
3.51	0.98	2.08	0.45	66
5.6	1.64	3.19	0.77	130
4.02	1.07	2.6	0.35	50
4.28	1.38	2.37	0.53	531
3.19	1.7	1.02	0.47	96
4.25	1.24	2.73	0.28	163
3.56	1.21	2.1	0.25	166
4.2	1.49	2	0.71	111
3.25	1.32	1.71	0.22	3
6.01	1.77	3.81	0.43	190
3.96	1.71	1.75	0.50	90
3.83	1.33	2.15	0.35	491
3.81	1.61	1.86	0.34	112
4.31	1.38	2.35	0.58	529
5.31	1.67	3.38	0.26	188
4.8	1.71	2.6	0.49	27
3.9	1.4	2.15	0.35	301
5.21	1.95	2.29	0.97	120
4.56	1.61	2.65	0.3	71
3.46	1.21	1.77	0.48	46
4.84	1.45	2.95	0.44	155
5.44	1.94	3.1	0.4	82

4.08	0.97	2.55	0.56	201
4.37	1.38	2.46	0.53	116
4.7	1.27	3.11	0.32	344
4.9	1.57	3.04	0.29	100
4.6	1.72	2.56	0.32	103
4.52	1.57	2.51	0.44	75
5.37	1.54	3.07	0.76	210
4.82	1.63	2.79	0.4	63
5.09	1.81	2.75	0.53	305
3.11	1.06	1.75	0.3	291
5.32	1.4	3.33	0.59	1061
4.68	1.64	2.82	0.22	28
4.67	1.3	2.99	0.38	86
4.08	0.88	2.56	0.64	317
3.03	0.91	1.89	0.23	3
3.89	1.15	2.41	0.33	170
5.34	1.37	3.6	0.37	101
4.5	1.41	2.69	0.4	89
5.69	2	3.17	0.52	904
3.61	1.14	2.21	0.26	133
4.77	1.1	3.24	0.43	348
5.28	1.33	3.46	0.49	174
5.05	1.57	3.08	0.4	153
5.29	1.45	3.63	0.21	504
4.83	1.23	3.36	0.24	453
4.72	1.51	2.82	0.39	378
4.38	1.22	2.83	0.41	459
5.11	1.28	3.12	0.71	140
3.78	1.37	2.03	0.38	3
4.43	1.85	2.01	0.57	164
3.57	1.07	2.04	0.46	19
3.8	1.05	2.52	0.23	226
5.19	2.09	2.88	0.22	310
4.69	1.27	3.13	0.29	68
6.52	1.48	4.27	0.77	133
4.35	1.31	2.42	0.62	361
4.52	1.58	2.55	0.39	298
3.91	1.48	2.05	0.38	96
3.74	1.29	1.88	0.57	133
8	1.51	5.77	0.72	784
2.97	1.25	1.51	0.21	27
6.92	1.64	4.23	1.05	131
5.15	1.19	3.31	0.65	484
3.96	1.22	2.23	0.51	47
4.15	1.24	2.66	0.25	94
3.27	1.24	1.79	0.24	154
3.55	1.26	1.81	0.48	313
6.4	2.2	3.25	0.95	97

3.52	0.94	2.05	0.53	146
3.41	0.87	2.15	0.39	217
4.87	1.62	2.85	0.4	228
4	1.38	2.01	0.61	772
4.76	1.13	3.34	0.29	701
4.28	0.84	3.07	0.37	41
4	1.26	2.37	0.37	28
5.12	1.74	3.01	0.37	165
4.37	1.31	2.75	0.31	75
4.1	1.14	2.4	0.56	127
3.55	0.93	2.06	0.56	153
5.02	1.63	3.14	0.25	71
4.28	1	2.86	0.42	47
4.84	1.24	3.19	0.41	46
6.04	1.46	3.82	0.76	149
4.51	1.55	2.17	0.79	118
4.48	1.39	2.51	0.58	64
4.01	1.66	2.04	0.31	163
5.17	1.51	3.1	0.56	400
4.43	1.27	2.82	0.34	205
4.02	1.05	2.58	0.39	219
4.05	1.58	2.11	0.36	63
3.89	1.35	1.55	0.99	453
4.73	1.24	3.04	0.45	191
4.7	0.92	3.43	0.35	471
4.97	2.07	2.63	0.27	276
5.17	1.49	3.34	0.34	428
4.85	1.32	3.22	0.31	119
3.92	1.16	2.29	0.47	1068
4.84	1.33	3.06	0.45	300
2.71	0.82	1.36	0.53	58
4.38	1.26	2.86	0.26	216
6.22	2.17	3.42	0.63	131
4	1.46	2.2	0.34	63
4.56	1.43	2.5	0.63	9
4.46	1.11	2.57	0.26	925
3.83	1.6	1.82	0.41	3
5.05	1.76	3.03	0.26	117
4.09	1.31	2.3	0.48	121
4.36	1.08	2.69	0.59	60
4.38	1.36	2.7	0.32	238
3.55	1.3	1.73	0.52	40
3.84	1.04	2.44	0.36	324
4.55	1.37	2.8	0.38	97
3.07	0.85	1.81	0.41	98
5.38	1.41	3.48	0.49	624
5.37	1.29	3.26	0.82	73
4.5	1.24	2.61	0.65	622

4.49	1.16	2.97	0.36	200
5.74	1.23	4.09	0.42	406
3.77	1.29	2.19	0.29	43
4.13	1.19	2.49	0.45	87
5.52	1.43	3.64	0.45	327
4.25	0.89	2.83	0.53	140
5.42	1.47	3.42	0.53	293
4.48	1.23	2.77	0.48	490
3.39	1.08	1.74	0.57	11
4.05	1.09	2.28	0.68	271
5.05	1.39	3.26	0.4	48
4.69	1.3	3.04	0.35	148
4.25	1.21	2.58	0.46	279
4.07	1.29	2.41	0.37	97
4.69	1.64	2.69	0.36	46
4.71	1.58	2.64	0.49	126
4	0.92	2.52	0.56	204
3.62	1.3	1.88	0.44	155
7.15	1.63	4.45	1.07	239
3.54	1.22	2.07	0.25	172
5.25	1.15	3.54	0.56	344
4.67	1.23	3.06	0.38	745
5.67	1.12	4.07	0.48	397
4.37	1.16	2.77	0.44	215
4.27	1.27	2.56	0.44	314
4.35	1.06	2.74	0.55	176
5.49	1.03	3.79	0.67	52
3.8	1.21	2.23	0.36	94
3.87	0.99	1.75	1.13	147
5.8	1.25	3.75	0.8	294
3.69	0.89	2.42	0.38	99
5.3	1.24	3.54	0.52	177
5.41	1.51	3.3	0.6	136
7.48	0.95	2.96	3.57	68
4.05	2.13	1.35	0.57	3
5.87	1.69	3.22	0.96	213
4.83	1.32	2.91	0.6	294
4	1.23	2.19	0.58	42
4.84	1.11	3.21	0.52	546
5.07	1.29	3.27	0.51	321
3.5	1.13	2.07	0.3	46
4.75	1.2	2.91	0.64	98
4.04	1.34	2.46	0.24	108
5.36	1.53	3.41	0.42	142
6.14	1.42	3.79	0.93	133
5.08	1.31	3.42	0.35	137
5.11	1.1	3.22	0.79	380
5.5	1.87	2.95	0.68	115

3.93	1.24	2.47	0.22	112
3.58	1.19	2.16	0.23	82
4.03	1.53	2.12	0.38	128
3.54	1.22	1.55	0.77	8
4.08	0.91	2.75	0.42	119
5.93	1.52	3.56	0.85	321
4.62	1.11	2.87	0.64	666
6.08	1.18	3.77	1.13	40
4.89	1.59	2.55	0.75	187
5.11	1.26	3.41	0.44	446
4.86	1.21	3.18	0.47	63
5.18	1.39	3.27	0.52	85
4.66	1.08	3.21	0.37	72
4.31	1.05	2.72	0.54	98
4.39	1.13	2.5	0.76	104
3.29	1.22	1.8	0.27	132
5.47	1.05	3.66	0.76	175
4.32	1	2.84	0.48	87
3.9	0.83	2.56	0.51	308
4.61	1.4	2.56	0.65	40
4.3	1.2	2.55	0.55	87
3.48	0.82	2	0.66	91
3.76	0.83	2.29	0.64	194
5.2	1.38	3.16	0.66	423
3.51	1.15	1.95	0.41	319
5.06	1.03	3.61	0.42	663
5.11	1.04	3.42	0.65	425
5.68	1.67	2.81	1.2	861
4.33	0.82	3.05	0.46	257
5.51	1.04	3.89	0.58	186
3.94	1.02	2.37	0.55	47
3.64	0.94	1.98	0.72	11
3.37	1.07	1.6	0.7	185
4.58	1.34	2.76	0.48	97
6.95	2.16	4.13	0.66	148
4.12	1.07	2.63	0.42	584
4	0.93	2.62	0.45	104
4.25	1.23	2.77	0.25	303
5.07	1.18	3.28	0.61	180
4.95	1.26	2.62	1.07	326
5.06	1.34	3	0.72	104
5.8	1.64	3	1.16	74
5.95	1.18	4.14	0.63	47
3.94	0.93	2.33	0.68	206
4.21	0.98	2.63	0.6	414
3.94	0.83	2.01	1.1	47
3.76	0.58	1.86	1.32	252
4.43	1.03	2.7	0.7	3

5.84	1.38	3.86	0.6	103
3.98	0.88	2.25	0.85	46
3.31	0.67	1.35	1.29	19
3.64	1.02	1.6	1.02	3
4.87	0.72	1.57	2.58	131
3.36	0.74	1.08	1.54	11
4.51	1.05	2.17	1.29	3
