# Supplementary material for: Correlation between inflammatory marker and lipid metabolism in patients with uterine leiomyomas
Source: Front Med (Lausanne). 2023 May 3;10:1124697. doi: 10.3389/fmed.2023.1124697 (PMC10189017; doi:10.3389/fmed.2023.1124697)
Supplement: Supplementary file 2 [file Data_Sheet_2.pdf]

XH	PEID	AGE	HIGH	WEIGHT	HIGH1	BMI	PLT
1	85648885	49	149	47.8	1.49	21.53	259
2	85986234	40	151.5	41.9	1.52	18.26	310
3	88370384	46	152	51.5	1.52	22.29	308
4	88360917	47	153.5	52.1	1.54	22.11	231
5	88363280	46	153.5	54.6	1.54	23.17	303
6	88343723	44	154	59.6	1.54	25.13	334
7	88366378	42	155	47.4	1.55	19.73	281
8	88269689	49	155	49.4	1.55	20.56	248
9	88311850	47	155	70.5	1.55	29.34	278
10	88363297	48	155.5	49.6	1.56	20.51	257
11	88367032	50	155.5	50.6	1.56	20.93	259
12	88370403	47	155.5	62.7	1.56	25.93	226
13	88363319	50	155.5	71.2	1.56	29.45	358
14	88343800	47	156	46.2	1.56	18.98	219
15	88269682	44	156	46.3	1.56	19.03	206
16	88369973	47	156	50	1.56	20.55	286
17	88366706	41	156	50.5	1.56	20.75	255
18	88367555	49	156	57.5	1.56	23.63	323
19	88421882	46	156	59	1.56	24.24	326
20	88363298	49	156	60.1	1.56	24.70	277
21	88363300	49	156	69	1.56	28.35	277
22	88361729	47	156.5	51.5	1.57	21.03	202
23	88343770	47	156.5	52.4	1.57	21.39	187
24	88343711	50	156.5	53.4	1.57	21.80	271
25	88343790	44	156.5	54.4	1.57	22.21	203
26	88370238	45	156.5	57.8	1.57	23.60	156
27	88421729	46	156.5	72.4	1.57	29.56	342
28	88343732	48	156.5	74	1.57	30.21	394
29	88370255	46	157	54.1	1.57	21.95	327
30	88343765	41	157	54.6	1.57	22.15	409
31	88343717	42	157	60.7	1.57	24.63	269
32	88343755	48	157	63.4	1.57	25.72	233
33	88367335	46	157	64.1	1.57	26.01	308
34	88343795	42	157	75.6	1.57	30.67	218
35	88343775	47	157.5	50.3	1.58	20.28	292
36	88370481	44	157.5	51.7	1.58	20.84	240
37	88366824	45	157.5	51.9	1.58	20.92	227
38	88343782	41	157.5	55	1.58	22.17	269
39	88363056	50	157.5	59.9	1.58	24.15	294

40	88343773	46	157.5	63.1	1.58	25.44	239
41	88370350	46	157.5	72.8	1.58	29.35	271
42	88363307	50	158	52.3	1.58	20.95	287
43	88370533	45	158	53.4	1.58	21.39	259
44	88385776	42	158	57	1.58	22.83	398
45	88363306	50	158	59.9	1.58	23.99	273
46	88343757	46	158	61.6	1.58	24.68	214
47	88370591	50	158	73.9	1.58	29.60	427
48	88365734	50	158	76	1.58	30.44	433
49	88343809	50	158.5	50.4	1.59	20.06	227
50	88421838	44	158.5	52.2	1.59	20.78	123
51	88167633	46	158.5	55	1.59	21.89	405
52	88363288	47	158.5	56.3	1.59	22.41	267
53	88367336	46	158.5	56.3	1.59	22.41	240
54	88366810	45	158.5	56.7	1.59	22.57	295
55	88279924	42	158.5	62.9	1.59	25.04	351
56	88370608	46	158.5	63.5	1.59	25.28	281
57	88343720	47	158.5	64.4	1.59	25.63	319
58	88343799	40	159	44.5	1.59	17.60	219
59	88343774	44	159	47.6	1.59	18.83	245
60	88343764	47	159	49.4	1.59	19.54	241
61	88343805	45	159	50.3	1.59	19.90	199
62	88367556	42	159	50.8	1.59	20.09	277
63	88370229	43	159	54.4	1.59	21.52	327
64	88421875	40	159	56.8	1.59	22.47	392
65	88370365	46	159	60	1.59	23.73	300
66	88303860	47	159	63.1	1.59	24.96	229
67	88370228	46	159	63.2	1.59	25.00	258
68	88370341	49	159	65.8	1.59	26.03	231
69	88367330	43	159.5	48.8	1.60	19.18	257
70	88421826	40	159.5	51.5	1.60	20.24	265
71	88366404	49	159.5	53.1	1.60	20.87	260
72	88343734	44	159.5	54.1	1.60	21.27	245
73	88343817	48	159.5	54.6	1.60	21.46	276
74	88370799	50	159.5	61.8	1.60	24.29	248
75	88370414	50	159.5	62.8	1.60	24.69	215
76	88363292	46	159.5	64.9	1.60	25.51	272
77	88370480	41	160	45	1.60	17.58	234
78	88366484	45	160	50	1.60	19.53	216
79	88370551	46	160	52	1.60	20.31	245

80	88363313	49	160	52.3	1.60	20.43	190
81	88367332	44	160	62.7	1.60	24.49	247
82	88343735	44	160	63.9	1.60	24.96	234
83	88366839	43	160	84.6	1.60	33.05	407
84	88343792	49	160.5	52.3	1.61	20.30	247
85	88366384	48	160.5	52.5	1.61	20.38	220
86	88421706	48	160.5	53.4	1.61	20.73	344
87	88370483	40	160.5	53.4	1.61	20.73	243
88	88358325	43	160.5	54.7	1.61	21.23	206
89	88370391	49	160.5	56.9	1.61	22.09	262
90	88370620	47	160.5	58.3	1.61	22.63	202
91	88367349	47	160.5	59.7	1.61	23.18	276
92	88256538	46	160.5	61.6	1.61	23.91	207
93	88370597	47	160.5	70.5	1.61	27.37	164
94	88343777	48	161	50.7	1.61	19.56	205
95	88343787	44	161	53.1	1.61	20.49	297
96	88370816	50	161	56.8	1.61	21.91	245
97	88343803	50	161	58.2	1.61	22.45	274
98	88343756	46	161	61.8	1.61	23.84	299
99	88366846	41	161	63	1.61	24.30	310
100	88367334	45	161	63.2	1.61	24.38	273
101	88343781	45	161.5	49.1	1.62	18.83	162
102	88269686	47	161.5	50.5	1.62	19.36	257
103	88370502	40	161.5	56.8	1.62	21.78	378
104	88363282	46	161.5	57.7	1.62	22.12	205
105	88369945	49	161.5	58.9	1.62	22.58	222
106	88370520	42	161.5	61.7	1.62	23.66	259
107	88391007	44	161.5	64.2	1.62	24.61	306
108	88366834	47	161.5	67.2	1.62	25.76	378
109	88343759	42	162	52.9	1.62	20.16	246
110	88366314	40	162	53	1.62	20.20	216
111	88367260	45	162	53.9	1.62	20.54	156
112	88370381	49	162	56.2	1.62	21.41	284
113	88370523	40	162	58	1.62	22.10	361
114	88421845	43	162	61.4	1.62	23.40	241
115	88340627	44	162	62.6	1.62	23.85	253
116	88370619	44	162	70	1.62	26.67	256
117	88421796	46	162	71	1.62	27.05	230
118	88343730	46	162	71.8	1.62	27.36	263
119	88370206	42	162	74.2	1.62	28.27	357

120	88421890	45	162.5	50.5	1.63	19.12	234
121	88343767	44	162.5	54.4	1.63	20.60	202
122	88370550	45	162.5	54.5	1.63	20.64	270
123	88370366	44	162.5	56.4	1.63	21.36	147
124	88367554	42	162.5	57.4	1.63	21.74	314
125	88167619	40	162.5	57.6	1.63	21.81	440
126	88363291	48	162.5	60.4	1.63	22.87	281
127	88363299	48	162.5	63.1	1.63	23.90	173
128	88370511	46	162.5	63.7	1.63	24.12	294
129	88370517	42	162.5	67.5	1.63	25.56	308
130	88279929	42	162.5	69.1	1.63	26.17	265
131	88343731	48	162.5	71.9	1.63	27.23	350
132	88370808	50	162.5	71.9	1.63	27.23	99
133	88269299	40	162.5	75.3	1.63	28.52	281
134	88370611	41	162.5	80.7	1.63	30.56	207
135	88421843	48	163	48.9	1.63	18.40	183
136	88343813	47	163	49	1.63	18.44	228
137	88399202	50	163	54	1.63	20.32	258
138	88370616	43	163	56	1.63	21.08	326
139	88370803	50	163	56.4	1.63	21.23	261
140	88269681	43	163	56.5	1.63	21.27	248
141	88421842	46	163	58.3	1.63	21.94	374
142	88343797	49	163	58.7	1.63	22.09	246
143	88370624	48	163	59.2	1.63	22.28	154
144	88363301	48	163	59.4	1.63	22.36	337
145	88343766	50	163	59.4	1.63	22.36	263
146	88413410	42	163	62.8	1.63	23.64	349
147	88311584	47	163	63.6	1.63	23.94	220
148	88322143	44	163	64.1	1.63	24.13	370
149	88370261	42	163	64.2	1.63	24.16	338
150	88301334	44	163	67.9	1.63	25.56	385
151	88370278	48	163	68.5	1.63	25.78	296
152	88421792	40	163	72.6	1.63	27.33	311
153	88309150	43	163	74	1.63	27.85	295
154	88366549	40	163	74.8	1.63	28.15	320
155	88343780	42	163	78.7	1.63	29.62	251
156	88370371	47	163	89.8	1.63	33.80	410
157	88370383	46	163.5	42.4	1.64	15.86	355
158	88370230	45	163.5	51.4	1.64	19.23	209
159	85919705	48	163.5	52.1	1.64	19.49	164

160	88366767	47	163.5	55.3	1.64	20.69	162
161	88269683	44	163.5	55.5	1.64	20.76	214
162	88369946	45	163.5	57.5	1.64	21.51	121
163	88370704	43	163.5	57.7	1.64	21.58	303
164	88343819	40	163.5	58	1.64	21.70	264
165	88269687	48	163.5	58.3	1.64	21.81	271
166	88343760	48	163.5	59.1	1.64	22.11	212
167	88370796	47	163.5	60.7	1.64	22.71	381
168	88370249	43	163.5	66.3	1.64	24.80	223
169	88358326	47	163.5	68.8	1.64	25.74	300
170	88370582	46	163.5	72.8	1.64	27.23	313
171	88390190	41	163.5	78.3	1.64	29.29	297
172	88370276	48	164	54.5	1.64	20.26	153
173	88366120	42	164	56.3	1.64	20.93	233
174	88343728	47	164	59	1.64	21.94	185
175	88173404	44	164	59.4	1.64	22.09	171
176	88279972	44	164	59.9	1.64	22.27	257
177	88370298	44	164	63.6	1.64	23.65	145
178	88366439	49	164	64	1.64	23.80	325
179	88421645	46	164	65.9	1.64	24.50	146
180	88370246	48	164	68.4	1.64	25.43	229
181	88363304	48	164	68.8	1.64	25.58	283
182	88370513	42	164	82.9	1.64	30.82	282
183	88367551	42	164.5	45	1.65	16.63	334
184	88367342	49	164.5	54.5	1.65	20.14	129
185	88370456	42	164.5	56.6	1.65	20.92	331
186	88362627	49	164.5	57.9	1.65	21.40	232
187	88367357	41	164.5	58.1	1.65	21.47	296
188	88370795	47	164.5	61.8	1.65	22.84	234
189	88343750	47	164.5	64.9	1.65	23.98	336
190	88343712	40	164.5	65.1	1.65	24.06	399
191	88370636	45	164.5	74.5	1.65	27.53	354
192	88343747	42	165	53.5	1.65	19.65	266
193	88420722	43	165	54.5	1.65	20.02	301
194	88343818	42	165	57.4	1.65	21.08	152
195	88366778	45	165	58.9	1.65	21.63	233
196	88370303	43	165	62.5	1.65	22.96	226
197	88369968	42	165	63.8	1.65	23.43	265
198	88361002	47	165	64	1.65	23.51	208
199	88370800	50	165	66.2	1.65	24.32	277

200	88279938	43	165	73.3	1.65	26.92	231
201	88370209	42	165	80	1.65	29.38	299
202	88413418	43	165	84.5	1.65	31.04	266
203	88361024	42	165.5	53.8	1.66	19.64	333
204	88343763	49	165.5	55.3	1.66	20.19	176
205	88343748	41	165.5	56	1.66	20.45	212
206	88370501	48	165.5	60.7	1.66	22.16	259
207	88367567	47	165.5	63	1.66	23.00	269
208	88343724	45	165.5	65.1	1.66	23.77	245
209	88369967	44	165.5	65.3	1.66	23.84	300
210	88370234	49	165.5	68.7	1.66	25.08	244
211	88280023	42	165.5	70.2	1.66	25.63	394
212	88343713	43	166	46.9	1.66	17.02	300
213	88370284	44	166	52.7	1.66	19.12	189
214	88421725	44	166	54.3	1.66	19.71	335
215	88370515	47	166	58.6	1.66	21.27	135
216	88343733	42	166	60.9	1.66	22.10	279
217	88370265	40	166	62.7	1.66	22.75	217
218	88358982	48	166	64.4	1.66	23.37	218
219	88366440	45	166.5	55	1.67	19.84	204
220	88421655	47	166.5	55.8	1.67	20.13	239
221	88366479	48	166.5	56.3	1.67	20.31	230
222	88370605	46	166.5	57	1.67	20.56	300
223	88369910	43	166.5	59.2	1.67	21.35	221
224	88367364	50	166.5	59.4	1.67	21.43	221
225	88343718	40	166.5	62.3	1.67	22.47	351
226	88366403	43	166.5	65.8	1.67	23.74	228
227	88343802	42	166.5	66	1.67	23.81	311
228	88370236	45	166.5	69.1	1.67	24.93	253
229	88366185	44	166.5	77	1.67	27.78	269
230	88370544	42	167	52.3	1.67	18.75	173
231	88343754	45	167	55	1.67	19.72	263
232	88421684	45	167	63.5	1.67	22.77	305
233	88366933	50	167	64.2	1.67	23.02	289
234	88375583	43	167	69.5	1.67	24.92	242
235	88366499	41	167	77.2	1.67	27.68	318
236	88370529	41	167.5	64.3	1.68	22.92	221
237	88269678	40	167.5	67.9	1.68	24.20	219
238	88421660	40	167.5	70.9	1.68	25.27	168
239	88369931	47	167.5	76.4	1.68	27.23	235

240	88343796	42	167.5	91.1	1.68	32.47	317
241	88413202	44	168	51.6	1.68	18.28	219
242	88369947	42	168	60.4	1.68	21.40	385
243	88363290	47	168	60.9	1.68	21.58	257
244	88279859	42	168	62.1	1.68	22.00	269
245	88343744	49	168	62.3	1.68	22.07	220
246	88173400	42	168	62.9	1.68	22.29	263
247	88369929	46	168	68.8	1.68	24.38	296
248	88374419	50	168	69	1.68	24.45	334
249	88279952	44	168	71.5	1.68	25.33	392
250	88279957	42	168	84.9	1.68	30.08	285
251	88343821	42	168.5	57.3	1.69	20.18	508
252	88343762	49	168.5	59	1.69	20.78	275
253	88343768	42	168.5	68.6	1.69	24.16	257
254	88420723	43	168.5	70.1	1.69	24.69	270
255	88421833	44	168.5	82.5	1.69	29.06	329
256	85274360		169	54	1.69	18.91	216
257	88370281	44	169	57.7	1.69	20.20	323
258	88343749	40	169	58.3	1.69	20.41	215
259	88370277	46	169	60.4	1.69	21.15	245
260	88366785	44	169	66.2	1.69	23.18	203
261	88343806	47	169	70.2	1.69	24.58	288
262	88343783	47	169	71.7	1.69	25.10	327
263	85817093	42	169	74.1	1.69	25.94	383
264	88370243	40	169	74.9	1.69	26.22	314
265	88366309	50	169.5	60	1.70	20.88	371
266	88366205	42	169.5	60	1.70	20.88	162
267	88370618	40	170	55.1	1.70	19.07	240
268	88370406	48	170	55.8	1.70	19.31	181
269	88370581	47	170	58.9	1.70	20.38	271
270	88343714	46	170	61.2	1.70	21.18	192
271	88363312	49	170	62.3	1.70	21.56	312
272	88370337	44	170	63.7	1.70	22.04	205
273	88370788	49	170	64.3	1.70	22.25	221
274	88370394	45	170	66.5	1.70	23.01	237
275	88343722	48	170	68.1	1.70	23.56	244
276	88343820	47	170	70.3	1.70	24.33	372
277	88369966	49	170	74.6	1.70	25.81	317
278	88370387	46	170	74.9	1.70	25.92	216
279	88370573	47	170	75.8	1.70	26.23	216

280	88370347	43	170	81.6	1.70	28.24	237
281	88370811	50	170.5	61.7	1.71	21.22	204
282	88370385	47	170.5	62.2	1.71	21.40	299
283	88356347	49	171	63.2	1.71	21.61	256
284	88370594	46	171	65.8	1.71	22.50	303
285	88350503	46	171	72.1	1.71	24.66	298
286	88366840	43	171	82.5	1.71	28.21	340
287	88343729	43	171	95.9	1.71	32.80	305
288	88413226	49	171	98.1	1.71	33.55	330
289	88370585	40	171.5	65.8	1.72	22.37	294
290	88370454	47	172.5	59.4	1.73	19.96	281
291	88418822	48	172.5	60.2	1.73	20.23	322
292	88394666	40	172.5	62.1	1.73	20.87	230
293	88370339	41	172.5	71.9	1.73	24.16	253
294	88369970	47	173	61.5	1.73	20.55	226
295	88370300	45	173	66.5	1.73	22.22	154
296	88370353	47	173	73.8	1.73	24.66	277
297	88343743	44	173.5	59.6	1.74	19.80	349
298	88366931	47	173.5	63.3	1.74	21.03	360
299	88366793	44	174	64.5	1.74	21.30	227
300	88350849	49	176	71	1.76	22.92	216
301	88370338	47	177	71.6	1.77	22.85	187
302	88370235	47	178	77.5	1.78	24.46	218
303	88370207	42	178.5	121.1	1.79	38.01	378

L	N	M	PLR	NLR
2.36	3.69	0.29	109.75	1.56
2.69	3.79	0.38	115.24	1.41
2.37	5.73	0.38	129.96	2.42
1.77	1.84	0.18	130.51	1.04
2.72	3.32	0.39	111.40	1.22
2.07	2.6	0.45	161.35	1.26
1.16	5.45	0.44	242.24	4.70
1.95	2.31	0.32	127.18	1.18
1.77	3.28	0.34	157.06	1.85
2.8	4.58	0.49	91.79	1.64
1.62	2.48	0.27	159.88	1.53
2.69	2.75	0.28	84.01	1.02
1.52	2.63	0.26	235.53	1.73
2.14	3.94	0.27	102.34	1.84
1.82	2.55	0.29	113.19	1.40
1.29	2.57	0.25	221.71	1.99
1.88	2.34	0.24	135.64	1.24
1.89	3.68	0.31	170.90	1.95
1.22	2.53	0.28	267.21	2.07
2.43	3.43	0.37	113.99	1.41
3.18	4.48	0.26	87.11	1.41
1.5	2.76	0.39	134.67	1.84
2.27	1.27	0.2	82.38	0.56
1.7	3.77	0.28	159.41	2.22
2.57	2.62	0.23	78.99	1.02
1.04	3.74	0.23	150.00	3.60
3.42	4.7	0.39	100.00	1.37
3.82	5.03	0.38	103.14	1.32
2.24	3.18	0.32	145.98	1.42
1.78	3.78	0.32	229.78	2.12
1.42	3.67	0.31	189.44	2.58
1.43	2.93	0.35	162.94	2.05
2.22	3	0.26	138.74	1.35
1.66	3.11	0.28	131.33	1.87
1.69	5.15	0.36	172.78	3.05
1.29	3.85	0.23	186.05	2.98
1.47	3.62	0.26	154.42	2.46
2.41	4.1	0.25	111.62	1.70
1.97	3.4	0.33	149.24	1.73

1.91	3.2	0.29	125.13	1.68
2.78	5.02	0.35	97.48	1.81
2.31	3.36	0.29	124.24	1.45
1.46	2.6	0.28	177.40	1.78
1.27	3.67	0.31	313.39	2.89
2.83	2.92	0.26	96.47	1.03
1.33	3.45	0.36	160.90	2.59
2.15	4.76	0.44	198.60	2.21
2.47	2.94	0.34	175.30	1.19
1.39	1.59	0.23	163.31	1.14
1.47	2.6	0.23	83.67	1.77
2.15	4.18	0.4	188.37	1.94
1.58	2.84	0.32	168.99	1.80
1.66	2.48	0.24	144.58	1.49
2	3.65	0.29	147.50	1.83
1.98	3.08	0.4	177.27	1.56
1.78	2.09	0.29	157.87	1.17
3.68	3.86	0.41	86.68	1.05
1.75	3.1	0.32	125.14	1.77
1.99	2.61	0.26	123.12	1.31
1.59	2.33	0.28	151.57	1.47
1.46	2.87	0.33	136.30	1.97
2.09	4.48	0.44	132.54	2.14
2.28	4.63	0.43	143.42	2.03
1.85	3.48	0.37	211.89	1.88
1.74	3.96	0.35	172.41	2.28
0.87	1.83	0.31	263.22	2.10
1.58	2.66	0.32	163.29	1.68
1.72	1.79	0.18	134.30	1.04
1.55	3.62	0.34	165.81	2.34
2.87	4.96	0.32	92.33	1.73
2.86	3.07	0.4	90.91	1.07
1.31	2.25	0.23	187.02	1.72
2.67	2	0.21	103.37	0.75
1.76	3.79	0.33	140.91	2.15
2.15	4.11	0.22	100.00	1.91
2.84	4.19	0.46	95.77	1.48
3.26	4.23	0.31	71.78	1.30
1.32	1.57	0.19	163.64	1.19
1.92	3.03	0.39	127.60	1.58

1.93	1.35	0.32	98.45	0.70
1.4	3.22	0.34	176.43	2.30
2.5	4.82	0.46	93.60	1.93
2.08	5.78	0.45	195.67	2.78
2.52	1.71	0.32	98.02	0.68
1.54	3.22	0.22	142.86	2.09
1.56	1.5	0.25	220.51	0.96
2.08	3.42	0.25	116.83	1.64
2.69	4.02	0.31	76.58	1.49
2.62	3.14	0.36	100.00	1.20
2.76	5.25	0.28	73.19	1.90
1.79	2.91	0.21	154.19	1.63
2.06	3.34	0.38	100.49	1.62
2.44	3.54	0.4	67.21	1.45
1.61	1.47	0.18	127.33	0.91
2.32	2.54	0.31	128.02	1.09
1.97	3.28	0.34	124.37	1.66
1.55	5.02	0.4	176.77	3.24
1.79	3.1	0.29	167.04	1.73
2.32	3.86	0.5	133.62	1.66
2.41	6.37	0.45	113.28	2.64
1.64	1.84	0.27	98.78	1.12
1.66	3.99	0.25	154.82	2.40
1.57	3.04	0.3	240.76	1.94
3.09	2.2	0.29	66.34	0.71
2.92	3.86	0.43	76.03	1.32
1.93	2.96	0.21	134.20	1.53
2.79	2.58	0.37	109.68	0.92
1.96	3.72	0.35	192.86	1.90
2.4	2.51	0.3	102.50	1.05
2.06	2.64	0.24	104.85	1.28
2.39	3.27	0.31	65.27	1.37
1.88	1.91	0.17	151.06	1.02
2.92	3.73	0.3	123.63	1.28
2.74	3.69	0.3	87.96	1.35
2.81	4.6	0.24	90.04	1.64
1.18	5.57	0.13	216.95	4.72
1.26	2.29	0.16	182.54	1.82
2.14	5.78	0.44	122.90	2.70
1.35	2.34	0.23	264.44	1.73

1.62	3.97	0.21	144.44	2.45
1.68	2.94	0.41	120.24	1.75
2.45	2.89	0.19	110.20	1.18
1.71	4.27	0.31	85.96	2.50
1.85	2.67	0.19	169.73	1.44
2.43	6.11	0.43	181.07	2.51
2.49	2.96	0.37	112.85	1.19
1.57	1.67	0.21	110.19	1.06
1.83	3.44	0.26	160.66	1.88
1.87	2.24	0.31	164.71	1.20
1.27	3.05	0.39	208.66	2.40
3.2	3.93	0.48	109.38	1.23
1.75	2.53	0.32	56.57	1.45
2.31	2.24	0.23	121.65	0.97
1.99	4.27	0.22	104.02	2.15
1.15	1.53	0.13	159.13	1.33
1.89	2.1	0.19	120.63	1.11
2.02	2.51	0.34	127.72	1.24
1.61	1.87	0.33	202.48	1.16
2.51	2.59	0.33	103.98	1.03
1.66	2.07	0.32	149.40	1.25
2.05	2.73	0.28	182.44	1.33
1.59	2.37	0.25	154.72	1.49
1.08	1.81	0.22	142.59	1.68
1.37	2.9	0.24	245.99	2.12
0.89	1.77	0.31	295.51	1.99
1.65	3.88	0.4	211.52	2.35
1.35	2.54	0.24	162.96	1.88
2.37	4.96	0.31	156.12	2.09
2.61	4.75	0.42	129.50	1.82
1.96	3.83	0.44	196.43	1.95
1.86	3.56	0.27	159.14	1.91
2.48	3.86	0.29	125.40	1.56
1.88	5.2	0.34	156.91	2.77
2.43	4.02	0.44	131.69	1.65
2.65	4.5	0.27	94.72	1.70
2.15	5.01	0.39	190.70	2.33
1.3	2.35	0.16	273.08	1.81
1.27	2.33	0.24	164.57	1.83
4.36	2.64	0.42	37.61	0.61

1.6	2.46	0.35	101.25	1.54
2.05	3.74	0.35	104.39	1.82
1.48	3.89	0.37	81.76	2.63
3.24	3.3	0.3	93.52	1.02
2.04	2.14	0.44	129.41	1.05
2.09	2.99	0.35	129.67	1.43
2.4	2.48	0.32	88.33	1.03
1.42	2.76	0.17	268.31	1.94
2.14	2.18	0.28	104.21	1.02
2.72	4.79	0.5	110.29	1.76
2.56	2.09	0.26	122.27	0.82
1.55	3.56	0.26	191.61	2.30
1.5	2.07	0.28	102.00	1.38
1.83	2.59	0.24	127.32	1.42
2.92	3.67	0.35	63.36	1.26
1.83	5.2	0.49	93.44	2.84
1.65	3.36	0.37	155.76	2.04
1.01	2.89	0.21	143.56	2.86
3.38	4.3	0.47	96.15	1.27
2.25	3.2	0.34	64.89	1.42
1.65	2.18	0.23	138.79	1.32
1.76	3.08	0.31	160.80	1.75
2.03	3.04	0.31	138.92	1.50
1.23	2.15	0.33	271.54	1.75
1.47	2.11	0.23	87.76	1.44
2.43	2.56	0.3	136.21	1.05
1.22	2.11	0.26	190.16	1.73
1.7	1.33	0.27	174.12	0.78
2.48	2.48	0.27	94.35	1.00
1.7	4.17	0.38	197.65	2.45
1.51	2.93	0.23	264.24	1.94
2.12	3.47	0.38	166.98	1.64
1.71	1.37	0.27	155.56	0.80
2.57	2.54	0.28	117.12	0.99
1.2	2.31	0.2	126.67	1.93
1.99	3.28	0.21	117.09	1.65
1.49	2.35	0.25	151.68	1.58
1.63	4.75	0.3	162.58	2.91
1.85	3.73	0.28	112.43	2.02
2.29	3.7	0.24	120.96	1.62

1.95	3.5	0.29	118.46	1.79
2.19	4.16	0.34	136.53	1.90
1.79	3.64	0.33	148.60	2.03
1.72	4.17	0.35	193.60	2.42
1.36	1.99	0.23	129.41	1.46
1.82	2.1	0.19	116.48	1.15
2.81	2.32	0.3	92.17	0.83
3.21	3.96	0.33	83.80	1.23
1.23	5.58	0.28	199.19	4.54
3.02	2.36	0.32	99.34	0.78
1.96	3.21	0.41	124.49	1.64
1.89	6.04	0.54	208.47	3.20
1.41	2.97	0.35	212.77	2.11
1.62	2.28	0.23	116.67	1.41
3.32	4.48	0.49	100.90	1.35
1.69	2.72	0.26	79.88	1.61
2.95	3.03	0.26	94.58	1.03
1.5	3.58	0.41	144.67	2.39
1.69	2.14	0.13	128.99	1.27
2.29	4.7	0.22	89.08	2.05
2.06	3.24	0.33	116.02	1.57
2.31	3.74	0.34	99.57	1.62
2.01	3.82	0.28	149.25	1.90
1.85	3.64	0.49	119.46	1.97
1.68	2.8	0.3	131.55	1.67
1.87	1.86	0.29	187.70	0.99
1.11	1.7	0.21	205.41	1.53
2.87	4.25	0.27	108.36	1.48
3.01	4.88	0.37	84.05	1.62
2.04	4.22	0.26	131.86	2.07
1.27	2.95	0.24	136.22	2.32
1.78	3.34	0.25	147.75	1.88
2.27	3.36	0.25	134.36	1.48
2.23	3.04	0.34	129.60	1.36
1.99	3.05	0.41	121.61	1.53
2.09	3.56	0.3	152.15	1.70
1.86	2.21	0.21	118.82	1.19
1.83	3.81	0.3	119.67	2.08
1.19	2.55	0.18	141.18	2.14
2.02	4.9	0.31	116.34	2.43

2.26	3.81	0.36	140.27	1.69
1.45	3.6	0.3	151.03	2.48
1.21	4.22	0.51	318.18	3.49
1.98	3.8	0.28	129.80	1.92
1.82	2.76	0.36	147.80	1.52
1.04	3.94	0.25	211.54	3.79
2.15	2.53	0.28	122.33	1.18
1.91	2.81	0.3	154.97	1.47
1.67	6.69	0.45	200.00	4.01
2.71	2.91	0.61	144.65	1.07
1.72	2.89	0.16	165.70	1.68
1.85	4.21	0.31	274.59	2.28
1.43	3.57	0.21	192.31	2.50
1.47	2.1	0.18	174.83	1.43
2.49	2.71	0.34	108.43	1.09
2.34	4.8	0.42	140.60	2.05
2.22	3.27	0.26	97.30	1.47
2.29	4.32	0.32	141.05	1.89
2.26	3.39	0.31	95.13	1.50
2.08	1.29	0.2	117.79	0.62
2.33	3.57	0.37	87.12	1.53
2.81	3.49	0.18	102.49	1.24
2.09	4.14	0.29	156.46	1.98
2.17	3.63	0.33	176.50	1.67
1.69	3.23	0.34	185.80	1.91
1.93	2.41	0.31	192.23	1.25
2.22	2.83	0.19	72.97	1.27
1.61	2.69	0.28	149.07	1.67
1.63	1.92	0.27	111.04	1.18
1.45	4.17	0.33	186.90	2.88
2.36	2.16	0.27	81.36	0.92
2.3	3.93	0.4	135.65	1.71
1.81	1.81	0.2	113.26	1.00
2.24	3.64	0.25	98.66	1.63
1.94	3.96	0.39	122.16	2.04
2.53	2.01	0.2	96.44	0.79
1.98	6.15	0.52	187.88	3.11
2.75	2.39	0.32	115.27	0.87
1.45	2.11	0.25	148.97	1.46
1.65	3.85	0.34	130.91	2.33

1.43	2.6	0.2	165.73	1.82
2.24	2.77	0.2	91.07	1.24
2.06	2.26	0.17	145.15	1.10
1.83	2.6	0.32	139.89	1.42
2.23	3.37	0.37	135.87	1.51
1.83	2.46	0.25	162.84	1.34
2.45	2.11	0.45	138.78	0.86
2.61	6.39	0.53	116.86	2.45
2.41	3.84	0.46	136.93	1.59
2.1	2.43	0.19	140.00	1.16
3.13	3.06	0.29	89.78	0.98
1.32	3.97	0.23	243.94	3.01
2.11	2.09	0.22	109.00	0.99
2.31	2.18	0.22	109.52	0.94
1.08	1.86	0.23	209.26	1.72
1.9	3.09	0.46	81.05	1.63
1.67	3.21	0.3	165.87	1.92
1.97	3.33	0.39	177.16	1.69
2.21	1.99	0.18	162.90	0.90
3.3	4.29	0.36	68.79	1.30
1.58	3.14	0.24	136.71	1.99
2.27	3.23	0.53	82.38	1.42
1.42	2.45	0.21	153.52	1.73
2.06	5.83	0.32	183.50	2.83

MLR	SII	HDL-C	LDL-C	VLDL-C	TG	TC
0.12	404.96	1.55	2.35	0.4	0.99	4.3
0.14	436.77	1.91	3.01	0.27	1.29	5.19
0.16	744.66	1.74	3.49	0.52	1.36	5.75
0.10	240.14	1.95	3.18	0.47	0.74	5.6
0.14	369.84	1.34	4.25	1.3	6.13	6.89
0.22	419.52	1.2	2.27	0.24	0.9	3.71
0.38	1320.22	1.17	1.84	0.2	1.03	3.21
0.16	293.78	1.92	3.52	0.63	1.87	6.07
0.19	515.16	1.1	3.27	0.63	2.4	5
0.18	420.38	1.76	3.36	0.42	0.76	5.54
0.17	396.49	1.76	4.44	0.22	0.79	6.42
0.10	231.04	1.41	3.36	0.62	1.77	5.39
0.17	619.43	1.27	2.44	0.33	0.91	4.04
0.13	403.21	1.86	3.27	0.74	1.5	5.87
0.16	288.63	1.55	2.33	0.36	1.15	4.24
0.19	569.78	1.42	3.9	0.6	1.67	5.92
0.13	317.39	1.98	2.68	0.44	0.71	5.1
0.16	628.91	1.86	2.9	0.47	1.01	5.23
0.23	676.05	1.66	2.21	0.32	0.8	4.19
0.15	390.99	2.04	3.6	0.43	0.6	6.07
0.08	390.24	1.36	4.37	0.52	1.91	6.25
0.26	371.68	2.07	1.7	0.29	0.55	4.06
0.09	104.62	1.78	3.45	0.62	1.51	5.85
0.16	600.98	1.61	3.09	0.51	1.61	5.21
0.09	206.95	1.43	2.94	0.38	1.08	4.75
0.22	561.00	1.08	3.28	0.39	1.19	4.75
0.11	470.00	1.21	3.16	0.24	1.46	4.61
0.10	518.80	1.49	4.12	0.54	1.3	6.15
0.14	464.22	1.21	3.3	0.58	2.03	5.09
0.18	868.55	1.35	3.76	0.42	1.14	5.53
0.22	695.23	2.09	2.78	0.47	0.84	5.34
0.24	477.41	2.04	2.31	0.41	0.46	4.76
0.12	416.22	1.84	1.82	0.2	0.8	3.86
0.17	408.42	1.38	3.34	0.71	1.14	5.43
0.21	889.82	1.99	3.88	0.8	1.41	6.67
0.18	716.28	1.32	2.03	0.17	0.68	3.52
0.18	559.01	1.2	3.47	0.53	1.46	5.2
0.10	457.63	1.58	3.75	0.47	1.53	5.8
0.17	507.41	1.69	1.84	0.34	0.79	3.87

0.15	400.42	1.96	2.62	0.31	0.8	4.89
0.13	489.36	1.35	2.89	0.79	3.24	5.03
0.13	417.45	2.44	3.19	0.54		6.17
0.19	461.23	1.42	2.5	0.32	0.6	4.24
0.24	1150.13	1.61	2.45	0.2	0.41	4.26
0.09	281.68	1.63	3.93	0.74	1.45	6.3
0.27	555.11	1.35	2.89	0.53	2.04	4.77
0.20	945.36	1.21	3.43	0.75	2.99	5.39
0.14	515.39	1.24	3.63	0.47	1.39	5.34
0.17	259.66	1.6	3.76	0.47	0.71	5.83
0.16	217.55	1.63	1.77	0.36	0.85	3.76
0.19	787.40	1.46	2.33	0.44	0.6	4.23
0.20	479.92	1.7	2.99	0.33	0.46	5.02
0.14	358.55	1.62	3.38	0.5	1.91	5.5
0.15	538.38	2.06	2.2	0.21	0.68	4.47
0.20	546.00	1.63	2.72	0.22	0.68	4.57
0.16	329.94	1.59	3.2	0.55	1.11	5.34
0.11	334.60	1.23	3.17	0.21	1.09	4.61
0.18	387.94	2.39	1.78	0.5	0.85	4.67
0.13	321.33	1.51	2.28	0.33	0.85	4.12
0.18	353.16	1.2	3.42	0.5	0.85	5.12
0.23	391.18	1.88	1.26	0.32	0.41	3.46
0.21	593.76	1.52	2.75	0.36	1.35	4.63
0.19	664.04	1.9	3.05	0.4	0.78	5.35
0.20	737.38	1.26	1.55	0.07	0.75	2.88
0.20	682.76	1.29	1.87	0.29	0.99	3.45
0.36	481.69	1.43	3.69	0.4	0.54	5.52
0.20	434.35	1.49	3.1	0.45	0.73	5.04
0.10	240.40	1.38	2.53	0.44	1.16	4.35
0.22	600.22	1.84	3.24	0.63	1.74	5.71
0.11	457.98	1.78	2.11	0.38	1.26	4.27
0.14	279.09	1.6	2.62	0.4	0.76	4.62
0.18	420.80	1.46	2.19	0.48	1.29	4.13
0.08	206.74	1.51	3.77	0.6	1.94	5.88
0.19	534.05	1.94	4.91	0.54	1.58	7.39
0.10	411.00	1.37	3.83	0.51	2.94	5.71
0.16	401.30	1.35	3.2	0.94	2.85	5.49
0.10	303.63	1.43	2.76	0.51	1.07	4.7
0.14	256.91	2.3	2.63	0.13	0.39	5.06
0.20	386.64	1.59	2.78	0.23	0.85	4.6

0.17	132.90	2.21	1.98	0.34	0.56	4.53
0.24	568.10	1.06	2.75	0.4	1.86	4.21
0.18	451.15	1.4	1.81	0.43	1.03	3.64
0.22	1130.99	1.65	3.32	0.33	0.69	5.3
0.13	167.61	1.73	2.99	0.63	1.09	5.35
0.14	460.00	1.85	2.08	0.28	0.65	4.21
0.16	330.77	1.36	2.5	0.29	1.17	4.15
0.12	399.55	1.57	3.09	0.48	0.8	5.14
0.12	307.85	1.29	3.21	0.9	3.2	5.4
0.14	314.00	1.96	3.13	0.29	0.53	5.38
0.10	384.24	1.28	3.28	0.34	1.35	4.9
0.12	448.69	2.19	2.09	0.33	0.66	4.61
0.18	335.62	1.09	2.19	1.1	4.02	4.38
0.16	237.93	1.53	3.26	0.74	1.31	5.53
0.11	187.17	1.89	1.9	0.39	0.87	4.18
0.13	325.16	1.81	2.06	0.42	0.68	4.29
0.17	407.92	1.8		0.65	1.09	5.72
0.26	887.41	1.63	3.15	0.28	0.95	5.06
0.16	517.82	1.07		0.42	1.01	4.79
0.22	515.78	1.56	2.03	0.4	0.78	3.99
0.19	721.58	1.8	2.7	0.42	1.08	4.92
0.16	181.76	1.73	2.56	0.4	0.74	4.69
0.15	617.73	1.52	3.63	0.71	1.62	5.86
0.19	731.92	1.15	2.89	0.31	1.32	4.35
0.09	145.95	1.57	3.56	0.44	1.17	5.57
0.15	293.47	1.85	2.73	0.22	1.22	4.8
0.11	397.22	1.58	3.11	0.35	0.91	5.04
0.13	282.97	1.21	2.38	0.42	2.34	4.01
0.18	717.43	1.76	2.84	0.39	0.64	4.99
0.13	257.28	1.32	2.53	0.25	0.81	4.1
0.12	276.82	1.53	2.4	0.41	0.97	4.34
0.13	213.44	1.31	2.35	0.24	1.01	3.9
0.09	288.53	1.66	3.06	0.45	0.87	5.17
0.10	461.14	1.37	3.41	0.28	0.73	5.06
0.11	324.56	1.2	3.46	0.64	2.85	5.3
0.09	414.16	1.29	2.65	0.29	1.05	4.23
0.11	1208.41	1.25	2.16	0.36	0.8	3.77
0.13	418.02	1.49	1.89	0.37	0.8	3.75
0.21	710.35	1.66	5.39	0.86	1.68	7.91
0.17	618.80	1.17	3.08	0.15	1.01	4.4

0.13	573.44	2.17	3.02	0.45	0.76	5.64
0.24	353.50	1.97	3.18	0.4	1.21	5.55
0.08	318.49	1.62	2.99	0.59	1.16	5.2
0.18	367.07	1.87	2.37	0.39	0.63	4.63
0.10	453.18	0.96	2.67	0.45	1.99	4.08
0.18	1106.34	1.42	3.15	0.3	1.6	4.87
0.15	334.04	1.62	3.13	0.46	0.8	5.21
0.13	184.02	1.53	2.17	0.44	0.43	4.14
0.14	552.66	1.99	2.53	0.38	0.7	4.9
0.17	368.94	1.82	1.87	0.2	0.71	3.89
0.31	636.42	1.61	2.01	0.16	0.55	3.78
0.15	429.84	1.89	4.43	0.75	1.35	7.07
0.18	143.13	1.72	2.82	0.42	0.58	4.96
0.10	272.48	1.51	2.76	0.28	0.99	4.55
0.11	444.17	1.45	2.71	0.48	1.16	4.64
0.11	243.47	1.73	2.23	0.27	0.38	4.23
0.10	253.33	1.55	3.87	0.59	1.44	6.01
0.17	320.58	2.22	2.57	0.62	0.88	5.41
0.20	378.65	1.4	2.24	0.21	0.48	3.85
0.13	269.32	2.15	3.03	0.63	0.79	5.81
0.19	309.25	1.42	1.83	0.34	0.56	3.59
0.14	498.06	1.47	2.03	0.16	0.43	3.66
0.16	366.68	1.32	2.46	0.35	0.57	4.13
0.20	258.09	1.46	2.1	0.22	0.54	3.78
0.18	713.36	1.51	2.92	0.32	0.47	4.75
0.35	523.04	1.67	3.41	0.57	0.91	5.65
0.24	820.68	1.45	3.47	0.52	1.53	5.44
0.18	413.93	2.29	3.97	0.41	0.77	6.67
0.13	774.35	1.72	3.1	0.3	0.96	5.12
0.16	615.13	1.94	2.79	0.42	0.87	5.15
0.22	752.32	1.1	2.21	0.44	1.54	3.75
0.15	566.54	1.47	3.2	0.57	1.33	5.24
0.12	484.06	1.51	2.2	0.39	1.43	4.1
0.18	815.96	1.44	3.61	0.38	1.37	5.43
0.18	529.38	1.05	3.38	0.49	1.8	4.92
0.10	426.23	1.34	2.92	0.46	1.28	4.72
0.18	955.40	1.16	2.74	0.21	1.34	4.11
0.12	641.73	1.44	2.94	0.57	1.11	4.95
0.19	383.44	1.75	2.58	0.34	0.69	4.67
0.10	99.30	2.5	3	0.68	0.9	6.18

0.22	249.08	1.39	2.67	0.48	1.33	4.54
0.17	390.42	1.43	2.66	0.24	0.84	4.33
0.25	318.03	1.65	2.49	0.4	1.93	4.54
0.09	308.61	1.31	1.91	0.65	2.95	3.87
0.22	276.94	1.49	3.56	0.55	1.64	5.6
0.17	387.70	1.66	3.01	0.64	1.14	5.31
0.13	219.07	1.74	3.25	0.5	0.68	5.49
0.12	740.54	1.21	2.96	0.21	0.59	4.38
0.13	227.17	1.23	3.23	0.5	1.43	4.96
0.18	528.31	1.23	2.8	3.27	11	7.3
0.10	255.54	1.22	3.15	0.44	2.41	4.81
0.17	682.14	1.83	2.75	0.52	2.29	5.1
0.19	211.14	1.77	2.74	0.35	1	4.86
0.13	329.77	1.49	2.54	0.19	0.53	4.22
0.12	232.52	1.44	3.51	0.52	1	5.47
0.27	485.90	1.49	2.65	0.4	0.87	4.54
0.22	523.35	2.01	2.79	0.57	1.1	5.37
0.21	414.90	1.56	1.92	0.25	1.06	3.73
0.14	413.46	1.19	2.49	0.46	1.91	4.14
0.15	207.64	1.13	2.73	0.61	2.91	4.47
0.14	302.56	1.29	2.88	0.4	1.83	4.57
0.18	495.25	1.53	3.61	0.25	0.72	5.39
0.15	422.31	1.35	2.66	0.22	1.35	4.23
0.27	583.82	2.21	2.15	0.4	0.5	4.76
0.16	185.16	1.33	3.54	0.49	0.95	5.36
0.12	348.71	1.23	2.22	0.19	1.23	3.64
0.21	401.25	1.65	2.42	0.36	0.69	4.43
0.16	231.58	1.45	3.96	0.4	0.91	5.81
0.11	234.00	1.24	3.26	0.65	1.67	5.15
0.22	824.19	1.83	3.12	0.74	1.83	5.69
0.15	774.22	1.47	3.09	0.57	0.98	5.13
0.18	579.42	1.12	2.26	0.35	1.72	3.73
0.16	213.11	1.97	2.16	0.37	0.59	4.5
0.11	297.49	1.84	1.88	0.28	0.61	4
0.17	292.60	1.62	1.72	0.33	0.66	3.67
0.11	384.04	1.87	2.88	0.59	1.1	5.34
0.17	356.44	1.39	1.96	0.3	1.05	3.65
0.18	772.24	1.42	3.67	0.37	1.83	5.46
0.15	419.37	1.57	3.67	0.64	1.02	5.88
0.10	447.55	1.9	3.96	0.3	1.06	6.16

0.15	414.62	1.12	2.49	0.3	1.06	3.91
0.16	567.96	1.13	2.58	0.62	2.66	4.33
0.18	540.92	0.97	2.74	0.36	1.88	4.07
0.20	807.33	1.53	2.44	0.25	0.74	4.22
0.17	257.53	1.56	3.22	0.37	0.62	5.15
0.10	244.62	2.01	2.01	0.37	0.44	4.39
0.11	213.84	1.58	2.8	0.34	0.94	4.72
0.10	331.85	1.56	2.43	0.32	0.83	4.31
0.23	1111.46	1.34	1.93	0.22	1	3.49
0.11	234.44	1.9	2.99	0.18	0.92	5.07
0.21	399.61	1.28	2.89	0.47	2	4.64
0.29	1259.13	1.2	2.31	0.53	2.56	4.04
0.25	631.91	1.34	2.67	0.47	0.89	4.48
0.14	266.00	1.38	2.09	0.29	0.79	3.76
0.15	452.05	1.17	2.88	0.42	1.71	4.47
0.15	217.28	1.42	3.09	0.73	2	5.24
0.09	286.57	1.59	3.58	0.46	0.73	5.63
0.27	517.91	0.97	3.21	0.47	1.51	4.65
0.08	276.05	0.91	2.94	0.37	1.34	4.22
0.10	418.69	1.58	2.42	0.34	0.55	4.34
0.16	375.90	1.58	3.62	0.67	2.05	5.87
0.15	372.38	1.78	2.21	0.49	0.83	4.48
0.14	570.15	1.91	2.66	0.33	0.54	4.9
0.26	434.83	1.46	2.64	0.15	0.91	4.25
0.18	368.33	1.35	3.32	0.46	1.31	5.13
0.16	349.12	1.44	2.81	0.26	0.61	4.51
0.19	349.19	1.33	2.56	0.17	0.91	4.06
0.09	460.54	1.03	3.22	0.51	2.06	4.76
0.12	410.18	1.14	3.04	0.56	2.19	4.74
0.13	556.46	1.2	3.71	0.75	1.91	5.66
0.19	401.85	1.98	2.9	0.52	0.87	5.4
0.14	493.49	1.44	2.45	0.38	0.58	4.27
0.11	451.45	1.27	3.92	0.36	1.24	5.55
0.15	393.97	1.48	2.87	0.33	1.06	4.68
0.21	370.90	1.59	2.66	0.32	0.99	4.57
0.14	541.67	1.72	3.13	0.49	0.86	5.34
0.11	262.59	1.84	1.1	0.4	0.65	3.34
0.16	455.95	1.93	1.99	0.37	0.62	4.29
0.15	360.00	1.81	1.33	0.22	0.49	3.36
0.15	570.05	1.13	3.08	0.7	2.33	4.91

0.16	534.41	1.78	2.98	0.47	1.82	5.23
0.21	543.72	2.32	2.27	0.33	0.87	4.92
0.42	1342.73	1.95	2.69	0.26	0.61	4.9
0.14	493.23	1.6	3.05	0.31	1.03	4.96
0.20	407.93	1.33	1.19	0.3	0.93	2.82
0.24	833.46	1.88	2.64	0.14	0.58	4.66
0.13	309.48	1.5	2.97	0.51	1.44	4.98
0.16	435.48	1.7	2.75	0.28	0.8	4.73
0.27	1338.00	1.01	2.57	0.74	1.93	4.32
0.23	420.93	1.71	2.95	0.37	1.14	5.03
0.09	478.87	1.24	2.92	0.45	1.01	4.61
0.17	1156.04	1.06	2.54	0.6	2.56	4.2
0.15	686.54	1.52	2.61	0.81	2.35	4.94
0.12	367.14	1.15	2.23	0.11	0.82	3.49
0.14	293.86	1.91	2.3	0.27	0.82	4.48
0.18	674.87	1.22	3.27	0.66	2.83	5.15
0.12	318.16	1.42	2.94	0.39	1.46	4.75
0.14	609.33	1.48	2.76	0.42	1.1	4.66
0.14	322.50	1.47	2.44	0.32	0.87	4.23
0.10	151.95	1.65	2.57	0.39	0.52	4.61
0.16	311.03	1.19	3.16	0.41	2.09	4.76
0.06	357.69	1.82	3.25	0.46	1.19	5.53
0.14	647.74	1.19	3.21	0.43	1.65	4.83
0.15	640.69	1.35	3.79	0.41	2.2	5.55
0.20	600.13	1.43	2.59	0.21	0.79	4.23
0.16	463.27	1.53	3.31	0.59	1.27	5.43
0.09	206.51	1.43	2.13	0.41	1.31	3.97
0.17	400.99	1.9	1.68	0.33	0.51	3.91
0.17	213.20	1.39	2.83	0.27	0.77	4.49
0.23	779.36	1.23	1.76	0.34	0.83	3.33
0.11	175.73	1.63	3.67	0.62	1.53	5.92
0.17	533.11	1.29	3.93	0.57	2.02	5.79
0.11	205.00	1.96	2.08	0.32	0.87	4.36
0.11	359.13	1.48	3.06	0.31	0.8	4.85
0.20	483.77	1.35	4.17	0.62	1.31	6.14
0.08	193.85	1.45	3.45	0.57	1.37	5.47
0.26	1155.45	1.37	4.34	0.69	1.61	6.4
0.12	275.50	1.32	2.88	0.24	0.63	4.44
0.17	314.32	1.63	2.44	0.46	1.11	4.53
0.21	504.00	1.41	3.23	0.71	2.02	5.35

0.14	430.91	1.14	2.48	0.41	1.75	4.03
0.09	252.27	1.5	2.67	0.39	0.62	4.56
0.08	328.03	1.02	2.43	0.68	2.29	4.13
0.17	363.72	1.56	2.15	0.25	0.81	3.96
0.17	457.90	1.33	3.47	0.52	1.65	5.32
0.14	400.59	1.34	2.5	0.65	1.85	4.49
0.18	292.82	1.12	3.61	0.48	2.44	5.21
0.20	746.72	1.65	2.53	0.37	1.39	4.55
0.19	525.81	1.51	4.2	0.47	1.54	6.18
0.09	340.20	1.3	1.61	0.58	1.89	3.49
0.09	274.72	1.57	3.26	1.04	1.63	5.87
0.17	968.44	2.16	2.06	0.44	0.39	4.66
0.10	227.82	1.94	3.04	0.49	0.62	5.47
0.10	238.76	1.8	3.1	0.69	0.83	5.59
0.21	389.22	1.4	3.23	0.27	0.85	4.9
0.24	250.45	1.41	1.62	0.2	0.48	3.23
0.18	532.44	0.99	2.52	0.64	2.74	4.15
0.20	589.93	1.39	4.34	1.04	1.42	6.77
0.08	324.16	1.82	2.72	0.27	0.58	4.81
0.11	295.10	1.27	2.89	0.23	0.92	4.39
0.15	429.27	1.32	2.97	0.54	1.18	4.83
0.23	266.08	1.9	2.37	0.31	0.49	4.58
0.15	376.13	1.98	2.74	0.39	0.7	5.11
0.16	1069.78	1.04	2.06	0.34	1.53	3.44
