# Supplementary figures and images for: Correlation between inflammatory marker and lipid metabolism in patients with uterine leiomyomas
Source: Front Med (Lausanne). 2023 May 3;10:1124697. doi: 10.3389/fmed.2023.1124697 (PMC10189017; doi:10.3389/fmed.2023.1124697)

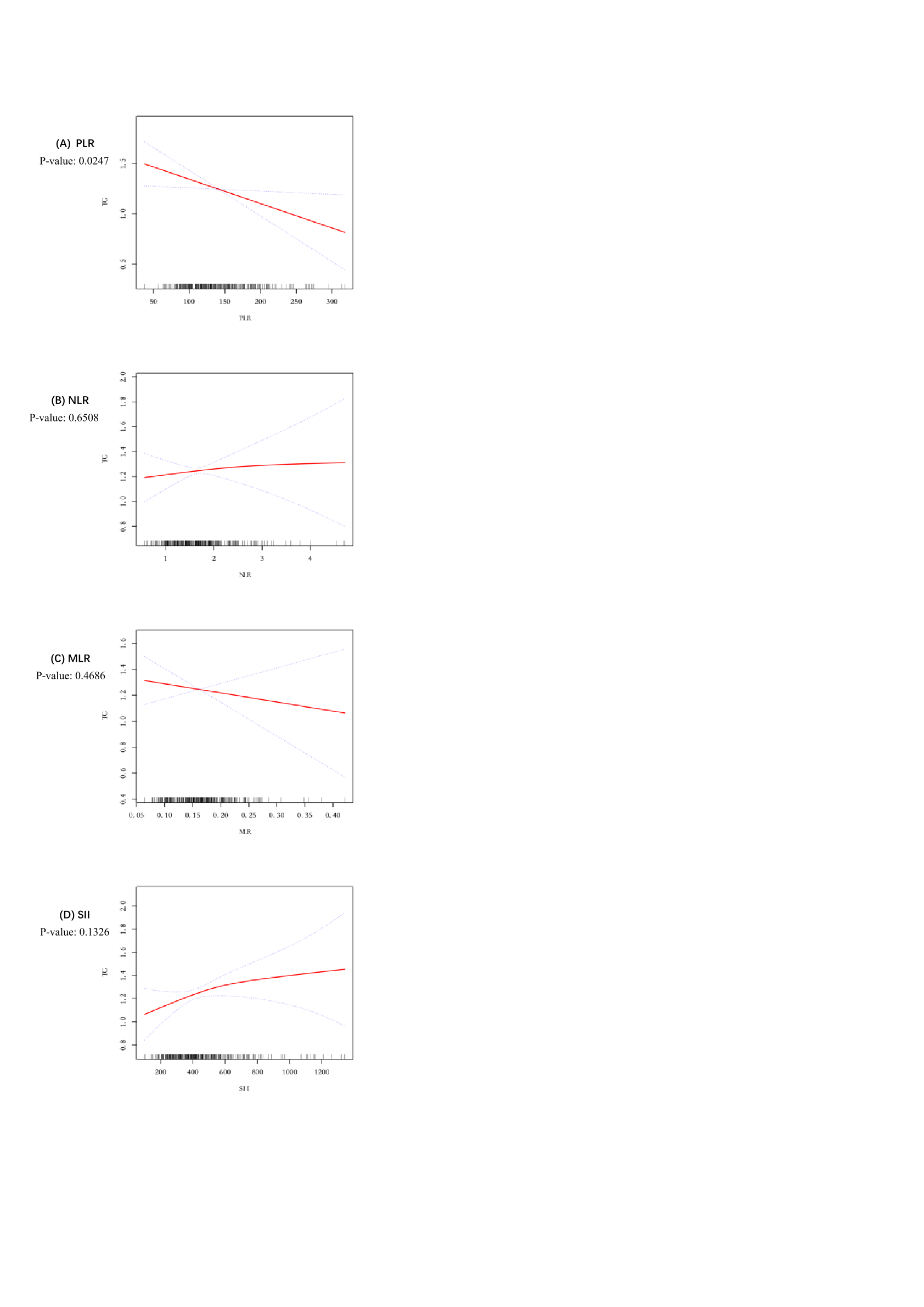

Supplement: Supplementary file 3 [file Image_1.tiff]
