# Supplementary material for: Correlation between inflammatory marker and lipid metabolism in patients with uterine leiomyomas
Source: Front Med (Lausanne). 2023 May 3;10:1124697. doi: 10.3389/fmed.2023.1124697 (PMC10189017; doi:10.3389/fmed.2023.1124697)
Supplement: Supplementary file 4 [file Table_1.docx]

**Supplementary Table 1.** Relationship between inflammatory markers and TG (mmol/L) in different models in healthy populations

| Variable | Crude Model  *β* (95% CI) P-value | Model I  *β* (95% CI) P-value |
| --- | --- | --- |
| PLR | -0.00 (-0.00, -0.00) 0.0202 | -0.00 (-0.00, -0.00) 0.0247 |
| NLR | 0.03 (-0.12, 0.18) 0.7013 | 0.04 (-0.12, 0.19) 0.6508 |
| MLR | -0.74 (-2.63, 1.15) 0.4418 | -0.70 (-2.60, 1.19) 0.4686 |
| SII | 0.00 (-0.00, 0.00) 0.1664 | 0.00 (-0.00, 0.00) 0.1326 |

Model I adjusted for age.
